# Psychometric Evidence of Instruments for Assessing Mental Health in Older Adults from Latin America and the Caribbean: A Scoping Review

**DOI:** 10.3390/healthcare14020265

**Published:** 2026-01-21

**Authors:** Claudia Miranda-Castillo, Stella-Maria Paddick, María O. León-Campos, Pedro Molleda, Javiera Rosell, Margarita Valenzuela

**Affiliations:** 1 Instituto de Investigación del Cuidado en Salud, Faculty of Nursing, Universidad Andres Bello, Santiago 8370035, Chile; 2 Millennium Institute for Care Research, Santiago 7820436, Chile; javiera.rosell@uchile.cl (J.R.); marga.valen.a@gmail.com (M.V.); 3 Translational and Clinical Research Institute, Newcastle University, Newcastle NE1 7RU, UK; stella-maria.paddick@newcastle.ac.uk; 4 M&L Salud Mental, Santiago 7500508, Chile; mariaolvidolc@gmail.com (M.O.L.-C.); p.molleda.c@gmail.com (P.M.); 5 Department of Psychology, Faculty of Social Sciences, Universidad de Chile, Santiago 7750000, Chile

**Keywords:** psychometric properties, screening tools, older adults, Latin America and the Caribbean, mental health

## Abstract

**Highlights:**

**What are the main findings?**

- Most studies assessing mental health in older adults from Latin America and the Caribbean focused primarily on cognitive function, with a predominance of research conducted in Brazil.
- There are a lack of standardized validity criteria, and few studies address psychosocial or emotional dimensions of mental health in aging.

**What are the implications of the main findings?**

- There is an urgent need to develop and validate culturally sensitive instruments that assess the broader spectrum of mental health in older adults.
- Expanding validated instruments across countries in the region would improve the quality and comparability of research and clinical practice.

**Abstract:**

Background/Objectives: Screening instruments are relevant for assessing the mental health of older adults, favoring clinical practice and research. However, there is a need for instruments with sufficient evidence of validity for use in Latin America and the Caribbean (LAC), where they are growing. This scoping review aims to evaluate the existing evidence regarding the psychometric properties of mental health measurement instruments used with older adults from LAC. Methods: Eight databases (PubMed, CINAHL, Medline, Embase, Scielo, Scopus, Web of Science, PsycINFO) were searched to extract relevant articles about instruments to measure mental health outcomes in older adults in English, Spanish, and Portuguese. After the first screening and duplicate removal, 6307 were retrieved. Results: The full-text screening identified 312 articles for inclusion in the review. Most of the articles were from Brazil (49.04%). One hundred eighty instruments (52.79%) assessed cognition, mainly using the Mini-Mental State Examination (MMSE), followed by 11.14% for depression, most commonly with the Yesavage Depression Scale (GDS). The assessment of validity was predominantly based on two criteria (34%), and 56% of the evidence was published in the last 10 years. To improve usability, we have provided a practical guide to help LAC clinicians and researchers identify and select robustly validated and contextually appropriate assessment instruments. Conclusions: There is still little evidence of the validity of instruments that measure other aspects of mental health beyond cognitive functioning. There seems to be no unified set of criteria in Latin America for determining whether an instrument has sufficient valid evidence. More work is needed on cross-cultural validity evidence within the region.

## 1. Introduction

According to the World Health Organization (WHO), one in six people worldwide will be aged 60 or over by 2030. It is estimated that around 14% of adults aged 70 or over will be living with a mental health disorder, accounting for 6.8% of the total years lived with disability for this age group [1]. Mental health conditions involve a broad aspect of pathologies, such as mental, neurological, and substance use disorders [2]. Without a doubt, mental health problems or common mental disorders impact the quality of life of people. This topic is especially relevant among older adults, who face age-related changes that require different coping strategies [3]. They are affected by social determinants such as stigma against mental illness and by inequity in mental health care [4]. In Latin America and the Caribbean (LAC), the prevalence rates of mental health disorders are higher than in countries in the Northern Hemisphere [5]. The timely recognition and treatment of mental health conditions in older adults is essential. However, according to the Pan American Health Organization (PAHO), the region’s average public expenditure on mental health is barely 2.0% of the health budget [6].

In this context, it is relevant to develop reliable and valid instruments to assess aspects of older adults’ mental health, such as mood, life satisfaction, and cognitive functioning. Assessment instruments are crucial for improving mental health interventions, especially in low and middle-income countries [7]. The correct use of screening instruments is beneficial in primary care settings, enabling health service professionals to make appropriate assessments and referrals for older adults [8,9].

The validity of an instrument is the extent to which the instrument captures the construct being measured, considering whether the evidence and theory support the interpretation of the instrument’s scores [10]. For this reason, validity is essential when developing tests, and specific standards must be met. This involves integrating everything, from the content to the social consequences of using the scores, ensuring they are embedded in a coherent context [11]. This is the only way to gather evidence that supports the interpretation of the instrument’s scores for a specific purpose [10].

It is crucial to note that evidence of an instrument’s validity in a particular context does not imply it will work similarly in other populations. It is incorrect to use an instrument validated in another country or for a different age range without evidence of validity in the actual context [10]. For example, in older adults, depressive disorders could appear with a somatic symptomatology rather than a depressive mood [12,13]. In addition, considerable differences exist across cultures, and clinical presentations may vary depending on the degree of assimilation, educational experience, acculturation, and cohort experiences [9]. Hence, it is essential to adapt and validate the screening instruments to ensure their applicability in specific settings (e.g., the older population in LAC). In this sense, it is crucial to clarify the current evidence on the validity of the instruments used to assess the mental health of older populations in LAC.

Despite the widespread use of mental health assessment instruments in older populations, there is limited synthesized evidence on which instruments have been validated for use in LAC and which psychometric properties have been evaluated in this context. In particular, it remains unclear which instruments are valid for assessing mental health outcomes in older adults in the region. Thus, the research question was: What evidence is available on the psychometric properties of instruments that assess mental health among older adults in Latin America and the Caribbean?

This scoping review aimed to assess the existing literature on measurement instruments used to identify mental health outcomes in older adults in LAC, with evidence of validity. The results are relevant to researchers, as they summarize the available evidence on different instruments in the region and could inform future studies in this area. This information could also help clinicians and healthcare professionals to select the most suitable instruments for evaluating older adults’ mental health, particularly in primary care settings in LAC.

This is the first review aimed at exploring and systematizing the instruments used for mental health assessment of older adults in the LAC.

## 2. Materials and Methods

To maintain the rigor of the review, we followed five steps. (1) identification of the research question; (2) search and identification of relevant studies according to our pre-defined inclusion criteria; (3) screening and selection of studies; (4) charting data; (5) reporting the results according to PRISMA guidelines [14]. **The study protocol was registered in the Open Science Framework (OSF) on 15 January 2026 (10.17605/OSF.IO/YFT2K) [15].**

**Eligibility Criteria**

The studies were selected from 1990 to April 2024. This period was chosen to include evidence that was up to date, given the rapid increase in the older population in Latin America, which should imply a constant update of the scientific evidence on the instruments to be adapted to the new realities of this age group.

The eligibility criteria were defined in accordance with the PCC framework (see Table 1). Articles were excluded if they did not fit the following inclusion and exclusion criteria.

The sources of information were formal databases that met the criteria of relevance and importance in the field of study. The search process was conducted in the region’s three main languages: Spanish, Portuguese, and English. The whole electronic search strategy, including all search terms and limits, is available in Supplementary Materials Document S1. An overview of the methodological stages and rigorous procedures followed in this review is presented in Table 2.

**Procedure**

**Figure 1:**
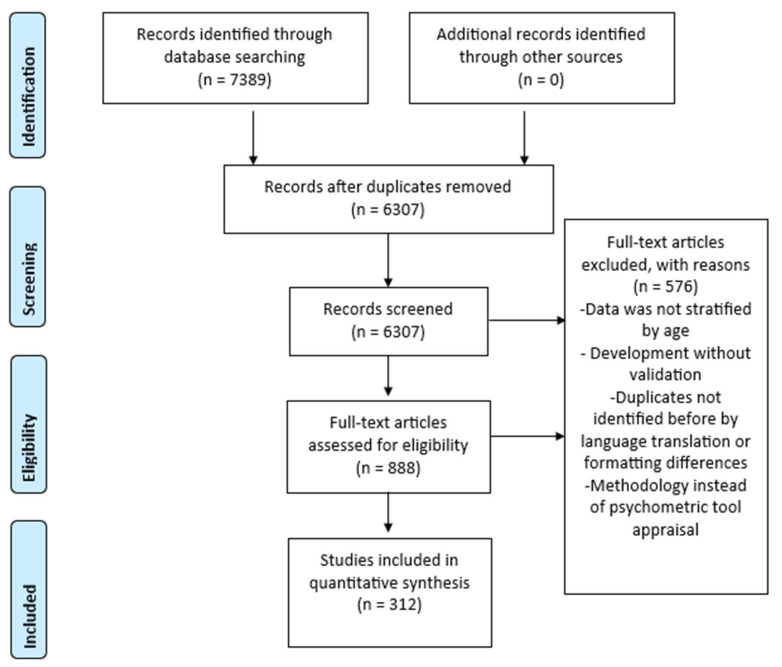
Flow Diagram.

To ensure consistency across languages, a glossary of search terms was developed in Spanish, English, and Portuguese (Supplementary Materials Document S2). Four reviewers (M.O.L.-C., P.M., S.-M.P., and M.V.) independently screened titles and abstracts using Rayyan QCRI, enabling a blind review process. Records with discrepancies in inclusion decisions were subsequently reviewed by a fifth senior researcher (C.M.-C.), who acted as an arbitrator. The study selection process is summarized in a PRISMA-ScR flow diagram (Figure 1).

Full-text screening and data extraction were conducted independently by the same four reviewers. A standardized data extraction form was initially drafted by two reviewers (M.O.L.-C. and P.M.) and subsequently reviewed and refined by two additional reviewers (C.M.-C. and S.-M.P.). The form was piloted using a subset of included studies to ensure its relevance and consistency with the review objectives. Following this pilot phase, no further modifications were required.

Extracted data were organized in a charting data (Supplementary Materials Document S3), including bibliographic information, study setting, sample characteristics, instrument features, and psychometric analyses (e.g., reliability, diagnostic accuracy, construct and/or criterion validity, and factor analysis). For analytical purposes, results were synthesized descriptively and grouped by country, publication year, instrument, purpose, and type of psychometric evidence. To facilitate future use of the identified instruments, the charting data were organized by country, allowing clinicians and researchers to locate relevant instruments readily.

## 3. Results

Following database searches, 7389 articles were identified, of which 6307 remained after duplicates were removed. Following title and abstract screening, 5419 records were excluded. Of the 888 articles selected for full-text review, a further 576 were excluded, leaving 312 studies included in the review (see Figure 1).

Figure 1 PRISMA-ScR flow diagram showing the identification, screening, and inclusion process of studies in the scoping review [14].

The charting data (Supplementary Materials Document S3) summarizes the research’s origin (author, country, city, setting), the characteristics of the sample and methodology, and the primary analyses of the validation. It also displays the details of the analyses carried out in each research and the figures used to validate each instrument.

To address the research question regarding the available psychometric evidence in LAC, the extracted data were synthesized into categories to enable a comprehensive mapping of the literature. The geographic distribution was analyzed to identify regional production patterns, the temporal trend to understand the field’s evolution, and the specific instruments and outcomes to detect areas of over-reliance (e.g., cognition) versus neglected dimensions. Finally, we categorized the types of psychometric analyses performed to evaluate how well regional research aligns with international validity and reliability standards.

### 3.1. Synthesis by Country and Temporal Distribution of Studies

The results revealed significant variations in the number of publications by country (see Table 3). Almost half of the included articles (49%) were conducted in Brazil. The majority of these publications were produced during the last decade (2014–2024), with 184 articles representing 60% of the total sample.

**Figure 2:**
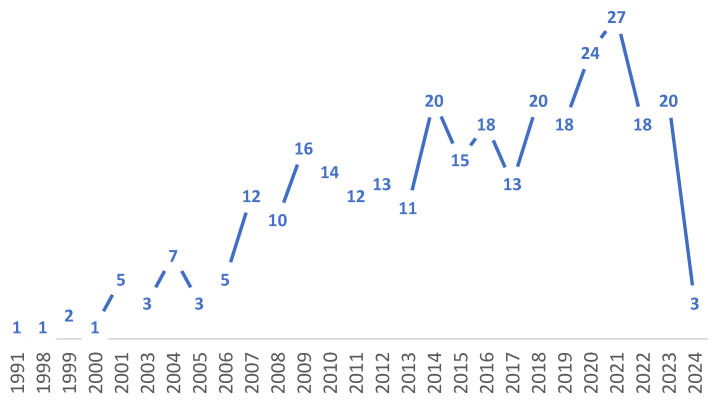
Number of articles by year of publication used to assess mental health in older adults from Latin America and the Caribbean.

The highest number of publications was observed in 2021, with 27 studies being published that year. Figure 2 illustrates the temporal distribution of these publications, between 1991 and 2024.

Figure 2 Annual distribution of included studies published between 1991 and April 2024 in the scoping review on psychometric instruments for mental health assessment in older adults from Latin America and the Caribbean.

### 3.2. Synthesis by Instrument and Outcome Measured

The scoping review identified a wide variety of assessment tools, with 179 different psychometric instruments found across the 312 included studies. When analyzing the relationship between these instruments and their primary purposes, it became clear that there was a strong focus on cognitive assessment, with this category representing 180 articles (52.7%) in the regional literature. This category encompasses all types of evaluation for dementia and other cognitive impairments.

Within this domain, the most commonly validated instrument was the Mini-Mental State Examination (MMSE) [17], appearing in 22 studies (6.4%), followed by the Montreal Cognitive Assessment (MoCA) [18] in 18 studies (5.2%), and the Addenbrooke’s Cognitive Examination (ACE) [19] in 14 studies (4.1%). Other frequently used instruments for cognitive screening included the Clock Drawing Test (CDT) [20] (9; 2.6%), the Informant Questionnaire on Cognitive Decline in the Elderly (IQCODE) [21] (6; 1.7%), and the Rowland Universal Dementia Assessment Scale (RUDAS) [22] (4; 1.1%).

Depression was identified as the second most frequently studied outcome (38 articles, 11.1%). The primary instrument used for this purpose was the Geriatric Depression Scale (GDS) in its various formats [23], which was validated in 21 studies (6.1%). This was followed by the Center for Epidemiologic Studies Depression Scale (CES-D) [24], which was found in four articles.

Quality of life was assessed in 34 articles (9.9%). In this category, the World Health Organization Quality of Life (WHOQOL) [25] was the most frequently used (13 studies), alongside the Short Form Health Survey Questionnaire (SF-36) [26] (7 studies) (See Supplementary Materials Document S4, Tables S1 and S2).

Finally, the review identified several less-explored psychosocial dimensions. Spirituality was the focus of seven articles (2%), while loneliness was assessed in six articles (1.7%). Other outcomes, including anxiety, life satisfaction, mistreatment, resilience, and social support, were each examined in only four studies (1.1%). The remaining instruments were only mentioned in three or fewer studies across the review.

### 3.3. Synthesis by Type of Analysis Performed to Validate Instruments

Regarding the analyses conducted in the reviewed articles, one-third (34%) assessed the validity of the instruments using two different criteria. Reliability was the most frequently analysed aspect in 201 studies (64.4%) employing intra- and inter-class correlation, internal consistency, and test–retest reliability. One hundred sixty-five (52.9%) articles included convergent, concurrent, and predictive validity. Sensitivity, specificity, cut-off points, and area under the curve (AUC) were reported in 149 (47.8%) studies. Factor analysis was used in 110 articles (35.3%), followed by adaptation (46, 14.7%), translation (40, 12.8%), divergent validity (30, 9.6%), and normative data (15, 4.8%). Rasch analysis, identified as a new psychometric approach, was reported in only 13 studies (4.2%). The development process of new instruments was discussed in 11 articles (3.5%) (Supplementary Materials Documents S4, Table S3).

### 3.4. Practical Guide for Clinicians and Researchers: Using the Charting Data in Supplementary Materials Document S3

To enhance the practical applicability of the findings, the charting data (Supplementary Materials Document S3) was structured as a decision-support and research-planning instrument to assist health professionals and academics in LAC. This data can be used as follows:

1. Country-based selection: Clinicians should first identify instruments validated in their country or in culturally similar settings, as psychometric properties are context-dependent and not universally transferable.
2. Outcome-oriented filtering: The “Purpose” column allows users to identify instruments according to the clinical or research outcome of interest (e.g., cognitive functioning, depressive symptoms, quality of life).
3. Assessment of psychometric robustness: The “Analyses performed” column enables evaluation of the strength of validity evidence. Instruments supported by multiple sources of evidence (e.g., reliability, validity, diagnostic accuracy) should be prioritized over those based on a single analysis.
4. Population and setting alignment: Information on sample characteristics and recruitment settings (e.g., urban or rural) allows clinicians to assess the similarity between the validation sample and their own patient population, which is particularly relevant in the heterogeneous LAC context.

This guide can also be used by researchers to select valid instruments for their studies and to identify areas of mental health assessment that need more evidence.

## 4. Discussion

This scoping review identified 312 validation studies of instruments measuring mental health and various psychosocial outcomes among older adults in LAC. These studies reveal significant geographical and thematic imbalances in the available psychometric evidence for older adults in the LAC region. To our knowledge, this is the first review to systematically map and synthesise psychometric evidence on mental health assessment instruments for older adults in the LAC region. While previous reviews have examined individual instruments [27] or focused on specific conditions [28], none have provided a comprehensive regional overview of validated instruments and their psychometric properties in this population.

A marked geographic concentration of research was observed, with nearly half of the studies conducted in Brazil. Although this is partly understandable given Brazil’s larger population size, the difference exceeds what would be expected based on population size alone. One possible explanation is that Brazilian researchers have developed a stronger local culture of translating and adapting instruments into Portuguese. In contrast, Spanish-speaking LAC countries frequently rely on instruments validated in Spain. This reliance on external validations poses a risk to clinical accuracy, as instruments used in LAC must be sensitive to the region’s structural inequalities, urban–rural realities, and racial and ethnic diversity [29]. Despite this, only 14.7% of the studies reported conducting cultural adaptations of instruments, and only 12.8% included translation processes. In LAC, mental health, socioeconomic status, and lifestyle factors significantly influence healthy aging and cognitive functioning in older adults. These factors, combined with widespread income inequality, highlight the importance of using valid and context-specific instruments for effective interventions [4,30].

In terms of thematic focus, this scoping review identified 179 distinct psychometric instruments. However, a clear trend emerged towards assessing cognitive performance, accounting for 52.79% of the identified studies. This aligns with the global urgency of addressing population ageing. According to the WHO, the number of people living with dementia is projected to rise from 55 million in 2019 to 139 million by 2050 [31]. The situation in LAC is even more pressing, with an estimated 4.5 million cases in 2019 expected to rise to 13.7 million by 2050, a 205% increase [32]. Timely diagnosis and treatment are essential for improving quality of life [33].

However, while the timely diagnosis of neurocognitive disorders is essential, cognition represents only one aspect of mental health. The mental health of older adults should be understood as a multidimensional construct, with cognitive status clearly distinguished from other essential domains, such as emotional, spiritual, and psychosocial well-being, and affective state. Our findings suggest that these non-cognitive domains are significantly underrepresented in regional validation efforts. For instance, instruments assessing depression, loneliness, and anxiety accounted for just 11.14%, 1.76%, and 1.17% of studies, respectively. Given the high global prevalence of these conditions [1,34,35,36,37], which has been exacerbated further by the pandemic [38,39], there is a critical need to validate reliable instruments that address the full range of mental health issues in the region [40,41,42,43], ensuring a holistic approach to ageing.

Furthermore, to advance research into the phenomena experienced by older adults, other psychosocial aspects should be assessed using appropriate measures. For instance, positive factors such as resilience, coping skills, self-efficacy, and optimism could mitigate adverse outcomes [44,45,46], yet these have rarely been considered in instrument validation studies in LAC.

Validity should not be understood as a static property of an instrument, but rather as the accumulation of scientific evidence that supports the use of instruments in a specific setting [10]. Since no screening instrument has universal applicability, the selection of instruments in Latin America and the Caribbean must be based on critical evaluation, ensuring that the measurements truly reflect the cultural and linguistic characteristics of the population being assessed. This approach avoids relying on external instruments and promotes the use of accumulated evidence to ensure accurate interpretations across diverse regional contexts [47].

The analyses across the articles were heterogeneous, suggesting that there are no clear, unified criteria for determining the validity of a psychometric instrument in the LAC region. To strengthen the interpretation of these findings, it is crucial to ground them in well-established frameworks, such as the Standards for Educational and Psychological Testing [10]. According to these standards, validity is not an inherent property of a test, but rather an argument based on accumulated evidence that supports the interpretation of scores for a specific purpose.

Our findings suggest that the validity of mental health assessment instruments in LAC is still primarily based on classical test theory (CTT). In contrast, approaches based on item response theory (IRT), including Rasch analysis, are only represented in a small proportion of the reviewed studies. IRT and Rasch models have several advantages over CTT. These include more detailed item-level diagnostics, independence of item parameters from the sample, and the ability to create linear and interval-level scales. This provides more substantial evidence for instrument validity [48]. This highlights the need to expand the use of advanced psychometric methods in future regional validation studies.

Therefore, there is no single “gold standard” for declaring an instrument valid. Instead, validation requires multiple sources of evidence, including reliability (i.e., consistency of scores), evidence supporting the intended interpretations and uses of scores, and fairness (i.e., equivalence of use across groups) [49,50].

Our mapping of the reported analyses reveals an emphasis on specific sources of validity evidence, while others are underrepresented.

- Evidence based on internal structure: Although factor analysis was reported in 35.3% of studies to provide evidence of internal structure, the low frequency of divergent validity (9.6%) suggests that little attention was paid to establishing the distinctiveness of the measured constructs.
- Evidence based on relations to other variables: A substantial proportion of studies (47.8%) focused on diagnostic accuracy, offering evidence based on relations to other variables, such as criterion validity, sensitivity, and specificity.
- Reliability evidence: Although reliability was the most frequently reported analysis (64.4%), it primarily reflects internal consistency and stability. While these are necessary, they are insufficient on their own to support a comprehensive validity argument.

It is concerning that 21% of studies relied on a single analysis to justify the use of an instrument. Robust validation requires multiple sources of evidence, including content-related evidence and considerations of fairness, both of which remain underreported in the region. Aligning future research in LAC with international standards would enable validation practices to evolve from single-metric reporting to a more comprehensive and theoretically coherent evidence base. This scoping review is a first step towards achieving this objective.

In the context of an ageing population, particularly in the LAC region, it is crucial to gather evidence on the effectiveness of screening instruments for evaluating the mental health of older adults. This information must continually be updated due to the heterogeneity of this age group and generational changes (for example, older adults from the 1960s differ from those born in 2025).

Undoubtedly, using validated instruments can reduce costs and the overuse of health services by enabling adequate evaluation, intervention, and case management for older patients [51]. Screening for mental health issues can serve the valuable purpose of identifying the need for further investigation by relevant staff and, ideally, triggering appropriate treatment responses [52]. Finally, more evidence is needed regarding the cost-effectiveness of screening tools, particularly in the LAC region [53].

**Limitations and future research**

One limitation of this review is the search strategy, which may have excluded validation studies in the general population that included older individuals.

Additionally, the search intentionally excluded gray literature such as dissertations, theses, conference proceedings, and government reports. While this approach ensures that the summarized evidence meets the standards of peer-reviewed publication, it is possible that additional validation studies or emerging instruments used in local clinical settings were missed. Furthermore, although this review followed the five-step framework for scoping reviews to map the evidence, no formal quality assessment of the individual studies was performed, which is consistent with the mapping objectives of this methodology.

Future research could consider other relevant outcomes and explore instruments with evidence of validity in older populations across different countries in the region. In addition, researchers from LAC should strengthen collaborative networks to develop cross-cultural research on the assessment of mental health and the psychosocial aspects of aging. Specifically, future research should:

1. Expand the scope of validation to include affective, psychosocial, and positive mental health constructs (e.g., depression, loneliness, resilience, coping, and well-being), taking a multi-dimensional approach to mental health in older adults.
2. Apply advanced psychometric approaches, such as item response theory and Rasch models, to improve the precision, invariance, and fairness of measurements across diverse contexts.
3. Include underrepresented populations (e.g., rural, indigenous, and socioeconomically marginalized older adults) to ensure that the instruments are valid, equitable, and contextually appropriate.
4. Prioritize cultural adaptation over simple translation to ensure that instruments are sensitive to the linguistic, cultural, and socio-economic heterogeneity of the ageing population.
5. Move away from single-metric reports towards comprehensive validity arguments that incorporate multiple sources of evidence, including content and fairness evidence.
6. Strengthen collaborative regional networks to develop cross-cultural research, moving away from isolated national studies towards shared regional standards.

## 5. Conclusions

The validation studies of psychometric instruments for mental health problems of the older population in LAC have increased in recent years. However, there appears to be no unified criterion for declaring that an instrument has sufficient evidence for its use. There is a need for more research across the region and for the diversification of the variables measured for validation. Finally, future studies should adopt rigorous methodologies when validating psychometric instruments.

## Figures and Tables

**Table 1:** Summary of Inclusion and Exclusion Criteria.

Category	Inclusion Criteria	Exclusion Criteria
Population	Older adults (aged 60+ or mean age ≥ 60).	Samples not stratified by age or younger populations.
Concept	Psychometric instruments assessing mental health disorders or psychosocial outcomes relevant to mental health in older adults (e.g., cognition, depression, anxiety, resilience, quality of life).	Studies not addressing mental health assessment, focusing solely on physical health outcomes, or describing instruments without a clear link to mental health.
Context	Latin America and the Caribbean (LAC) region.	Studies conducted outside the LAC region, or with mixed samples where LAC-specific data were not reported.
Study Design	Studies reporting at least one psychometric property, including adaptation, translation, development, reliability, validity, diagnostic accuracy, factor structure, or normative data.	Instrument development studies without any psychometric evaluation or validation-related analyses.
Sources	Peer-reviewed articles published between 1990 and 2024 in English, Spanish, or Portuguese.	Duplicates, conference abstracts, editorials, reviews, or studies published in languages other than those specified.

**Table 2:** Methodological stages and rigorous procedures [14].

Stage (PRISMA-ScR)	Actions and Rigor Safeguards
1. Research question identification	The research question was defined using the PCC (Population, Concept, Context) framework, with a focus on the psychometric evidence of mental health assessment instruments for older adults in LAC.
2. Identification of relevant studies	A comprehensive search was conducted in the following formal databases: PubMed, CINAHL, Medline, Embase, SciELO, Scopus, Web of Science, and PsycINFO. Searches were performed in Spanish, Portuguese, and English using a trilingual glossary. The whole search strategy and limits are reported in Supplementary Materials Document S1.
3. Study screening and selection	The title and abstract were screened independently by four reviewers using the Rayyan QCRI tool in a blind process. Any discrepancies were resolved by a fifth senior reviewer acting as arbitrator [16]. The selection process is summarised in a PRISMA-ScR flow diagram (Figure 1).
4. Data charting and extraction	A standardised data extraction form was developed by two reviewers and refined by two more. The form was piloted on selected studies to ensure consistency and relevance before complete data extraction.
5. Collating, summarizing, and reporting results	The data were organised into a charting extraction (see Supplementary Materials Document S3), which included study characteristics, sample details, instrument features, and psychometric analyses. The results were synthesized descriptively and analytically, and organised by country, instrument, purpose, and type of psychometric evidence.

**Table 3:** Country frequency of studies used to assess mental health in older adults from Latin America and the Caribbean.

Country	n	%
Brazil	153	49.04
Chile	36	11.54
Peru	31	9.94
Mexico	30	9.62
Colombia	20	6.41
Argentina	19	6.09
LA	12	3.85
Cuba	3	0.96
Costa Rica	2	0.64
Ecuador	2	0.64
Venezuela	2	0.64
Peru and Spain	1	0.32
Spain, Cuba, and Colombia	1	0.32

Values represent the number (n) and percentage (%) of studies conducted in each country among all included studies.

## Data Availability

No new data were generated in this study.

## Supplementary Materials

The following supporting information can be downloaded at: https://www.mdpi.com/article/10.3390/healthcare14020265/s1, Document S1: Search strategy; Document S2: Glossary of the search; Document S3: Charting data extraction; Document S4: Table S1: Frequency of the psychometric tool; Table S2: Frequency of psychometric instrument purpose; Table S3: Frequency of types of analyses.
