# Supplementary material for: Psychometric Evidence of Instruments for Assessing Mental Health in Older Adults from Latin America and the Caribbean: A Scoping Review
Source: Healthcare (Basel). 2026 Jan 21;14(2):265. doi: 10.3390/healthcare14020265 (PMC12841404; doi:10.3390/healthcare14020265)
Supplement: Supplementary file 1 [file healthcare-14-00265-s001.zip › 2_Supplemental Material S1_Search Strategyv2.pdf]

Supplemental Material S1 for:

# Psychometric evidence of instruments for assessing mental health in older adults from Latin America and the Caribbean: A scoping review.

This supplemental material presents the complete search strategies used in PubMed, CINAHL, MEDLINE, Embase, SciELO, Scopus, Web of Science and PsycINFO. The basic strategy was adapted to the syntax and indexing requirements of each database. Due to the need for simplification in the SciELO database, its adapted search strategy is presented.

**Limits applied:** articles published between 1990 and 2024.

**Date last searched:** April 2024

**English search strategy:**

(screening OR "mass screening" OR screen* OR "case finding" OR "geriatric assessment" OR "symptom assessment" OR diagnog*)

AND

(aged OR "aged, 80 and over" OR elder* OR aging OR "older adults" OR "aged 60")

AND

("latin america" OR peru OR colombia OR ecuador OR chile OR "south america" OR "central america" OR caribbean OR mexico OR argentina OR paraguay OR uruguay OR surinam OR guyana OR "french guiana" OR belize OR "el salvador" OR "costa rica" OR honduras OR nicaragua OR guatemala OR panama OR venezuela OR cuba OR "dominican republic" OR haiti OR guadeloupe OR martinique OR "saint martin" OR "saint barthelemy" OR "saint pierre")

AND

(validation OR adaptation OR accuracy OR sensitivity OR specificity OR "neuropsychological test*" OR "observer variation" OR "psychometrics" OR "translation" OR "reproducibility of results" OR "area under curve" OR "ROC curve" OR "factor analysis" OR "procedures techniques utili?ation" OR "diagnostic techniques procedures" OR "culturally competent care" OR "psychometrics instrumentation")

AND

(depress* OR "mood disorder" OR anxiety OR "stress disorder*" OR "post-traumatic stress disorder*" OR "psychological stress" OR "acute traumatic stress disorder*" OR frailty OR "frail elder*" OR "cognition disorder*" OR "neurocognitive disorder*" OR "mental disorder*" OR neurocognitiv* OR "cognitive decline" OR "cognitive dysfunction*" OR MCI OR "cognitive imparment*" OR dementia OR "frontotemporal dementia" OR "vascular dementia" OR "multi-infarct dementia" OR "alzheimer disease" OR alzheimer* OR "mental disorder*" OR "psychiatric disorder*")

OR

(loneliness OR "social capital" OR "social support" OR "mental health" OR wellbeing OR "quality of life" OR happiness OR resilience OR "psychological adaptation" OR coping OR satisfaction OR "activities of daily living")

**Spanish search estrategy**

cribado OR tamizaje OR "cribado masivo" OR screen* OR "busqueda de casos" OR "evaluacion geriatrica" OR "evaluacion sintoma*" OR diagnos* OR identificacion

AND

"tercera edad" OR anciano* OR "personas mayores" OR envejecimiento OR "adulto* mayor*" OR "edad sobre 60" OR vejez OR geria*

AND

latinoamerica OR "america latina" OR "am?rica del sur" OR "sudamerica" OR "centroamerica" OR "america central" OR caribe OR peru OR colombia OR ecuador OR chile OR me?ico OR argentina OR paraguay OR uruguay OR surinam OR guyana OR "guayana francesa" OR belice OR "el salvador" OR "costa rica" OR honduras OR nicaragua OR guatemala OR panama OR venezuela OR cuba OR "republica dominicana" OR haiti OR guadalupe OR martinica OR "san martin" OR "san bartolome" OR "san pedro y miquelon"

AND

"estado mental" OR "test de demencia" OR validacion OR adaptacion OR precision OR sensibilidad OR especificidad OR "prueba* neuropsicologica*" OR "variabilidad intra-observador*" OR "variabilidad inter-observador*" OR "psicometrica*" OR traduccion OR "reproducibilidad" OR "área bajo la curva" OR "curva roc" OR "analisis factorial*" OR "tecnica* diagnostica*" OR "culturalmente competente" OR "instrumentacion psicometrica" OR "instrumento* psicometrico*"

AND

(depresi* OR "trastorno* del animo" OR ansiedad OR "trastorno ansioso*" OR "estres postraumatico*" OR "estres psicologico" OR "estres agudo*" OR fragil* OR "trastorno* cognitivo*" OR "trastorno* neurocognitivo*" OR "trastorno* mental*" OR "enfermedad* psiquiatrica*" OR "trastorno psiquiatrico" OR neurocognitiv* OR "deterioro* cognitivo*" OR DCL OR "deterioro cognitivo leve" OR demencia OR alzheimer*)

OR

(soledad OR "capital social" OR "apoyo social" OR "salud mental" OR bienestar OR "calidad de vida" OR felicidad OR resiliencia OR "adaptación psicológica" OR afrontamiento OR satisfacción OR "actividades cotidianas")

**Portuguese Search strategy**

(psicometri* OR escala OR questionário OR inventário OR teste OR triagem OR "rastreio em massa" OR "pesquisa de caso" OR "avaliação geriátrica" OR "avaliação dos sintomas" OR diagnost* OR identificação OR medida* OR ferramenta*)

AND (envelhecido* OR "terceira idade" OR antigo* OR "pessoa maior" OR "pessoas maiores" OR envelhecimento OR "adulto mais velho" or "idosos" OR "idade acima de 60" OR velhice OR geriatr* OR "dade acima de 80")

AND ("america latina" OR "america do sul" OR "america central" OR caribe OR peru OR colômbia OR equador OR chile OR brasil OR mejico OR mexico OR argentina OR paraguai OR uruguai OR suriname OR guiana OR "guiana francesa" OR belize OR "o salvador" OR "costa rica" OR honduras OR nicarágua OR guatemala OR panama OR venezuela OR cuba OR "republica dominicana" OR haiti OR guadalupe OR martinica OR "são martim" OR "san bartolomeu" OR "san pedro e miquelon")

AND (validação OR adaptação OR precisão OR especificidade OR fiabilidade OR "validade de critério" OR "variabilidade intra-observador" OR "variabilidade interobservador" OR "confiabilidade interobservador" OR "confiabilidad intra-observadores" OR tradução OR reprodutibilidade OR "área sob a curva" OR "curva roc" OR "análise fatorial" OR psicometri* OR sensibilidade OR "variação observadora" OR "reprodutibilidade do resultado" OR "reprodutibilidade dos resultados")

AND ("depressão maior" OR depress* OR "transtorno de humor" OR "trastornos de humor" OR ansiedade OR "desordem ansiosa" OR "estresse pós-traumático" OR "estresse psicológico" OR "estresse agudo" OR fragil* OR "velho fragil" OR "velhos fragiles" OR "distúrbio cognitivo" OR "distúrbios cognitivos" OR "distúrbio neurocognitivo" OR "distúrbios neurocognitivos" OR "trastorno mental" OR "trastornos mentales" OR "doença psiquiátrica" OR "doenças psiquiátricas" OR "trastorno psiquiatrico" OR "trastornos psiquiatricos" OR neurocognitiv* OR "declínio cognitivo" OR "disfunção cognitiva" OR "disfunções cognitivas" OR "comprometimento cognitivo" OR DCL OR "comprometimento cognitivo leve" OR demência OR alzheimer* OR "dependência de álcool" OR "viciado em álcool" OR "viciados em álcool" OR vício OR alcoolismo OR "dependência de drogas" OR "viciado em drogas" OR "viciados em drogas" OR "abuso de drogas" OR "abuso de álcool" OR "problemas com álcool" OR "problemas com drogas" OR "abuso de substâncias" OR solidão OR "capital social" OR "suporte social" OR "saúde mental" OR "bem estar" OR "qualidade de vida" OR felicidade OR resiliência OR "adaptação psicológica" OR enfrentamento OR satisfação OR "atividades cotidianas" OR viciante OR "consumo de álcool" OR "Distúrbios relacionados aos opióides" OR "dependência de heroína" OR "distúrbios relacionados ao álcool" OR "abuso de medicamentos controlados" OR "distúrbios relacionados à cocaína" OR "abuso de drogas" OR "distúrbios relacionados a substâncias" OR "detecção de abuso de drogas" OR viciado*)

**SciELO search strategy**

((depresi* OR "trastorno* del animo" OR ansiedad OR "trastorno ansioso*" OR "estres postraumatico*" OR "estres psicologico" OR "estres agudo*" OR fragil* OR "trastorno* cognitivo*" OR "trastorno* neurocognitivo*" OR "trastorno* mental*" OR "enfermedad* psiquiatrica*" OR "trastorno psiquiatrico" OR neurocognitiv* OR "deterioro* cognitivo*" OR DCL OR "deterioro cognitivo leve" OR demencia OR alzheimer*) OR (soledad OR "capital social" OR "apoyo social" OR "salud mental" OR bienestar OR "calidad de vida" OR felicidad OR resiliencia OR "adaptación psicológica" OR afrontamiento OR satisfacción OR "actividades cotidianas")) AND (cribado OR tamizaje OR "cribado masivo" OR screen* OR "busqueda de casos" OR "evaluacion geriatrica" OR "evaluacion sintoma*" OR diagnos* OR identificación) AND ("tercera edad" OR anciano* OR "personas mayores" OR envejecimiento OR "adulto* mayor*" OR "edad sobre 60" OR vejez OR geria*) AND (latinoamerica OR "america latina" OR "am?rica del sur" OR "sudamerica" OR "centroamerica" OR "america central" OR caribe OR peru OR colombia OR ecuador OR chile OR me?ico OR argentina OR paraguay OR uruguay OR surinam OR guyana OR "guayana francesa" OR belice OR "el salvador" OR "costa rica" OR honduras OR nicaragua OR guatemala OR panama OR venezuela OR cuba OR "republica dominicana" OR haiti OR guadalupe OR martinica OR "san martin" OR "san bartolome" OR "san pedro y miquelon") AND ("estado mental" OR "test de demencia" OR validacion OR adaptacion OR precision OR sensibilidad OR especificidad OR "prueba* neuropsicologica*" OR "variabilidad intra-observador*" OR "variabilidad inter-observador*" OR "psicometrica*" OR traduccion OR "reproducibilidad" OR "área bajo la curva" OR "curva roc" OR "analisis factorial*" OR "tecnica* diagnostica*" OR "culturalmente competente" OR "instrumentacion psicometrica" OR "instrumento* psicometrico*")

**Alternative search strategy**

"tercera edad" OR anciano* OR "personas mayores" OR envejecimiento OR "adulto* mayor*" OR "edad sobre 60" OR vejez OR geria*

AND

latinoamerica OR "america latina" OR "america del sur" OR "sudamerica" OR "centroamerica" OR "america central" OR caribe OR peru OR colombia OR ecuador OR chile OR mejico OR mexico OR argentina OR paraguay OR uruguay OR surinam OR guyana OR "guayana francesa" OR belice OR "el salvador" OR "costa rica" OR honduras OR nicaragua OR guatemala OR panama OR venezuela OR cuba OR "republica dominicana" OR haiti OR guadalupe OR martinica OR "san martin" OR "san bartolome" OR "san pedro y miquelon"

AND

psicometri* OR escala OR cuestionario OR inventario OR índice OR test **OR cribado OR tamizaje OR "cribado masivo" OR screen* OR "busqueda de casos" OR "evaluacion geriatrica" OR "evaluacion sintoma*" OR diagnos* OR identificacion**

AND

validacion OR adaptacion OR precision OR sensibilidad OR especificidad OR confiabilidad OR "validez de criterio" OR "variabilidad intra-observador*" OR "variabilidad inter-observador*" OR traduccion OR "reproducibilidad" OR "area bajo la curva" OR "curva roc" OR "analisis factorial*"

AND

depresi* OR "trastorno* del animo" OR ansiedad OR "trastorno ansioso*" OR "estres postraumatico*" OR "estres psicologico" OR "estres agudo*" OR fragil* OR "trastorno* cognitivo*" OR "trastorno* neurocognitivo*" OR "trastorno* mental*" OR "enfermedad* psiquiatrica*" OR "trastorno psiquiatrico" OR neurocognitiv* OR "deterioro* cognitivo*" OR DCL OR "deterioro cognitivo leve" OR demencia OR alzheimer* OR soledad OR "capital social" OR "apoyo social" OR "salud mental" OR bienestar OR "calidad de vida" OR felicidad OR resiliencia OR "adaptación psicologica" OR afrontamiento OR satisfacción OR "actividades cotidianas"

**SciELO alternative Search strategy**

("tercera edad" OR anciano* OR "personas mayores" OR envejecimiento OR "adulto* mayor*" OR "edad sobre 60" OR vejez OR geria*) AND (latinoamerica OR "america latina" OR "am?rica del sur" OR "sudamerica" OR "centroamerica" OR "america central" OR caribe OR peru OR colombia OR ecuador OR chile OR me?ico OR argentina OR paraguay OR uruguay OR surinam OR guyana OR "guayana francesa" OR belice OR "el salvador" OR "costa rica" OR honduras OR nicaragua OR guatemala OR panama OR venezuela OR cuba OR "republica dominicana" OR haiti OR guadalupe OR martinica OR "san martin" OR "san bartolome" OR "san pedro y miquelon") AND (psicometri* OR escala OR cuestionario OR inventario OR indice OR test **OR cribado OR tamizaje OR "cribado masivo" OR screen* OR "busqueda de casos" OR "evaluacion geriatrica" OR "evaluacion sintoma*" OR diagnos* OR identificacion**) AND (validacion OR adaptacion OR precision OR sensibilidad OR especificidad OR confiabilidad OR "validez de criterio" OR "variabilidad intra-observador*" OR "variabilidad inter-observador*" OR traduccion OR "reproducibilidad" OR "area bajo la curva" OR "curva roc" OR "analisis factorial*") AND (depresi* OR "trastorno* del animo" OR ansiedad OR "trastorno ansioso*" OR "estres postraumatico*" OR "estres psicologico" OR "estres agudo*" OR fragil* OR "trastorno* cognitivo*" OR "trastorno* neurocognitivo*" OR "trastorno* mental*" OR "enfermedad* psiquiatrica*" OR "trastorno psiquiatrico" OR neurocognitiv* OR "deterioro* cognitivo*" OR DCL OR "deterioro cognitivo leve" OR demencia OR alzheimer* OR soledad OR "capital social" OR "apoyo

social" OR "salud mental" OR bienestar OR "calidad de vida" OR felicidad OR resiliencia OR "adaptación psicologica" OR afrontamiento OR satisfacción OR "actividades cotidianas")
