# Supplementary material for: Psychometric Evidence of Instruments for Assessing Mental Health in Older Adults from Latin America and the Caribbean: A Scoping Review
Source: Healthcare (Basel). 2026 Jan 21;14(2):265. doi: 10.3390/healthcare14020265 (PMC12841404; doi:10.3390/healthcare14020265)
Supplement: Supplementary file 1 [file healthcare-14-00265-s001.zip › 2_Supplemental Material S4_Outcome..pdf]

Supplemental Material S4 for:

Psychometric evidence of instruments for assessment of mental health of older adults of Latin America. A scoping review

Table S1. Frequency of instruments used to assess mental health in older adults from Latin America and the Caribbean.

Name Tool	n	%
Mini-Mental State Examination	22	6.45
Geriatric Depression Scale	21	6.16
Montreal Cognitive Assessment	18	5.27
Addenbrooke’s Cognitive Examination	14	4.11
World Health Organization Quality of Life	13	3.81
Clock Drawing Test	9	2.64
Short form health survey questionnaire	7	2.05
Cambridge Cognitive Examination	6	1.76
nformant Questionnaire on Cognitive Decline in the Elderly	6	1.76
Clinical Dementia Rating	4	1.17
Center for Epidemiologic Studies Depression Scale	4	1.17
Rowland Universal Dementia Assessment Scale	4	1.17
Brief Resilience Coping Scale	3	0.88
De Jong Gierveld Loneliness Scale	3	0.88
Dementia Rating Scale	3	0.88
European Depression Scale	3	0.88
Geriatric Anxiety Inventory	3	0.88
Leganés Cognitive Test	3	0.88
Photo-test	3	0.88
Quality of Life in Alzheimer’s Disease	3	0.88
Syndrom-Kurztest	3	0.88
Satisfaction With Life Scale	3	0.88
Questionnaire Alzheimer’s Disease 8	2	0.59
Alzheimer’s Disease Cooperative Study – Activities of Daily Living	2	0.59
Boston Naming Test	2	0.59
BRIEF spiritual/religious coping scale	2	0.59
Cognitive Reserve Questionnaire	2	0.59
Cornell Scale for Depression in Dementia	2	0.59
Community screening instrument for dementia	2	0.59
Delirium diagnostic tool-provisional	2	0.59
Baptista Depression Scale - Older Version	2	0.59
Everyday Cognition Scale	2	0.59

Escala de Soledad en la Tercera Edad	2	0.59
The Frontal Assessment Battery	2	0.59
Free and Cued Selective Reminding Test	2	0.59
Geriatric Mental State Examination	2	0.59
INECO Frontal Screening	2	0.59
Kessler Psychological Distress Scale (10-item)	2	0.59
Montgomery–Åsberg Depression Rating Scale	2	0.59
Michigan Alcohol Screening Test	2	0.59
Memory Alteration Test	2	0.59
Multifactorial Memory Questionnaire	2	0.59
Multidimensional Scale of Perceived Social Support	2	0.59
Mental Status Questionnaire	2	0.59
California Older Person Pleasant Events Schedule	2	0.59
Pain Assessment Checklist for Seniors with Limited Ability to Communicate	2	0.59
Patient Health Questionnaire	2	0.59
Test Your Memory	2	0.59
WHO Disability Assessment Schedule	2	0.59
COV10-Qol	1	0.29
Conflict Tactics Scale	1	0.29
10-point Cognitive Screener	1	0.29
Three Words-Three Shapes Test	1	0.29
Five-word Test	1	0.29
Anxiety about Aging Scale	1	0.29
Spiritual Wellbeing Scale	1	0.29
Alzheimer's Disease Assessment Scale	1	0.29
Multidimensional Assessment of Older People in Primary Care	1	0.29
ANU Alzheimer Disease Risk Index	1	0.29
Anosognosia Questionnaire in Dementia	1	0.29
confronting loneliness subscale	1	0.29
Attitudes towards Sexuality in the Elderly	1	0.29
Bayer-Activities of Daily Living scale	1	0.29
Behavioral Assessment of the Dysexecutive Syndrome	1	0.29
Brief Assessment Scale for Depression	1	0.29
Blessed Dementia Scale	1	0.29
Bender Gestalt Test	1	0.29

Brian Health Assessment	1	0.29
Brief Index of Religiousness and Spirituality	1	0.29
Barrow Neurological Institute	1	0.29
Blessed Dementia Scale.	1	0.29
Brief-COPE	1	0.29
Cambridg Examination for Mental Disorders of the Elderly – Revised	1	0.29
Camberwell Assessment of Need for the Elderly	1	0.29
Computer-Administered Neuropsychological Screen for Mild Cognitive Impairment	1	0.29
Coronavirus Anxiety Scale	1	0.29
Caregiver Abuse Screen	1	0.29
CASP-19	1	0.29
Cross Cultural Cognitive Examination	1	0.29
Cognitive Complaints Questionnaire	1	0.29
Consortium to Establish a Registry for Alzheimer´s Disease	1	0.29
Cognitive Function Instrument	1	0.29
Category Fluency Test	1	0.29
5-item Coping with Humor Scale	1	0.29
Cognitive Telephone Screening Instrument	1	0.29
Coin Test	1	0.29
The Canadian occupational performance measure	1	0.29
Cognitive Change Questionnaire	1	0.29
Combined Screening Interpretation Score	1	0.29
Category Verbal Fluency	1	0.29
Disability Assessment for Dementia	1	0.29
Quality-of-Life Assessment in Dementia	1	0.29
Dysexecutive Questionnaire	1	0.29
General Activities of Daily Living Scale	1	0.29
Delirium predictive score	1	0.29
Scale of Self-Efficacy for Aging	1	0.29
Autobiographic Episodic Memory Interview	1	0.29
Aged adult adaptation scale to their residence	1	0.29
Despair scale for older adults	1	0.29
Semantic Memory Assessment Battery for Older Adults	1	0.29
ENEDAM	1	0.29
Euro-Quol 5	1	0.29

Elderly Quality of Life Index	1	0.29
Emotional Regulation Questionnaire	1	0.29
Eurotest	1	0.29
INECO Frontal Screening	1	0.29
Family Abuse Screening Questionnaire	1	0.29
Fear of COVID-19 Scale	1	0.29
FTD Rating Scale	1	0.29
Geriatric PsychoSocial Assessment of	1	0.29
Healthy Ageing Index	1	0.29
Health Perceptions Questionnaire	1	0.29
Iowa Gambling Task	1	0.29
Leisure Attitude Measurement	1	0.29
Lasher and Faulkender Anxiety	1	0.29
Minnesota Living with Heart Failure	1	0.29
LIFE-H	1	0.29
Life Orientation Test	1	0.29
Life Satisfaction Index short form	1	0.29
Memory Binding Test	1	0.29
Memory Complaint Scale	1	0.29
Modified Fatigue Impact Scale	1	0.29
Massachusetts General Hospital-Sexual Functioning Questionnaire	1	0.29
MHAS Questionnaire	1	0.29
Multidimensional Individual and Interpersonal Resilience Measure	1	0.29
Mini-Cog	1	0.29
Mini-SEA	1	0.29
Meaning in Life Questionnaire	1	0.29
Minilinguistic State Examination	1	0.29
Mindful Awareness Attention Scale	1	0.29
Medical Outcomes Studysocial Support Survey	1	0.29
Scale of Happiness of the Memorial University of New Foundland	1	0.29
Proposed care need classification	1	0.29
NeuroBel	1	0.29
Neuropsychological evaluation test – Neuropsi	1	0.29
Nottingham Health Profile	1	0.29
Older Adult Lifestyle Scale	1	0.29

Pain Locus of control scale	1	0.29
Premorbid Cognitive Abilities Scale	1	0.29
Parkinson's Disease Cognitive Rating Scale	1	0.29
Parkinson’s Disease Quality of Life Questionnaire Ecuador version	1	0.29
Pentagon Drawing Test	1	0.29
Pesotest	1	0.29
The pFCSRT-IR (picture Free and Cued Selective Reminding with Immediate Recall)	1	0.29
10-item Purpose in Life scale	1	0.29
Pain Intensity Measure for Persons with Dementia	1	0.29
Modified Parkinson Psychosis Rating Scale	1	0.29
Prospective Memory (ProM) tests	1	0.29
Prefrontal Symptoms Inventory	1	0.29
Perceived Stress Scale	1	0.29
Perceived social Support Questionnaire	1	0.29
Questionnaire of Communitysocial Support	1	0.29
Quality of Life Scale	1	0.29
r-SMS	1	0.29
Rey-Auditory Verbal Learning Test	1	0.29
Rivermead Behavioural Memory Test	1	0.29
Relevant Outcome Scale for Alzheimer ́s disease	1	0.29
Relationship Scales Questionnaire	1	0.29
Successful Aging Inventory	1	0.29
Short Blessed test	1	0.29
Revised version of the Scale	1	0.29
Scales for Outcomes in Parkinson's Disease-PsychoSocial Questionnaire	1	0.29
semantic fluency test	1	0.29
Spirituality Index	1	0.29
Scale of Losses Experienced in Old Age	1	0.29
Dr. Reed’s Spiritual Perspective Scale	1	0.29
Memory and behaviour symptoms checklist	1	0.29
Self-reporting questionnaire-SRQ 20	1	0.29
STADP	1	0.29
Quality of life and swallowing questionnaire for individuals with Parkinson's disease	1	0.29
Targeted Geriatric Assessment	1	0.29

Tuokko's Clock Test	1	0.29
The neuropsychological battery	1	0.29
UCLA	1	0.29
Visual Analogue Scale of Happiness and the Cornell Scale for Depression in Dementia	1	0.29
Vulnerability to Abuse Screening Scale	1	0.29
Vitor Quality of Life Scale for the Elderly	1	0.29
The Wagnild and Young Resilience Scale	1	0.29
Will-to-Live Scale	1	0.29

Values represent the number (n) and percentage (%) of times each instrument was reported across all included studies

Table S2. Frequency of psychometric instrument purposes in older adults from Latin America and the Caribbean

Purpose	n	%
Cognition	180	52.79
Depression	38	11.14
Quality of life	34	9.97
Spirituality	7	2.05
Loneliness	6	1.76
Anxiety	4	1.17
Life satisfaction	4	1.17
Mistreatment	4	1.17
Resilience	4	1.17
Social support	4	1.17
Daily activities cognitive impairment	3	0.88
Delirium	3	0.88
Happiness	3	0.88
Health	3	0.88
Pain	3	0.88
Pleasant activities	3	0.88
Alcohol abuse	2	0.59
Mental distress	2	0.59
Needs	2	0.59
Quality of Life	2	0.59
Sexuality	2	0.59
Adaptation to nursing homes	1	0.29
ageing-related anxiety	1	0.29
cope (Humor)	1	0.29
Coping	1	0.29
Coping and resilience	1	0.29
Coronaphobia	1	0.29
Decision making	1	0.29
Emotional regulation	1	0.29
Family abuse	1	0.29
Fatigue	1	0.29

Fear	1	0.29
Geriatric assesment (health)	1	0.29
Health (Disability)	1	0.29
Health (successful aging)	1	0.29
Health status	1	0.29
Hopeless	1	0.29
Lifestyle	1	0.29
Losses Experienced	1	0.29
Meaning in life	1	0.29
Minfulness	1	0.29
Optimism	1	0.29
Psychosis	1	0.29
Purpose in life	1	0.29
Quality of social participation	1	0.29
Relationships	1	0.29
Self-efficay	1	0.29
Stress	1	0.29
Will to live	1	0.29

Values represent the number (n) and percentage (%) of instrument mentions categorized by primary psychometric purpose across all included studies

Table S3. Frequency of types of analyses performed in studies from Latin America and the Caribbean.

Analysis	n	%
Reliability	201	64.4
Construct and/or Criterion Validity	165	52.9
Diagnostic accuracy	149	47.8
Factor Analysis	110	35.3
Adaptation	46	14.7
Translation	40	12.8
Divergent validity	30	9.6
Normative data	15	4.8
Rasch model	13	4.2
Development	11	3.5

Values represent the proportion of analyses identified across all included studies (n = total number of analyses).
