# Supplementary material for: Psychometric Evidence of Instruments for Assessing Mental Health in Older Adults from Latin America and the Caribbean: A Scoping Review
Source: Healthcare (Basel). 2026 Jan 21;14(2):265. doi: 10.3390/healthcare14020265 (PMC12841404; doi:10.3390/healthcare14020265)
Supplement: Supplementary file 1 [file healthcare-14-00265-s001.zip › Supplementary Material S2_Glossary of the search..pdf]

| Area | Term in English | Term in Spanish |
|---|---|---|
| **Tools and their use** | | |
| | psychometr* | psicometri* |
| | scale | escala |
| | questionnaire | cuestionario |
| | inventory | inventario |
| | test | test |
| | screen* | cribado |
| | screening | tamizaje |
| | "mass screening" | "cribado masivo" |
| | "case finding" | "busqueda de casos" |
| | "geriatric evaluation" | "evaluacion geriatrica" |
| | "geriatric assessment" | "evaluacion geriatrica" |
| | Symptom assessment | "evaluacion sintoma*" |
| | "symptom evaluation" | "evaluacion sintoma*" |
| | diagnos* | diagnost* |
| | identification | identificacion |
| | measure | medida* |
| | tool | herramienta* |
| **Target population** | | |
| | aged | envejecido |
| | senior OR senior* | |
| | "third age" | "tercera edad" |
| | elder* | ancian* |
| | "old* people" | "persona* mayor*" |
| | "old* person" | "persona* mayor*" |
| english | ageing OR aging | envejecimiento |
| | "older adult*" | "adulto* mayor*" |
| | "aged 60" | "edad sobre 60" |
| | "old age" | vejez |
| | geriatr* | geriatr* |
| | aged 80 | "edad sobre 80" |
| **Geolocation** | | |
| | "latin america" | latinoamerica |
| | "latin america" | "america latina" |
| | "south america" | "america del sur" |
| | "south america" | sudamerica |
| | "central america" | centroamerica |
| | "central america" | "america central" |
| | caribbean | caribe |
| | peru | peru |
| | colombia | colombia |
| | ecuador | ecuador |
| | chili | chile |
| | chile | chile |
| | brasil | brasil |
| | brazil | brazil |
| | mexico | mejico |
| | mexico | mexico |

| | | |
|---|---|---|
| | argentina | argentina |
| | paraguay | paraguay |
| | uruguay | uruguay |
| | surinam | surinam |
| | guyana | guyana |
| | "french guiana" | "guayana francesa" |
| | belize | belice |
| | "el salvador" | "el salvador" |
| | "costa rica" | "costa rica" |
| | honduras | honduras |
| | nicaragua | nicaragua |
| | guatemala | guatemala |
| | panama | panama |
| | venezuela | venezuela |
| | cuba | cuba |
| | "dominican republic" | "republica dominicana" |
| | haiti | haiti |
| | guadeloupe | guadalupe |
| | martinique | martinica |
| | "saint martin" | "san martin" |
| | "saint barthelemy" | "san bartolome" |
| | "saint pierre" | "san pedro y miquelon" |
| **study quality** | | |
| | validation | validacion |
| | adaptation | adaptacion |
| | accuracy | precision |
| | specificity | especificidad |
| | reliability | confiabilidad |
| | "criterion validity" | "validez de criterio" |
| | "intra-observer reliability" | observador*" |
| | "inter-observer reliability" | observador*" |
| UK english not USA | inter-rater reliability | observador" |
| UK english not USA | intra-rater reliability | observador" |
| | translation | traduccion |
| | reproducibility | reproducibilidad |
| | "area under curve" | "area bajo la curva" |
| | "roc curve" | "curva roc" |
| | "factor analysis" | "analisis factorial*" |
| meanings in english | psychometr* | psicometri* |
| | sensitivity | Sensibilidad |
| | observer variation | "variacion del observador" |
| | reproducibility of results' | resultado*" |
| **outcomes** | | |
| | major depression | mayor |
| | depress* | depresi* |
| | "mood disorder" | "trastorno* del animo" |
| | anxiety | ansiedad |

| | |
|---|---|
| "anxiety disorder" | "trastorno* ansioso*" |
| "post-traumatic stress" | "estres postraumatico" |
| "psychological stress" | "estres psicologico" |
| "acute stress" | "estres agudo" |
| Frail* | fragil* |
| frail elderly' | "ancian* fragil*" |
| "cognitive disorder*" | "trastorno* cognitivo*" |
| "neurocognitive disorder*" | neurocognitivo*" |
| "mental disorder*" | "trastorno* mental*" |
| "psychiatric illness" | psiquiatrica*" |
| "psychiatric disorder*" | "trastorno* psiquiatrico*" |
| neurocognitiv* | neurocognitiv* |
| cognitive decline | "declive cognitivo" |
| cognitive dysfunction | "disfuncion* cognitiva*" |
| "cognitive impairment" | "deterioro cognitivo" |
| MCI | DCL |
| "mild cognitive impairment" | "deterioro cognitivo leve" |
| dementia | demencia |
| alzheimer* | alzheimer* |
| "addict* to alcohol" | "adiccion al alcohol" |
| "addict* to alcohol" | "adict* al alcohol" |
| addiction | adiccion |
| alcoholism | alcoholismo |
| "addict* to drugs" | "adiccion a las drogas" |
| "addict* to drugs" | "adict* a las drogas" |
| "drug addiction" | drogadiccion |
| "drug abuse" | "abuso de drogas" |
| "alcohol abuse" | "abuso de alcohol" |
| "alcohol problems" | "problemas con el alcohol" |
| "drug problems" | "problemas con las drogas" |
| "substance abuse" | "abuso de sustacias" |
| loneliness | soledad |
| "social capital" | "capital social" |
| "social support" | "apoyo social" |
| "mental health" | "salud mental" |
| wellbeing | bienestar |
| "quality of life" | "calidad de vida" |
| happiness | felicidad |
| resilience | resiliencia |
| "psychological adaptation" | "adaptacion psicologica" |
| coping | afrontamiento |
| satisfaction | satisfaccion |
| "activities of daily living" | "actividades cotidianas" |
| "daily activities" | "actividades cotidianas" |
| alcohol misuse mp | "abuso de alcohol" |
| substance misuse | "abuso de sustancias" |
| alcohol dependence.mp. | /"dependencia al alcohol" |

| | |
|---|---|
| *Behavior, Addictive | adictiv* |
| *Substance Abuse, Oral | abuse"** |
| misuse.mp. | |
| *Alcohol Drinking | "consumo de alcohol" |
| *Alcoholism | alcoholismo |
| *Opioid-Related Disorders | los opiaceos" |
| *Heroin Dependence | "dependencia a la heroina" |
| Intravenous | abuse"** |
| *Alcohol-Related Disorders | alcohol" |
| *Prescription Drug Misuse | recetados" |
| *Cocaine-Related Disorders | la cocaina" |
| *Drug Misuse | "abuso de drogas" |
| Disorders | las sustancias" |
| *Substance Abuse Detection | drogas" |
| addict*.mp | adict* |
| substance.mp | |

**Term in Portuguese**

psychometri *
escala
questionário
inventário
teste
triagem
triagem
"rastreio em massa"
"pesquisa de caso"
"avaliação geriátrica"
"avaliação geriátrica"
"avaliação dos sintomas *"
"avaliação dos sintomas *"
diagnóstico *
identificação
medida *
ferramenta *

envelhecido

"terceira idade"
antigo *
"pessoa maior*"
"pessoa maior*"
envelhecimento
"adulto * mais velho *"
"idade acima de 60 anos"
velhice
geriatr *
"Idade acima de 80"

América Latina
"América Latina"
"América do Sul"
américa do sul
América Central
"América Central"
caribe
Peru
Colômbia
Equador
pimentão
pimentão
brasil
brasil
mexicano
méxico

argentina
paraguai
Uruguai
suriname
guiana
"Guiana Francesa"
belize
"o salvador"
"Costa Rica"
Honduras
Nicarágua
Guatemala
Panamá
Venezuela
Cuba
"República Dominicana"
haiti
Guadalupe
Martinica
"são Martim"
"San Bartolomeu"
"San Pedro e Miquelon"

validação
adaptação
precisão
especificidade
fiabilidade
"validade de critério"
observador *"
interobservador *"
interobservador"
observador"
tradução
reprodutibilidade
"área sob a curva"
"curva roc"
"análise fatorial *"
psychometri *
Sensibilidade
"variação observadora"
resultado *"

maior
depressão *
"transtorno de humor *"
ansiedade

"desordem * ansiosa *"
"estresse pós-traumático"
"estresse psicológico"
"estresse agudo"
frágil *
"velho * frágil *"
"distúrbio cognitivo *"
"distúrbio neurocognitivo *"
"transtorno mental*"
"doença psiquiátrica *"
"transtorno psiquiátrico *"
neurocognitiv *
"declínio cognitivo"
"disfunção cognitiva *"
cognitivo"
DCL
cognitivo leve"
demência
Alzheimer *
"dependência de álcool"
"viciado * em álcool"
vício
alcoolismo
"dependência de drogas"
"viciado * em drogas"
dependência de drogas
"abuso de drogas"
"abuso de álcool"
"problemas com álcool"
"problemas com drogas"
"abuso de substâncias"
solidão
"capital social"
"suporte social"
"saúde mental"
bem estar
"qualidade de vida"
felicidade
resiliência
"adaptação psicológica"
enfrentamento
satisfação
"atividades cotidianas"
"atividades cotidianas"
"abuso de álcool"
"abuso de substâncias"
"dependência de álcool"

viciante *
substâncias" **

"consumo de álcool"
alcoolismo
opióides"
"dependência de heroína"
substâncias" **
álcool"
controlados"
cocaína"
"abuso de drogas"
substâncias"
drogas"
viciado *
