# Supplementary material for: Psychometric Evidence of Instruments for Assessing Mental Health in Older Adults from Latin America and the Caribbean: A Scoping Review
Source: Healthcare (Basel). 2026 Jan 21;14(2):265. doi: 10.3390/healthcare14020265 (PMC12841404; doi:10.3390/healthcare14020265)
Supplement: Supplementary file 1 [file healthcare-14-00265-s001.zip › Supplementary Material S3_Charting data extraction.pdf]

Autor	Tittle
Belaus, A. and Fernandez, L. A. and Farias-Sarquis, Y. and Bueno, A. M.	Is the Mattis Dementia Rating Scale appropriate to detect Mild Cognitive Impairment?
Blanco, R. and Roman, F. and Iturry, M. and Leis, A. and Russo, M. J. and Bartoloni, L. and Barcelo, E. and Allegri, R. F.	AD8-Argentina questionnaire to detect dementia in primary health care
Fernandez, A. L. and Fulbright, R. L.	Construct and Concurrent Validity of the Spanish Adaptation of the Boston Naming Test
Serrani, D.	Spanish Validation of the TYM Test for Dementia Screening in an Argentine Population
Salvia, M. G. and Dawidowski, A. and Schapira, M. and Figar, S. and Soderlund, M. E. and Seinhart, D. and Camera, L. and Teri, L.	Spanish Revised Memory and Behavior Problems Checklist Scale (SpRMBPC): trans-cultural adaptation and validation of the RMBPC questionnaire
Roman, Fabian and Iturry, Mónica and Rojas, Galeno and Barceló, Ernesto and Buschke, Herman and Allegri, Ricardo F.	Validation of the Argentine version of the Memory Binding Test (MBT) for Early Detection of Mild Cognitive Impairment
Russo, M. J. and Cohen, G. and Mendez, P. C. and Campos, J. and Martin, M. E. and Clarens, M. F. and Tapajoz, F. and Harris, P. and Sevlever, G. and Allegri, R. F.	Utility of the Spanish version of the Everyday Cognition scale in the diagnosis of mild cognitive impairment and mild dementia in an older cohort from the Argentina-ADNI
Russo, Maria Julieta and Iturry, Monica and Sraka, Maria Alejandra and Bartoloni, Leonardo and Carnero Pardo, Cristobal and Allegri, Ricardo Francisco	Diagnostic accuracy of the phototest for cognitive impairment and dementia in Argentina
Seinhart, D. B. and Castro, D. and Borgioli, D. and Guelar, V. and Sanchez, V. N. and Vicario, A. and Leguizamon, P. P. and Pawluk, M. S. and Sueldo, R. D. and Del Sueldo, M. and De Azkue, M. I. and Taragano, F. E.	Validation of the brief version of the community screening instrument for dementia (CSID) in a rural population in Argentina

Sousa, L. and Vivas, L.	Normative values of the Addenbrooke's Cognitive Examination (ACE) for low soICo-educational level population
Gonzalez Aguilar, María Josefina, Alba Ferrara, Lucía.	CSIS: Proposal for a new Combined Screening Interpretation Score for patients with Mild Cognitive Impairment
Vivas, L., Martínez-Cuitiño, M., Manoiloff, L., Romanelli, S., Garrard, P.	Translation and linguistic and cultural adaptation of the Minilinguistic State Examination into Argentine Spanish
Cervigni, M., Martino, P., Alfonso, G., Politis, D.	Montreal Cognitive Assessment (MoCA): Normative data for Rosario metropolitan area population, Argentina
Llarena Nuñez, S., Bruno, D.	Validation of the Cognitive Complaints Questionnaire
Martino, P., Caycho-Rodríguez, T., ValenICa, P.D., Politis, D., Gallegos, M., Bortoli, M.Á.D., Cervigni, M	Cognitive Reserve Questionnaire: psychometric analysis from the item response theory
Serrano, CeIClia M and Sorbara, Marcos and Minond, Alexander and Finlay, John B and Arizaga, Raul L and Iturry, Monica and Martinez, PatriICa and Heinemann, Gabriela and Gagliardi, Celina and Serra, Andrea and Magliano,	Validation of the Argentine version of the Montreal Cognitive Assessment Test (MOCA): A screening tool for Mild Cognitive Impairment and Mild Dementia in Elderly.
Tartaglini, María FlorenICa, Hermida, Paula Daniela, Ofman, Silvia Deborah, Feldberg, Carolina, Freiberg-Hoffmann, Agustín.	Yesavage's Geriatric Depression Questionnaire (GDS-VE): Analysis of its internal structure in older adults living in Buenos Aires, Argentina
Richard's, M.M., Krzemien, D., Comesaña, A., Zamora, E.V., Cupani, M.	Confirmatory factor analysis of the Spanish version of the brief-COPE in Argentine elderly people
Martino, P. and Cervigni, M. and Caycho-RodrÃ-guez, T. and ValenICa, P. and Politis, D.	Cognitive reserve questionnaire: The psychometric properties in an Argentinian population

Bruno, D, Slachevsky, A, Fiorentino, N, Rueda, D S, Bruno, G, Tagle, A R, Olavarria, L, Flores, P, Lillo, P, Roca, M, Torralva, T.	Argentinian/Chilean validation of the Spanish-language version of Addenbrooke's Cognitive Examination III for diagnosing dementia
Aliberti, M. J. R., Apolinario, D., Suemoto, C. K., Melo, J. A., Fortes, S. Q. and Saraiva, M. D., Trindade, C. B., Covinsky, K. E., Jacob, W.	Targeted Geriatric Assessment for Fast-Paced Healthcare Settings: Development, Validity, and Reliability
Almeida, Carla Bezerra Lopes and Félix, Ricardo Humberto and Cendoroglo, Maysa Seabra and Santos, Fania Cristina	Pain-induced depression in the elderly: Validation of psychometric properties of the Brazilian version of the "Geriatric Emotional Assessment of Pain" - GEAP-b
Bahia, Valeria Santoro and Carthery-Goulart, Maria Teresa and Novelli, MarICa M. and Kato-Narita, Eliane M. and Areza-Fegyveres, Renata and Caramelli, Paulo and Nitrini, Ricardo	Functional disability in Alzheimer disease: A validation study of the Brazilian version of the Disability Assessment for Dementia (DAD-Br)
Batistoni, S. S. T. and Neri, A. L. and Cupertino, Apfb	Validity of the Center for Epidemiological Studies Depression Scale among Brazilian elderly
Batistoni, Samila Sathler Tavares and Ordonez, Tiago NasICmento and da Silva, ThaÃs Bento Lima and do NasICmento, PrisICla Pascarelli Pedrico and Cachioni, Meire	Emotional Regulation Questionnaire (ERQ): Psychometric Indicators and Affective Relations in an Elderly Sample
BertolucIC, P. H. and Okamoto, I. H. and Brucki, S. M. and Siviero, M. O. and Toniolo Neto, J. and Ramos, L. R.	Applicability of the CERAD neuropsychological battery to Brazilian elderly
Bezerra T, Karol and Martins Gazoni, Fernanda and Liausu Cherpak, Guilherme and Clasen Lorenzet, Isabel and Dos Santos, LuICana Alves and Maria Nardes, Edlene and Dos Santos, FÃ¢nia Cristina	Pain assessment in elderly with dementia: Brazilian validation of the PACSLAC scale
Blay, S. L. and De Jesus Mari, J. and Ramos, L. R. and Ferraz, M. P. T.	Validity of a Brazilian version of the mental status questionnaire as a screening test for dementia among elderly Urban subjects. A pilot study
Borges, Marcus Kiiti and Jacinto, Alessandro Ferrari and ICtero, Vanessa de Albuquerque	Cross-cultural adaptation of the "Australian National University Alzheimer's Disease Risk Index" for the Brazilian population

Brito-Marques, P. R. and Cabral-Filho, J. E.	The role of education in mini-mental state examination: a study in Northeast Brazil
Brito-Marques, P. R. and Cabral-Filho, J. E.	Influence of age and schooling on the performance in a modified Mini-Mental State Examination version: a study in Brazil northeast
Caldas, V. V. and Zunzunegui, M. V. and Freire Ado, N. and Guerra, R. O.	Translation, cultural adaptation and psychometric evaluation of the Leganes cognitive test in a low educated elderly Brazilian population
Camozzato, A. L. and Kochhann, R. and Godinho, C. and Costa, A. and Chaves, M. L.	Validation of a telephone screening test for Alzheimer's disease
Canali, F. and Brucki, S. M. and BertolucIC, P. H. and Bueno, O. F.	Reliability study of the Behavioral Assessment of the Dysexecutive Syndrome adapted for a Brazilian sample of older-adult controls and probable early Alzheimer's disease patients
Carvalho, V. A. and Barbosa, M. T. and Caramelli, P.	Brazilian Version of the Addenbrooke Cognitive Examination-revised in the Diagnosis of Mild Alzheimer Disease
Carvalho, Viviane Amaral and Caramelli, Paulo	Brazilian adaptation of the Addenbrooke Cognitive Examination-Revised (ACE-R)
Da Silva, J. V. and Baptista, M. N.	Vitor Quality of Life Scale for the Elderly: evidence of validity and reliability
Damasio, B. F. and Koller, S. H.	Meaning in Life Questionnaire: Adaptation process and psychometric properties of the Brazilian version
Damin, Antonio Eduardo and Nitrini, Ricardo and Brucki, Sonia Maria Dozzi	Cognitive Change Questionnaire as a method for cognitive impairment screening

Dantas, Raquel Batista and Oliveira, Graziella Lage and Silveira, Andrea Maria	Psychometric properties of the Vulnerability to Abuse Screening Scale for screening abuse of older adults
de Araujo, N. B. and Nielsen, T. R. and Engedal, K. and Barca, M. L. and Coutinho, E. S. and Laks, J.	Diagnosing dementia in lower educated older persons: validation of a Brazilian Portuguese version of the Rowland Universal Dementia Assessment Scale (RUDAS)
de Assis, E. N. and Loureiro, F. S. and Menta, C. and Nogueira, E. L. and da Silva, I. G. and von Gunten, A. and Neto, A. C.	Translation and Brazilian adaptation of the Relationship Scales Questionnaire (RSQ)
Fagundes Chaves, M. L. and Camozzato, A. L. and Godinho, C. and Kochhann, R. and Schuh, A. and De Almeida, V. L. and Kaye, J.	Validity of the clinical dementia rating scale for the detection and staging of dementia in Brazilian patients
Ferrari Jacinto, Alessandro and De Oliveira Aguiar, Ana Cristina ProcÃ³pio and De Melo Franco, Fabio Gazelato and Ikeda Ribeiro, Miriam and de Albuquerque ICtero, Vanessa	Dementia Rating Scale psychometric study and its applicability in long term care institutions in Brazil
Ferreira, H. G. and Barham, E. J. and Fontaine, Amgv	A Measure to Assess Elderly Brazilians' Involvement in Pleasant Activities: Initial Evidence of Internal and External Validity
Ferrer, M. L. P.	WHODAS 2.0-BO: normative data for the assessment of disability in older adults
Flaks, Mariana K. and Forlenza, Orestes V. and Pereira, Fernanda S. and Viola, LuICane F. and Yassuda, Monica S.	Short Cognitive Performance Test: Diagnostic accuracy and education bias in older Brazilian adults
Flaks, Mariana K. and Yassuda, Monica S. and Regina, Ana Carolina B. and ICd, Carlo G. and Camargo, Candida H. and Cattaz, Wagner F. and Forlenza, Orestes V.	The Short Cognitive Performance Test (SKT): A preliminary study of its psychometric properties in Brazil
Fleck, M. P. and Chachamovich, E. and Trentini, C.	Development and validation of the Portuguese version of the WHOQOL-OLD module

Fortes, S. Q. and Apolinario, D. and Melo, J. A. and Suzuki, I. and Sitta, M. D. and Leme, L. E. G.	Predicting delirium after hip fracture with a 2-min cognitive screen: prospective cohort study
Fuzikawa, C. and Lima-Costa, M. F. and Uchoa, E. and Barreto, S. M. and Shulman, K.	A population based study on the intra and inter-rater reliability of the clock drawing test in Brazil: the Bambui Health and Ageing Study
Goncalves Ferreira, Heloísa and Joan Barham, Elizabeth	Factor Structure of the Brazilian Version of the California Older Person's Pleasant Events Schedule
Hirata, E. S. and Almeida, O. P. and Funari, R. R. and Klein, E. L.	Validity of the Michigan Alcoholism Screening Test (MAST) for the detection of alcohol-related problems among male geriatric outpatients
Jacinto, A. F. and Aguiar, A. C. and Franco, F. G. and Ribeiro, M. I. and ICtero, V. A.	Dementia Rating Scale psychometric study and its applicability in long term care institutions in Brazil
Jacinto, A. F. and Brucki, S. M. and Porto, C. S. and Martins, M. D. and ICtero, V. D. and Nitrini, R.	Suggested instruments for General Practitioners in countries with low schooling to screen for cognitive impairment in the elderly
Jacinto, Alessandro Ferrari and Brucki, Sonia Maria Dozzi and Porto, Claudia Sellitto and Martins, Milton de Arruda and Nitrini, Ricardo	Screening of cognitive impairment by general internists using two simple instruments
Kano, MarICa Yumi and Santos, Manoel Antonio dos and Pillon, Sandra Cristina	Use of alcohol in the elderly: transcultural validation of the Michigan Alcoholism Screening Test - Geriatric Version (MAST-G)
Leite, K. S. and Miotto, E. C. and Nitrini, R. and Yassuda, M. S.	Boston Naming Test (BNT) original, Brazilian adapted version and short forms: normative data for illiterate and low-educated older adults
Lessnau Coutinho, FranICs and Cavalheiro Hamdan, Amer and Nunes Baptista, Makilim	Baptist scale for elderly people's depression-EBADEP-ID: validity evidence

Lima, D. A. and Lourenco, R. A.	Cross-cultural adaptation of section A of the Cambridge Examination for Mental Disorders of the Elderly-Revised Version (CAMDEX-R) for dementia diagnosis
Lima, F. M. and Hyde, M. and Chungkham, H. S. and Correia, C. and Campos, A. S. and Campos, M. and Novaes, M. and Laks, J. and Petribu, K.	Quality of Life amongst Older Brazilians: A Cross-Cultural Validation of the CASP-19 into Brazilian-Portuguese
Lorenzet, I. C. and Dos Santos, F. C. and De Souza, P. M. R. and Gambarro, R. C. and Coelho, S. and Cendoroglo, M. S.	Assessment of pain in elderly patients with dementia: Translation and transcultural adaptation of the instrument PACSLAC into Portuguese.
Lourenço, R. A., Ribeiro-Filho, S. T., Moreira, I.deF., Paradela, E. M., & Miranda, A. S.	The Clock Drawing Test: performance among elderly with low educational level
Lourenco, R. A. and Sanchez, M. A. D.	Accuracy of the Brazilian Version of the Informant Questionnaire on Cognitive Decline in the Elderly at Screening for Dementia in Community-Dwelling Elderly participants: Findings From FIBRA-RJ Study
Lourenco, R. A. and Veras, R. P.	Mini-Mental State Examination: psychometric characteristics in elderly outpatients
Luft, C. D. and Sanches, S. D. and Mazo, G. Z. and Andrade, A.	Brazilian version of the Perceived Stress Scale: translation and validation for the elderly
Almeida, O. P.	The mini-mental state examination and the diagnosis of dementia in Brazil
Almeida, O. P. and Almeida, S. A.	Reliability of the Brazilian version of the geriatric depression scale (GDS) short form
Almeida, O. P. and Almeida, S. A.	Short versions of the geriatric depression scale: A study of their validity for the diagnosis of a major depressive episode according to ICD-10 and DSM-IV

Alvarenga, M. R. M. and Oliveira, M. A. D. and Faccenda, O.	Depressive symptoms in the elderly: analysis of the items of the Geriatric Depression Scale
Apolinario, D., Brucki, S. M., Ferretti, R. E., Farfel, J. M., Magaldi, R. M., Busse, A. L., & Jacob-Filho, W.	Estimating premorbid cognitive abilities in low-educated populations
Apolinario, D., Dos Santos, M. F., Sassaki, E., Pegoraro, F., Pedrini, A. V. A., Cestari, B., Amaral, A. H., Mitt, M., Müller, M. B., Suemoto, C. K., & Aprahamian, I.	Normative data for the Montreal Cognitive Assessment (MoCA) and the Memory Index Score (MoCA-MIS) in Brazil: Adjusting the nonlinear effects of education with fractional polynomials
Aprahamian, I. and Martinelli, J. E. and Cecato, J. and Izbicki, R. and Yassuda, M. S.	Can the CAMCOG be a good cognitive test for patients with Alzheimer's disease with low levels of education?
Aprahamian, Ivan and Martinelli, Jose Eduardo and Neri, Anita Liberalesso and Yassuda, Monica Sanches	The accuracy of the Clock Drawing Test compared to that of standard screening tests for Alzheimer's disease: Results from a study of Brazilian elderly with heterogeneous education backgrounds
Atalaia-Silva, K. C. and Lourenco, R. A.	Translation, adaptation and construct validation of the Clock Test among elderly in Brazil
Casamali, F. F. C. and Schuch, F. B. and Scortegagna, S. A. and Legnani, E. and De Marchi, A. C. B.	Accordance and reproduICbility of the electronic version of the WHOQOL-BREF and WHOQOL-OLD questionnaires
Cassimiro, L. and Fuentes, D. and Nitrini, R. and Yassuda, M. S.	Decision-making in Cognitively Unimpaired Illiterate and Low-educated Older Women: Results on the Iowa Gambling Task
Castelo, M. S. and Coelho, J. M. and Carvalho, A. F. and Lima, J. W. O. and Noleto, J. C. S. and Ribeiro, K. G. and Siqueira-Neto, J. I.	Validity of the Brazilian version of the Geriatric Depression Scale (GDS) among primary care patients
Castro, P. C. and Driusso, P. and Oishi, J.	Convergent validity between SF-36 and WHOQOL-BREF in older adults

Castro-Costa, E. and Dewey, M. E. and Uchoa, E. and Firmo, J. O. and Lima-Costa, M. F. and Stewart, R.	Construct Validity of the mini mental state examination across time in a sample with low-education levels: 10-year follow-up of the Bambui Cohort Study of Ageing
Castro-Costa, E. and Fuzikawa, C. and Ferri, C. and Uchoa, E. and Firmo, J. and Lima-Costa, M. F. and Dewey, M. E. and Stewart, R.	Dimensions Underlying the Mini-Mental State Examination in a Sample With Low-Education Levels: The Bambui Health and Aging Study
Castro-Costa, E. and Fuzikawa, C. and Uchoa, E. and Firmo, J. O. A. and Lima-Costa, M. F.	Norms for the mini-mental state examination - Adjustment of the cut-off point in population-based studies (evidences from the Bambui health aging study)
Cecato, Juliana FranICsca	Pentagon Drawing Test: some data from Alzheimer's disease, Paraphrenia and Obsessive compulsive disorder in elderly patients
Cecato, J. F. and Fiorese, B. and Montiel, J. M. and Bartholomeu, D. and Martinelli, J. E.	Clock drawing test in elderly individuals with different education levels: correlation with clinical dementia rating
Cecato, J. F. and Martinelli, J. E. and Izbicki, R. and Yassuda, M. S. and Aprahamian, I.	A subtest analysis of the Montreal cognitive assessment (MoCA): which subtests can best discriminate between healthy controls, mild cognitive impairment and Alzheimer's disease?
Cecato, Juliana FranICsca and Montiel, José Maria and Bartholomeu, Daniel and Martinelli, José Eduardo	MoCa predictive power in neuropsychological assessment of patients with dementia
Chachamovich, E. and Fleck, M. P. and Power, M.	Is Geriatric Depression Scale-15 a suitable instrument for measuring depression in Brazil? Results of a Rasch analysis
Chachamovich, E. and Fleck, M. P. and Trentini, C. and Power, M.	Brazilian WHOQOL-OLD Module version: a Rasch analysis of a new instrument
Chachamovich, E. and Trentini, C. and Fleck, M. P.	Assessment of the psychometric performance of the WHOQOL-BREF instrument in a sample of Brazilian older adults

Chaves Gde, F. and Oliveira, A. M. and Chaves, J. A. and Forlenza, O. V. and Aprahamian, I. and Nunes, P. V.	Assessment of impairment in activities of daily living in mild cognitive impairment using an individualized scale
Cintra, F. C. M. D. C., Cintra, M. T. G., Nicolato, R., Bertola, L., Ávila, R. T., Malloy-Diniz, L. F., Moraes, E. N., & Bicalho, M. A. C.	Functional decline in the elderly with MCI: Cultural adaptation of the ADCS-ADL scale
Correia, Clarice Camara and Lima, Fabia and Junqueira, Franco and Campos, Marilia Siqueira and Bastos, Othon and Petribu, Katia and Laks, Jerson and Galvin, James E.	AD8-Brazil: Cross-Cultural Validation of the Ascertaining Dementia Interview in Portuguese
Maia, A. L. G. and Godinho, C. and Ferreira, E. D. and Almeida, V. and Schuh, A. and Kaye, J. and Chaves, M. L. F.	Application of the Brazilian version of the CDR scale in samples of dementia patients
Martins, S. P. and Damasceno, B. P.	Accuracy of prospective memory tests in mild Alzheimer's disease
Massena, P. N. and de Araujo, N. B. and Pachana, N. and Laks, J. and de Padua, A. C.	Validation of the Brazilian Portuguese Version of Geriatric Anxiety Inventory - GAI-BR
Memoria, C. M. and Yassuda, M. S. and Nakano, E. Y. and Forlenza, O. V.	Brief screening for mild cognitive impairment: validation of the Brazilian version of the Montreal cognitive assessment
Memoria, C. M. and Yassuda, M. S. and Nakano, E. Y. and Forlenza, O. V.	Contributions of the Computer-Administered Neuropsychological Screen for Mild Cognitive Impairment (CANS-MCI) for the diagnosis of MCI in Brazil
Miranda, Diane da Costa and Brucki, Sonia Maria Dozzi and Yassuda, Mónica Sanches	The Mini-Addenbrooke's Cognitive Examination (M-ACE) as a brief cognitive screening instrument in Mild Cognitive Impairment and mild Alzheimer's disease
Montano, M. B. and Ramos, L. R.	Validity of the Portuguese version of Clinical Dementia Rating

Matias, A. G., Fonsêca, M.deA., Gomes, M.deL., & Matos, M. A.	Indicators of depression in elderly and different screening methods
Costa, Erico and Barreto, Sandhi M. and Uchoa, Elizabeth and Firmo, Joselia O. and Lima-Costa, Maria Fernanda and Prince, Martin	Is the GDS-30 better than the GHQ-12 for screening depression in elderly people in the community? The Bambui Health Aging Study (BHAS)
Moura, S.M., Haase V.G.	Psychometric properties and normative data of the Three Words-Three Shapes Test (3P3F) in Brazil
Novelli, Marcia M. and Nitrini, Ricardo and Caramelli, Paulo	Validation of the Brazilian version of the Quality of Life Scale for Patients with Alzheimer's Disease and their caregivers (QOL-AD)
Paradela, Emylucy Martins Paiva and Lourenco, Roberto Alves	Is the Cambridge Cognitive Examination - Revised a good tool for detection of dementia in illiterate Brazilian older adults?
Paradela, E. M. and Lopes Cde, S. and Lourenco, R. A.	Reliability of the Brazilian version of the Cambridge Cognitive Examination Revised CAMCOG-R
Paradela, E. M. P. and Lourenco, R. A. and Veras, R. P.	Validation of geriatric depression scale in a general outpatient clinic
Paschoal, S. M. P. and Jacob Filho, W. and Litvoc, J.	Development of Elderly Quality of Life Index – EqoLI: item reduction and distribution into dimensions
Paula, J. J. and Bertola, L. and Avila, R. T. and Assis Lde, O. and Albuquerque, M. and Bicalho, M. A. and Moraes, E. N. and Nicolato, R. and Malloy-Diniz, L. F.	Development, validity, and reliability of the General Activities of Daily Living Scale: a multidimensional measure of activities of daily living for older people
Paula, Jonas Jardim de and Melo, Larissa Pacheco Cunha and Nicolato, Rodrigo and Moraes, Edgar Nunes de and Bicalho, Maria ApareICda and Hamdan, Amer Cavalheiro and Malloy-Diniz, Leandro Fernandes	Fidedignidade e validade de construto do Teste de Aprendizagem Auditivo-Verbal de Rey em idosos brasileiros

Perroco, Tibor Rilho and Damin, Antonio Eduardo and Frota, Norberto A. and Silva, Mari-Nilva M. and Rossi, Viviane and Nitrini, Ricardo and Bottino, Cassio M.	Short IQCODE as a screening tool for MCI and dementia: Preliminary results
Perroco, T. R. and Bustamante, S. E. and Moreno Mdel, P. and Hototian, S. R. and Lopes, M. A. and Azevedo, D. and Litvoc, J. and Filho, W. J. and Bottino, C. M.	Performance of Brazilian long and short IQCODE on the screening of dementia in elderly people with low education
Pinho, M. X. and Custodio, O. and Makdisse, M. and Carvalho, A. C. C.	Reliability and Validity of the Geriatric Depression Scale in Elderly Individuals with Coronary Artery Disease
Pinto, T. C. C. and Machado, L. and Bulgacov, T. M. and Rodrigues, A. L. and Costa, M. L. G. and Ximenes, R. C. C. and Sougey, E. B.	Influence of Age and Education on the Performance of Elderly in the Brazilian Version of the Montreal Cognitive Assessment Battery
Ribeiro Filho, S. T., & Lourenço, R. A.	The performance of the Mini-Cog in a sample of low educational level elderly
Tinoco, M. A., Gouveia, E. R., Ihle, A., & Marques, A.	The Cognitive Telephone Screening Instrument (COGTEL): a reliable and valid tool for the assessment of cognitive functioning in the Brazilian elderly
Scazufca, M. and Almeida, O. P. and Vallada, H. P. and Tasse, W. A. and Menezes, P. R.	Limitations of the Mini-Mental State Examination for screening dementia in a community with low soICoeconoMCI status
Sanchez, M. A. and Lourenco, R. A.	Informant Questionnaire on Cognitive Decline in the Elderly (IQCODE): cross-cultural adaptation for use in Brazil
Sanchez, M. A. and Lourenco, R. A.	Screening for dementia: Brazilian version of the Informant Questionnaire on Cognitive Decline on the Elderly and its psychometric properties
Scazufca, M. and Menezes, P. R. and Vallada, H. and Araya, R.	Validity of the self reporting questionnaire-20 in epidemiological studies with older adults: results from the Sao Paulo Ageing & Health Study

Schultz, R. and Siviero, M. and BertolucIC, P.	The cognitive subscale of the "Alzheimer's Disease Assessment Scale" in a Brazilian sample
Silva, P. A. B. and Soares, S. M. and Santos, J. F. G. and Silva, L. B.	Cut-off point for WHOQOL-bref as a measure of quality of life of older adults
Silva, S. M. and Santana, A. N. C. and da Silva, N. N. B. and Novaes, Mrcg	VES-13 and WHOQOL-bref cutoff points to detect quality of life in older adults in primary health care
Silva, Ldve and de Oliveira, G. M. and Yokomizo, J. E. and Saran, L. F. and Bottino, C. M. D. and Yassuda, M. S.	The Geriatric Anxiety Inventory in primary care: applicability and psychometric characteristics of the original and short form
Santos, M. T. F. and Sougey, E. B. and Alchieri, J. C.	VALIDITY AND RELIABILITY OF THE SCREENING TEST FOR ALZHEIMER'S DISEASE WITH PROVERBS (STADP) FOR THE ELDERLY
Nunes, Paula V. and Diniz, Breno S. and Radanovic, MarICa and Abreu, Izabella D. and Borelli, Danilo T. and Yassuda, Monica S. and Forlenza, Orestes V.	CAMCOG as a screening tool for diagnosis of mild cognitive impairment and dementia in a Brazilian clinical sample of moderate to high education
Paradela, E. M. and Lopes Cde, S. and Lourenco, R. A.	Portuguese adaptation of the Cambridge Cognitive Examination-Revised in a public geriatric outpatient clinic
Portugal Mda, G. and Coutinho, E. S. and Almeida, C. and Barca, M. L. and Knapskog, A. B. and Engedal, K. and Laks, J.	Validation of Montgomery-Asberg Rating Scale and Cornell Scale for Depression in Dementia in Brazilian elderly patients
Cristovão Ribeiro, C., Liberalesso Neri, A., & Sanches Yassuda, M.	Semantic-cultural validation and internal consistency analysis of the Purpose in Life Scale for brazilian older adults
Rodrigues, G. R., Oliveira, D. S., Foss, M. P., & Takayanagui, O. M.	Cross-cultural adaptation and validation of the episodic autobiographic memory interview for Brazilian Portuguese

Simon, Sharon Sanz and Ávila, Renata Thomas and Vieira, Gilson and Bottino, Cássio Machado de Campos	Metamemory and aging: Psychometric properties of the Brazilian version of the Multifactorial Memory Questionnaire for elderly
Sousa, R. M. and Scazufca, M. and Menezes, P. R. and Crepaldi, A. L. and Prince, M. J.	Feasibility and reliability of the elderly version of the Camberwell Assessment of Needs (CANE): results from the Sao Paulo Ageing & Health Study
Ventura, M. and Bottino, C. M.	[Reliability study of the Brazilian version of a structured interview for the diagnosis of dementia]
Yassuda, M. S. and Flaks, M. K. and Viola, L. F. and Pereira, F. S. and Memoria, C. M. and Nunes, P. V. and Forlenza, O. V.	Psychometric characteristics of the Rivermead Behavioural Memory Test (RBMT) as an early detection instrument for dementia and mild cognitive impairment in Brazil
Vitorino, L. M. and Low, G. and Vianna, L. A. C.	Assessing the BRIEF spiritual/religious coping scale among older Brazilians
Zucoloto, Miriane LuICndo and Santos, Scarlet Feitosa and Terada, Natalia Akemi Yamada and Martinez, Edson Zangiacomi	Construct and/or Criterion Validity of the Brazilian version of the Medical Outcomes Studysocial Support Survey (MOS-SSS) in a sample of elderly users of the primary healthcare system
Diniz, Juliana GarICa and da Silva, Alfredo Carlos and Nobrega, Ana Caline	Quality of life and swallowing questionnaire for individuals with Parkinson's disease: Development and validation
Saccomann, Izabel C. and ICntra, Fernanda A. and Gallani, Maria CeIClia B.	Psychometric properties of the Minnesota Living with Heart Failure--Brazilian version--in the elderly
Saraiva, M. D. and Venys, A. and Abdalla, F. and Bianconi, B. and Sousa, D. and Henrique, E. and Fernandes, M. and Pisoli, P. and Cavalheiro, M. and Suzuki, G. and Serrano, P. and Mazza, M. and Lima, L. and Hiratsuka, M. and Jacob-Filho,	AMPI-AB accuracy: A multidimensional questionnaire for the management of the public healthcare for older people in the city of SaO Paulo, Brazil
Reichenheim, M. E. and Paixao, C. M., Jr. and Moraes, C. L.	Reassessing the Construct and/or Criterion Validity of a Brazilian version of the instrument Caregiver Abuse Screen (CASE) used to identify risk of domestic violence against the elderly

Reichenheim, M. and Sanchez, M. A. D. and Lourenco, R. A.	Re-assessing the dimensional structure of the Informant Questionnaire on Cognitive Decline in the Elderly (IQCODE): empirical evidence for a shortened Brazilian version
Prigatano, G. P. and Souza, L. M. N. and Braga, L. W.	Performance of a Brazilian sample on the Portuguese translation of the BNI Screen for Higher Cerebral Functions
Nunes DP, Brito TRP, Corona LP, Alexandre TS, Duarte YAO.	Elderly and caregiver demand: proposal for a care need classifi cation
Baptista, M. N., Santos, L. M., & Filho, N. H	Bifactor analysis of the Baptista's Depression Scale
Folquitto, Jefferson C. and Bustamante, Sonia E. and Barros, Sergio B. and Azevedo, Dionisio and Lopes, Marcos A. and Hototian, Sergio R. and Filho, Wilson Jacob and Litvoc, Julio and Bottino, Cassio M.	The Bayer-Activities of Daily Living scale (B-ADL) in the differentiation between mild to moderate dementia and normal aging
FranICsca Cecato, Juliana and Galeote, Livia and Eduardo Martinelli, Josao	Sensitivity of visuoconstructive praxis from MoCA in Mild Cognitive Impairment and Alzheimer's disease
Cesar, Karolina G. and Brucki, Sonia M. and Takada, Leonel T. and NasICmento, Luiz Fernando C. and Gomes, Camila M. and Almeida, Milena C. and Oliveira, Maira O. and Porto, Fabio H. and Senaha, Mirna L. and Bahia,	Performance of the Visual Analogue Scale of Happiness and of the Cornell Scale for Depression in Dementia in the Tremembe Epidemiological Study, Brazil
Cesar, Karolina G. and Yassuda, MÃ´nica S. and Porto, Fabio H. G. and Brucki, Sonia M. D. and Nitrini, Ricardo	MoCA Test: normative and diagnostic accuracy data for seniors with heterogeneous educational levels in Brazil
Faria, Cdcm and Teixeira-Salmela, L. F. and NasICmento, V. B. and Costa, A. P. and Brito, N. D. P. and Rodrigues-De-Paula, F.	Comparisons between the Nottingham Health Profile and the Short Form-36 for assessing the quality of life of community-dwelling elderly
Pinto, T. C. C. and MacHado, L. and Costa, M. L. G. and Santos, M. S. P. and Bulgacov, T. M. and Rolim, A. P. P. and Silva, G. A. and Rodrigues-Junior, A. L. and Sougey, E. B. and Ximenes, R. C. C.	Accuracy and Psychometric Properties of the Brazilian Version of the Montreal Cognitive Assessment as a Brief Screening Tool for Mild Cognitive Impairment and Alzheimer's Disease in the Initial Stages in the Elderly

Araujo, L. G. and Lima, D. M. F. and Sampaio, R. F. and Pereira, L. S. M.	Pain Locus of control scale: adaptation and reliability for elderly
Simon, S. S. and Avila, R. and Vieira, G. and Campos Bottino, C. M.	Brazilian version of the multifactorial memory questionnaire for older adults: Preliminary results
Cecato, Juliana Francisca, Balduino, Everton, Martinelli, José Eduardo, Aprahamian, Ivan.	Brief version of the CAMCOG for illiterate older adults with Alzheimer's dementia
Tavares-Júnior, José Wagner Leonel, Braga-Neto, Pedro, Bonfadini, Janine de Carvalho, Bittencourt, Lays, Lopes, Candida Helena, Mendes, Larissa, Siqueira-Neto, José Ibiapina, Sousa, Valéria, Amaral, Anina, Carrilho, Carolina	Clinical characteristics and diagnostic accuracy of the revised Addenbrooke Cognitive Examination (ACE-R) in older adults with a low educational level
Rebellato, C., Fontaine, A.M.G.V., Matsukura, T.S.	Social participation of Independent Older Adults: Analysis of the Items of the LIFE-H 3.1-Brazil
Lima-Silva, T.B., Mioshi, E., Bahia, V.S., Cecchini, M.A., Cassimiro, L., Guimarães, H.C., Gambogi, L.B., Caramelli, P., Balthazar, M., Damasceno, B., Brucki, S.M.D., de Souza, L.C., Nitrini, R., Yassuda, M.S.	Disease Progression in Frontotemporal Dementia and Alzheimer Disease: The Contribution of Staging Scales
Ferreira da Mata, LuICana Regina, Prette Kuznier, Tatiane, Carrilho Menezes, Aline, Azevedo, ICssa, Amorim Amaral, FabríICa Moreira, Machado Chianca, Tânia Couto.	Validity and reliability of the UCLA Loneliness Scale version 3 among aged Brazilians
Ricardo Zibetti, Murilo and Hermes Pereira, Andressa and Julia Lehnen, Ana and Zuneda, Gabriela and Machado, Fabiula and Kochhann, Renata and Paz Fonseca, Rochele and Marceli Trentini, Clarissa	Brazilian adaptation of pictoric Free and Cued Selective Reminding Test with Immediate Recall (pFCSRT-IR)
Veras, Carolina and Hartle, Larissa and Araujo, Verónica C. and Charchat-Fichman, Helenice	Normative Data of Geriatric Depression Scale in Community-Dwelling Elderly Sample in Rio de Janeiro
Cecato, Juliana, Elia Fuentes, Débora, Eduardo Martinelli, José.	Evidence of validity for the Bender Gestalt Test: normative data in the neuropsychological evaluation of Brazilian elderly in Alzheimer's disease and Vascular Dementia

Lins, G. O. A., Lima, N. A. D. S., Sousa, G. S., Guimarães, F. J., Frazão, I. D. S., & Perrelli, J. G. A.	Validity and reliability of Kessler Psychological Distress Scale for Brazilian elderly: a cross-sectional study
Neves, Tatiana Reis Fabiano, de Araújo, Narahyana Bom, de Oliveira Silva, Felipe, Ferreira, José ViníICus Alves, Nielsen, Thomas Rune, Engedal, Knut, Laks, Jerson, Deslandes, Andrea Camaz.	Accuracy of the semantic fluency test to separate healthy old people from patients with Alzheimer's disease in a low education population
Lopes, Josiane, Araújo, Hayslenne Andressa Gonçalves de Oliveira, Smaili, Suhaila Mahmoud.	Fatigue in Parkinson's disease: Brazilian validation of the modified fatigue impact scale
Studart-Neto, Adalberto, Moraes, Natália Cristina, Spera, Raphael Ribeiro, Merlin, Silvia Stahl, Parmera, Jacy Bezerra, Jaluul, Omar, SanchesYassuda, Mônica, Brucki, Sonia Maria Dozzi, Nitrini, Ricardo.	Translation, cross-cultural adaptation, and validity of the Brazilian version of the Cognitive Function Instrument
Ferreira, L.K. and Filgueiras Meireles, J.F. and de Oliveira Gomes, G.A. and Caputo Ferreira, M.E.	Development and Psychometric Evaluation of a Lifestyle Evaluation Instrument for Older Adults
Ferreira, J.D.R., Miranda, M.F., Miranda, M.F., Romano-Silva, M.A., Bicalho, M.A.C., Viana, B.M.	Translation and validity of the Multidimensional Individual and Interpersonal Resilience Measure
Rodrigues, R.C.S., de Araújo-Monteiro, G.K.N., Marcolino, E.C., Brandão, B.M.L.S., Barbosa, L.A., de Moraes, R.M., Souto, R.Q.	ABUSE AGAINST THE ELDERLY PERSON: ANALYSIS OF THE INTERNAL CONSISTENCY OF INSTRUMENTS
ForaICepe, M. and Silva, A.E.V.F. and Fares, T.G. and Santos, F.C.	Pain in older adults with dementia: Brazilian validation of Pain Intensity Measure for Persons with Dementia (PIMD)
Amaral-Carvalho, V., Lima-Silva, T.B., Mariano, L.I., De Souza, L.C., Guimarães, H.C., Bahia, V.S., Nitrini, R., Barbosa, M.T., Yassuda, M.S., Caramelli, P.	Brazilian Version of Addenbrooke's Cognitive Examination - Revised in the Differential Diagnosis of Alzheimer'S Disease and Behavioral Variant Frontotemporal Dementia
de Oliveira, C.R. and de Lima, M.M.B.M.P. and Barroso, S.M. and de Lima Argimon, I.I.	Psychometric properties of the Dysexecutive Questionnaire (DEX): a study with Brazilian older adults

de Castro, V.C. and Radovanovic, C.A.T. and Dellaroza, M.S.G. and Pedroso, B. and Silva, E.S. and Carreira, L.	Construct and/or Criterion Validity and internal consistency of the Brazilian version of Leisure Attitude Measurement for the elderly
Lins, G.O.A., Lima, N.A.D.S., de Sousa, G.S., Guimarães, F.J., Frazão, I.D.S., Perrelli, J.G.A.	Validity and reliability of Kessler Psychological Distress Scale for Brazilian elderly: a cross-sectional study
Ferreira, O.D.L. and Barbosa, L.N.F. and Alchieri, J.C.	Validity Evidences of the Prefrontal Symptoms Inventory for the Elderly Brazilian Population
De Melo, D.M. and Barbosa, A.J.G. and De Castro, N.R. and Neri, A.L.	Mini-mental state examination in brazil: An item response theory analysis
Carrasco, M. P. and Villarroel, L. and Andrade, M. and Calderon, J. and Gonzalez, M.	Development and validation of a delirium predictive score in older people
Espinoza, I., Osorio, P., Torrejon, M. J., Lucas-Carrasco, R., & Bunout, D.	Validation of the WHOQOL-BREF quality of life questionnaire among Chilean older people
Fornazzari, L. and Cumsille, F. and Quevedo, F. and Quiroga, P. and Rioseco, P. and Klaasen, G. and Martinez, C. and Rhode, G. and Sacks, C. and Rivera, E. and Gassic, I. and Hammersley, F. and Hoppe, A. and Arriagada, P. and Flaskamp, R.	Spanish validation of the Syndrom Kurztest (SKT)
Gallardo-Peralta, L. and Cuadra-Peralta, A. and Camara-Rojo, X. and Gaspar-Delpino, B. and Sanchez-Lillo, R.	Validation of the successful aging inventory in chilean older people
Gallardo-Peralta, Lorena P. and Cuadra-Peralta, Alejandro and Veloso-Besio, Constanza	Validation of a Brief Index of Religiosity and Spirituality Among Elderly People
Gallardo-Peralta, Lorena P. and Luis Gálvez-Nieto, Jose	Validation of the Community Support Questionnaire among Elderly Chilean People

Hoyl, T. and Valenzuela, E. and Marin, P. P.	Depression in the aged: preliminary evaluation of the effectiveness, as an screening instrument, of the 5-item version of the Geriatric Depression Scale. [Spanish]
Iturra-Mena, A. M.	Adaptation and preliminary validation of a screening test for dementia in Chile: The eurotest.
Jimenez, D. and Lavados, M. and Rojas, P. and Henriquez, C. and Silva, F. and Guillon, M.	Performance of an abbreviated mini mental examination to detect dementia in older people
Lera, L. and Fuentes-GarICa, A. and Sanchez, H. and Albala, C.	Validity and reliability of the SF-36 in Chilean older adults: the ALEXANDROS study
Miranda-Castillo, C. and Contreras, D. and Garay, K. and Martinez, P. and Leon-Campos, M. O. and Farhang, M. and Moran, J. and Fernandez-Fernandez, V.	Validation of the Geriatric Anxiety Inventory in Chilean older people
Diaz, E.M., Moraga, E.F., Soromaa, H	Reliability and Construct and/or Criterion Validity of Munsch test to measure happiness in the Elderly chilean population
Munoz-Neira, C. and Henriquez Chaparro, F. and Delgado, C. and Brown, J. and Slachevsky, A.	Test Your Memory-Spanish version (TYM-S): a validation study of a self-administered cognitive screening test
Muñoz-Neira, C., Henríquez Ch, F., Ihnen J, J., Sánchez C, M., Flores M, P., & Slachevsky Ch, A.	Psychometric properties and diagnostic usefulness of the Addenbrooke's Cognitive Examination-Revised in a Chilean elderly sample
Pinto Santuber, C. and Lara Jaque, R. and Espinoza Lavoz, E. and Montoya Cáceres, P.	Psychometric properties of the scale perceivedsocial support Zimet in older adults of Primary Health Care
Quiroga, P. and Albala, C. and Klaasen, G.	Validation of a screening test for age assoICated cognitive impairment, in Chile]

Urzua, A. and Navarrete, M.	Factor analysis of abbreviated versions of the WHOQoL-Old in Chilean older people
Gallardo-Peralta, L.P., Sánchez-Iglesias, I., Galdós, J.S., Roda, A.B.L.D., Sánchez-Moreno, Y.E.	Validation of Perceivedsocial Support Questionnaire for a multi-ethnic population of Chilean older adults
Grandi, Fabrissio, Martínez-Pernía, David, Parra, Mario, Olavarria, Loreto, Huepe, David, Alegria, PatriICa, Aliaga, Álvaro, Lillo, PatriICa, Delgado, Carolina, Tenorio, Marcela, Rosas, Ricardo, López, Oscar, Becker, James, Slachevsky, Andrea.	Standardization and diagnostic utility of the Frontal Assessment Battery for healthy people and patients with dementia in the Chilean population
Bello-Lepe, S, Alonso-Sánchez, MF, Ortega, A, Gaete, M, Veliz, M, Lira, J, Salas, CPP.	Montreal Cognitive Assessment as Screening Measure for Mild and Major Neurocognitive Disorder in a Chilean Population
Gallardo-Peralta, LP, Rodríguez-Blázquez, C, Ayala-García, A, Fotjaz, MJ.	VALIDATION OF THE BRIEF RESILIENT COPING SCALE (BRCS) IN A MULTIETHNIC SAMPLE OF CHILEAN OLDER PEOPLE
Jorquera-Cox, M., Leiva-Gutiérrez, J., Gutiérrez-Carmona, A., Ardiles-Irarrázabal, R.-A., Valdivia-Rojas, Y.	VALIDATION OF THE SPANISH VERSION OF P. REED'S SPIRITUAL PERSPECTIVE SCALE IN OLDER PEOPLE IN CHILE
Plaza-Troncoso, N., Juncos-Rabadán, O., Troncoso-Pantoja, C.	Adaptation and validation of aged adults adaptation scale to their residence (EAPAR) in Chile
CanICno, M., Rehbein, L., Gómez-Pérez, D., Ortiz, M.S.	Psychometric properties of three instruments to detect dementia
Caldichoury, N., Soto-Añari, M., Camargo, L., Porto, M.F., Herrera-Pino, J., Shelach, S., Rivera-Fernández, C., Ramos-Henderson, M., Gargiulo, P.A., López, N.	Clinical utility of Phototest via teleneuropsychology in Chilean rural older adults
Aslan, Joseph, Cova, Félix, Saldivia, Sandra, Bustos, Claudio, Inostroza, Carolina, Rincón, Paulina, Ortiz, Camila, Bühring, Vasily.	Psychometric Properties of the Patient Health Questionnaire-9 in Elderly Chilean Primary Care Users.

Sepúlveda-Ibarra, C., Chaparro, F.H., Marcotti, A., Soto, G., Slachevsky, A.	Normalization of Rowland Universal Dementia Assessment Scale (RUDAS) in Chilean older people
Bello-Lepe, S., Alonso-Sánchez, M.F., Perez-Salas, C.P., Veliz, M., Gaete, M., Lira, J.	The efficacy of the picture version of the free and cued selective reminding test to detect significant neurocognitive defiICts in a Chilean population
Pérez-Villalobos, C., Briede-Westermeyer, J.C., Schilling-Norman, M.J., Contreras-Espinoza, S.	Multidimensional scale of perceivedsocial support: evidence of validity and reliability in a Chilean adaptation for older adults
Lera, L., Marquez, C., Saguez, R., Moya, M.O., Angel, B., Albala, C.	Quality of life of older people with depression and dependence: validity of the SF-12 (short form health survey) questionnaire
Rodríguez-Blázquez, C., Ayala-García, A., Forjaz, M.J., Gallardo-Peralta, L.P.	Validation of the De Jong Gierveld Loneliness Scale, 6-item version, in a multiethnic population of Chilean older adults
Calderón, C., Beyle, C., Véliz-GarICá, O., Bekios-Calfa, J.	Psychometric properties of Addenbrooke's Cognitive Examination III (ACE-III): An item response theory approach
Thumala-Dockendorff, D. and Assar, R. and Wenk, E. and Arnold-Cathalifaud, M. and Villagra, R. and Lillo, P. and Slachevsky, A.	Construction and validation of a scale of losses experienced in old age (SLO)
Gallardo-Peralta, L.P., Rodríguez-Blázquez, C., Ayala-García, A., Forjaz, M.J.	Multi-ethnic validation of 15-item Geriatric Depression Scale in Chile
García, Ó.V., Carvajal, C.C., Sandoval, C.B	Psychometric properties of the addenbrooke's cognitive examination III (ACE-III) for the detection of dementia
Vera-Calzaretta, A., Klaassen, G., Medel, J.P.B., Contreras, L., Werlinger, E., Gonzalez-Burboa, A., Salazar, O., Fuentealba, M., Juica, S.	Spanish translation, retranslation, and content validation of the Quality of Life in Alzheimer's Disease scale in patients with Alzheimer's dementia

Schnettler, B. and Miranda-Zapata, E. and Lobos, G. and Lapo, M. D. and Adasme-Berrios, C. and Hueche, C.	Measurement invariance in the Satisfaction with Life Scale in Chilean and Ecuadorian older adults
Bacca, Angela Maria and Gonzalez, Angelica and Uribe Rodriguez, Ana Fernanda	Validation of the Scale of Yesavage (reduced version) in Colombian olders
Bastidas-Bilbao, Hamer and Gonzalez, G. and Camacho, Leonidas Castro	Psychometric properties of the Spanish versions of the BAS-DEP and EBAS-DEP scales as screening instruments for depression in a multisite geriatric Colombian sample: An exploratory study
Campo-Arias, Adalberto and Urruchurtu Mendoza, Yorjany and Solano Morales, Tharim	Internal consistency and exploratory factorial analysis of the Yesavage Geriatric Depression Scale (GDS-15) in Cartagena (Colombia)
Cantor Nieto, Martha Isabel, & Avendaño Prieto, Bertha Lucía.	Psychometric properties of a screening test for dementia Pesotest in clinical and non-clinical samples of elderly people
Cardona Jiménez, Jairo León and Villamil Gallego, María Mercedes and Henao Villa, Eucaris and Quintero Echeverri, Ángela	ESTE scale validation to measure loneliness in adult population
Gil, L. and Ruiz de Sanchez, C. and Gil, F. and Romero, S. J. and Pretelt Burgos, F.	Validation of the Montreal Cognitive Assessment (MoCA) in Spanish as a screening tool for mild cognitive impairment and mild dementia in patients over 65 years old in Bogota, Colombia
Gomez, F. and Zunzunegui, M. V. and Lord, C. and Alvarado, B. and GarICa, A.	Applicability of the MoCA-S test in populations with little education in Colombia
Gómez-Angulo, Carine and Campo-Arias, Adalberto	Escala de Yesavage para Depresión Geriátrica (GDS-15 y GDS-5): estudio de la consistenICa interna y estructura factorial
Cerquera Córdoba, Ara Mercedes and Cala Rueda, María Lucía and Galvis ApariICo, Mayra Juliana	Construct Validation of the ESTE-R Scale for Measuring Loneliness in Old Age in Bucaramanga, Colombia

Melguizo-Herrera, E., Álvarez-Romero, Y., Cabarcas-Mendoza, M. V., Calvo-Rodríguez, R. S., Flórez-Almanza, J., Moadie-Contreras, O. P., & Campo-Arias, A.	Validity and Reliability of the Attitudes Toward Sexuality in the Elderly Questionnaire in Cartagena, Colombia
Rojas-Gualdron, D. F. and Segura, C. A. and Cardona, A. D. and Segura, C. A. and Garzon, D. M. O.	Rasch analysis of the Mini Mental State Examination (MMSE) in older adults in Antioquia, Colombia
Rojas-Gualdrón, Diego Fernando, Díaz Gordon, PatriICa, Jaramillo Ortegón, Diana PatriICa, Ortega Ortiz, Martha Eugenia, Castellanos Ruiz, Julialba, & González Marín, Andrea del Pilar.	Rasch analysis of the WHOQOL-BREF in older adults from Bucaramanga and Manizales
Zenger, M. and Finck, C. and Zanon, C. and Jimenez, W. and Singer, S. and Hinz, A.	Evaluation of the Latin American version of the Life Orientation Test-Revised
Ruelas-González, María Guadalupe, Obando Guerrero, Lina M., Betancourth Zambrano, Sonia, Monterrubio-Flores, Eric, Ojeda Rosero, Elizabeth, Saturno Hernández, Pedro J.	Adaptation and validation of the Screening Questionnaire for Family Abuse of the Elderly in the soICocultural context of Colombia
Bonilla-Santos, J., González-Hernández, A., Sierra-Barón, W., Gómez-Acosta, A., Cala-Martínez, D.Y.	Evidence of validity and reliability of the Colombian version of Addenbroke's Cognitive Examination Revised (ACE-R)
Ramos, C., Pulido, J., Bedoya, J.D., Madrigal, C., Giraldo, M., Alzate, D., Aguirre-Acevedo, D.C., Lopera, F., García, J.	Validation of the Anosognosia Questionnaire in Dementia in Individuals With Major Neurocognitive Disorder in COLOMBIA
Franco, J.G., Trzepacz, P.T., Sepúlveda, E., Ocampo, M.V., Velásquez-Tirado, J.D., Zaraza, D.R., Restrepo, C., Giraldo, A.M., Serna, P.A., Zuluaga, A., López, C.	Delirium diagnostic tool-provisional (DDT-Pro) scores in delirium, subsyndromal delirium and no delirium
Clavijo-Moran, HJC, Alvarez-García, D, Pinilla-Monsalve, GD, Muñoz-Ospina, B, Orozco, J.	Psychometric properties and Construct and/or Criterion Validity of the Parkinson's Disease-Cognitive Rating Scale (PD-CRS) in Colombia
Campo-Arias, A. and Reyes-Ortiz, C.A.	Psychometric Performance of the Memory Complain Scale among Colombian Individuals of 60 Years and Older

Franco, J.G., Ocampo, M.V., Velásquez-Tirado, J.D., Zaraza, D.R., Giraldo, A.M., Serna, P.A., López, C., Zuluaga, A., Sepúlveda, E., Kean, J., Trzepacz, P.T	Validation of the delirium diagnostic tool-provisional (DDT-Pro) with medical inpatients and comparison with the confusion assessment method algorithm
Valdivieso-Mora, E. and Ivanisevic, M. and Shaw, L. A. and Garnier-Villarreal, M. and Green, Z. D. and Salazar-Villanea, M. and Moncada-Jimenez, J. and Johnson, D. K.	Health-Related Quality of Life of Older Adults in Costa Rica as Measured by the Short-Form-36 Health Survey
Villalobos, M.A.V., Ureña, B.S., Mora, C.A., Mora, L.S., Barquero, C.E.R.	Psychometric Properties of The Wagnild and Young Resilience Scale in Costa Rican Older Adults
Broche-Perez, Y. and Lopez-Pujol, H. A.	Validation of the Cuban Version of Addenbrooke's Cognitive Examination-Revised for Screening Mild Cognitive Impairment
Fernández-Fleites, Zoylen, Jiménez-Puig, Elizabeth, Broche-Pérez, Yunier, Morales-Ortiz, Sheyla, Luzardo, Darlyn Alejandra Reyes, Crespo-Rodríguez, Luis Ramón.	Evaluation of sensitivity and specificity of the INECO Frontal Screening and the Frontal Assessment Battery in mild cognitive impairment
Rodríguez-Salgado, Ana M, Llibre-Guerra, Jorge J, Tsoy, Elena, Peñalver-Guia, Ana Ibis, Bringas, Giosmany, Erlhoff, Sabrina J, Kramer, Joel H, Allen, Isabel Elaine, Valcour, Victor, Miller, Bruce L, Llibre-Rodríguez, Juan J, Possin, Katherine L.	A Brief Digital Cognitive Assessment for Detection of Cognitive Impairment in Cuban Older Adults.
Serrano-Duenas, M. and Martinez-Martin, P. and Vaca-Baquero, V.	Validation and cross-cultural adjustment of PDQL-questionnaire, Spanish version (Ecuador) (PDQL-EV)
Erazo, M., Fors, M., Mullo, S., González, P., Viada, C.	Internal Consistency of Yesavage Geriatric Depression Scale (GDS 15-Item Version) in Ecuadorian Older Adults.
Brailean, A. and Guerra, M. and Chua, K. C. and Prince, M. and Prina, M. A.	A multiple indicators multiple causes model of late-life depression in Latin American countries
Daskalopoulou, C. and Chua, K. C. and Koukounari, A. and Caballero, F. F. and Prince, M. and Prina, A. M.	Development of a healthy ageing index in Latin American countries - a 10/66 dementia research group population-based study

Guerra, M. and Ferri, C. and Llibre, J. and Prina, A. M. and Prince, M.	Psychometric properties of EURO-D, a geriatric depression scale: a cross-cultural validation study
Chua, K. C. and Bohnke, J. R. and Prince, M. and Banerjee, S.	Health-Related Quality-of-Life Assessment in Dementia: Evidence of Cross-Cultural Validity in Latin America
Virues-Ortega, Javier and Carod-Artal, FranICsco Javier and Serrano-Duenas, Marcos and Ruiz-Galeano, Gabriela and Meza-Rojas, Gloria and Velazquez, Carolina and MCIheli, Federico and Martinez-Martin, Pablo	Cross-cultural validation of the Scales for Outcomes in Parkinson's Disease-PsychoSocial Questionnaire (SCOPA-PS) in four Latin American countries
Virues-Ortega, J. and Rodriguez-Blazquez, C. and MCIheli, F. and Carod-Artal, F. J. and Serrano-Duenas, M. and Martinez-Martin, P.	Cross-Cultural Evaluation of the Modified Parkinson Psychosis Rating Scale Across Disease Stages
Sousa, R. M. and Dewey, M. E. and Acosta, D. and Jotheeswaran, A. T. and Castro-Costa, E. and Ferri, C. P. and Guerra, M. and Huang, Y. Q. and Jacob, K. S. and Pichardo, J. G. R. and Ramirez, N. G. and Rodriguez, J. L. and Rodriguez, M. C.	Measuring disability across cultures - the psychometric properties of the WHODAS II in older people from seven low- and middle-income countries. The 10/66 Dementia Research Group population-based survey
Sosa, Ana Luisa and Albanese, Emiliano and Prince, Martin and Acosta, Daisy and Ferri, Cleusa P. and Guerra, Mariella and Huang, Yueqin and Jacob, K. and de Rodriguez, Juan Llibre and Salas, Aquiles and Yang, Fang and	Population normative data for the 10/66 Dementia Research Group cognitive test battery from Latin America, India and China: A cross-sectional survey
Prince, M. and Acosta, D. and Chil, H. and Copeland, J. and Dewey, M. and Scazufca, M. and Varghese, M. and Dementia Res, Grp	Effects of education and culture on the validity of the Geriatric Mental State and its AGECAT algorithm
Méndez Chacón, Ericka.	Psychometric evaluation of the abbreviated scale of Yesavage depression in older adults in several ICties in Latin America: Studies SABE and CRELES
Acosta Quiroz, C., Vales García, J., Echeverría Castro, S., Serrano Encinas, D., García Flores, R.	Psychometric properties of the Quality of Life-Old Questionnaire (WHOQOL-OLD) in Mexican older adults
Aguilar-Navarro, S. G., Fuentes-Cantú, A., Ávila-Funes, J. A., García-Mayo, E. J.	Validity and reliability of the screening questionnaire for geriatric depression used in the Mexican Health and Age Study

Aguilar-Navarro, S., Mimenza-Alvarado, A.J., PalaICos-García, A., Samudio-Cruz, A., Gutiérrez-Gutiérrez, L., Ávila-Funes, J.	Validity and Reliability of the Spanish Version of the Montreal Cognitive Assessment (MoCA) for the Detection of Cognitive Impairment in Mexico
Aguilar-Navarro, S.G., Mimenza-Alvarado, A.J., Samudio-Cruz, M., Hernández-Contreras, F.J., Gutiérrez-Gutiérez, L.A., Ramírez-González, F., Avila-Funes, J.A.	Validation of the Clock Drawing Test Scoring Method in older adults with neurocognitive disorder
Beaman, Peter E. and Reyes-Frausto, Sandra and GarICa-Pena, Carmen	Validation of the Health Perceptions Questionnaire for an older Mexican population
Estrada, Marcela Sanchez and Forteza, Catalina Gonzalez and GarICa, Rebeca Robles and Palos, PatriICa Andrade	Development and psychometric evaluation of a spirituality index for elders in Mexico
Giraldo-Rodriguez, L. and Rosas-Carrasco, O.	Development and psychometric properties of the Geriatric Mistreatment Scale
Gonzalez Tovar, Jose and Martin Favela, Azeneth Guadalupe and Garza Sanchez, Rosa Isabel	Internal structure and convergence of a scale of hopelessness for older adults in Mexico
Hernández-Navor, Julio César and Guadarrama-Guadarrama, Rosalinda and Castillo-Arrellano, Sara Stephanie and Arzate Hernández, Giovanny and Márquez-Mendoza, Octavio	VALIDATION OF THE WHOQOL-OLD IN ELDERLY POPULATION OF MEXICO
Kantún-Marín, María Amparo de Jesús and Moral de la Rubia, José and Gómez-Meza, Marco ViniICo and Salazar-González, Bertha CeIClia	Validation of the Life Satisfaction Index for the third age
Longoria-Ibarrola, E. Mariana and Acosta-Castillo, G. Isaac and Rosales-Méndez, MauriICo and Andrade-Calderón, Paola and San Pedro-Caligua, Oscar and Sosa-Ortiz, Ana Luisa	Concurrent validation of the relevant outcome scale for Alzheimer’s disease (ROSA) in mexican patients
Mejia, S. and Gutierrez, L. M. and Villa, A. R. and Ostrosky-Solis, F.	Cognition, functional status, education, and the diagnosis of dementia and mild cognitive impairment in Spanish-speaking elderly

Mejia-Arango, S. and Wong, R. and MCIhaels-Obregon, A.	Normative and standardized data for cognitive measures in the Mexican Health and Aging Study
Salinas-Rodríguez, Aarón, Manrique-Espinoza, Betty, Acosta-Castillo, Gilberto Isaac, Franco-Núñez, Aurora, Rosas-Carrasco, Óscar, Gutiérrez-Robledo, Luis Miguel, & Sosa-Ortiz, Ana Luisa.	Validation of a cutoff point for the short version of the Depression Scale of the Center for Epidemiologic Studies in older Mexican adults
Sanchez-Arenas, R. and Vargas-Alarcon, G. and Sanchez-GarICa, S. and GarICa-Pena, C. and Gutierrez-Gutierrez, L. and Grijalva, I. and GarICa-Dominguez, A. and Juarez-Cedillo, T.	Value of EQ-5D in Mexican city older population with and without dementia (SADEM study)
Sánchez-García, S., Juárez-Cedillo, T., García-González, J. J., Espinel-Bermúdez, C., Gallo, J. J., Wagner, F. A., Vázquez-Estupiñán, F., & García-Peña, C.	Usefulness of two instruments in assessing depression among elderly Mexicans in population studies and for primary care
Rivera-Ledesma, Armando, Montero-López Lena, María, González-Celis Rangel, Ana Luisa, & Sánchez-Sosa, Juan José	AGEING ANXIETY SCALE THE LASHER AND FAULKENDER AGEING ANXIETY SCALE: PSYCHOMETRIC PROPERTIES IN MEXICAN OLDER ADULTS
Rosas-Carrasco, O. and Torres-Arreola, L. D. and Guerra-Silla, M. D. and Torres-Castro, S. and Gutierrez-Robledo, L. M.	Validation of the Quality of Life in Alzheimer's Disease (QOL-AD) scale in Mexican patients with Alzheimer, vascular and mixed-type dementia
Rivera-Ledesma, Armando and Lena, Maria Montero-Lopez	Measurements of religious coping and spirituality in older Mexican adults
Reyes-Ortega, M. and Soto-Hernandez, A. L. and Milla-Kegel, J. G. and GarICa-Ramirez, A. and Hubard-Vignau, L. and Mendoza-Sanchez, H. and Mejia-Garza, L. A. and GarICa-Pena, M. C. and Wagner-Echeagaray, F. A.	Revision of the Center for Epidemiologic Studies Depresion Scale (CES-D). Pilot study with a Mexican geriatric sample
Duarte Ayala, Rocío Elizabeth, Velasco Rojano, Ángel Eduardo.	Quality of Life Scale in Mexican Elderly: Psychometric validation
Núñez-Fernández, S., Rivera, D., Arroyo-Anlló, E.M., Ortiz Jiménez, X.A., Camino-Pontes, B., Salinas Martínez, R., Arango-Lasprilla, J.C.	Validation of the Norma Latina Neuropsychological Assessment Battery in Patients with Alzheimer's Disease in Mexico

Aguirre, S.I., Ornelas, M., Blanco, H., Jurado-García, P.J., Benavides, E.V., Rodríguez-Villalobos, J.M., Jiménez-Lira, C., Blanco, J.R.	Quality of Life in Mexican Older Adults: Factor Structure of the SF-36 Questionnaire
Mimenza-Alvarado, A.J., Bombón-Albán, P., Duarte-Flores, J.O., Gutiérrez-Gutiérrez, L., Ávila-Funes, J.A., Aguilar-Navarro, S.G.	Five-Word Test, Screening of Mixed Dementia in Older Adults. Validation Study
Aguilar-Navarro, Sara Gloria, Sánchez, Brenda Lorena Pillajo, Gutiérrez, Lidia Antonia Gutiérrez, Arias-Trejo, Natalia, Quiroz, Yakeel T, Alvarado, Alberto José Mimenza.	Cross-cultural adaptation of the everyday cognition scale (M-ECog) in older Mexican adults with cognitive impairment.
Giraldo-Rodríguez, Liliana, Álvarez-ICsneros, Teresa, Agudelo-Botero, Marcela.	Psychometric Properties of the 11-Item De Jong Gierveld Loneliness Scale in a Representative Sample of Mexican Older Adults.
Acosta Quiroz, Christian Oswaldo, García-Flores, Raquel, Echeverría-Castro, Sonia Beatriz.	The Geriatric Depression Scale (GDS-15): Validation in Mexico and Disorder in the State of Knowledge.
Giraldo-Rodríguez, L., López-Ortega, M.	Validation of the Short-Form 36 Health Survey (SF-36) for use in Mexican older persons
Zueck-Enríquez, M.C., Soto, M.C., Aguirre, S.I., Ornelas, M., Blanco, H., Peinado, J.E., Barrón-Luján, J.C., Aguirre, J.F.	Evidence of validity and reliability of the lasher and faulkender anxiety about aging scale in mexican older adults
Hernández-Soberón, J.C., Torres-Obregón, R., Carrillo Cervantes, A.L., Medina-Fernández, I.A., Sierra, J.C., Onofre-Rodríguez, D.J.	Psychometric properties of the Massachusetts General Hospital-Sexual Functioning Questionnaire in elderly people
Lazo-Porras, M. and Pesantes, M. A. and Miranda, J. J. and Bernabe-Ortiz, A.	Evaluation of cognitive impairment in elderly population with hypertension from a low-resource setting: Agreement and bias between screening tools
Caycho-Rodríguez, T., Reyes-Bossio, M., Ventura-León, J., Arias Gallegos, W. L., Domínguez-Vergara, J., & Azabache-Alvarado, K.	Psychometric evidence of a brief version of the Coping Humor Scale in elderly peruvians

Caycho-Rodriguez, Tomas and Ventura-Leon, Jose and Azabache-Alvarado, Karla and Barboza-Palomino, Miguel and Fergusson-Cardenas, Ingrid and Rojas-Jara, Claudio	Evidence psychometric initial of the Scale of Self-Efficacy for Aging (EAEE) in Peruvian elderly
Caycho-Rodríguez MSc, Tomás, Ventura-León MSc, José, García Cadena PhD, Cirilo H., Barboza-Palomino MSc, Miguel, Arias Gallegos MSc, Walter L., Dominguez-Vergara MSc, Julio, Azabache-Alvarado MSc, Karla, Cabrera-Orosco	Psychometric Evidence of the Diener's Satisfaction with Life Scale in Peruvian Elderly
Caycho-Rodriguez, T. and Ventura-Leon, J. and GarICa-Cadena, C. H. and Tomas, J. M. and Dominguez-Vergara, J. and Daniel, L. and Arias-Gallegos, W. L.	Psychometric evidence of a brief measure of resilience in non-institutionalized Peruvian older adults
Custodio, N. and Alva-Diaz, C. and Becerra-Becerra, Y. and Montesinos, R. and Lira, D. and Herrera-Perez, E. and Castro-Suarez, S. and Cuenca-Alfaro, J. and Valeriano-Lorenzo, E.	Performance of cognitive brief test in elderly patients with dementia in advanced stage living in an Urban community of Lima, Peru.
Custodio, N., García, A., Montesinos, R., Lira, D., & Bendezú, L.	Validation of the Clock Drawing Test - Manos version as screening test for detection of dementia in older people of Lima, Perú.
Custodio, Nilton and Lira, David and Herrera-Perez, Eder and Montesinos, Rosa and Castro-Suarez, Sheila and Cuenca-Alfaro, Jose and Valeriano-Lorenzo, LuICa	Memory alteration test to detect amnestic mild cognitive impairment and early Alzheimer's dementia in population with low educational level
Custodio, N. and Montesinos, R. and Lira, D. and Herrera-Perez, E. and Chavez, K. and Hernandez-Crdova, G. and Cuenca, J. and Gamboa, C. and Metcalf, T.	Validation of the RUDAS in Patients With a Middle-Level Education in Lima, Peru
Soto-Añari, Marcio, & Belón-HerIClla, María V.	Sensibility and specificity indicators for two cut-off points of the Mini Mental State Examination: A preliminary study
Zegarra-Valdivia, J., Denegri Solís, L., & Chino-Vilca, B.	Effectiveness of the photo-test front of the MMSE, for the screening of cognitive deterioration in Peruvian population.
Sancho, P. and Caycho-Rodriguez, T. and Ventura-Leon, J. and Tomas, J. M. and Reyes-Bossio, M.	Does the Spanish version of the SWLS measure the same in Spain and Peru?

Oscanoa, Teodoro	Clock drawing test in Alzheimer disease screening
Oscanoa, Teodoro J, ICeza, Edwin, Parodi, José F, & Paredes, Napoleón.	Evaluation of peruvian money test in screening of cognitive impairment among older adults
Custodio, N., Alva-Diaz, C., Becerra-Becerra, Y., Montesinos, R., Lira, D., Herrera-Pérez, E., Castro-Suárez, S., Cuenca-Alfaro, J., & Valeriano-Lorenzo, E.	Performance of cognitive brief test in elderly patients with dementia in advanced stage living in an Urban community of Lima, Peru
Custodio N, Malaga M, Montesinos R, Chambergo-MCIhilot D, Baca F, Carbajal JC, Huilca JC, Herrera-Perez E, Lira D, Diaz MM, Lanata S.	The Memory Alteration Test Is Correlated with Clinical, Cerebrospinal Fluid, and Brain Imaging Markers of Alzheimer Disease in Lima, Peru
Custodio, Nilton, Montesinos, Rosa, Lira, David, Herrera-Perez, Eder, Chavez, Kristhy, Reynoso-Guzman, Willyams, Pintado-Caipa, Maritza, Cuenca, José, Gamboa, Carlos, Metcalf, Tatiana.	Validation of the RUDAS for the Identification of Dementia in Illiterate and Low-Educated Older Adults in Lima, Peru
Campos-Vasquez, F., Valdez-Murrugarra, N., Soto-Tarazona, A., Camacho-Caballero, K., Rodriguez-Cuba, M.A., Parodi, J.F., Runzer-Colmenares, F.M.	Concordance between the Mini-Mental State Examination, Short Portable Mental Status Questionnaire and Montreal Cognitive Assessment Tests for Screening for Cognitive Impairment in Older Adults
Custodio, N., Montesinos, R., Cruzado, L., Herrera-Perez, E., Failoc-Rojas, V.E., Pintado-Caipa, M., Seminario G, W., Cuenca, J., Gamboa, C., Diaz, M.M.	Social Cognition and Behavioral Assessments Improve the Diagnosis of Behavioral Variant of Frontotemporal Dementia in Older Peruvians With Low Educational Levels
Marreros-Tananta, J., Guerrero-Alcedo, J.M.	Psychometric properties of the neuropsychological evaluation test – Neuropsi in peruvian population
Caycho-Rodríguez T, Vilca LW, Peña-Calero BN, Barboza-Palomino M, White M, Reyes-Bossio M.	Measurement of coronaphobia in older adults: Validation of the Spanish version of the Coronavirus Anxiety Scale.
Custodio, N, Montesinos, R, Alva-Diaz, C, Pacheco-Barrios, K, Rodriguez-Calienes, A, Herrera-Pérez, E, Becerra-Becerra, Y, Castro-Suárez, S, Pintado-Caipa, M, del Castillo, RC, Cuenca, J, Lira, D	Diagnostic accuracy of brief cognitive screening tools to diagnose vascular cognitive impairment in Peru

Caycho-Rodríguez, T., Carbajal-León, C., Vilca, L.W., Reyes-Bossio, M., Gallegos, M., Esteban, R.C., Noe-Grijalva, M., Gallegos, W.L.A., Delgado-Campusano, M., Muñoz-del-Carpio-Toia, Á.	Impact of COVID-19 on quality of life in Peruvian older adults: Construct and/or Criterion Validity, reliability and invariance of the COV19—Impact on Quality of Life (COV19-QoL) measurement
Livia-Segovia, J. and Grasso, L. and Herrera-Pino, A.D. and Ortiz-Morán, M. and Benavides-Munarriz, N.	Standardization of the Semantic Memory Evaluation Battery (EMSEA) in institutionalized elderly adults of Lima
Caycho-Rodríguez, T., Tomás, J.M., Hontangas, P.M., Ventura-León, J., Burga-León, A., Barboza-Palomino, M., Reyes-Bossio, M., Peña-Calero, B.N., White, M.	Validation of the De Jong Gierveld Loneliness Scale in Peruvian old adults: a study based on SEM and IRT multidimensional models
Custodio, N., Montesinos, R., Chambergo-Michilot, D., Herrera-Perez, E., Pintado-Caipa, M., Seminario G, W., Cuenca, J., Mesía, L., Failoc-Rojas, V.E., Diaz, M.M.	A Functional Assessment Tool to Distinguish Controls From Alzheimer's Disease in Lima, Peru
Montesinos, R. and Parodi, J.F. and Diaz, M.M. and Herrera-Perez, E. and Valeriano-Lorenzo, E. and Soto, A. and Delgado, C. and Slachevsky, A. and Custodio, N.	Validation of Picture Free and Cued Selective Reminding Test for Illiteracy in Lima, Peru
Caycho-Rodríguez, T., Tomás, J.M., Barboza-Palomino, M., Ventura-León, J., Gallegos, M., Reyes-Bossio, M., Vilca, L.W.	Assessment of Fear of COVID-19 in Older Adults: Validation of the Fear of COVID-19 Scale
Caycho-Rodríguez, T., Tomás, J.M., Ventura-León, J., Sancho, P., Cabrera-Orosco, I., Barboza-Palomino, M.	Measurement of the will to live in older adults: Transcultural adaptation, validity and reliability of the Will-to-Live Scale
Caycho-Rodríguez, T., Tomás, J.M., Ventura-León, J., Carranza Esteban, R.F., Oblitas Guadalupe, L.A., Reyes-Bossio, M., García Cadena, C.H., Cabrera-Orosco, I.	Factorial validity and invariance analysis of the five items version of Mindful Awareness Attention Scale in older adults
Queirolo Ore, S.A., Barboza-Palomino, M., Ventura-León, J.	Measuring the quality of life in institutionalized seniors in Lima (Peru)
Tomas, J.M., Caycho-Rodríguez, T., Ventura-León, J., Sancho, P., García, C.H., Arias, W.L.	Measurement Invariance of the Brief Resilient Coping Scale (BRCS) in Peruvian and Spanish Older Adults

Adrián, José A, Bermúdez-Llusá, Geidy, Caramés, José M, Rodríguez-Parra, María J, Arango-Lasprilla, Juan C.	The NeuroBel: A Screening Test for Verbal Language Impairment in Spanish-Speaking Elderly People With Cognitive Decline.
Árraga Barrios, Marisela Virginia, & Sánchez Villarroel, Marhilde	Validity and reliability of the Scale of Happiness from Lima in Venezuelan elderly people
Ferreira-Correia, A, Campagna, I.	ASSESSMENT OF COGNITIVE IMPAIRMENT IN LOW-RESOURCES CONTEXTS (THE VENEZUELAN EXAMPLE): ARE THE MINI-MENTAL STATUS EXAMINATION AND THE CLOCK DRAWING TEST USEFUL?

Year	Country	City	Name of tool
2015	Argentina	Cordova	Mattis Dementia Rating Scale (MDRS)
2016	Argentina	Rio de la plata	Questionnaire Alzheimer's Disease 8 (AD8)
2015	Argentina	Cordova	Boston Naming Test (BNT)
2013	Argentina	Santa Fe	Test Your Memory
2011	Argentina	buenos aires	spanish revised memory and behaviour symptoms checklist (SRMBPC)
2016	Argentina	Buenos aires	Memory Binding Test (MBT)
2018	Argentina	Buenos aires	Everyday Cognition (ECog) scale - Spanish
2014	Argentina	Buenos aires	Photo-test (PT)
2016	Argentina	Rancul	Community screening instrument for dementia (CSID)

2017	Argentina	Mar del Plata	Addenbrooke's Cognitive Examination (ACE)
2020	Argentina	Buenos aires	Combined Screening Interpretation Score
2023	Argentina	N/I	Minilinguistic State Examination (MLSE)
2022	Argentina	Rosario	Montreal Cognitive Assessment (MoCA)
2021	Argentina	N/I	Cognitive Complaints Questionnaire (CCQ) and Addenbrooke's Cognitive Examination-III (ACE-III)
2022	Argentina	Rosario	cuestionario de reserva cognitiva (CRC)
2020	Argentina	Buenos aires	Montreal Cognitive Assessment (MoCA)
2021	Argentina	Buenos aires	Yesavage’s Geriatric Depression Questionnaire (GDS-VE)
2022	Argentina	Mar del Plata	Brief-COPE
2021	Argentina	N/I	Cognitive Reserve Questionnaire

2020	LA	N/I	Addenbrooke's Cognitive Examination III (ACE-III)
2018	Brazil	Sao Paulo	Targeted Geriatric Assessment (TaGA)
2017	Brazil	Sao Paulo	Geriatric PsychoSocial Assessment of Pain-induced Depression (GEAP) scale.
2010	Brazil	Sao Paulo	Disability Assessment for Dementia Brazilian version(DAD-Br).
2007	Brazil	Juiz de For a	Center of Epidemiological Studies – Depression (CES-D)
2013	Brazil	Sao Paulo	Emotional Regulation Questionnaire (ERQ)
2001	Brazil	Sao Paulo	Consortium to Establish a Registry for Alzheimer´s Disease (CERAD)
2016	Brazil	Sao Paulo	Pain Assessment Checklist for Seniors (PACSLAC)
1991	Brazil	Sao Paulo	Mental Status Questionnaire (MSQ)
2017	Brazil	Curitiba	Australian National University - Alzheimer’s Disease Risk Index (ANU-ADRI)

2004	Brazil	Pernambuco	Mini-Mental State Examination (MMSE)
2005	Brazil	Pernambuco	Mini-Mental State Examination (MMSE)
2012	Brazil	Santa Cruz	Leganes cognitive test (LCT)
2011	Brazil	Porto Alegre	Brazilian telephone version of the Mini Mental State Examination (Braztel-MMSE)
2011	Brazil	Sao Paulo	Behavioral Assessment of the Dysexecutive Syndrome (BADS)
2010	Brazil	Belo Horizonte	Addenbrooke Cognitive Examination-revised (ACE-R)
2007	Brazil	N/I	Addenbrooke Cognitive Examination-revised (ACE-R)
2016	Brazil	Itajubá, Piranguinho, Pouso Alegre, and Santa Rita do Sapucaí in the state of Minas	Vitor Quality of Life Scale for the Elderly (VITOR QLSE).
2015	Brazil	22 states of Brazil	Meaning in Life Questionnaire (MLQ)
2015	Brazil	Sao Paulo	Cognitive Change Questionnaire (brief version CQQ-8 and CQQ-22)

2017	Brazil	Belo Horizonte, State of Minas Gerais	Vulnerability to Abuse Screening Scale (VASS)
2018	Brazil	N/I	Rowland Universal Dementia Assessment Scale
2019	Brazil	Porto Alegre	Relationship Scales Questionnaire (RSQ)
2007	Brazil	Porto Alegre	Clinical Dementia Rating (CDR)
2012	Brazil	Sao Paulo	Dementia Rating Scale (DRS)
2015	Brazil	Sao Carlos	California Older Person's Pleasant Events Schedule (COPPES)- OPPES-BR (Brazilian version)
2019	Brazil	Sao Paulo	World Health Disability Assessment Schedule (WHODAS 2.0)
2009	Brazil	Sao Paulo	Short Cognitive Performance Test (SKT)
2006	Brazil	Sao Paulo	Short Cognitive Performance Test (Syndrom-Kurztest, SKT)
2006	Brazil	Porto Alegre	World Health Organization Quality of Life for Older Persons (WHOQOL-OLD)

2016	Brazil	Sao Paulo	10-point Cognitive Screener (10-CS)
2003	Brazil	Bambuı	Clock drawing test (CDT)
2017	Brazil	Sao Carlos, Sao Paulo	California Older Person Pleasant Events Schedule – OPPES-BR (Brazilian version)
2001	Brazil	Sao Paulo	Michigan Alcoholism Screening Test (MAST)
2012	Brazil	Sao Paulo	Dementia Rating Scale
2014	Brazil	Sao Paulo	Functional Assessment Questionnaire/FAQ, Category Verbal Fluency/CVF, Clock Drawing Test /CDT.
2014	Brazil	Sao Paulo	Category Fluency Test (CFT) and the Functional Activities Questionnaire (FAQ)
2014	Brazil	Sao Carlos, Sao Paulo	Michigan Alcoholism Screening Test – Geriatric Version (MAST-G)
2016	Brazil	Sao Paulo	Boston Naming Test (BNT) versions (original, adapted, 30 item even, 30 item odd and CERAD)
2016	Brazil	N/I	Baptista Depression Scale - Senior Version (EBADEP-ID)

2010	Brazil	N/I	Cambridge Examination for Mental Disorders of the Elderly-Revised Version (CAMDEX-R)
2014	Brazil	ReICfe	CASP-19
2011	Brazil	N/I	Pain Assessment Checklist for Seniors with Limited Ability to Communicate (PACSLAC)
2008	Brazil	Rio de Janeiro	Clock drawing test (CDT)
2014	Brazil	Rio de Janeiro	Questionnaire on Cognitive Decline in the Elderly (IQCODE-BR)
2006	Brazil	Rio de Janeiro	Mini-Mental State Examination (MMSE); the Montreal Cognitive Assessment (MoCA); Leganés Cognitive Test (LCT).
2007	Brazil	Florianópolis	Perceived Stress Scale (PSS)
1998	Brazil	Sao Paulo	Mini-Mental State Examination (MMSE)
1999	Brazil	Sao Paulo	Geriatric Depression Scale (GDS)
1999	Brazil	Sao Paulo	Geriatric Depression Scale (GDS)

2012	Brazil	Dourados	Geriatric Depression Scale (GDS)
2013	Brazil	Sao Paulo	Premorbid Cognitive Abilities Scale (PCAS)
2018	Brazil	Sao Paulo	Montreal Cognitive Assessment (MoCA)
2011	Brazil	Sao Paulo	Cambridge Cognitive Examination (CAMCOG)
2010	Brazil	Sao Paulo	Clock Drawing Test (CDT)
2008	Brazil	Juiz de Fora	Tuokko's Clock Test
2019	Brazil	Tapejara-RS	WHOQOL-Bref web and WHOQOL-Old web
2019	Brazil	Sao Paulo	Iowa Gambling Task (IGT)
2010	Brazil	Fortaleza	Geriatric Depression Scale (GDS)
2014	Brazil	Sao Carlos	36-item survey health status (SF-36) and the World Health Organization quality of life assessment instrument (WHOQOL-BREF)

2014	Brazil	Bambui	Mini-Mental State Examination (MMSE)
2009	Brazil	Bambui	Mini-Mental State Examination (MMSE)
2008	Brazil	Bambui	Mini-Mental State Examination (MMSE)
2016	Brazil	Jundiaí	Pentagon Drawing Test (PDT)
2012	Brazil	Jundiaí	Clock Drawing Test (CDT)
2016	Brazil	Jundiaí	Montreal Cognitive Assessment (MoCA)
2014	Brazil	Sao Paulo	Montreal Cognitive Assessment (MoCA)
2010	Brazil	N/I	Geriatric Depression Scale-15 (GDS-15)
2008	Brazil	Southern Brazil	WHOQOL-OLD
2007	Brazil	N/I	World Health Organization's Quality of Life Instrument–Short Version (WHOQOL-BREF)

2016	Brazil	Sao Paulo	The Canadian occupational performance measure (COPM)
2017	Brazil	Minas de Gerai	Alzheimer’s Disease Cooperative Study – Activities of Daily Living (ADCS-ADL)
2011	Brazil	Recife	AD8-Brazil
2006	Brazil	Porto Alegre	Montgomery-Asberg rating scale
2012	Brazil	Campinas	Prospective Memory (ProM) tests
2014	Brazil	Porto Alegre	Geriatric Anxiety Inventory (GAI)
2012	Brazil	Sao Paulo	Montreal Cognitive Assessment (MoCA)
2014	Brazil	Sao Paulo	Computer-Administered Neuropsychological Screen for Mild Cognitive Impairment (CANS-MCI)
2018	Brazil	Sao Paulo	Mini-Addenbrooke’s Cognitive Examination (M-ACE)
2005	Brazil	Sao Paulo	Clinical Dementia Rating (CDR)

2015	Brazil	Bahía	Patient Health Questionnaire-9 (PHQ) and the 15-item Geriatric Depression Scale (GDS-15)
2006	Brazil	Minas de Gerais	Geriatric Depression Scale (GDS-30)
2008	Brazil	Belo Horizonte region metropolitana	Three Words-Three Shapes Test (3P3F)
2010	Brazil	Sao Paulo	QOL-AD Brazil (tested 3 versions in common use)
2014	Brazil	Rio de janerio	Br-CAMCOG-R
2009	Brazil	Rio de janerio	Br-CAMCOG-R
2005	Brazil	Rio de janerio	GDS-15, Brazilian Portuguese version (Almeida 1999)
2008	Brazil	Sao Paulo	Elderly Quality of Life Index – EqoLI
2014	Brazil	Belo Horizonte	General Activities of Daily Living Scale /(DLS)
2012	Brazil	Belo Horizonte	Rey-Auditory Verbal Learning Test

2008	Brazil	Sao Paulo	Short IQ Code
2009	Brazil	Sao Paulo	Brazilian versions of the IQCODE L, S and a new short version (SBr) (15 items)
2010	Brazil	Sao Paulo	GDS-15, Brazilian Portuguese version
2018	Brazil	ReICfe	MOCA-Brazil
2009	Brazil	Rio de janerio	mini-cog
2019	Brazil	Apuí, (Fonte Boa) and Manaus (Amazonas),	Cognitive Telephone Screening Instrument (COGTEL)
2009	Brazil	Sao Paulo	MMSE (brazilian version)
2009	Brazil	Rio de janerio	Informant Questionnaire on Cognitive Decline in the Elderly Brazil version (IQCODE-BR)
2013	Brazil	Rio de janerio	Informant Questionnaire on Cognitive Decline in the Elderly Brazil version (IQCODE-BR)
2009	Brazil	Sao Paulo	Self-reporting questionnaire-SRQ 20

2001	Brazil	Sao Paulo	Cognitive subscale of the "Alzheimer's Disease Assessment Scale" (ADAS-Cog)
2014	Brazil	Belo Horizonte	World Health Organization Quality of Life-Bref (WHOQOL-bref)
2019	Brazil	Samambaia	WHOQOL-bref
2016	Brazil	Sao Paulo	Geriatric Anxiety Inventory (GAI) and GAI short form (GAI-SF)
2009	Brazil	natal, reICfe	STADP
2008	Brazil	Sao Paulo	CAMCOG
2009	Brazil	Rio de janerio	BR-CAMCOG-R Brazil version of CAMCOG
2012	Brazil	N/I	Montgomery-[Angstrom]sberg. Depression Rating Scale (MADRS) and Cornell Scale for Depression in Dementia (CSDD)
2009	Brazil	FIBRA network (7 ICties in south east Brazil	10-item Purpose in Life scale - Brazilian Adaptation
2015	Brazil	Ribeirao Preto	Autobiographic Episodic Memory Interview (EAMI)

2016	Brazil	Sao Paulo	Multifactorial Memory Questionnaire (MMQ)- Brazil version
2009	Brazil	Sao Paulo	Camberwell Assessment of Needs for the Elderly Scale (CANE)
2001	Brazil	Sao Paulo	ENEDAM
2010	Brazil	Sao Paulo	Rivermead Behavioural Memory Test (RBMT)
2018	Brazil	pouso alegre, santa rita do sapucai	49-item BRIEF-SRC spiritual/religious coping scale
2019	Brazil	Sao Paulo	Medical Outcomes Studysocial Support Survey (MOS-SSS)
2018	Brazil	Salvador, Bahia	Quality of life and swallowing questionnaire for individuals with Parkinson's disease
2007	Brazil	Sao Paulo	Minnesota Living with Heart Failure(LHFQ)
2019	Brazil	Sao Paulo	Multidimensional Assessment of Older People in Primary Care (AMPI-AB)
2009	Brazil	Rio de janerio	Caregiver Abuse Screen (CASE)

2015	Brazil	Rio de janerio	Informant Questionnaire on Cognitive Decline in the Elderly (IQCODE)
2018	Brazil	Country-wide 35% central west, 34% north-northeast, 31% south-southeast	Barrow Neurological Institute (BNI) Screen for Higher Cerebral Functions (BNIS)
2018	Brazil	Sao Paulo	Proposed care need classification
2019	Brazil	minais gerias, curitiba, sao paulo	Baptista's Depression Scale-older version (EBADEP-ID).
2007	Brazil	Sao Paulo	Bayer-Activities of Daily Living scale (B-ADL)
2018	Brazil	Jundiai	Montreal Cognitive Assessment (MoCA)
2014	Brazil	Sao Paulo	Visual Analogue Scale of Happiness and the Cornell Scale for Depression in Dementia
2018	Brazil	Trembembe	Montreal Cognitive Assessment (MoCA)
2011	Brazil	Belo Horizonte	Nottingham Health Profile (NHP) and the Short Form-36 (SF-36)
2019	Brazil	ReICfe	Montreal Cognitive Assessment (MoCA)

2010	Brazil	Belo Horizonte	Pain Locus of control scale
2015	Brazil	Sao Paulo	Multifactorial Memory Questionnaire (MMQ)
2021	Brazil	Jundiaí	Cambridge Cognition Examination (CAMCOG)
2021	Brazil	São Luís and Fortaleza	Addenbrooke's Cognitive Examination Revised (ACE-R)
2023	Brazil	Sao Paulo	LIFE-H 3.1-Brazil
2020	Brazil	N/I	FTD Rating Scale (FTD-FRS)
2022	Brazil	Minas Gerais	UCLA
2020	Brazil	N/I	The pFCSRT-IR (picture Free and Cued Selective Reminding with Immediate Recall)
2020	Brazil	Río de Janeiro	Geriatric Depression-15 Scale (GDS-15)
2020	Brazil	N/I	Bender Gestalt Test (BGT)

2021	Brazil	Pernambuco State	Kessler Psychological Distress Scale
2020	Brazil	Río de Janeiro	semantic fluency test
2020	Brazil	Londrina	Modified Fatigue Impact Scale (MFIS-PD/BR).
2022	Brazil	Sao Paulo	Cognitive Function Instrument
2023	Brazil	N/I	“Older Adult Lifestyle Scale” (OALS)
2023	Brazil	Belo Horizonte	Multidimensional Individual and Interpersonal Resilience Measure (MIIRM).
2023	Brazil	Paraíba and Pernambuco	Hwalek-Sengstock Elder Abuse Screening Test (H-S/EAST), Conflict Tactics Scale (CTS-1)
2023	Brazil	Sao Paulo	Pain Intensity Measure for Persons with Dementia (PIMD)
2022	Brazil	Belo horizonte and Sao Paulo	Addenbrooke’s Cognitive Examination Revised (ACE-R)
2021	Brazil	Rio Grande do Sul and in the Southwest of Santa Catarina	Dysexecutive Questionnaire (DEX)

2021	Brazil	Maringá, Paraná	Leisure Attitude Measurement
2021	Brazil	Vitória de Santo Antão, Pernambuco	Kessler Psychological Distress Scale
2020	Brazil	Paraíba and Pernambuco	Prefrontal Symptoms Inventory (PSI) (abbreviated version)
2020	Brazil	N/I	Mini-Mental State Examination (MMSE)
2013	Chile	Santiago	Delirium predictive score (DPS)
2011	Chile	Santiago	WHOQOL-BREF (World Health Organization Quality of Life, brief version)
2001	Chile		Syndrom Kurztest Test (SKT)
2007	Chile	Arica and Parinacota	Successful Aging Inventory (SAI)
2018	Chile	Arica and Parinacota	Brief Index of Religiousness and Spirituality
2018	Chile	Arica and Parinacota	Questionnaire of Communitysocial Support

2000	Chile	Santiago	Geriatric Depression Scale 5-item (GDS 5-item)
2007	Chile	Viña del Mar	Eurotest ("Test de los Pesos")
2017	Chile	N/I	Mini-Mental State Examination included in the Chilean Functional assessment of elderly people (MM-SE-EFAM) Short version
2013	Chile	Santiago	Short-form-36 health survey (SF-36)
2019	Chile	Metropolitan and Valparaiso	Geriatric Anxiety Inventory (GAI)
2009	Chile	Maule, Chile	MUNSCH (Scale of Happiness of the Memorial University of NewFoundland
2014	Chile	santiago metropolitan region	Test Your Memory-Spanish version (TYM-S)
2012	Chile	santiago metropolitan region	ACE-R
2014	Chile	Chillan, Bio Bio	Zimet multidimensional perceived social support scale
2004	Chile	ConcepICon and VIII region	MMSE and PFAQ

2013	Chile	antofagasta	WHOQoL-Old (abbreviated)
2021	Chile	Regions of Arica and Parinacota and La Araucanía of Chile.	Perceived social Support Questionnaire (PSSQ)
2021	Chile	N/I	The Frontal Assessment Battery (FAB)
2020	Chile	Iquique (north), Valparaíso (center), and Concepción (south).	Montreal Cognitive Assessment (MoCA)
2020	Chile	north and south of Chile	Brief Resilient Coping Scale (BRCS)
2023	Chile	Antofagasta	Dr. Reed's Spiritual Perspective Scale
2022	Chile	Concepción y Arauco	Aged adults adaptation scale to their residencea (EAPAR-Chile)
2020	Chile	Temuco	Mini Mental State Examination (MMSE), the Montreal Cognitive Assessment (MoCA) and the Adenbrookke´s Cognitive Examination (ACE-R)
2022	Chile	Los Lagos region	Phototest (PT)
2020	Chile	Concepción and Talcahuano	Patient Health Questionnaire (PHQ-9)

2023	Chile	N/I	Rowland Universal Dementia Assessment Scale (RUDAS)
2023	Chile	North, center and south of Chile. Iquique, Valparaíso, and ConcepICón,	The Free and Cued Selective Reminding Test (FCSRT)
2021	Chile	ConcepICón	Multidimensional Scale of Perceived social Support (MSPSS)
2021	Chile	All Chilean regions	Short form health survey questionnaire (SF-12)
2021	Chile	N/I	De Jong Gierveld Loneliness Scale, 6-item version (DJGLS-6)
2021	Chile	N/I	Addenbrooke's Cognitive Examination III
2021	Chile	Región Metropolitana	Scale of Losses Experienced in Old Age (SLO).
2020	Chile	north and south of Chile	15-item Geriatric Depression Scale
2020	Chile	N/I	Addenbrooke's Cognitive Examination III
2023	Chile	ConcepICón	Quality of Life in Alzheimer’s Disease

2017	LA	Maule, Chile; Guayas, Ecuador	satisfaction with life scale (SWLS)
2004	Colombia	Cali	Scale of Depression of Yesavage (reduced versión GDS-15)
2011	Colombia	Pasto and Bogotá	Brief Assessment Scale for Depression (BAS-DEP) and the Even Briefer Assessment Scale for Depression (EBAS-DEP)
2008	Colombia	Cartagena	Yesavage Geriatric Depression Scale (GDS-15)
2016	Colombia	N/I	Pesotest
2010	Colombia	Bello	ESTE
2015	Colombia	Bogota	Montreal Cognitive Assessment Spanish version (MoCA-S)
2012	Colombia	Manizales	Spanish version of the Montreal Cognitive Assessment (MoCA-S)
2011	Colombia	Cartagena	Geriatric Depression Scale (GDS-5 and GDS-15),
2013	Colombia	Bucaramanga	ESTE-R

2015	Colombia	Cartagena	Attitudes towards Sexuality in the Elderly Questionnaire (ASEQ)
2017	Colombia	Antioquia	MMSE (MEEM)
2018	Colombia	Bucaramanga, Manizales	WHOQOL-BREF
2013	Colombia	Bogotá, Cali, Medellín, Barranquilla, Bucaramanga, Pereira, Cartagena,	Life Orientation Test (LOT-R)
2021	Colombia	N/I	Family Abuse Screening Questionnaire
2024	Colombia	Huila: 64.6% Caquetá: 35.4%	Addenbrooke's Cognitive Examination Revised (ACE-R)
2023	Colombia	Medellín	Anosognosia Questionnaire in Dementia (AQ-D)
2020	Colombia	Medellín	Delirium diagnostic tool-provisional (DDT-Pro)
2022	Colombia	Cali	Parkinson's Disease Cognitive Rating Scale (PD-CRS)
2023	Colombia	N/I	Memory Complaint Scale (MCS-15)

2020	Colombia	Medellín	Delirium diagnostic tool-provisional (DDT-Pro)
2018	Costa Rica	San Jose and Guanacaste	Health-related quality of life (Short-Form-36 [SF-36])
2024	Costa Rica	N/I	The Wagnild and Young Resilience Scale
2017	Cuba	Villa Clara	Cuban Version of Addenbrooke's Cognitive Examination-Revised
2021	Cuba	Santa Clara	Frontal Assessment Battery (FAB) and INECO Frontal Screening (IFS)
2021	Cuba	La Habana	Brian Health Assessment (BHA)
2004	Ecuador	Quito	Parkinson’s Disease Quality of Life Questionnaire Ecuador version (PDQL-EV)
2020	Ecuador	Quito	Geriatric Depression-15 Scale (GDS-15)
2015	LA	N/I	Euro-D depression scale
2019	LA	N/I	Healthy Ageing Index

2015	LA	N/I	EURO-D
2019	LA	N/I	DEMQOL and DEMQOL-Proxy.
2009	LA	Brasilia, quito, AsunICon. Buenos Aires	Scales for Outcomes in Parkinson's Disease-PsychoSocial Questionnaire (SCOPA-PS)
2010	LA	Brasilia, quito, AsunICon. Buenos Aires	Modified Parkinson Psychosis Rating Scale (mPPRS)
2010	LA	Not stated, as per 10/66	World Health Organization Disability Assessment Schedule (WHODAS II)
2009	LA	Not stated, as per 10/67	Community Screening Instrument for Dementia (CSI 'D')
2004	LA	Guadalajara, mexico city, sao paulo, botucatu, sao jose, la habana. Others not	Geriatric Mental State (GMS) (dementia) including EURO-D (depression)
2021	LA	N/I	Yesavage
2013	Mexico	Sonora	WHOQOL-OLD
2007	Mexico	N/I	MHAS Questionnaire

2017	Mexico	Mexico city	Montreal Cognitive Assessment (MoCA/MoCA-E)
2018	Mexico	N/I	Clock Drawing Test (CDT)
2003	Mexico	Mexico city	Health Perceptions Questionnaire (HPQ)
2012	Mexico	N/I	Spirituality Index
2012	Mexico	Mexico city	Geriatric Mistreatment Scale (GMS)
2019	Mexico	Coahuila	Despair scale for older adults
2015	Mexico	Toluca	WHOQOL-OLD
2013	Mexico	Monterrey	Life Satisfaction Index short form (LSITA-SF)
2014	Mexico	Mexico city	Relevant Outcome Scale for Alzheimer ́s disease (ROSA)
2004	Mexico	Mexico city	Minimental State Examination, Brief Neuropsychological Test Battery, Short Blessed test, Pfeffer Functional Activities Questionnaire, and Blessed

2015	Mexico	N/I	Cross Cultural Cognitive Examination (CCCE)
2014	Mexico	Morelos y Tlaxcala	CES-D 7 (brief)
2014	Mexico	Mexico city	Euro-Quol 5
2008	Mexico	Mexico city	GDS, CES-DR
2007	Mexico	Mexico city	Anxiety about Aging Scale - Mexico adaptation
2010	Mexico	Mexico city	Quality of Life in Alzheimer's Disease(QOL-AD)
2007	Mexico	N/I	Scale of Positive and Negative Patterns of ReligiousCoping Methods (Brief-RCOPE+, Brief-RCOPE-), IMSOL confronting loneliness subscale (ARS),
2003	Mexico	Mexico city	Epidemiologic Studies Depresion Scale (CES-D)
2020	Mexico	Mexico city	Quality of Life Scale
2022	Mexico	N/I	The neuropsychological battery

2022	Mexico	Chihuahua and Monterrey	SF-36 Health Questionnaire
2023	Mexico	ICudad de México	Five-Word Test
2023	Mexico	n/I	everyday cognition scale (M-ECog)
2023	Mexico	Mexico city and Xalapa, Veracruz.	De Jong Gierveld Loneliness Scale (DJGLS)
2021	Mexico	Sonora	Geriatric Depression-15 Scale (GDS-15)
2024	Mexico	Mexico city and Xalapa, Veracruz	Short Form 36 (SF-36) Health Survey
2021	Mexico	Chihuahua	Lasher and Faulkender Anxiety
2020	Mexico	Saltillo, Coahuila	Massachusetts General Hospital-Sexual Functioning Questionnaire (MGH-SFQ)
2016	Peru	Tumbes	Mini-Mental State Examination (MMSE); the Montreal Cognitive Assessment (MoCA); Leganés Cognitive Test (LCT).
2019	Peru	Trujillo	5-item Coping with Humor Scale (CHS-5)

2018	Peru	Trujillo	Scale of Self-Efficacy for Aging (EAEE)
2018	Peru	Trujillo	Diener's Satisfaction with Life Scale (SWLS)
2018	Peru	Trujillo	Brief Resilient Coping Scale (BRCS)
2016	Peru	Lima	Clock Drawing Test Manos versión (PDR-M) and Mini Mental State Examination - Peruvian version (MMSE)
2011	Peru	Lima	Clock Drawing Test (PDR-M)
2017	Peru	Lima	Memory Alteration Test (M@T)
2019	Peru	Lima	Rowland Universal Dementia Assessment Scale (RUDAS-PE)
2017	Peru	Arequipa	MMSE (Modified version custodio-lira and robles-arana)
2019	Peru	Arequipa	Photo-test
2019	Peru	N/I	life satisfaction scale (SWLS)

2004	Peru	Lima	Clock Drawing Test
2016	Peru	Lima	Coin Test
2016	Peru	Lima	Clock drawing test- Manos versión (PDR-M) and Mini Mental State Examination - Peruvian version (MMSE)
2023	Peru	Lima	Memory Alteration Test (M@T)
2020	Peru	Lima	Rowland Universal Dementia Assessment Scale (RUDAS-PE)
2021	Peru	Lima or Collao	MMSE, SPMSQ, MoCA
2021	Peru	Lima	Mini-SEA, r-SMS, and IFS
2022	Peru	Lima	Neuropsychological evaluation test – Neuropsi
2022	Peru	Lima	Coronavirus Anxiety Scale (CAS)
2022	Peru	Lima	Peruvian version of Addenbrooke's Cognitive Examination (ACE-Pe), of INECO Frontal Screening (IFS-Pe) and of the Mini-Mental

2023	Peru	N/I	Impact on Quality of Life (COV19-QoL)
2023	Peru	Lima	Semantic Memory Assessment Battery for Older Adults (EMSEA)
2023	Peru	Trujillo	De Jong Gierveld Solitude Scale (DJGLS)
2022	Peru	Lima	Alzheimer's Disease Cooperative Study-Activities of Daily Living (ADCS-ADL)
2022	Peru	Callao	Free and Cued Selective Reminding Test-Picture version (FCSRT-Picture)
2022	Peru	Lima	Fear of COVID-19 Scale (FCV-19S)
2021	Peru	La libertad	Will-to-Live Scale (WTLS)
2021	Peru	San Martín	Mindful Awareness Attention Scale (MMAS-5)
2020	Peru	Lima	World Health Organization Quality of Life-Old (WHOQOL-OLD)
2021	Peru and Spain	Trujillo (Peru) and ValenICa (Spain)	Brief Resilient Coping Scale (BRCS)

2023	Spain, Cuba, and Colombia	N/I	NeuroBel
2011	Venezuela	N/I	Revised version of the Scale of Happiness from Lima for elderly people
2022	Venezuela	Caracas	MMSE and The clock drawing test

Setting	Key words 2	Number of criteria 2	study tipe
N/I	Diagnostic accuracy	1	Cross-sectional
N/I	reliability, diagnostic accuracy, Construct and/or Criterion Validity, translation, adaptation	5	Cross-sectional
Urban	Diagnostic accuracy, Construct and/or Criterion Validity,	2	Cross-sectional
Urban	Reliability, Construct and/or Criterion Validity, diagnostic accuracy	3	Cross-sectional
Urban	translation, adaptation, reliability, Construct and/or Criterion Validity, divergent validity, factor analysis	6	Cross-sectional
Urban	Diagnostic accuracy, Construct and/or Criterion Validity	2	Cross-sectional
Urban	Reliability, diagnostic accuracy, Construct and/or Criterion Validity	3	Cross-sectional
Urban	diagnostic accuracy, Construct and/or Criterion Validity	2	Cross-sectional
Rural	Reliability, diagnostic accuracy, Construct and/or Criterion Validity,	3	Cross-sectional

N/I	Reliability, diagnostic accuracy, Construct and/or Criterion Validity	3	Cross-sectional
N/I	Diagnostic Accuracy	1	Cross-sectional
N/I	Translation, adaptation	2	Cross-sectional
Urban	Normative data	1	cross-sectional
N/I	Reliability, adaptation	2	Cross-sectional
N/I	Reliability, Construct and/or Criterion Validity, rash model	3	Cross-sectional
Urban	Reliability, diagnostic accuracy, Construct and/or Criterion Validity	3	Cross-sectional
N/I	Reliability, Factor analysis	2	Cross-sectional
N/I	Reliability, factor analysis	2	Cross-sectional
N/I	Reliability, Construct and/or Criterion Validity, factor analysis	3	Cross-sectional

N/I	Reliability, Diagnostic Accuracy	2	Cross-sectional
N/I	Reliability, diagnostic accuracy, Construct and/or Criterion Validity, development	4	Cross-sectional
N/I	Reliability, Construct and/or Criterion Validity and translation, adaptation	4	Cross-sectional
N/I	Realiability, Construct and/or Criterion Validity, diagnostic accuracy	3	Cross-sectional
Urban	Reliability, diagnostic accuracy, factor analysis	3	Cross-sectional
Urban	Reliability, factor analysis	2	Cross-sectional
N/I	Diagnostic accuracy, translation, adaptation	3	Cross-sectional
Urban	Reliability, Construct and/or Criterion Validity	2	Cross-sectional
Urban	Diagnostic accuracy	1	Cross-sectional
N/I	translation, adaptation, reliability	3	Longitudinal

N/I	Reliability, adaptation	2	Cross-sectional
N/I	Contruct validity, adaptation	1	Cross-sectional
Urban	Reliability, Construct and/or Criterion Validity, translation, adaptation	4	Longitudinal
Urban	Reliability, diagnostic accuracy, Construct and/or Criterion Validity	3	Cross-sectional
N/I	Diagnostic accuracy, Construct and/or Criterion Validity	2	Cross-sectional
N/I	Diagnostic accuracy	1	Cross-sectional
N/I	Translation, adaptation	2	Cross-sectional
Urban	Reliability, Construct and/or Criterion Validity, divergent validity, factor analysis	4	Cross-sectional
N/I	Factor analysis	1	Cross-sectional
N/I	Reliability, diagnostic accuracy, Construct and/or Criterion Validity	3	Cross-sectional

Urban	Reliability, factor analysis and adaptation	3	Cross-sectional
N/I	Diagnostic accuracy	1	Cross-sectional
N/I	Translation, adaptation, reliability	3	Validation
Urban	Reliability, diagnostic accuray, Construct and/or Criterion Validity	3	Cross-sectional
Urban	Diagnostic accuracy, Construct and/or Criterion Validity	2	Cross-sectional
Urban	Relibility, Factor analysis	2	Cross-sectional
Urban	Normative data	1	Cross-sectional
Urban	Diagnostic accuracy	1	Cross-sectional
Urban	Reliability, diagnostic accuracy, Construct and/or Criterion Validity.	3	Cross-sectional
Urban	Reliability, factor analysis, development	3	Cross-sectional

Urban	Diagnostic accuracy	1	Prospective cohort study.
N/I	Reliability	1	Cross-sectional
Urban	Factor analysis	1	Cross-sectional
Urban	Diagnostic accuracy, Construct and/or Criterion Validity	2	Cross-sectional
Urban	Diagnostic accuracy, Construct and/or Criterion Validity	2	Cross-sectional
N/I	Diagnostic accuracy	1	Cross-sectional
N/I	Diagnostic accuracy	1	Cross-sectional
Urban	Reliability, diagnostic accuracy, translation, adaptation	4	Cross-sectional
N/I	Reliability, translation, normative data	3	Cross-sectional
N/I	Reliability, Construct and/or Criterion Validity, divergent validity	3	Cross-sectional

N/I	Reliability, translation, adaptation	3	Cross-sectional
N/I	Reliability, factor analysis, translation, adaptation	4	Cross-sectional
N/I	Translation and adaptation	2	Cross-sectional
N/I	reliability, diagnostic accuracy	2	Cross-sectional
Urban	Diagnostic accuracy,Construct and/or Criterion Validity	2	Cross-sectional
Urban	Diagnostic accuracy	1	Cross-sectional
N/I	Reliability, factor analysis, translation	3	Cross-sectional
N/I	Diagnostic accuracy	1	Cross-sectional
N/I	Reliability	1	Cross-sectional
N/I	Reliability, diagnostic accuracy, Construct and/or Criterion Validity	3	Cross-sectional

Urban	Factor analysis	1	Cross-sectional
N/I	Reliability and Construct and/or Criterion Validity	2	Cross-sectional
N/I	Normative data, diagnostic accuracy	2	Cross-sectional
N/I	Diagnostic accuracy	1	Cross-sectional
N/I	Diagnostic accuracy, Construct and/or Criterion Validity	2	Cross-sectional
N/I	Construct and/or Criterion Validity, divergent validity, translation, adaptation	4	Cross-sectional
N/I	Reliability	1	Cross-sectional
N/I	Construct and/or Criterion Validity	1	Cross-sectional
N/I	Diagnostic accuracy, Construct and/or Criterion Validity	2	Cross-sectional
N/I	Reliability and Construct and/or Criterion Validity	2	Cross-sectional

N/I	Factor analysis	1	Longitudinal
N/I	Factor analysis	1	Cross-sectional
N/I	Normative data	1	Cross-sectional
N/I	Diagnostic accuracy	1	Cross-sectional
N/I	Construct and/or Criterion Validity, divergent validity	2	Cross-sectional
N/I	Diagnostic accuracy	1	Cross-sectional
N/I	Diagnostic accuracy, Construct and/or Criterion Validity, divergent validity	3	Cross-sectional
N/I	Rasch analysis	1	Cross-sectional
N/I	Rasch analysis	1	Cross-sectional
N/I	Construct and/or Criterion Validity, reliability	2	Cross-sectional

N/I	Reliability, Construct and/or Criterion Validity	2	Cross-sectional
N/I	Reliability, diagnostic accuracy, translation, adaptation	4	Cross-sectional
N/I	Reliability, Construct and/or Criterion Validity, diagnostic accuracy	3	Cross-sectional
N/I	Reliability, Construct and/or Criterion Validity	2	Cross-sectional
N/I	Diagnostic accuracy	1	Cross-sectional
N/I	Reliability, Construct and/or Criterion Validity, diagnostic accuracy	3	Cross-sectional
N/I	Reliability, Construct and/or Criterion Validity, diagnostic accuracy	3	Cross-sectional
N/I	Reliability, diagnostic accuracy, Construct and/or Criterion Validity, factor analysis, translation, adaptation	6	Cross-sectional
N/I	Realiability, diagnostic accuracy, Construct and/or Criterion Validity	3	Cross-sectional
N/I	Diagnostic accuracy	1	Longitudinal study

N/I	Reliability, diagnostic accuracy, Construct and/or Criterion Validity	3	Cross-sectional
N/I	reliability, Diagnostic accuracy	2	Longitudinal study
Urban	normative data, translation, adaptation, diagnostic accuracy, Construct and/or Criterion Validity	5	Cross-sectional
Urban	reliability, Construct and/or Criterion Validity	2	Cross-sectional
Urban	diagnostic accuracy, Construct and/or Criterion Validity	2	Cross-sectional
Urban	reliability	1	Cross-sectional
Urban	Construct and/or Criterion Validity, diagnostic accuracy	2	Cross-sectional
Urban	development	1	Cross-sectional
Urban	Reliability, diagnostic accuracy, development, Construct and/or Criterion Validity, factor analysis	5	Cross-sectional
Urban	reliability, Construct and/or Criterion Validity, factor analysis	3	Cross-sectional

Urban	diagnostic accuracy, Construct and/or Criterion Validity	2	Cross-sectional
Urban	diagnostic accuracy	1	Cross-sectional
Urban	diagnostic accuracy, Construct and/or Criterion Validity, reliability, factor analysis	4	Cross-sectional
Urban	Normative data	1	Cross-sectional
Urban	diagnostic accuracy, Construct and/or Criterion Validity	2	Cross-sectional
Urban	reliability, translation, Construct and/or Criterion Validity	3	Cross-sectional
Urban	diagnostic accuracy, Construct and/or Criterion Validity	2	Cross-sectional
Urban	Reliability, translation, adaptation	3	Cross-sectional
Urban	diagnostic accuracy, Construct and/or Criterion Validity	2	Cross-sectional
Urban	diagnostic accuracy, construct and/or criterion validity	2	Cross-sectional

Urban	translation, adaptation	2	Cross-sectional
Urban	diagnostic accuracy	1	Cross-sectional
Urban	diagnostic accuracy	1	Cross-sectional
Urban	reliability, Construct and/or Criterion Validity, divergent validity	3	Cross-sectional
Urban	reliability, diagnostic accuracy, Construct and/or Criterion Validity, divergent validity	4	Cross-sectional
Urban	Construct and/or Criterion Validity, diagnostic accuracy	2	Cross-sectional
Urban	Cross-cultural adaptation	1	Cross-sectional
Urban	Reliability, diagnostic accuracy	2	Cross-sectional
Urban	adaptation, translation, reliability	3	Cross-sectional
Urban	Construct and/or Criterion Validity, adaptation, translation, reliabilty	4	Cross-sectional

Urban	adaptation, translation, reliability, Construct and/or Criterion Validity, divergent validity	5	Cross-sectional
Urban	reliability, Construct and/or Criterion Validity	2	Cross-sectional
Urban	reliability, translation	2	Cross-sectional
Urban	relaibility, diagnostic accuracy, Construct and/or Criterion Validity	3	Cross-sectional
Urban	reliability, factor analysis, Construct and/or Criterion Validity, divergent validity	4	Cross-sectional
Urban	factor analysis, reliability	2	Cross-sectional
Urban	development, factor analysis, reliability, divergent validity	4	Cross-sectional
Urban	Construct and/or Criterion Validity, divergent validity	2	Cross-sectional
Urban	reliability, Construct and/or Criterion Validity, diagnostic accuracy	3	longitudinal (md follow-up 16 months) consecutive sample
Urban	Reliability, Construct and/or Criterion Validity, factor analysis	3	Cross-sectional

Urban	factor analysis	1	Cross-sectional
Urban	normative data	1	Cross-sectional
Urban	reliability, development	2	Cross-sectional
Urban	factor analysis, diagnostic accuracy, reliability	3	Cross-sectional
N/I	Reliability, diagnostic accuracy	2	Cross-sectional
N/I	Dagnostic accuracy	1	Cross-sectional
Urban and rural	Construct and/or Criterion Validity	1	Cross-sectional
N/I	Diagnostic accuracy, normative data	2	Cross-sectional
N/I	Reliability	1	Cross-sectional
N/I	Reliability, diagnostic accuracy, Construct and/or Criterion Validity	3	Cross-sectional

N/I	Reliability, adaptation	2	Cross-sectional
N/I	Reliability, Construct and/or Criterion Validity, translation, divergent validity	4	Longitudinal
N/I	Diagnostic Accuracy, Construct and/or Criterion Validity	2	cross-sectional
N/I	Diagnostic Accuracy, Construct and/or Criterion Validity	2	cross-sectional
Urban	Reliability, Factor analysis	2	Cross-sectional
N/I	Reliability, Construct and/or Criterion Validity	2	Longitudinal
N/I	Reliability, Construct and/or Criterion Validity, fator analysis	3	Cross-sectional
N/I	Translation,adaptation, reliability	3	cross-sectional
N/I	normative data, Construct and/or Criterion Validity	2	Cross-sectional
N/I	Diagnostic accuracy, Construct and/or Criterion Validity	2	Cross-sectional

N/I	Reliability, diagnostic accuracy, Construct and/or Criterion Validity	3	Cross-sectional
N/I	Diagnostic accuracy, Construct and/or Criterion Validity	2	Cross-sectional
N/I	Reliability, diagnostic accuracy, Construct and/or Criterion Validity, divergent Validity	4	Cross-sectional
N/I	Reliability, diagnostic accuracy, Construct and/or Criterion Validity, divergent validity, translation, adaptation	6	Cross-sectional
N/I	Reliability, diagnostic accuracy, Construct and/or Criterion Validity, factor analysis, development	5	Cross-sectional
N/I	Reliability, factor analysis, translation, adaptation	4	Cross-sectional
N/I	Reliability	1	cross-sectional
N/I	Reliability, diagnostic accuracy, Construct and/or Criterion Validity	3	Cross-sectional
N/I	Diagnostic accuracy	1	Cross-sectional
N/I	Relibility, Construct and/or Criterion Validity, Factor analysis	3	Cross-sectional

N/I	Reliability, factor analysis	2	cross-sectional
N/I	Reliability, diagnostic accuracy, Construct and/or Criterion Validity	3	cross-sectional
N/I	Reliability, Construct and/or Criterion Validity, factor analysis, translation	4	Cross-sectional
Urban	Rash model	1	Cross-sectional
Urban	Development, diagnostic accuracy	2	Prospective cohort study
Urban	Reliability, factor analysis	2	Cross-sectional
Urban	Normative data, Construct and/or Criterion Validity	2	Cross-sectional
Urban and rural	Reliability, factor analysis	2	Cross-sectional
Urban and rural	Reliability, Construct and/or Criterion Validity, factor analysis, adaptation	4	Cross-sectional
Urban and rural	Reliability, Factor analysis	2	Cross-sectional

Urban	Reliability, diagnostic accuracy, Construct and/or Criterion Validity	3	Cross-sectional
N/I	Reliability, diagnostic accuracy, adaptation	3	Cross-sectional
N/I	Diagnostic accuracy	1	Cross-sectional
Urban	Reliability, factor analysis	2	Cross-sectional
Urban	Realiability, Construct and/or Criterion Validity, divergent validity, factor analysis	4	Cross-sectional
N/I	Construct and/or Criterion Validity, divergent validity, reliability, factor analysis, normative data	5	Cross-sectional
Urban	Reliability, Construct and/or Criterion Validity, divergent validity, adaptation	4	Cross-sectional
Urban	Construct and/or Criterion Validity, diagnostic accuracy, adaptation, translation, reliability	5	Cross-sectional
Urban	factor analysis, reliability	2	Cross-sectional
Urban and rural	Construct and/or Criterion Validity, diagnostic accuracy	2	Cross-sectional

N/I	factor analysis, reliability,	2	Cross-sectional
Rural	Reliability, Construct and/or Criterion Validity, Factor analysis	3	Cross-sectional
N/I	Reliability, diagnostic accuracy, Construct and/or Criterion Validity, factor analysis	4	cross-sectional
N/I	Reliability, diagnostic accuracy, Construct and/or Criterion Validity	3	Cross-sectional
Rural	Reliability, Construct and/or Criterion Validity, rasch model	3	Cross-sectional
N/I	Reliability, Factor analysis	2	Cross-sectional
Rural and Urban	Realiability, Construct and/or Criterion Validity, divergent validity	3	Cross-sectional
N/I	Reliability, Factor analysis	2	Cross-sectional
Rural	Diagnostic accuracy	1	Cross-sectional
N/I	Reliability, Diagnostic Accuracy, factor analysis, Construct and/or Criterion Validity	4	Cross-sectional

Urban 35 (25.9)<br>Rural 100 (74)	Normative data	1	Cross-sectional
N/I	Diagnostic accuracy	1	Cross-sectional
Rural and Urban	Reliability, factor analysis	2	Cross-sectional
Rural and Urban	Reliability, diagnostic accuracy, Construct and/or Criterion Validity	3	Cross-sectional
N/I	Reliability, Construct and/or Criterion Validity, diagnostic accuracy, fator analysis, rash model	5	cross-sectional
N/I	Factor analysis	1	cross-sectional
Urban and rural	Reliability, diagnostic accuracy, Construct and/or Criterion Validity, factor analysis, development	5	Cross-sectional
Rural	Reliability, Construct and/or Criterion Validity, rash model	3	Cross-sectional
Urban and rural	Diagnostic accuracy, factor analysis	2	Cross-sectional
N/I	Translation, adaptation	2	Translation and content validation

N/I	Construct and/or Criterion Validity, factor analysis	2	Cross-sectional
Urban	Reliability	1	Cross-sectional
Urban	Realiability, Construct and/or Criterion Validity, diagnostic accuracy, factor analysis, rash model	5	Cross-sectional
N/I	Reliability and factor analysis	2	Cross-sectional
N/I	Reliability, diagnostic accuracy, Construct and/or Criterion Validity, factor analysis, rash analysis	5	Cross-sectional
N/I	Reliability, factor analysis, adaptation	3	Cross-sectional
N/I	Reliability, diagnostic accuracy, Construct and/or Criterion Validity.	3	Cross-sectional
Urban	Reliability	1	Cross-sectional
Urban	Reliability, Construct and/or Criterion Validity, factor analysis	3	Cross-sectional
Urban	Reliability, factor analysis	2	Cross-sectional

N/I	Reliability, factor analysis	2	Cross-sectional
Urban and rural	rasch analysis	1	cross-sectional
Urban	adaptation, translation, reliability, rasch analysis	4	Cross-sectional
Urban	factor analysis, Construct and/or Criterion Validity, reliability	2	Cross-sectional
N/I	Reliability, Diagnostic Accuracy, factor analysis, adaptation	4	cross-sectional
Rural	Reliability, diagnostic accuracy	2	Cross-sectional
Urban	Reliability, diagnostic accuracy, Construct and/or Criterion Validity, factor analysis	4	Cross-sectional
N/I	Diagnostic Accuracy, Construct and/or Criterion Validity	2	Cross-sectional
N/I	Reliability, diagnostic accuracy, Construct and/or Criterion Validity, factor analysis	4	Cross-sectional
N/I	Reliability, Construct and/or Criterion Validity, factor analisys	3	Cross-sectional

N/I	Reliability, diagnostic accuracy, Construct and/or Criterion Validity, translation	4	Cross-sectional
Rural and Urban	factor analysis	1	Cross-sectional
N/I	Reliability, factor analysis, adaptation	3	Cross-sectional
N/I	Reliability, diagnostic accuracy, Construct and/or Criterion Validity	3	Cross-sectional
N/I	Diagnostic Accuracy, Construct and/or Criterion Validity	2	cross-sectional
N/I	Diagnostic accuracy, Construct and/or Criterion Validity	2	Cross-sectional
Urban	Construct and/or Criterion Validity, reliability	2	Cross-sectional
N/I	Reliability, factor analysis	2	Cross-sectional
rural and Urban(Peru and Mexico). Other countries Urban	Factor analysis	1	Cross-sectional
Urban and Rural	Reliability, factors analysis	2	Cross-sectional

Urban and rural	Reliability, diagnostic accuracy, Construct and/or Criterion Validity, factor analysis	4	Cross-sectional
N/I	Reliability, factor analysis	2	Cross-sectional
Urban - multicentre	Adaptation, reliability, factor analysis, Construct and/or Criterion Validity	4	Cross-sectional
Urban-multicentre	reliability, factor analysis, Construct and/or Criterion Validity, divergent validity	4	Cross-sectional
Urban (cuba, dominican republic, venezuela) Urban and rural (peru, mexico)	factor analysis, reliability	2	Cross-sectional
Urban and rural	normative data	1	Cross-sectional
Urban and rural	Reliability, diagnostic accuracy, Construct and/or Criterion Validity, factor analysis, translation.	5	Cross-sectional
N/I	Reliability, factor analysis, rash model	3	Longitudinal
N/I	Factor analysis, Construct and/or Criterion Validity, divergent validity, reliability	4	Cross-sectional
Rural and Urban	Reliability, diagnostic accuracy	2	Cross-sectional

N/I	Reliability, diagnostic accuracy	2	Cross-sectional
N/I	Reliability, diagnostic accuracy, Construct and/or Criterion Validity	3	Cross-sectional
Urban	Reliability, factor analysis, translation, adaptation	4	Cross-sectional
Urban	Reliability, Construct and/or Criterion Validity, divergent validity, factor analysis	4	Cross-sectional
Urban	Reliability, development	2	Cross-sectional
N/I	Factor analysis, reliability, Construct and/or Criterion Validity	3	Cross-sectional
Urban	Reliability, factor analysis	2	Cross-sectional
N/I	Reliability, Construct and/or Criterion Validity, divergent validity, factor analysis	4	Cross-sectional
Urban	Construct and/or Criterion Validity, divergent validity	2	Cross-sectional
N/I	Diagnostic accuracy	1	Cross-sectional

N/I	Normative data	1	Cross-sectional
community Urban and rural stratified	reliability, Construct and/or Criterion Validity, diagnostic accuracy	3	Cross-sectional
Urban	Construct and/or Criterion Validity, reliability	2	Cross-sectional
Urban	diagnostic accuracy, factor analysis, Construct and/or Criterion Validity, reliability	3	Cross-sectional
N/I	reliability, adaption, factor analysis, Construct and/or Criterion Validity	4	Cross-sectional
Urban	reliability, Construct and/or Criterion Validity, divergent validity, translation and adaptation	5	Cross-sectional
Urban	reliability, factor analysis, Construct and/or Criterion Validity	3	Cross-sectional
Urban	translation, adaptation, reliability, Construct and/or Criterion Validity, factor analysis	5	Cross-sectional
N/I	Reliability, Factor analysis, development	3	Cross-sectional
N/I	Diagnostic accuracy	1	Cross-sectional

N/I	Reliability, factor analisys	2	cross-sectional
N/I	Diagnostic accuracy, Construct and/or Criterion Validity	2	Cross-sectional
N/I	Reliability, diagnostic accuracy, Construct and/or Criterion Validity	3	cross-sectional
N/I	Reliability, diagnostic accuracy, fator analysis	3	cross-sectional
N/I	Reliability, diagnostic accuracy, Construct and/or Criterion Validity	3	Cross-sectional
N/I	Reliability, Construct and/or Criterion Validity,factor analysis,	3	Cross-sectional
N/I	Reliability, Construct and/or Criterion Validity, divergent validity, factor analysis	4	Cross-sectional
N/I	Reliability, Factor analysis, Construct and/or Criterion Validity	3	Cross-sectional
Semi-Urban	Construct and/or Criterion Validity	1	Cross-sectional
N/I	Reliability, Construct and/or Criterion Validity, divergent validity, factor analysis	4	Cross-sectional

N/I	Construct and/or Criterion Validity, divergent validity, factor analysis	3	Cross-sectional
N/I	Reliability, Construct and/or Criterion Validity, divergent validity, factor analysis	4	Cross-sectional
N/I	Reliability, Construct and/or Criterion Validity, divergent validity, factor analysis	4	Cross-sectional
Urban	Diagnostic accuracy	1	Cross-sectional
Urban	Reliability, Construct and/or Criterion Validity	2	Cross-sectional
Urban	Reliability, Construct and/or Criterion Validity, diagnostic accuracy	3	Cross-sectional
Urban	Reliability, Construct and/or Criterion Validity, diagnostic accuracy	3	Cross-sectional
Urban	Diagnostic accuracy, Construct and/or Criterion Validity	2	Cross-sectional
Urban	reliability, diagnostic accuracy, Construct and/or Criterion Validity	3	Cross-sectional
N/I	reliability, factor analysis	2	Cross-sectional

Urban	Construct and/or Criterion Validity, diagnostic accuracy	2	Cross-sectional
Urban	Construct and/or Criterion Validity, diagnostic accuracy	2	Cross-sectional
Urban	Diagnostic accuracy	1	Cross-sectional
N/I	Diagnostic Accuracy, Construct and/or Criterion Validity	2	Cross-sectional
N/I	Reliability, Diagnostic Accuracy, Construct and/or Criterion Validity	3	cross-sectional
N/I	Reliability, diagnostic accuracy, Construct and/or Criterion Validity	3	Cross-sectional
N/I	Diagnostic accuracy	1	Cross-sectional
N/I	Reliability, factor analysis	2	Cross-sectional
N/I	Reliability, Construct and/or Criterion Validity, fator analysis	3	Cross-sectional
N/I	Diagnostic accuracy	1	Cross-sectional

N/I	Reliability, factor analysis	2	Cross-sectional
N/I	Reliability, Diagnostic accuracy, Construct and/or Criterion Validity, factor analysis	4	Cross-sectional
N/I	Reliability, Construct and/or Criterion Validity, factor analysis, rash model	4	Cross-sectional
N/I	Reliability, Construct and/or Criterion Validity, diagnostic accuracy	3	Cross-sectional
Urban	Reliability, diagnostic accuracy, Construct and/or Criterion Validity, adaptation	4	Cross-sectional
N/I	Reliability, factor analysis	2	Cross-sectional
N/I	Reliability, Construct and/or Criterion Validity, factor analysis, translation	4	Cross-sectional
N/I	Reliability, Construct and/or Criterion Validity, factor analysis	3	Cross-sectional
N/I	Reliability, Construct and/or Criterion Validity, factor analysis	3	Cross-sectional
N/I	Reliability, factor analysis	2	cross-sectional

N/I	Reliability, diagnostic accuracy,	2	Cross-sectional
N/I	Reliability, factor analysis	2	Cross-sectional
N/I	Diagnostic accuracy, Construct and/or Criterion Validity,	2	Cross-sectional

Aim	purpose tool	age
To evaluate the sensitivity and specificity of the MDRS, and to localize the optimal cutoff score for MCI.	Cognition	≥ 60 years
To establish the validity, reliability and discriminative proprietiesand translation of AD8-arg for use in	Cognition	mean age 70 years
to assess the construct and concurrent validity of the Spanish-language version	Cognition	≥ 60
Validation of the screening test in a spanish-speaking population, identification of cut-off scores	Cognition	≥ 60
translate, culturally adapt and validate the complete RMBPC scale for use in spanish-speaking populations	Cognition	mn age 81
validate the Argentine version of the MBT in a Latin American population and to estimate the diagnostic accuracy as a	Cognition	mn age 65.3 MCI, 67.5 controls
Evaluate discriminant validity of ECog in cognitively intact controls (CN) MCI and mild AD to establish diagnostic	Cognition	mn age 71.89
To estimate the diagnostic accuracyof the Phototest for cognitive impairment and dementia and to compare it with that of	Cognition	60 to 85 years
To present the Argentinian validation of the brief CSID version	Cognition	≥ 60 years

To analyze the psychometric properties of ACE in a sample of old people with low soICo-educational level with and	Cognition	mean ≥ 78 years
To assess a new way of interpreting widely used screening tests, generating a new score: the CSIS (Combined	Cognition	60 - 85 years, Control group = 76.33 ± 6.18, MCI Group = 75.74 ± 6.38
To present the translation and linguistic and cultural adaptation of the Spanish Minilinguistic State Examinational in Argentina.	Aphasia	55–85 years, Pilot 1= 71.15 (9.02), pilot 2= 63.61 (10.40)
To obtain normative data for the Montreal Cognitive Assessment (MoCA) in Argentinean adults and older adults in the	Cognition	≥ 50 years, mean 66.1 (8.7)
The aim of this study is to assess the content validity and reliability of the CQC by means of expert judgement and internal	Cognition	N/I
to perform an analysis of the information quality of the CQQ from the point of view of item response theory (IRT), in order to	Cognition	Mean = 66.8 years
To validate the MoCA in the elderly and study its usefulness in MCI and MD.	Cognition	≥ 60 years, Mean = 73.4 (SD 6.9)
To analyze the internal structure of the GDS-VE, for penICl-paper and Internet modalities examining the metric	Depression	between 60 and 93 years (M=70.70; SD=6.81)
To determine its fit in an Argentine elderly adult sample and to confirm the three-dimensional structure suggested by the	Coping	60 to 92 years
To investigate the psychometric properties of Rami Cognitive Reserve Questionnaire in an Argentinian population.	Cognition	mean age= 66.1

To validate the ACE-III test in an Argentinean and Chilean population.	Cognition	Control=68.03, ATD= 76.9,bv-FTD=68.06
Develop a new tool; assess reliability and validity of the tool	Geriatric assesment (health)	≥ 65 years
Translation and cross-cultural adaptation into Brazilian Portuguese, and study of the psychometric properties of the tool	Pain	≥ 80 years
To verify the applicability and validity of the Brazilian version of the Disability Assessment for Dementia (DAD-Br).	Daily activities cognitive impairment	≥ 65 years
To obtain internal construct and criteria validity for the Center of Epidemiological Studies – Depression scale in	Depression	≥ 60 years
To examine psychometric indicators of the Emotional Regulation Questionnaire (ERQ) and their relationship with measures	Emotional regulation	≥ 60 years
To evalute the applicability of the portuguese-brazilian version	Cognition	≥ 65 years
To validate the Pain Assessment Checklist for Seniors with Limited Ability to Communicate –	Pain	≥ 60 years
To assess the validity of the MSQ by comparing the screening scores against a standardized psychiatric evaluation, the Clinical	Cognition	≥ 65 years
The aim of this study was to devise an adapted version of the ANU-ADRI for use in Brazil.	Cognition	≥ 40 years (60 years mean age)

To propose a new version of MMSE as a screening test to assess Illiterate and low education people.	Cognition	≥ 60 years
To assess the cognitive performance of low and middle educated old people in a modified version of the adapted to	Cognition	≥ 60 years
To validate the Leganés cognitive test (LCT) for cognitive screening in low educated elderly Brazilians.	Cognition	65 to 74 years
To evaluate the reliability, validity and clinical utility of a Brazilian telephone version of the Mini Mental State examination (Braztel-	Cognition	≥ 60 years
To examine executive function using the Behavioral Assessment of the Dysexecutive Syndrome for	Cognition	≥ 60 years
To investigate the accuracy of the Brazilian version of the Addenbrooke Cognitive Examination-revised (ACE-R) in the diagnosis of mild	Cognition	≥ 70 years
To translate and adapt the ACE-R for use in the Brazilian population.	Cognition	≥ 60 years
To assess Construct and/or Criterion Validity and reliability	Quality of life	≥ 60 years
To present the validation process and the psychometric properties of the Brazilian version of the Meaning in Life	Meaning in life	≥ 18 years (18-91 years)
To evaluate whether the CCQ can accurately distinguish normal subjects from individuals with Mild Cognitive Impairment	Cognition	≥ 60 years

Adapt and evaluate the psychometric properties of the Vulnerability to Abuse Screening Scale.	Mistreatment	≥ 60 years
To validate the Rowland Universal Dementia Assessment Scale for use in Brazil (RUDAS-BR).	Cognition	≥ 60 years
To describe the process of translating and adapting the Relationship Scales Questionnaire (RSQ) from English into Brazilian	Relationships	≥ 60 yeasr
Evaluated the accuracy of the CDR for determining and staging dementia	Cognition	≥ 60 years
To evaluate the diagnostic sensitivity, specificity, and agreement of the Dementia Rating Scale with clinical diagnosis of	Cognition	≥ 60
Assess reliability and factor analysis of the adaptated tool	Pleasant activities	≥ 60 years
To examine the normative data of WHODAS 2.0-BO for older Brazilians	Health (Disability)	≥ 60 years
Diagnostic accuracy for mild cognitive impairment and dementia.	Cognition	AD = 74.67 años (± 7.9)<br>MCI = 70.03 años (± 6.2)<br>NC = 68.27 años (± 6.1)
To evaluate the psychometric properties of the SKT in a sample of older Brazilians;	Cognition	≥ 60 years (ranged 43-85 years)
Describing the development and validation of the tool in Brazil	Quality of life	≥ 60 years

To investigate the properties of the tool for predicting delirium in hip fracture patients.	Cognition	≥ 60 years
To deter-<br>mine intra and inter–rater reliabilities	Cognition	≥ 60 years
To investigate internal structure validity evidence of the Brazilian version	Pleasant activities	≥ 60 years
To investigate the va-<br>lidity of the MAST in a sample of male subjects attending a geriatric outpatient service of a	Alcohol abuse	≥ 60
The aim of this study was to evaluate the diagnostic sensitivity, specificity, and agreement of the DRS with clinical diagnosis of IC	Cognition	≥ 60
To screen some IC instruments frequently used by Brazilian speICalists in dementia were analyzed to	Cognition	≥ 65 years
To verify<br>the efficacy of simple instruments in the screening of cognitive impairment in elders.	Cognition	≥ 65 years
To evaluate the internal consistency	Alcohol abuse	≥ 60
To describe and compare the performance of illiterate and low educated older adults, without evidence of cognitive	Cognition	≥ 60 years
To validate the test in Brazil and for different groups of older people	Depression	≥ 60

The first phase of the crosscultural equivalence of Section A in the Cambridge Examination for Mental Disorders of the	Cognition	≥ 60
The objective of this study was to translate and evaluate the use of the CASP-19 amongst older Brazilians.	Quality of life	≥ 65
To translate and adapt the English language "PACSLAC"	Pain	≥ 60 years
To compare the accuracy of four scales, and to determine the inter-rater reliability and the influence of schooling on Clock	Cognition	≥ 65 years
To determine the accuracy of the Brazilian version of the Informant Questionnaire on Cognitive Decline in the	Cognition	≥ 65 years
To assess the psychometric characteristics of the Mini-Mental State Examination in elderly outpatients who seek	Cognition	≥ 65 years
To translate the Perceived Stress Scale into Brazilian Portu- guese, and to assess its validity for measuring	Stress	≥ 60 years
to determine the best cut-off point of the MMSE for the diagnosis of dementia in a sample of elderly subjects	Cognition	≥ 60 years
To assess the test-retest reliability of short versions of the Geriatric Depression Scale (GDS) with 1, 4, 10, and 15 items.	Depression	≥ 60 years
To determine the validity of short Geriatric Depression Scale (GDS) versions for the detection of a major depressive	Depression	≥ 60 years

To verify the factor structure of the Geriatric Depression Scale of 15 items (GDS 15)	Depression	≥ 60 years
To develop an informant-based instrument that would provide a valid estimate of premorbid cognitive abilities	Cognition	≥ 60 years
To provide age-corrected and education-corrected norms for the Montreal Cognitive Assessment (MoCA) and the Memory	Cognition	50-90 years (stratified)
To evaluate whether the CAMCOG can be used as an accurate screening test among AD patients and normal controls with different educational levels.	Cognition	≥ 60 years
to evaluate the sensitivity and specificity of the CDT in a sample composed of older adults with Alzheimer's disease (AD)	Cognition	≥ 60 years
To translate and adapt the test to the Brazilian context, and to assess its construct validation.	Cognition	≥ 60 years
To verify the agreement and reproduICbility of the QOL assessment instruments WHOQOL-Bref and WHOQOL-Old, in	Quality of life	≥ 60 years
To describe the performance of a sample of healthy older women, who were illiterate or had low educational level, on the	Decision making	60–79 years
To determine the validity of the Brazilian version of the Geriatric Depression Scale (GDS) with 30 (GDS-30), 15 (GDS-15), 10 (GDS-	Depression	≥ 60 years
To compare the reliability and convergent validity of instruments assessing quality of life in Brazilian older adults.	Quality of life	≥ 60 years

To investigate whether longitudinal data on the structure of the mini mental state examination (MMSE) collected in an	Cognition	≥ 60 years
To investigate the validity of previously suggested dimensions underlying MMSE and differences in associations of these	Cognition	≥ 60 years
(1) to estimate the prevalence of cognitive impairment, using several cut-off points recommended by Brazilian	Cognition	≥ 60 years
To analyze the predictive function of the Pentagon's drawing test in elderly patients with organic and non-organic disorders.	Cognition	≥ 60 years
To correlate the results of CDT and other used diagnostic tests for dementia by CDR levels, providing	Cognition	≥ 60 years
To assess which MoCA subtests could best discriminate between healthy controls (HC), participants with MCI, and	Cognition	≥ 60 years
To correlate neuropsicometric tests in elderly over 4 years of schooling and assess MoCA accuracy in diagnosing	Cognition	≥ 60 years
To explore whether the 15-item GDS is a suitable instrument in a Brazilian sample.	Depression	≥ 60 years
To evaluate the Brazilian version of WHOQOL-OLD Module and to test potential changes to the instrument to increase its	Quality of life	≥ 60 years
To describe the psychometric properties of the World Health Organization’s Quality of Life Instrument–Short	Quality of life	≥ 60 years

To assess divergent validity and both intra-rater and inter-rater reliability for the use of COPM in MCI patients.	Daily activities cognitive impairment	≥ 65 years
Translation, transcultural adaptation and application to Brazilian Portuguese of the Alzheimer's Disease	Cognition	≥ 60 years
To produce the cross-cultural validation of the AD8 interview to the Brazilian Portuguese Language.	Cognition	≥ 65 years
The objective of the study was the analysis of agreement between the CDR scale with diagnostic criteria and mini mental	Cognition	70.8 mean
To verify the accuracy of prospective memory (ProM) tests in Alzheimer's disease (AD).	Cognition	≥ 70 years mean
to evaluate the psychometric properties of the Brazilian Portuguese version GAI (GAI-BR) in a sample from	Anxiety	≥ 60 years
To examine the reliability and validity of the Brazilian version of the MoCA test (MoCA-BR) in a sample of older individuals with	Cognition	≥ 65 years
To translate and culturally adapt the Brazilian Portuguese version of the CANS-MCI (CANS-MCI-BR) and to evaluate its	Cognition	≥ 65 years
To assess the performance of healthy elderly, MCI patients and mild AD patients using the Brazilian version of the	Cognition	≥ 60 years
To evaluate the validity of the Portuguese version of the Clinical Dementia Rating for classifying the cognitive	Cognition	≥ 65 years

To determine the prevalence of depressive symptoms among elderly and correlate the agreement between the	Depression	≥ 60 years
To compare the internal consistency and criterion validity of each against the gold standard of the Schedules for Clinical	Depression	≥ 60 years (second phase ≥ 75 years)
Describe translation, adaptation, Assess validity and derive normative values for Brazil	Cognition	≥ 60
evaluate reliability and Construct and/or Criterion Validity of QOL-AD	Quality of life	≥ 60
evaluate diagnostic accuracy in illiterate, low ed and middle ed sample vs DSM-IV dementia	Cognition	mn age 77
Reliability (test retest and interrater reliability) of the tool	Cognition	≥ 60 mn 76.1 ± SD 7.1 years
validate the tool in a public outpatient clinic	Depression	≥ 65
describe item reduction and distribution into dimensions in construction of a quality of life evaluation instrument for	Quality of life	≥ 60
Evaluate reliability (internal consistency) and validity (construct/criterion) of ADLs measure	Cognition	≥ 60
To search for evidence of reliability and Construct and/or Criterion Validity of the RAVLT, and to assess the influence of age,	Cognition	≥ 60

Evaluate performance of a short Brazilian version of IQCODE, compare against cognitive and functional scales, and investigate cut-	Cognition	mean age=70.5 years
Investigate the performance of Brazilian versions of the IQCODE L, S and a new short version (SBr) (15 items) for	Cognition	≥ 60 years
To evaluate the psychometric properties of the short version of the GDS in patients with CAD treated at a Cardiology	Depression	≥ 65 years
To provide normative data for the Brazilian version of the Montreal Cognitive Assessment (MoCA-BR) and to measure the effect	Cognition	≥ 65
To assess validity of the tool for screening dementia in low-literate elders	Cognition	≥ 65
To assess the reliability and concurrent validity (education, MMSE) and assess the effect of education	Cognition	60-85
To investigate diagnostic accuracy of the MMSE in a sample of low education and soICoeconoMCI status	Cognition	≥ 65
Translation and cross-cultural adaptation of the Informant Questionnaire on Cognitive Decline in the Elderly (IQCODE) and the	Cognition	23-85 years (informants)<br>≥ 65 years (patients)
To determine the criterion validity of the Brazilian version of the IQCODE (IQCODE-BR), inaddition to the evaluation of age and	Cognition	≥ 65
Examine validity of SRQ-20 in population of older adults from lowsoICoeconoMCI status living in Sao Paulo, Brazil,	Health	≥ 65

Adapt the ADAS-Cog for use for the Brazilian population	Cognition	≥ 50
To propose a cut-off for the World Health Organization Quality of Life-Bref (WHOQOL-bref) as a predictor of quality of life	Quality of life	≥ 60
To determine Vulnerable Elders Survey (VES-13) and WHOQOL-bref cutoff points to detect poor quality of life (QoL) in older	Quality of life	≥ 60
To analyze the applicability and psychometric properties of the Portuguese version of the Geriatric Anxiety Inventory	Anxiety	≥ 55
Validating the convergent and discriminating construct and determining the reliability of STADP	Cognition	≥ 60
To determine cut off values for mild cognitive impairment and mild dementia in a cohort of 8 years education or more	Cognition	mn 69.6 years
To adapt the CAMCOG to brazilian portuguese	Cognition	60-90
assess the validity of Montgomery-[Angstrom]sberg. Depression Rating Scale (MADRS) and Cornell Scale	Depression	≥ 65
To perform the semantic-cultural validation and internal consistency analysis of the 10-item Purpose in Life scale of Ryff	Purpose in life	≥ 80
To translate, adapt and validate the Autobiographic Episodic Memory Interview (EAMI)15 in a population of	Cognition	≥ 65

To examine the psychometric properties of the Brazilian version of the Multifactorial Memory Questionnaire (MMQ)	Cognition	≥ 60
assess the feasibility, reliability and convergent validity of the Camberwell Assessment of Needs for the Elderly Scale in older	Needs	mn age 72.8 (65-88)
Assess reliability and consistency of the ENEDAM structured interview for determination of demetnia	Cognition	63-86 (md 75.5)
Examine the validity of the Brazilian version of the RBMT to detect cognitive decline	Cognition	NC: 68.21 (6.19)<br>MCI: 69.76 (5.85)<br>AD: 74.56 (5.98)
Assessment of the validity and reliability of the 49-item BRIEF-SRC scale among older Brazilians	Spirituality	community mn age = 67.22, care home residents mn age = 76.56
assess psychometric properties of the scale	Social support	≥ 60
To develop a quality of life and swallowing questionnaire for individuals with PD.	Quality of life	mn 62.7 (development), pilot mn 67.0
Evaluate the convergent, divergent and discriminative validity of the Brazilian version of LHFQ applied to elderly HF	Quality of life	≥ 60
Examine the validity and reliability of the "Multidimensional Assessment of Older People in Primary Care	Health status	≥ 60
Evaluates Construct and/or Criterion Validity of Brazilian Portuguese version of instrument Caregiver Abuse Screen	Mistreatment	≥ 65

Revisit the dimensional structure and propose a shorter version of the instrument.	Cognition	≥ 65
Obtain normative data for the Brazilian portuguese translation of the tool	Cognition	15-85 (subset aged ≥ 60 given)
to propose a care needs classification for older people living in the community	Needs	≥ 60
to conduct an exploratory factor analysis of baptistas depression scale, version for older people	Depression	60-90
To investigate the applicability of the Bayer - Activities of Daily Living scale and its effiICency in differentiating	Daily activities cognitive impairment	≥ 60 years
To analyze the subtest Cube drawing from MoCA and describe the sensitivity and specificity of this subtest in healthy	Cognition	≥ 60 years
To establish the correlation between the Visual Analogue Scale of Happiness and the Cornell Scale for Depression in	Depression	≥ 60 years
The aim was to provide MoCA norms and accuracy data for seniors with a lower education level, including illiterates	Cognition	≥ 60 years
To compare the Brazilian versions of the Nottingham Health Profile (NHP) and the Short Form Health Survey-36 (SF-36)	Quality of Life	≥ 60 years
To evaluate the applicability and the psychometric properties of Montreal Cogni-tive Assessment Brazilian	Cognition	≥ 65 years

The present study performed the cross-cultural adaptation of the Pain Locus of Control Scale (C form of	Pain	≥ 60 years
To analyze the psychometric properties of the MMQ in a Brazilian sample	Cognition	≥ 60 years
To evaluate a brief version of the CAMCOG for illiterate older adults (CAMCOG-BILL) with Alzheimer's dementia (AD)	Cognition	60 to 97 years
To determine the diagnostic accuracy of the Addenbrooke's Cognitive Examination Revised (ACE-R) as a cognitive screening	Cognition	Mean 73.16; SD 8.71
To verify the factor structure of the LIFE-H 3.1-Brasil in a sample of independent older adults, and to analyze their	Quality ofsocial participation	60 to 96 years
To evaluate the ability of the FTD Rating Scale (FTD-FRS) to detect functional and behavioral changes in patients diagnosed with	Cognition	50-87 years; bvFTD: Mean = 66.94 years<br>PPA: Mean = 61.42 years<br>AD: Media = 74.15 years
To assess the validity and reliability of the UCLA Loneliness Scale, version 3, in a sample of aged Brazilians.	Loneliness	≥ 60 years
To present the process of cross-cultural adaptation of pFCSRT-IRand to discuss the adaptation processes of instruments that use	Cognition	between the ages of 64 and 84
Expand the GDS-15 normative data to a sample of older adults from a community in Rio de Janeiro.	Depression	≥ 60 años
To present evidence of validity of BGT in healthy elderly and with diagnosis of dementia.	Cognition	≥ 60

To verify the validity and reliability of the Psychological Distress Scale (K10) for screening mental distress.	Mental distress	Mean age = 66.21 years (± 4.22)
Evaluate the accuracy of two semantic categories of the verbal fluency test (supermarket and animal categories) to separate	Cognition	≥ 60 years
To cross-culturally adapt and assess the psychometric properties of the Brazilian version of the Modified Fatigue Impact	Fatigue	≥ 50 years
To translate the CFI to Brazilian Portuguese, perform a cross-cultural adaptation, and analyze its diagnostic accuracy to	Cognition	≥ 45 years, mean = 63.4 (SD=11.3)
To develop and evaluate the psychometric qualities of a new instrument to assess older adults' lifestyles.	Lifestyle	≥ 60 years
To validate the translated and culturally adapted version of the Multidimensional Individual and	Resilience	≥ 60 years
To assess the internal consistency of instruments used in Brazil to measure situations of violence against the elderly person	Mistreatment	≥ 60 years
To validate to Brazilian version of the "Pain Intensity Measure for Persons with Dementia - PIMD-p".	Pain	≥ 60 years
To investigate the diagnostic accuracy of Addenbrooke's Cognitive Examination-Revised (ACE-R) for differentiating	Cognition	Patient groups= 45 to 89 Control group = 50 to 93 years
To verify the psychometric properties of the Dysexecutive Questionnaire (DEX) through exploratory factor	Cognition	≥ 60 years

To evaluate the Construct and/or Criterion Validity and internal consistency of the Brazilian version of Leisure Attitude	Pleasant activities	≥ 60
to verify the validity and reliability of the Psychological Distress Scale for screening mental distress.	Mental distress	60 to 76 years.
This study aimed to translate the Prefrontal Symptoms Inventory (PSI) (abbreviated version) for the elderly into Brazilian	Cognition	≥ 60 years
To assess item difficulty in the Brazilian version of the MMSE using the Rasch model and to detect	Cognition	≥ 65 years
To develop and validate a predictive score for inICdent delirium.	Delirium	≥ 65 years
To adapt and evaluate the psychometric properties of the tool in chilean population	Quality of life	≥ 60
To evaluate the efficacy of the tool as a neuropsychological instrument	Cognition	≥ 60 years
To validate the “Successful Aging Inventory” (SAI) in Chilean older people.	Health (successful aging)	≥ 60
to adapt a Brief Index of Religiousness and Spirituality,	Spirituality	≥ 60
To validate the Questionnaire of Communitysocial Support	Social support	≥ 60

To test the effectiveness of the 5-item version of the Geriatric Depression Scale (5-GDS)	Depression	≥ 60
To adapt and to validate the Eurotest in Chile.	Cognition	≥ 65
To assess the per-formance of the MMSE-EFAM to detect dementia.	Cognition	≥ 65
To validate short-form-36 health survey (SF-36) in a of Chilean older people	Quality of life	≥ 60
To validate the Geriatric Anxiety Inventory (GAI) in the country.	Anxiety	≥ 60 years
Adapt and conduct preliminary validation of the tool in Chile	Happiness	≥ 65
adapt and validate the tool in spanish speakers	Cognition	≥ 65
evaluate psychometric properties of the Addenbrooke's Cognitive Examination - Revised (ACE-R) in older chileans	Cognition	≥ 60
Determine the psychometric properties of the Multidimensional Scale Perceivedsocial Support Zimet in older adults.	Social support	≥ 65 years
To validate and determine cutoff points for a cognitive impairment screening test composed by theFolstein Mini Mental	Cognition	≥ 64

To compare the factor structure of the Quality of Life Scale WHOQoL-Old of the World Health Organization with the	Quality of life	60-90
This study examines the psychometric properties of the Perceivedsocial Support Questionnaire (PSSQ) for a multi-ethnic	Social support	Mean age (SD)= 72.1 (7.8) años
to (1) adapt FAB in a Chilean population; (2) study the psychometric properties of the FAB in a Chilean population; (3)	Cognition	Mean: HC = 55.35±18.096, DS= 74.2±7.626
To update the normative values, and diagnostic effiICency statistics of the MoCA to detect mild NCD in the Chilean population.	Cognition	NHE Mage = 71.49, SD age = 7.64; Nmild NCD Mage = 76.92, SD age = 8.71; Nmajor NCD Mage = 82.17, SD age = 7.89
To analyze the psychometric properties of the Brief Resilient Coping Scale (BRCS) for a multiethnic sample of	Coping and resilience	≥ 60 años, 72.07 years, SD= 7.81
To evaluate the psychometric properties (evidence of validity through factor structure and evidence of reliability)	Spirituality	Mean =71 years (SD= 8.2338)
To adapt and evaluate the psychometric characteristics of the Scale of Adaptation of the Older Adults to their Residence	Adaptation to nursing homes	aged 79 ± 7 years
To determine the factorial structure and the internal reliability of the Mini Mental State Examination (MMSE), the Montreal	Cognition	aged 54 to 88 years, mean = 69 years
To analyze the clinical utility of the Phototest, through telemediICne, to identify mild cognitive impairment in rural older	Cognition	≥ 65 years; MCI=70.342±9.51, HC=71.12±7.85
To assess the measurement properties (reliability, factor structure, and criterion validity) of the Patient Health	Depression	65 and 80 years

To obtain normative data on RUDAS in older Chilean people with up to 12 years of schooling, and to determine whether age	Cognition	≥ 60 years
This study aimed to obtain psychometric properties of visual Buschke and Grober The Free and Cued Selective Reminding Test	Cognition	HOA group 71.49 age mean (SD = ±7.64); mild NCD group 76.92 age mean (SD = ±8.71); NCD group (SD = ±7.89)
To evaluate the factorial and reliability structure of the Multidimensional Scale of Perceivedsocial Support within autonomous older	Social support	≥ 60 years
To validate the SF-12 (short-form) health related quality of care questionnaire (HRQoL) as an alternative of the SF-36	Quality of life	≥ 60 years
To analyse the psychometric properties of the De Jong Gierveld Loneliness Scale, 6-item version (DJGLS-6), in a	Loneliness	Mean age: Non-indigenous= 73.13 (7.68) Aymara = 70.85 (7.81) Mapuche= 72.07 (7.83)
To perform a psychometric evaluation of the ACE-III from a 2-parameter IRT model.	Cognition	≥ 60 years
Based on a prior categorization of six types of losses, developed upon the basis of reports by older adults, we sought to	Losses Experienced	≥ 65 years
The aim of this study was to analyze the psychometric properties of the 15-item Geriatric Depression Scale (GDS-15)	Depression	≥ 60 years
To evaluate the psychometric properties of Addenbrooke's Cognitive Examination III in a large sample of elderly people	Cognition	≥ 60 years
To translate into Chilean Spanish and carry out the content validation of the Quality of Life in Alzheimer's Disease scale	Quality of life	N/I

To evaluate measurement invariance of the SWLS across older adults from Chile and Ecuador	Life satisfaction	≥ 60
To standardize the Scale of Depression of Yesavage (reduced versión) in noninstitutionalized older adultos	Depression	≥ 60 years
To explore the psychometric properties of the Brief Assessment Scale for Depression (BAS-DEP) and	Depression	≥ 60 years
To determine the internal consistency, factor structure and construct reliability of the Yesavage Geriatric Depression Scale	Depression	≥ 65 years
To establish the psychometric properties of the screening test for dementia Pesotest in clinical and non-clinical	Cognition	≥ 65 years
To validate the loneliness measurement scale ESTE in adult population who assist to elderly groups in Bello.	Loneliness	≥ 55
To validate the MOCA-S	Cognition	≥ 65 years
To report on the applicability of the Spanish version of the Montreal Cognitive Assessment test (MoCA-S)	Cognition	≥ 65 years
The aim of this paper was to study the internal consistency, the factor structure and the construct reliability of the GDS-15	Depression	≥ 60
To analyses the psychometrics properties of the ESTE-R scacle ina colombian sample	Loneliness	≥ 65 years

To determine the internal consistency, dimensionality, differential item functioning (DIF) by gender and stability of the	Sexuality	≥ 60 years
To assess validity of the MMSE using a rasch model in a community sample	Cognition	≥ 60
To evaluate the validity evidences of a unidimensional approach to WHOQOL-BEF in older adults	Quality of life	N/I
test psychometric properties of this questionnaire based on a representative sample of the general population of	Optimism	51-60: n = 237; ≥ 60 n = 217
To adapt and validate the Family Abuse Screening Questionnaire for Elderly People in Colombia	Family abuse	N/I
To provide evidence that supports the validity and reliability of the Colombian version of the Addenbrooke's Cognitive	Cognition	≥ 60 years
To establish the validity and reliability of the Anosognosia Questionnaire in Dementia (AQ-D) in individuals diagnosed with	Anosognosia	Mean age 67.88 years (SD = 11.80)
To evaluate whether the Delirium Diagnostic Tool-Provisional (DDT-Pro), a 0–9 point scale with three items each representing	Delirium	≥ 60 years, No delirium (n = 98): 73.4 ± 8.2 años SSD (Subsyndromal delirium) (n = 54): 78.2 ± 9.4 años
To evaluate the psychometric properties of the PD-CRS in a Colombian population and evaluate the concurrent validity	Cognition	median age was 68 years (IQR 57-74)
The objective of this study was to know the dimensionality and internal consistency of the MCS-15 in Colombian older adults.	Cognition	60 and 98 years

To translate the DDT-Pro into Spanish to validate its performance among patients ≥ 60 years old who were admitted to an	Delirium	≥ 60 years
validity of a common measure of health-related quality of life (Short-Form-36 [SF-36]) in cognitively healthy older adults living	Quality of life	60-85
To analyze the psychometric characteristics of the Wagnild and Young Resilience Scale Argentine	Resilience	ADEP Pilot = 69.38 (± 2.71) PIPAMRO validation =6 9.31 (± 3.08)
To validate the Cuban ACE-R and to compare it with the MMSE	Cognition	≥ 65 years (
To compare the sensitivity and specificity of FAB and IFS in mild cognitive impairment (multiple-domain amnestic MCI	Cognition	HC= 76.25, MCI =78.63
To examine the accuracy and validity of the Brian Health Assessment (BHA) in detecting cognitive impairment in a Cuban	Cognition	Controls:70.4 (5.9) Cognitively Impaired:MCI =72.7 (7.5), Dementia = 74.1 (5.9)
To validate the Ecuadorian Spanish-language version of the Parkinson’s Disease Quality of Life Questionnaire (PDQL-EV)	Quality of life	mn 69.4 years
To assess the validity of a Spanish version of the Geriatric Depression-15 Scale (GDS-15) in Ecuadorian adults.	Depression	≥ 65 years
To establish the factor structure of Euro-D across six Latin American countries (aim related to validation)	Depression	≥ 65 years
the creation of a healthy ageing index (HAI) based on the WHO conceptual framework in a subset of Latin American countries.	Health	≥ 65 years

To assess the Construct and/or Criterion Validity of the EURO-D in large population-based survey samples of	Depression	≥ 65 years
To investigate the measurement invariance of a set of self- and informant-report HRQL measures developed in the United	Quality of life	≥ 70 years mean
cross-national validation of the Scales for Out-comes in Parkinson’s Disease-PsychoSocial questionnaire (SCOPA-PS) infour Latin	Quality of life	≥ 60
Assess psychometric properties of the scale including acceptability, internal consistency, factor structre, covergent and	Psychosis	≥ 60
Examine whether the 12-item WHODAS II meets criteria for measurement invariance across cultures, assess factor structure and	Quality of life	≥ 65
Present normative data for the CSI-D 10 word list and animal fluency across sites of the 10/66 group	Cognition	≥ 65
Assess the validity of GMS/AGECAT organicity and depression diagnoses in 26 centres in India, China, Latin America and	Cognition and depression	≥ 65
To seek for psychometric evidence to support the suitability of Yesavage's short-scale questionnaire (GDS-15) as a suitable	Depression	≥ 60 years
Assess reliability and validity of the tool	Quality of life	≥ 60 years
Assess reliability and validity of the tool	Depression	≥ 65 years

Assess reliability and validity of the tool	Cognition	≥ 60 years
Assess reliability and validity of the tool	Cognition	≥ 65 years
To translate, adapt, and validate the Health Perceptions Questionnaire for use on an older Mexican popularion.	Health	≥ 60 years
To design an index of spirituality for elders in Mexico	Spirituality	≥ 60 years
To develop and assess psychometric properties	Mistreatment	≥ 60 years
To analysis the internal structure	Hopeless	≥ 60 years
This study aims to validate the WHOQOL-OLD in Mexico	Quality of life	≥ 60 years
The 11-items LSITA scale shows preliminary good properties of reliability and validity in Mexican elderly people. We suggest its	Life satisfaction	≥ 60 years
To establish its concurrent validity,	Cognition	≥ 60 years
To assess the validity in terms of sensitivity, specificity, and detection characteristics of frequently used cognitive	Cognition	≥ 65 years

To describe the cognitive instrument used in the Mexican Health and Aging Study (MHAS) in Mexican individuals aged 60 and	Cognition	≥ 60 years
validate the CES-D in mexican elders and determine the correct cut point	Depression	≥ 60
To assess the validity and reliability of EQ-5D in older adults with and without dementia in Mexico city.	Quality of life	≥ 60
To determine the psychometric qualities of the CES-DR and GDS scales in the elderly and compare them to clinical psychiatric	Depression	≥ 60
Evaluate the psychometric properties ofthe original Lasher and Faulkender scale, as well as presenting an adapted version for	Anxiety	mn age 63
Adapt the QUOL-AD to spanish and evaluate reliability and validity in Mexico	Quality of life	≥ 60
Evaluate psychometric properties of three religious coping measures	Spirituality	≥ 50
Revise and create an adapted version of the CES-D for mexico and evaluate its psychometric properties	Depression	≥ 60 (60-92)
To develop and carry out a psychometric validation of a Quality of Life Scale of 15 items with ten Likert-Type response from not satisfied	Quality of life	60 - 72 years
To determine the discriminant validity of the Norma Latina Battery in a group of Mexican individuals with AD and a	Cognition	mean = 77.7 (SD = 6.3)

This research has two main goals: the first is to assess the psychometric properties of the SF-36 Health Questionnaire	Quality of life	≥ 60 years
Validate the 5WT for the detection of MixD.	Cognition	≥ 60 years
To establish the cross-cultural adaptation, validity, and reliability of the ECog Mexican version (M-ECog) in participants	Cognition	≥ 60 years; CH=70.1±7.7, SCD=72.32±7.96, MCI=73.3±6.9, Dementia = 77.9±9.8
To examine the psychometric properties of the Spanish version of the 11-item DJGLS in Mexican older adults.	Loneliness	≥ 60 years
To evaluate the reliability and validity of the Geriatric Depression Scale in its 15-item version (GDS-15) in Mexican older adults.	Depression	60 and 94
To evaluate the psychometric properties of SF-36 in a sample of 1,915 community-dwelling Mexicans 60 years and	Quality of life	≥ 60 years
To analyze the factor structure, internal consistency and factorial invariance by sex of the Lasher and Faulkender	Anxiety	≥ 60 years
To examine the psychometric properties of Massachusetts General Hospital-Sexual	Sexuality	60 to 81 years
To assess the agreement and bias of three common tools used for screening of cognitive impairment in people with hypertension	Cognition	≥ 65 years
To translate the 5-item Coping with Humor Scale (CHS-5) from English to Spanish and examine the evidence of reliability and	cope (Humor)	mean 72.8 years

To examine the psychometric evidences of the Self-Efficacy Scale for Aging (EAEE)	Self-efficay	65-98 years
To determine and analyze the validity and reliability evidences of the swls scores in a sample of Peruvian not	Life satisfaction	mean 72.8 years
the study provides evidence of the validity and reliability of the BRCS in non-institutionalized older adults in Peru.	Resilience	mean 72.8 years
Evaluate the performance of clock drawing test-Manos versión (PDR-M) and Mini Mental State Examination	Cognition	≥ 65 years
To assess the validity and reliability of the PDR-M to discriminate between patients with dementia and healthy control	Cognition	≥ 65 years
to assess the performance of Memory Alteration Test (M@T) to discriminate controls, patients with amnestic	Cognition	≥ 65 years
To assess the psychometric properties of the Peruvian version of the Rowland Universal Dementia Assessment	Cognition	≥ 60 years
To determine the optimal cut of score for MMSE in this population given previous heterogeneity, considering higher and	Cognition	55-96
To compare the diagnostic accuracy of fototest with that of the MMSE	Cognition	60-89
To evaluate the invariance of the life satisfaction scale (SWLS) in two samples of older adults in Spain and Peru.	Life satisfaction	Mn 72.42

To examine the discrimination ability of the 10-point clock drawing test in identification of alzheimer's disease	Cognition	≥ 60
To evaluate performance of the peruvian money test in screening dementia	Cognition	≥ 60
Evaluate the performance of clock drawing test-Manos versión (PDR-M) and Mini Mental State Examination - Peruvian	Cognition	≥ 65 years
examine the performance of the M@T at discriminating between different clinical stages of AD in a cohort of persons	Cognition	mean 75 years
To evaluate the performance of the Peruvian version of the Rowland Universal Dementia Assessment	Cognition	≥ 60 years
Determine the level of concordance between the Mini-Mental State Examination (MMSE), Short Portable Mental	Cognition	≥ 60 years
To evaluate the diagnostic accuracy of brief soICo-cognitive tests that may differentiate bvFTD and AD patients with low	Cognition	≥ 50 years; AD, 72.21 (3.48), bvFTD=64.28 (5.44)
To estimate the psychometric properties of the neuropsychological assessment instrument - Neuropsi in patients	Cognition	M=69,18<br>SD=15,551
To evaluate the psychometric properties of the CAS in this group.	Coronaphobia	≥ 60 years
To evaluate the diagnostic accuracy of three brief cognitive screening (BCS) tool	Cognition	≥ 55 years

To translate into Spanish and evaluate the psychometric evidence of the Impact on Quality of Life (COV19-QoL) applied	Quality of life	≥ 60 years
To standardize and evaluate the psychometric aspects of the battery in the Evaluation of Semantic Memory in Alzheimer's	Cognition	58 to 95 years
To evaluate the psychometric properties of the DJGLS in Peruvian older adults. SpeICfically, factorial validity,internal	Loneliness	61 to 91 years
To evaluate the performance of the ADCS-ADL in cognitively healthy controls and a population of Peruvian patients with a	Cognition	≥ 60 years
To validate the psychometric properties of the FCSRT-Picture version among Urban illiterate individuals in Callao, a	Cognition	≥ 50 years; Mean age= 70.2 ± 3.8 years
To evaluate the psychometric properties of the FCV-19S for use in older adults. SpeICfically, the evidence of validity	Fear	≥ 60 years
To translate the WTLS into Spanish, assess its internal structure, reliability, and the correlates between WTLS and life satisfaction,	Will to live	≥ 60 years
Evaluate the evidence of validity, based on internal structure, convergent and discriminant validity, reliability, and factorial	Minfulness	≥ 60 years
To analize the measurement properties of the WHOQOL-OLD questionnaire for Peruvian institutionalized seniors.	Quality of life	≥ 65 years
to evaluate the factorial invariance of the BRCS in samples of older adults in Peru and Spain, using multigroup Confirmatory	Resilience	≥ 60 years

This study replicated the work of BermúdezLlusá et al. (2019) to determine the reproduICbility and generalization of those	Cognition	59–92 years
To determine the psychometric properties: validity and reliability, of the Scale of Happiness from Lima	Happiness	≥ 60 years
to explore the diagnostic validity of the MiniMental Status Examination and the Clock Drawing Test, and a composite score of both	Cognition	≥ 40 years, Overal mean = 60.53

setting	Inclusion/exclusion	N	Interrater Reliability
Senior center	N/I	n = 60	N/I
Hospital and neurologcal institute	Exclusion: Psychiatric diseases and other neurological diseases.	n = 60 (control n = 19; AD n = 18; MCI=25)	N/I
Social clubs or community-dwelling	neuropsychological assessment, neurologic and psychi- atric evaluation, and imaging.	n = 59 (control=36; Alzheimer´s=23)	N/I
Primary care clinics	Inclusion: Known dementia and subtype, depression on GDS15, neurological disorders, motor or sensory disorder, head	n = 150; 100 controls, 50 cases with dementia	N/I
HIBA-HMO italian hospital of buenos aires	Illiterate caregivers excluded	n = 87 patient caregiver dyads; test-retest n = 15	N/I
Center for Aging and Memory Research of the Hospital General Dr Abel Zubizarreta	monolingual spanish speakers	n = 88 (46 NC; 42 MCI)	N/I
university clinic, Arg-ADNI cohort	as per ADNI, informant available. meeting MCI, mild AD criteria. Controls MMSE 24-30, CDR0, no memory complaints WMS-	n = 56 (15 controls, 28 MCI, 13 mild AD)	N/I
N/I	exclusions: stroke, Parkinson's disease, Geriatric Depression Scale (GDS) ≥ 5, HIV/AIDS, reversible dementia,	n = 30 controls, 61 amnestic MCI, 56 mild AD	N/I
N/I	Inclusion: ≥ 60 years, ambulatory, hearing and sight adequate for test performance	n = 76	N/I

Private practice	Exclusion: psychiatric, neurological diseases. Current consumption of antidepressive or anxiety medication.	n = 75 (n = 31 AD; n = 44 control)	N/I
Medical center and community	90 Spanish-speaking participants aged between 60 and 85 years (40 controls and 50 patients diagnosed with MCI).	90	N/I
community	Inclusion Criteria: 55-85 years. Cognitively healthy individuals. Native Argentine Spanish	Pilot 1= 20, pilot 2=31	N/I
Community	Exclusion criteria: a) history of at least one of the following medical conditions: cognitive impairment or dementia,	269	N/I
Medical center	N/I	54	N/I
Community	Inclusion Criteria: ≥ 18 years, Argentine. Exclusion Criteria: Uncompensated hearing loss (no hearing aids).Not	210	N/I
Outpatient care setting between September 2017 and May 2018.	Patients with < 3 years of education, sensory distUrbances, psychiatric disorders, or moderate-severe dementia were	399	K = coeffiICent: 0.69 (95% IC 0.54-0.69).
healthcare institutions	Inclusion criteria: Aged between 60 and 93 years.	696	N/I
different welfare institutions	inclusion criteria: a) absence of psychiatric disorders, neurological diseases, intellectual disability, motor or sensory	504	N/I
Community	Exclusion Criteria: Incomplete tests (did not complete the measurement instruments) Non-Argentinian nationals.	203	N/I

Cognitive Neurology in Argentina and the Cognitive Neurology and Dementias Unit of the Neurology Service-Hospital	The diagnosis of ATD was made according to the NINCDS-ADRDA criteria23 and that of bv-FTD according to the criteria	70 = Alzheimer disease, 31 = behavioural variant frontotemporal dementia, 139 = a	N/I
Day Hospital	Patients that had had an acute event or exacerbation of chronic disease.	n = 534 (53 for Interrater Reliability)	ICC = 0.92 (IC=0.87-0.95) p < 0.05; k = 0.62 - 0.87 (p < 0.05)
Community	Inclusion: chronic pain lasting six months or longer, and with pain intensity greater than or equal to 3, according to a	n = 48	Intra:92%; Inter: 98.5%
N/I	Exclusion: absence of an informant that lived together with the patient, lack of fluency in Portuguese, untreated	n = 129 (n = 89 carers alzheimer´s patients; n = 40 realiteves of control group of elderly)	N/I
homes of the elderly	N/I	n = 903	N/I
Open university for seniors	Inclusion: Incription of the elderly in the 1o se-mestre de 2010 en Universidade Aberta à Terceira Ida-	n = 153	N/I
University hospital (outpatients)	Control: no neurological decline or diseases; AD1: moderate loss memory, rating score 1 (CDR); AD2: severe memory loss, rating	n = 128 (control n = 85; AD1 n = 31; AD2=12)	N/I
Nursing home	Inclusion: Diasnosed with dementia. Exclusion: Patients who had no formal or informal caregiver to take	n = 50	85.2% inter-rater
Community-based elderly	N/I	n = 91 (control and mild/moderate dementia groups)	N/I
Public Hospital	Exclusion: Severe visual or hearing impairment or marked psychomotor disability (e.g. parkinsonism); a history of	n = 10	ICC = 0.954 (P < 0.001 for a confidence interval (IC) of 95%=[0.932;

N/I	Exclusion = low visual or auditory acuity, motor or rheumatic distUrbance, chronic alcoholism, cardiovascular disease,	n = 232 (n = 28 Illiterate;n = 119 1-4 schooling years; n = 85 5-8 schooling years)	N/I
N/I	Exclusion: low visual or auditory acuity, motor or rheumatic distUrbance, chronic alcoholism, cardiovascular disease,	n = 253 (n = 28 Illiterate; n = 119 1-4 schooling years; n = 85 5-8 schooling; n = 21 ≥ 8 scholling	N/I
homes of the elderly	Exclusion: Severe disabilities.	n = 59	ICC = 0.81, 95%IC=0.72–0.88.
Catchment area of the Hospital	Exclusion: history of deafness, complaint of hearing impairment, positive whispered-voice screening test and MMSE	n = 133 (n = 66 AD patients and n = 67 control)	N/I
Hospitals and community meeting places	Inclusion: Mini-Mental State Examination (MMSE) scores (S) equal to or higher than median values for persons of similar	n = 82 (n = 41 control; n = 41 Alzheimers´disease)	N/I
Hospital and seniors groups	Inlcusion control: no cognitive decline, neurological or psychiatric history.	n = 31 patients AD; n = 62 control.	N/I
N/I	Inlcusion control: no cognitive decline, neurological or psychiatric history. No illiterate	n = 21	N/I
N/I	Inclusion: preserved cognitive and communication skills, being a resident in the partiICpating ICties, and	n = 617	N/I
Social and occupational institutions	Inlcusion: agea and being interested in partiICpate.	n total=3020; n older sample=289	N/I
Health center or community	Exclusion: if they had no informant, presented visual or auditory defiICts precluding performance of the cognitive assess-	n = 123 (n = 42 control, n = 40 MCI, n = 41 with mild dementia)	N/I

Center for Elderly	Inclusion: age, suffiICent speech, hearing, and cognitive abilities (considering their MEEM performance).	n = 151	K= 0.97 ($p < 0.001$)
N/I	exclusion: neurological or psychiatric disordes and physical limitations or hearing impairment that precluded cognitive testing.	n = 135 (n = 60 control and n = 75 AD)	N/I
Primary health care program	Exclusion: cognitive impairment, drug abuse, psychiatric or physical illness	n = 43	N/I
N/I	Exclusion: substance abuse, psychiatric syndrome, depression.	n = 343 (AD = 121; VD = 113; questionable dementia = 61; healthy elderly = 48).	CDR global score 85%
Long-term care institution	N/I	n = 58	N/I
community-dwelling.	Exclusion: Cognitive impairment (MMSE).	n = 337	N/I
Geriatric speICalized center for medical consultations or rehabilitation.	Exclusion: Cognitive impairment, with mobility and orthostatic disabilities, and severe hearing or visual impairment.	n = 350	N/I
N/I	Exclusion: severe dementia and those with evidence of neurological or psychiatric comorbidities. Iliterate patients.	n = 184	N/I
outpatient clinic	Illiterate subjects and those younger than 60 years of age (except those with presenile AD)	n = 51 (mild/moderate: n = 16, MCI: n = 10, no-AD: n = 8, control: n = 17)	N/I
University hospital, nursing homes, and community.	Clinical ability to understand and answer the instruments	n = 424	N/I

Tertiary referral hospital	N/I	n = 147	N/I
N/I	Exclusion: Physical disabilities	n = 202	intra: k = 0.88; inter: K = 0.74
Community center, Family centers, retiree associations	Inclusion: Non-institutionalized older adult individuals with no cognitive impairment	n = 337	N/I
Geriatric outpatient service	Inclusion: Males who completed all the assesmente process. Exclusion: Severe dementia, physical	n = 122	N/I
Long-term care institution	N/I	n = 58	N/I
Tertiary hospital	N/I	n = 248	N/I
outpatient services of a public university hospital	Without Cognitive impairment (WIC), cognitively impaired not demented (ICND), dementia	n = 243 (WIC n = 202; ICND n = 22; Dementia n = 21)	N/I
homes of the elderly	Inclusion: age ≥ 60 years; resident in the munilCpality of São Carlos; (3) cut-off on the Mini-Mental State Exam (MMSE)	n = 111	N/I
Public geriatric outpatient service	Exclusion: age, neurological or psychiatric disorder, sensory impairment and over 4 years of education	n = 180	N/I
day center, community dwelling older adults and hospital	N/I	n = 202 (n = 987 day center; n = 65 community dwelling; n = 40 hospital)	N/I

Day centers or medical centers	inlusion: age, good cognitive and functional capacity	n = 160	Dream distUrbance: K = 0.984; depresivve humor: K =
Community dwelling older people	N/I	n = 87	N/I
N/I	N/I	N/I	N/I
Outpatient unit of Universidade do Estado do Rio de Janeiro Hospital.	Inclusion: age and preserved hearing and comprehension. Exclusion: advanced stage of cognitive	n = 211	ICC = 0.944 (Manos method)
clients of a Brazilian pri-vate health plan,	Exclusion: psychiatric disease, severe neurocognitive disorders, or severe motor disability after	n = 417	N/I
outpatient primary care clinic	Exclusion: Severe eye or hearing impairment or advanced cognitive disorders and/or mental diseases impairing test	n = 303	N/I
N/I	N/I	n = 76	N/I
Outpatient unit	N/I	n = 211	N/I
Outpatient unit	Inclusion: Diagnse of depression (ICD-10)	n = 51	GDS-15: rho = 0.86, p < 0.001; z = 1.60, p = 0.109; GDS-10: rho = 0.81, p <
Mental Health Unit	Inclusion: criteria for depressive disorder. Exclusion: severe sensory impairment, aphasia or Mini-Mental State score <	n = 64	N/I

Assisted by Family Health Strategy (FHS),	Exclusion: incapable of communicating, indigenous living in tribal villages and those who refused to partiICpate or sign the	n = 503	N/I
Geriatric memory clinic	Inclusion: age, availability of<br>a knowledgeable relative or close friend who had regular contact	n = 132 (n = 72 control; n = 33 MCI; n = 27 mild dementia)	ICC = 0.96 (IC 95% 0.92 to 0.99),
Community-dwelling outpatients	Exclusion: no history of neurologic or psychiatric diseases and were not taking any drugs with effects on the central	n = 597 (465 ≥ 60 years)	N/I
Geriatric Clinic	Exclusion: severe cognitive decline (CDR 3), major depression, any plegia or paresis, important tremor, functional impairment in both hands, severe visual	n = 321 (113 AD; 208 control)	N/I
community-dwelling outpatients	Exclusion: severe cognitive decline (CDR 3), major depression, any plegia or paresis, important tremor, functional impairment in	n = 220 (121 AD; 99 control)	N/I
Community	Inclusion: hear and understand well enough to be able to take part in an interview and signing the informed consent form.	n = 353	N/I
Integrated Group of Elderly (senior´s recreation group)	Exclusion: a) hearing impairment that compromised communication; b) visual incapacity that could	n = 98	ICC-BREF = 0.94 (IC 95% 0.90; 0.97); ICC-OLD= 0.92 (IC 95%0.87; 0.96)
Institute of gerontology	Inclusion: 0-4 years of eduaction, performance of the MMSE; uncorrected auditory<br>and/or visual defiICts;	n = 164 (60 lliterate, 52 1–2 years of education, 52 3–4 years).	N/I
Primary care center	N/I	n = 220	N/I
community-dwelling	Inlcusion: literate and physical assessed as being in good health	n = 278	N/I

N/I	N/I	n = 1558	N/I
N/I	N/I	n = 1558	N/I
N/I	N/I	n = 1558	N/I
Geriatrics and Gerontology Ambulatory in Jundiaí Medical School,	Inclusion: age and two years of school.	n = 64 (control = 22; AD = 14; OCD = 8; Paraphrenia = 20)	N/I
Geriatrics and Gerontology Ambulatory in Jundiaí Medical School,	Inclusion: age and one year of school.	n = 426	N/I
community-dwelling elderly	Exclusion: moderate and severe cognitive decline, major depression, any limb plegia or paresis, important tremor,	n = 136 (control = 39, MCI = 44, AD = 53).	N/I
Institute of Geriatrics and Gerontology	Exclusion: illiterate	n = 136	N/I
Nursing homes, older adults community groups, and a university hospital.	Exclusion: clinical inability to understand the instrument	n = 424	N/I
Nursing homes, older adults community groups, and a university hospital.	Inclusion: age 60 or above and clinical ability to understand and respond to the instruments administered.	n = 424	N/I
Nursing homes, older adults community groups, and a university hospital.	Inclusion: to understand the instruments applied, as assessed clinically.	n = 424	N/I

Psychogeriatric clinic	Inclusion: MCI diagnosis according to the Petersen criteria. Exclusion: illiteracy, dementia, depression, visual or	n = 58	Intra-rater: Difficulties ICC = 0.78, p < 0.001; Performance:
community	Exclusion: impaired mobility, vision or hearing defiICts, and those who did not complete all of the assessments.	n = 95 (MCI = 32; AD = 33; control = 30)	N/I
Community-dwelling elderly	N/I	n = 109 (dyad)	K = 0.889
N/I	Inclusion: dementia criteria	n = 269	K = = 0.73
Neuropsychology Unit	Exclusion: History of other neurological or psychiatric diseases, drug or alcohol addiction, and prior exposition to neurotoxic	n = 40 (control = 20, AD = 20)	N/I
Psychogeriatric clinic and community center	Exclusion: Cognitive impairment	n = 72	N/I
Community sources (community centers, newspaper, and radio ads)	Inclusion: ≥ 4 years of formal education, no important tremor, psychiatric illness such as schizophrenia, bipolar	n = 112 (Alzheimer's disease = 28; MCI = 43; normal controls = 41)	ICC 0.75 (p < 0.001)
Institute of Psychiatry	Inclusion: no descompensated disease, at least 4 years of education, important tremor, psychiatric illness	n = 97 (normal controls = 41; MCI = 35; AD = 21)	N/I
Neurology clinic and Referral Center for Cognitive Disorders	Inclusion: age	n = 84 (MCI = 36; AD = 23; control = 25)	N/I
sample from longitudinal study	N/I	n = 156 (MMSE < 26 n = 108; MMSE ≥ 26 n = 46)	N/I

Living for the Elderly Program	Exclusion: cognitive deficits as per the evaluation on the Mini Mental State Exam, some difficulty in communication, or were	n = 137	N/I
Bambui study	Exclusion: unable to complete the interview reliably or had a Mini-mental State Examination (MMSE) score	n = 392 (after second phase)	N/I
Community (controls) AND geriatric clinic (patients)	Patients with another neurological or psychiatric illness or severe cognitive impairment	n = 33 (patients) 350 normative controls	N/I
university hospital neurology clinic	≥ 60, presence of family caregiver (≥ 24h week contact), no serious language impairment	60 patient and carer dyads (30 = mild, 30 = moderate AD)	N/I
Public geriatric clinic	≥ 60 years MMSE ≥ 14, informant available, absence of sensiry, motor or other uncompensated conditions, delirium	n = 189	N/I
Public geriatric clinic	≥ 60 years MMSE ≥ 14, informant available, absence of sensiry, motor or other uncompensated conditions, delirium	n = 123 (IRR n = 60)	ICC 0.98
public hospital internal mediICne OPD	Exclusion: uncorrected hearing impairments, cognitive disorders and/or mental diseases at advanced stages	n = 302.	N/I
4 sites stratified for funcional capacity. Geriatric OPD, Sports Centre Group, Elderly Health Management	Exclusion: moderate/severe dementia, expression/comprehension aphasia/other disease	n = 193, convenience and quotas stratified for predicted dependence.	N/I
Secondary/tertiary referral centre OPD	Exclusion: severe sensory/motor impairment, psychotic symptoms, without caregivers	n = 178: (85 = amnestic MCI, 93 = mild probable AD NINCDS-ADRDA)	N/I
Community-dwelling healthy elders	Inclusion: Age ≥ 60, > 4 yrs school, MMSE ≥ 19, GDS total < 6, normal Katz/Lawton ADL/IADL score	n = 126	N/I

University hospital	N/I	n = 49	N/I
University hospital psychiatry and geriatric clinics	Inclusion: age, diagnosis of mild/moderate dementia; exclusion: other psychiatric disorder, severe dementia DSMIIIR, no	n = 34 Mild/mod dementia, 58 controls.	N/I
University hospital cardiology clinic	Inclusion: Objective coronary artery disease Exclusion mental or physical condition preventing completion of	n = 209	N/I
Elder care centres	Inclusion: Cognitively normal, no neuropsychiatric disease. Exclusion: unstable systeMCI diseases that	n = 110	N/I
Outpatient clinic	Inclusion: 4 years education or less. No serious visual, hearing impairment psychiatric disorder, disorder affecting	n = 306; 211 analysed (4 years education or less).	N/I
community (part of ageing study)	Inclusion: community resident aged 60-90, autonomous and independent in ADL and no health problems	n = 90	N/I
community (part of SPAH study)	Included those recruited for community study. Exclusions unable to answer, refusal, demmentia severe sensory	n = 1933	N/I
Geriatric outpatient clinic of university hospital	Accompanying carers of patients of the OPD who met this criteria: brazil nationality, ≥ 65 years, attending clinic and last	n = 169 informants (97 for retest)	N/I
University hospital OPD	Exclusion: MMSE 19 of less than 14, current psychiatric illnessand/or severe sensory deficits. Informants with a history	n = 169	N/I
community SPAH study	excluded: unable to complete questionnaire due to cognitive difficulties or incomplete data	n = 2072	N/I

university clinic	controls: ≥ 50 free of neurological or psychiatric disease and no drugs interfering with psychiatric evaluation. Cases mild	n = 96 control; 44 AD patients	N/I
Primary care clinics	aged ≥ 60; used primary health care in the district; consented; and completed the questionnaire. Exclusion: severe cognitive	n = 391, proportionately stratified sample	N/I
Primary care clinics	aged ≥ 60; used primary health care in the district; consented; and completed the questionnaire. Exclusion: severe cognitive	n = 466	N/I
Primary care clinics	Exclusions: Severe visual/auditory defiICts, advanced dementia, neurological/psychiatric syndromes (except	n = 55	N/I
specialist public and private clinics	≥ 1 years schooling, no visual/auditory deficits or other condition preventing participation. Exclusion GDS10+	n = 91 (28 AD 63 controls)	K = = 1 (sig p < 0.0001)
memory clinic	8 years education or more, attending memory clinic	n = 157	30 dementia, 65 MCI, and 62 controls
community	≥ 60 without cognitive or sensory impairment	n = 196 divided into 10 focus groups	N/I
geriatric outpatient clinic of university hospital	Inclusion: attending OPD for mental health service. Exclusion: informant present bipolar disorder, alcohol abuse, epilepsy,	n = 95 (convenience) DSM-IV dementia 71/95 DSM-IV depression 30/95	N/I
community study nested within the FIBRA study	not stated. As per FIBRA and consented. ≥ 80 years	n = 187	N/I
behavioural neurology service of university clinic	exclusion: GDS15 5+, Hachinski 4+, CVD on neuroimaging, excess alcohol, age < 65, schooling < 2 years,	n = 10 Controls, n = 11 AD	personal semantics 0.99 and autobiographica l events 0.99

N/I	cognitively normal, ≥ 60. Exclusions: psychiatric, neurological and/or medical condition affecting cognition HADS 9+,	n = 30	N/I
community (within sao paulo ageing and health study)	Inclusion: within Sao paulo ageing and health study ALSO sought healthcare in past 3 months and able to answer questions	n = 32	ICC total needs 0.99 (0.99-1.00) participants 0.99 (0.97-0.99) informants.
University geriatric medilCne clinic	Dementia (ICD-10/DSM-III-R), attendees of the clinic. Exclusions delirium, severe mental illness, drug or alcohol dependence)	n = 20 md age 75.5, md education (pilot n = 5)	K = 1 (DSM-III-R) K = 0.87 ICD10 AD, (1 other subtypes).
Psychogeriatric clinic of university hospital	N/I	n = 195 (NC = 78, MCI = 76, AD = 41)	N/I
community residents recruited from door to door survey and nursing home residents	N/I	n = 403 (68-93% had religious beliefs)	N/I
Primary care clinics	primary healthcare users aged ≥ 60 years, able to answer the entire instrument. Exclusions - refusal	n = 357	N/I
Hospital neurology clinic	Inclusion: PD, attending the neurological deaprtment, absence of other neurological or swalling disorder. MMSE ≥	n = 50 (development) n = 147 pts 47 controls (pilot), test-retest n = 44	N/I
University hospital clinics	Inclusion: current diagnosis HF, ≥ 3 months follow up, alert, able to communicate, oriented. Individuals undergoing	n = 170	N/I
public primary care unit	Exclusions: incomplete data. None others stated	n = 317	N/I
three geriatric outpatient units	older person unable to communicate, refused consent, MMSE 10 or less	n = 507 older person and carer dyads	K = = 0.77 (0.685-0.583)

geriatric clinic of university hospital or community, from FIBRA study	inc: clinic - brazilian citizen, ≥ 65 years, informant available ≥ 23 , comprehensive geriatric assessment (CGA) in last	n = 652 older persons and informant dyads (308 geriatric cognitive clinic	N/I
community convenience sample. Hospital staff, community residents, orthopaedic patients, patient caretakers	no psychiatric disorder and functioning well in local community. Those aged ≥ 60 had MMSE score normal for age and	n = 201	N/I
community sample within SABE study	Not stated, as per SABE study	n = 1413	N/I
Older people in hospital, day centre attendees, institutionalised people, receiving treatment for DSM-IV depression,	none listed, comorbid conditions accepted	n = 311 mn age 70.6% (subsamples n = 40, 97, 64, 48, 62)	N/I
Institute of psychiatry, geriatric services	Exclusion: presence of other psychiatric disorders observed duringthe clinical evaluation or absence of informants capable	n = 92 (n = 33 MMD and n = 59 control)	N/I
Geriatric center	Inclusion: at least 5 years of education	n = 119	N/I
community	Exclusion: only those who did not have informants to help answer the questionnaires.	n = 623	N/I
N/I	Exclusion: severe dementia	n = 630 (n = 385 control, n = 110 dementia, n = 135 impairment)	N/I
Community	N/I	n = 40	NHP = 0.99; SF-36 = 0.96
Medical center	Inclusion: at least 4 years of education	n = 229 (n = 110 control; n = 88 MCI; n = 31 AD)	ICC 0.96, (95% IC 0.94–0.98; $p < 0.001$)

Outpatient services Clinics School	Exclusion: acute pain, with visual and/or hearing defiICts and those with cognitive impairment assessed by Mini-mental	n = 68	N/I
N/I	N/I	n = 30	N/I
Medical center	Inclusion Criteria: CG: Independent ADLs, no neuropsychiatric disorders, no subjective memory complaints, no MCI.	246	N/I
Medical center	The main exclusion criteria were neurological diseases such as a history of stroke, traumatic brain injury, epilepsy, multiple sclerosis,	87 MD 49 MCI 29 HC	N/I
10 Health Strategy Family units	(i) be 60 years old or older; (ii) reside in the community; and (iii) be able to independently perform activities of daily	175	N/I
Medical center	N/I	70, bvFTD (n = 31) PPA (n = 12) AD (n = 27)	N/I
Family Health Strategy (ESF) units	Inclusion criteria: ≥ 60 years Preserved cognitive ability: Assessed using the Mini Mental State Examination	136	N/I
Community	Inclusion Criteria: ≥ 60 years Fluent in Portuguese. No uncorrected visual or perceptual impairments.	38	The inter-translator agreement rate was 84.4% (40 items) between
Community	Inclusion Criteria: Age: ≥ 60. Volunteers: Elderly individuals who volunteered to participate.	468	N/I
N/I	≥ 60 years, ≥ 1 years of schooling, consenting to take part in the study voluntarily and signing the informed consent form	285	N/I

Basic Health Units (BHU)	Inclusion Criteria: ≥ 60 years Literate. Followed by health teams from the respective health	75	N/I
Hospital	≥ 60 years; residents of the city of Rio de Janeiro; with a clinical diagnosis of AD and HE without a diagnosis of mental illness.	136	N/I
physical therapy outpatient clinic	≥ 50 years; diagnosis of idiopathic PD using the UK Brain Bank criteria; Brazilian nationality; rated stage I-IV on Hoehn and	90	High agreement between observers
Hospital	1) ≥ 45 years 2) to read and write 3) to read and understand a text in Portuguese 4) to have a next of kin.	72	N/I
Community and institutional	Inclusion criteria: Aged ≥ 60, institutionalized or non-institutionalized, and had internet access for video call data collection.	700	N/I
community and the Clinical Hospital of the Universidade Federal de Minas Gerais.	Belo Horizonte, with capacity to inform consent and to answer the questionnaire. Older adults with diagnosis of dementia	187	ICC was used, showing virtually absolute reliability
Hospital and community	Inclusion criteria: ≥ 60 years. Location: Hospitals (Paraíba). Primary health care unit	481	N/I
Geriatrics outpatient clinic and a long-term care facility (LTCF)	Inclusion Criteria: Dementia (any cause) diagnosed according to DSM-V. Impaired verbal	50	ICC of 0.970 ($p < 0.001$)
outpatient clinics at the Cognitive and Behavioral Neurology Units of two hospitals in Brazil: the Federal University of Minas	Inclusion criteria: Patients aged ≥ 45 with probable AD or probable bvFTD, and ≥ 4 years of education.	102	N/I
Community	Inclusion Criteria: Adults ≥ 60 years, community-recruited. Exclusion Criteria: History of	345	N/I

Urban	Inclusion criteria: ability to express oneself in BP, residing in the Urban area of the muniICpality, and meeting the MMSE score	384	N/I
N/I	60 years or over, literate and followed by the health teams of the respective BHU, from March 2017 to June 2018. Health unit	73	N/I
Social centers, residents' associations, and institutions for the elderly in the PB metropolitan area	Inclusion: ≥ 60 years, both sexes, with at least 1 year of formal education, fromsocial centers, residents' associations, and	256	N/I
Community	Exclusion criteria: Memory, attention, orientation, or communication issues suggestive of dementia.	2,734	N/I
University hospital	Exclusion: severe aphasia, coma and inability to partiICpate in cognitive assessments.	n = 374 (development cohort); n = 104 (Validation cohort)	N/I
Elderly Programs	N/I	n = 1174	N/I
Community or institutions	N/I	n = 168 (control = 90; mild = 28; moderate = 30; severe = 20)	N/I
N/I	N/I	n = 777	N/I
N/I	N/I	n = 750	N/I
N/I	N/I	n = 777	N/I

A geriatric outpatient clinic of a university teaching hospital.	Exclusion: Dementia diagnostic	n = 110	N/I
Hospital and primary care centers	Exclusion: sensory defiICt.	n = 60 (n = 30 dementia patients; n = 30 control)	N/I
Primary care center	Inlusion: Answering all the questions, have a caregiver, consent of the caregiver and the patient	n = 54	N/I
Community dwelling older adults	No exclusion criteria were considered.	n = 2143	N/I
Recreational centers of older adults	Inclusion: age	n = 301	N/I
Older persons clubs (convenience sample)	None listed	n = 30 (linguistic pilot) n = 300 (main study)	N/I
cognitive and neurological clinic, convenience sample	illiteracy, debilitating cognitive impairment that could interfere withneuropsychological assessment, underlying	74 (30 controls, 30 dementia, 14 MCI)	N/I
hospital neurology clinic, and available controls	age ≥ 60 chilean, > 4 years education	60 = dementia, 22 = MCI, 45 = controls	N/I
Primary care clinics	Inclusion: Normal cognition or MCI (SPMSQ), physically able to take part, consenting.	n = 87	N/I
community Urban and rural, nested in another community study	N/I	n = 94, (18 dementia) 50/94 < 6 years school	N/I

community (attending public services and non government organisations for elders)	consent. Otherwise not stated	n = 804 mn age aged 70 ± 6 years	N/I
Community	≥ 60 years participants should have no cognitive impairment and should reside in rural areas of the Arica and	n = 800	N/I
community and Cognitive Neurology and Dementia Unit, Hospital del Salvador, Santiago, Chile.	Healthy Controls (HC): Inclusion: Minimal writing ability, MMSE ≥ 24/30, Geriatric Depression Scale < 5, Zung Anxiety Scale <	344 HC, 156 DS	N/I
Community and Hsopital	Inclusion Criteria: Healthy Elderly (HE): No neurological conditions affecting cognitive performance.	226, NHE = 113, Nmild NCD = 65, Nmajor NCD = 48	N/I
Community	≥ 60 years, not having cognitive impairment and being resident in rural areas.	800, non-indigenous (231), indigenous Aymara (201) and Mapuche (368)	N/I
community	Inclusion criteria: ≥ 60 years old, residing in the city of Antofagasta. Exclusion criteria: being institutionalized and having	202	N/I
Elderly Long-Term Care Facilities (ELEAM)	Inclusion criterion: residents of an ELEAM. Exclusion criteria: health conditions (discomfort, pain, acute or	106	N/I
Community	Exclusion criteria: 1) having a diagnosis of dementia; 2) having had a stroke in the last 5 years; 3) having had a major depressive	203	N/I
Medical Center	Inclusion Criteria: Age: ≥ 65 years. EMPAM, rural Chile. Self-suffiICent or autonomous (EFAM).	241: 111 MCI and 130 controls	N/I
medical center	Inclusion criteria: Aged between 65 and 80 years. Self-dependent.	582	N/I

Elderly clubs and Programa Vínculos beneficiaries, a national policy by SENAMA (National Senior citizens Service) in Chile	Inclusion Criteria: ≥ 60 years 0-12 years of schooling. Functional hearing and vision (or use of	135	N/I
HOA group: Community centers and government programs for healthy older adults. Mild and Major NCD	Inclusion criteria: HOA: Cognitively healthy (EMPAM). Mild NCD: Cognitive changes, no functional	226; 113 healthy older adults (HOA), 65 mild neurocognitive disorder (NCD) and	N/I
Community	Inclusion Criteria: Older adults (≥ 60 years old). Autonomous (43+ points on the EFAM A exam,	399	N/I
Community	Inclusion: Individuals aged, residing in the community, with complete SF-36 responses. Exclusion: 642 individuals	4,124	N/I
community	Inclusion Criteria: Older adults (age unspeICfied) Indigenous and non-Indigenous individuals	800 [Non-indigenous (n = 231) Aymara (n = 201) Mapuche (n = 368)]	N/I
Medical center and community	N/I	1164	N/I
Community	Inclusion Criteria: Older adults living in the community, without severe health or cognitive impairments that would	249	N/I
Rural	Being aged 60 years or over, not having cognitive impairment and being resident in rural areas.	Total = 800, non-indigenous (231), indigenous Aymara (201) and Mapuche (368)	N/I
National Senior Adult Service and senior groups. and CESFAM (Primary Health Care Centers)	Inclusion criteria: General population: Aged ≥ 60, literate, no medical conditions affecting cognitive performance.	1164	N/I
Hospital	N/I	N/I	N/I

community	≥ 60, without physical or mental disabilities	n = 756 Chile; 817 Ecuador	N/I
Day centers	Inclusion: age and setting	n = 500	N/I
Primary attention, community dwelling older people	Exclusion: Cognitive decline, sensorial or motor problems.	n = 104	N/I
Community centers	Exclusion: Alzheimers´disease, Parkinson, cerebrovascular disease, or any auditory or cognitive impairment.	n = 185	N/I
Healthcare institutions or nursing homes,	Exclusion: Visual or hearing impairments, history of psychiatric illness, chronic substance abuse, neurological disorders,	n = 213 (n = 75, clinical samples; n = 138, non-clinical)	N/I
Senior center	Inlusion: without cognitive impairment and not institutionalized	n = 180	N/I
Hospital and community	Inclusion: Clinical diagnostic criteria for MD AND MCI.	n = 193 (patients=109; control=84)	ccc=0.093 ICC 95 [0.78, 1.00].
Social community associations	Exclusion: Severe disabilities (impossibility to perform) and people who had four or more errors in the orientation section of	n = 150	N/I
Community centers for older people	N/I	n = 105	N/I
community centers of older adults	Exclusion: physical, cognitive or sensorial impairment.	n = 383	N/I

Life Center	Exclusion: Cognitive impairment	n = 130	N/I
community	Inclusion: not resident in care facility	n = 4034	(NB other reliability completed in rasch model)
older persons day centres	N/I	n = 510	N/I
community sweep technique study	consent to partilCpate	51-60: n = 237; ≥ 60 n = 217	N/I
Medical center	N/I	n = 180	K = = 0.71
community	Inclusion Criteria: ≥ 60 years. Subjects who followed the stages of the study protocol, including a clinical interview and	314 , 235 MCI, 79 cognitively healthy	N/I
Neurosciences Group at the University of Antioquia.	Inclusion: ≥ 18 years old, MCI diagnosis per DSM-5, mild and moderate stages per CDR, able to read and write, and informant ≥ 18	189	N/I
Bolivariana University Clinic	Exclusion criteria were refusal to participate, stupor or coma at the assessment period, or severe language or	200	N/I
Foundation Valle del Lili University Hospital	Inclusion Criteria: Diagnosis of Parkinson's disease based on the UK Parkinson's Disease SoICety Brain Bank	100	N/I
SABE study (Health, Well-being, and Aging)	Older adults aged 60-98 from Bogotá.	1,957	N/I

Hospital	Inclusion criteria: patients had to be assessed during the first 12–48 hours following hospitalization. Exclusion	200	ICC = 0.873 (95% IC: 0.832–0.904) for physiICan administration
community (within Epidemiology and the Development of Alzheimer's Disease in Costa Rica (EDAD) study	MMSE ≥ 23, normal/corrected vision and hearing, adherence to medication. Exclusions major psychiatric disorder,	n = 250 mn age 68.28 (rural n = 71, Urban 168) mn ed 14.18 y (Urban) 10.7 (rural)	N/I
association of Pensioned Educators (ADEP), Comprehensive Programme for the Elderly in the Western Region	Inclusion Criteria: Cognitive capacity: Functional abilities. participation: ADEP: Monthly meetings.	pilot (N = 40), and another for the analysis of the psychometric properties (N = 100).	N/I
Nursing homes and day care centers	Inlusion: Clinical Dementia Rating Scale [CDR] score equal to 0.5 for MCI and CDr = 0 for control group.	n = 129 (n = 92 control; n = 37 MCI)	N/I
Community	Healthy Control Group Psychiatric history: Excluded if any psychiatric disorders were reported. Severe sensory defiICts:	89, HC (n = 59) MCI (n = 30)	N/I
medical center	Inclusion Criteria: Individuals confirmed as cognitively healthy after diagnostic validation. MCI or dementia diagnosis:	146 participants (cognitively healthy=53, MCI=46, dementia = 47)	N/I
neurology movements disorders clinic	inclusion: PD. Exclusion illiteracy, neurological, functional defiICts due to non PD cause serious concurrent pathology, or	n = 137 with PD n = 34 depression, n = 36 anxiety	N/I
in and outpatients from a geriatric health institution	Inclusion criteria: Subjects with diagnosis of dementia, with moderate to severe cognitive impairment or terminally ill	211	N/I
Community-dwelling older adults	Inclusion: age and no diagnose of dementia	n = 10405 (n = 2357, Cuba; n = 1592, Dominican republic; n = 1640, Mexico; n = 1638, Puerto Rico;	N/I
Catchment areas	N/I	n = 12,865	N/I

N/I	N/I	n = 12774 (Cuba = 2944; Dr = 2011; P. Rico= 2011; Peru Urban = 1381; Peru rural= 552;	N/I
community-dwelling older adults	N/I	DEMQOL UK: n =868 and LA: n = 417. Informant reports were collected using DEMQOL-Proxy UK:	N/I
University clinics in 4 centres	Inclusion: PD UK Parkinson’s Disease SoICety Brain Bank Criteria, ≥ 30 ys at onset, HY 1 to 5 . Exc : non- PD; 2) other	n = 331 (61 argentina, 127 brazil, 75 ecuador, paraguay	N/I
University clinics in 4 centres	Inclusion: PD UK Parkinson’s Disease SoICety Brain Bank Criteria, ≥ 30 ys at onset, HY 1 to 5 . Exc : non- PD; 2) other	n = 388	N/I
community based prevalence studies within 10/66 collaboration sites	All available/consenting residents and iinformants within catchments interviewed. As per 10/66	n = 2937 (cuba) 2001 dom republic 1380 peru Urban 552 peru rural 1947 venezuela 1002	N/I
community based prevalence studies within 10/66 collaboration sites	All available/consenting residents and iinformants within catchments interviewed. As per 10/66	n = 14,967	N/I
Urban and rural community sites	within 1066 collaboration study. Otherwise not stated	n = 2941 (1666 latin america and caribbean) n = 1242 for factor analysis	N/I
CRELES (Costa Rica Study on Longevity and Healthy Aging) with adults over 60 in Costa Rica.<br>SABE (Health, Well-being,	Inclusion criteria:<br>≥ 60years<br>Data from CRELES and SABE, including speICfic Latin American ICties.	The total sample for each country is more than 500 persons. The country with the fewest valid cases is	N/I
N/I	N/I	n = 794	N/I
Geriatric institute	Exclusions: no cognitive decline, not in antidepressants (last month)	n = 199	ICC = 0.993 p < 0.001

Hospital	Exclusions: Patients with uncontrolled neurological, toxic, metabolic, infectious, vascular or psychiatric diseases.	n = 168. cognitively healthy (n = 59), MCI (n = 52), dementia (n = 57).	ICC = 0.955 p < 0.001
N/I	Exclusions: No Illiteracy, no uncontrolled systeMCI, toxic, metabolic, infectious, vascular, neurological or psychiatric diseases, no	n = 167. cognitively healthy (n = 58), MCI (n = 52), dementia (n = 57).	ICC = 0.774 p < 0.001
Homes of the elderly	Inclusion: MMSE ≥ 24 PTS.	n = 4966	N/I
National Institute of Older Adults	N/I	n = 355	N/I
N/I	Exclusion: suffering from any acute or severe chronic illness, and being less alert or suffering from severe aphasia, severe impaired	n = 626	N/I
N/I	Exclusion: Diagnostic of cognitive impairment	n = 106	N/I
community dwelling	Exclusion: iliteracy and hearing problems	n = 120	N/I
Social programmes	Inclusion: age and literacy. Exclusion: Signs of dementia, psychosis or any physical disability.	n = 255	N/I
medical appointments on the National institute of neurology	Divided samples by diagnostic: Alzheimer´s disease (EA); Fronto-temporal dementia (DFT);	n = 101 (n = 23 (EA); n = 30 (DFT); n = 36 (DM); n = 12 (DV))	N/I
N/I	inclusion: memory complaints,normal activities of daily living, normal general cognitive function, memory	n = 314 (mild-moderate = 55, MCI = 74, control = 185)	N/I

N/I	Exclusion: Neuological or psychiatric diseases	n = 5120	N/I
community stratified sample	N/I	n = 229	N/I
community (part of sadem study)	Inclusion: i) ≥ 60, living in Mexico city; ii) registered with Mexican Institute ofsocial Security (IMSS); and iii) accepting to take	n = 2905	N/I
community - from study of dental caries risk factors	Inclusion: social security beneficiaries	n = 534	N/I
government health clinic	not stated, consent to participate, attending government clinic	Two samples EAE-0 n = 234, EAE-A n = 151	N/I
University memory clinic	inc: ≥ 60, literate, MMSE ≥ 10, informant available, consented. Controls ≥ 60, literate, MMSE ≥ 24 no subjective cognitive	n = 114	N/I
two health clinics. Onesocial security one older persons association	≥ 50, literate, consented, full data available	n = 129 (sample 1) n = 209 (sample 2)	N/I
family mediICne clinics	N/I	n = 288	N/I
Community	N/I	n = 446	N/I
N/I	Inclusion Criteria: Be Mexican and residing in Mexico. Aged between 50 and 90 years. For participants with	234 Mexican participants (117 HC and 117 individuals with AD)	N/I

N/I	People with symptoms of confusion or diagnosed with dementia or other serious psychiatric illness that prevented them from	970	N/I
Hospital	Exclusion criteria: ≤2 years of education, visual/auditory impairments affecting neuropsychological testing,	230 participants: cognitively healthy (NCS) (n = 70), mild NCD (n = 70), and major NCD (n = 90):	N/I
Hospital	Inclusion Criteria: CH group: Age ≥ 60, normal MMSE and MoCA-E scores. SCD group: SCD in last 5	200	ICC = 0.877 [95% IC: 0.850–0.902; p < 0.001].
medical center	Age ≥ 60, no cognitive impairment, regular residents. MMSE (Spanish) completed; exclusion for score < 24.	1913 participants	N/I
Community	Inclusion Criteria: 60-94 years Recruited from older adult groups in Sonora, Mexico. Informed Consent.	1178	N/I
Community	Inclusion Criteria: ≥ 60 years. Regular residents of the selected household. No cognitive impairment	n = 1,915	N/I
N/I	participants who resided in the city of Chihuahua, aged 60 or more years, who agreed to partiICpate in the study, and who did not	401	N/I
Community	1) being 60 years old or older, 2) having a sexual partner, and 3) having had sexual intercourse in the last 2 months.	634	N/I
Sub-sample of those originally enrolled in the Tumbes site of the CRONICAS Cohort Study	Inclusion: age and diagnosis of hypertension. Exclusion: Visual impairment.	n = 139	N/I
community centers of older adults	N/I	n = 236	N/I

community centers of older adults	N/I	n = 400	N/I
community centers of older adults	N/I	n = 236	N/I
community centers of older adults	N/I	n = 236	N/I
Community	Exclusion: visual, auditory or physical impairment. Language different than Spanish. History of addiction.	n = 210 (control = 107, dg dementia = 103)	N/I
Community	Exclusion: visual, auditory or physical impairment. Language different than Spanish. History of addiction.	n = 210 (control = 107, dg dementia = 103)	N/I
Elderly care home centers	Exclusion: Cognitive impairment, non-related to neurodegenerative, addiction or abuse, depression,	n = 283	N/I
outpatient clinics of neurology and rehabilitation	Exclusion: cognitive function may have been affected through the use of certain drugs or through a particular medical	n = 133	N/I
Older persons clubs and local alzheimers disease association	Inclusion: ≥ 55, no significant sensory impairment, first language spanish	n = 656	N/I
patients of the hospital psychology department	Exclusion: depression on GDS-15, known dementia, known neurological or psychiatric disease	n = 107 (59 without positive depression screen included).	N/I
N/I	N/I	n = 336	N/I

geriatric clinic	Inclusion: AD by NINCDS ADRA and clinical evaluation. Exclusion vision/motor impairment of hands, sensory	n = 31 AD; n = 31 controls	N/I
geriatric clinic	Inclusion, ≥ 60, attending geriatric clinic.	42 cases (DSM-IV dementia); 42 controls	N/I
community	Inclusion: according dementia criteria	n = 210	N/I
Peruvian Institute of Neurosciences; IPN	Controls: Healthy volunteers based on neuropsychological and neurological evaluation, recruited locally and	63 cognitively healthy, 53 amnestic MCI and 69 dementia due to AD	N/I
regional health clinics within Lima, Peru.	1. ≥ 60 years 2. Illiterate persons of at least 15 years old defined as one with no education 3. native Spanish speakers or speak Spanish	n = 187 (controls n = 60; MCI n = 64; dementia n = 63)	N/I
Medical Center.	Patients without complete MMSE, MoCA o SPMSQ scores were excluded from the study	1683	MMSE and MoCA K= 0.99 (95% IC: 0.99–1.00). MMSE and
Medical Center	Inclusion Criteria: ≥ 50 years, dementia diagnosis (DSM-V), mild to moderate cognitive impairment	51, AD (n = 33), DFTvb (n = 18)	N/I
Hospital	Inclusion Criteria: HC of elderly patients in geriatrics and neurology at FAP. Diagnosed with or without	432 medical records	N/I
Community	Inclusion criteria: Adults aged 60 and older.	274	N/I
Medical center	Inclusion Criteria: ≥ 55 years. Native Spanish speakers. At least 4 years of formal education.	204	N/I

N/I	1) ≥ 60 years 2) consent to partiICpate in the study. The exclusion criteria were: 1) age < 60 years; 2) not giving consent to	298	N/I
health centers and an institutionalized center	N/I	100	N/I
Centers for the Elderly	(1) people older than 60 years of age; (2) without any physical(functional) or mental disability (dementia) and; (3) who	235	N/I
Peruvian Institute of Neurosciences (IPN)	Inclusion Criteria: ≥ 60 years old. Diagnosis: Alzheimer's Disease (AD), Mild Cognitive Impairment	276 individuals (AD: 113, MCI: 68, controls: 95)	N/I
Community and medical center	Inclusion Criteria: ≥ 50 years old. Illiteracy: Less than 1 complete year of formal education and unable to	187; 63 patients with early Alzheimer's disease dementia (ADD), 60 amnestic mild	N/I
N/I	(1) being ≥ 60 and (2) not have intellectual difficulties that prevent understanding the instructions and the content of the survey.	400	N/I
centers for the Elderly	a) minimum age 60 years; b) no physical (functional) or mental (dementia) disabilities, and c) having given informed consent	235	ICC = 0.94 WTLS
community	Inclusion criteria: a) minimum age of 60 years; b) no apparent physical (functional) or mental (dementia) disability; c)	323	N/I
medical center	Not suffering from prior cognitive impairment (either moderate or severe) or symptoms of depression.	300	N/I
Community	(a) minimum age 60 years; (b) without any apparent physical (functional) or mental disability (dementia) and; (c) have	236 Peru, 133 Spain	N/I

Community and medical center	Inclusion Criteria:<br>AD group: GDS score of 4 (early Alzheimer’s disease).<br>MCI group: GDS score of 3 (mild cognitive	232, 76 had Alzheimer's disease (AD) in the initial phase, 75 had mild cognitive	Inter-rater agreement method of Morillama: NeuroBel
geriatric institutions and outpatients	N/I	n = 103	N/I
Hospital	Patients younger than 40 years of age, or with a previous history of neurological, psychiatric, endocrine or systeMCI	587	N/I

Test Retest (Reliability)	Internal Consistency (Reliability)	Sensitivity (Diagnostic Accuracy)	Specificity (Diagnostic Accuracy)	AUC (Diagnostic Accuracy)	Cut-off point (Diagnostic Accuracy)
N/I	N/I	42%	79%	N/I	133 pts
N/I	a = 0.754	80%	83%	0.89	2
N/I	N/I	39%	89%	N/I	N/I
N/I	a = 0.80	84%	95%	0.992 (95% IC 0.962-1)	40/50
Total: r = 0.65; 3-14 days	a = 0.85-0.9 in subtests	N/I	N/I	N/I	N/I
N/I	N/I	69%	88%	0.88	standard cut points
N/I	a = 0.98	MCI v AD 84, CN v MCI 90, CN v AD 99	MCI v AD 60, CN v MCI 80, CN v AD 82	MCI v AD 0.81, CN v MCI 0.96, CN v AD 0.99	MCI v AD 54, CN v MCI 45, CN v AD 44
N/I	N/I	AD 89.29, a-MCI 85.25	AD 96.67, aMCI 90	phototest a-MCI 0.93, AD 0.97	AD 27/28 a-MCI 30/31
N/I	a = 0.64	92%	78%	N/I	N/I

N/I	a = 0.709	84%	84%	N/I	70 score
N/I	N/I	CSI=0.84	CSI=0.90	AUC CSI = 0.93	86
N/I	N/I	N/I	N/I	N/I	N/I
N/I	N/I	N/I	N/I	N/I	N/I
N/I	a = 0.82	N/I	N/I	N/I	N/I
N/I	a = 0.81	N/I	N/I	N/I	N/I
rs= 0.949, rs= 0.949 (p < 0.001).	a: 0.8866.	84.8% for MCI; 100% for mild dementia.	69.7%.	MCI: 0.877 (95% IC [0.841-0.914]). Mild dementia: 0.99 (95% IC	≥ 25.
N/I	Internal Consistency (KR-20): 0.779 (penICl-and-paper)	N/I	N/I	N/I	N/I
N/I	Overall Reliability (2 Factor Model): Factor 1 = 0.695, Factor 2	N/I	N/I	N/I	N/I
N/I	ωNL = 0.72	N/I	N/I	N/I	N/I

N/I	a = 0.87	control group and ATD group = 98.5% /control group and in the bv-	control group and ATD group = 82.01%/control group and in	control group and ATD group = 0.98 (IC: 0.96-0.99; P < .01) /control group	ATD= 86. bv-FTD= 87
N/I	N/I	82% (Prefrail + frail); 81% (Frail)	72% (Prefrail + frail); 70% (Frail)	0.88 p < 0.05 (Prefrail + frail); 0.84 p < 0.05 (Frail)	0.3 (Prefrail + frail); 0.4 (Frail)
N/I	a = 0.835	N/I	N/I	N/I	N/I
N/I	a = 0.77	81.8% (I); 90% (II); 94.6% (III)	100% (I,II,III)	0.993	85 pts(I), 90 pts (II), 94.6 pts (III).
N/I	a = 0.860	74.60%	73.60%	N/I	≥ 11
0.706 (p < 0.001)	a = 0.730	N/I	N/I	N/I	N/I
N/I	N/I	Verbal fluency: 73%; Boston naming: 61.9%; MMSE:97.6%; Word list	Verbal fluency: 87.1%; Boston naming: 69.4%; MMSE: 75.3%; Word list	Verbal fluency: 0.877; Boston naming: 0.699; MMSE: 0.942; Word list	Verbal fluency: 1 pts; Boston naming: 12 pts; MMSE: 26 pts; Word list
K = of 0.381; 64.3% test-retest.	a = 0.827	N/I	N/I	N/I	N/I
N/I	N/I	80%	60.50%	N/I	1/2
N/I	N/I	N/I	N/I	N/I	N/I

N/I	N/I	N/I	N/I	N/I	N/I
N/I	N/I	N/I	N/I	N/I	N/I
N/I	N/I	N/I	N/I	N/I	N/I
r = 0.92, p = 0.01	N/I	94%	84%	0.982 (IC95% = 0.964–1.001)	15 pts
N/I	N/I	83%	83%	0.87 (IC 0.79-0.95)	12 pts
N/I	N/I	100%	82.26%	0.947	< 78 pts
N/I	N/I	N/I	N/I	N/I	N/I
r = 0.76	N/I	N/I	N/I	N/I	N/I
N/I	N/I	N/I	N/I	N/I	N/I
N/I	a = 0.876 (CCQ8); a = 0.936(CCQ22)	98.7% (CCQ-8)	66.6%(CCQ-8)	0.968 (p < 0.001) (CCQ-8)	≥ 1 (CCQ-8)

N/I	KR-20= 0.69 (p < 0.001)	N/I	N/I	N/I	N/I
N/I	N/I	81.50%	76.10%	(0.87 [95%IC 0.82-0.93])	< 23
N/I	N/I	N/I	N/I	N/I	N/I
N/I	N/I	86% (questionable and dementia cases)	80% (questionable and dementia cases)	N/I	N/I
N/I	N/I	86.4% (MMSE 61.9%)	63.9% (MMSE 86.5%)	N/I	N/I
N/I	Frequency a = 0.956; Pleasure a = 0.954; Obtained Pleasure a =	N/I	N/I	N/I	N/I
N/I	N/I	N/I	N/I	N/I	≥ 12 pts Severe disability, 6-11 Moderate disability, 2-5 Mild disability.
N/I	N/I	100% (NC vs AD, 8 years) 100% (NC vs AD, >8 years) 88% (MCI vs	82.1% (NC vs AD, 8 years) 87.5% (NC vs AD, >8 years) 74% (MCI vs	0.981 (NC vs AD, 8 years) 1.000 (NC vs AD, >8 years) 0.888 (MCI vs	7.5 (NC vs AD, 8 years) 6.5 (NC vs AD, >8 years) 9.5 (MCI vs AD,
N/I	a = 0.80	99.1% (without dementia) 77.9% (MCI and dementia)	N/I	N/I	≥ 9 points (without dementia) ≥ 5 points (MCI and dementia)
t = 1.08 (p = 0.28)	Ranged a = 0.710 (autonomy) to a = 0.885 (overall).	N/I	N/I	N/I	N/I

N/I	N/I	75%	75%	ROC=0.83 (95% IC 0.76–0.89).	≤5 pts (Youden Index)
N/I	N/I	N/I	N/I	N/I	N/I
N/I	N/I	N/I	N/I	N/I	N/I
N/I	N/I	91.40%	83.90%	0.945	4/5
N/I	N/I	86.4% (MMSE showed 61.9%)	63.9% (MMSE showed 86.5%)	N/I	N/I
N/I	N/I	FAQ: 84%; Verbal fluency (CVF): 71.3%; CDT: 79.2%; Delayed recall:	FAQ: 94%; Verbal fluency (CVF): 69.8%; CDT: 67.4%; Delayed recall:	FAQ: 0.945; Verbal fluency (CVF): 0.777; CDT: 0.821; Delayed recall:	FAQ: 3 pts; Verbal fluency (CVF): 10 pts; CDT: 6 pts; Delayed recall:
N/I	N/I	ICND (FAQ: 90.9%; CFT: 56.4%; FAQ+CFT: 86.4%). (FAQ:	ICND (FAQ:89.1%; CFT: 77.3%; FAQ+CFT: 80.6%)	ICND (FAQ:0.909 IC95 (0.836-0.983); CFT: 0.678 (0.578-	ICND (FAQ: 1; CFT: 11; FAQ+CFT: -2.54)
N/I	a = 0.7873	N/I	N/I	0.8851	5 positive responses
N/I	a = 0.856 (original), a = 0.848 (adapted), a = 0.698 (3o item	N/I	N/I	N/I	N/I
N/I	a = 0.92	N/I	N/I	N/I	N/I

Dream distUrbance: K = 0.477; depresivve humor: K =	N/I	N/I	N/I	N/I	N/I
N/I	N/I	N/I	N/I	N/I	N/I
N/I	N/I	N/I	N/I	N/I	N/I
N/I	N/I	Original score: 88% (Manos); 84% (Shulman); 62% (Wolf-Klein); 59%	Original score: 28% (Manos); 36% (Shulman); 69% (Wolf-Klein); 64%	0.668 (Manos); 0.684 (Shulman); 0.684 (Wolf-Klein); 0.657	Original score: 7/8 (Manos); 3/2 (Shulman); 6/7 (Wolf-Klein); 5/6
N/I	N/I	89%	72%	0.88 (95% confidence interval: 0.837-0.917)	3.26
N/I	N/I	Total: 80.8%; Illiterate: 73.5%; Literate: 75%.	Total: 65.3%; Illiterate: 73.9%; Literate: 69.7%.	0.807. 44.7% (positive), 90.7% (negative)	Total: 23/24; Illiterate: 18/19; Literate: 24/25.
N/I	r = 0.82 long version; r = 0.83 short version	N/I	N/I	N/I	N/I
N/I	N/I	General:84.3%; no formal education: 80%; School history: 77.8%	General: 60.3%; no formal education: 70.9%; School history: 75.4%	N/I	General:23/24 (cases/non-cases); no formal education:
GDS-15: K = 0.64; GDS-10: K = 0.60; GDS-4: K = 0.37	N/I	N/I	N/I	N/I	N/I
N/I	GDS-15: a = 0.81; GDS-10: a = 0.75; GDS-4: a = 0.41	GDS-15:92.7%, 97%; GDS-10: 80.5%, 84.8%; GDS-4:80.5%, 84.8%; GDS-	GDS-15: 65.2%, 97%; GDS-10: 78.3%, 67.7%; GDS-4: 78.3%,67.7%;	N/I	GDS-15:4/5 pts;GDS-10: 4/5 pts; GDS-4:2/3 pts

N/I	N/I	N/I	N/I	N/I	N/I
N/I	a = 0.90	N/I	N/I	N/I	N/I
N/I	N/I	87% (original); 67% (Conservative)	N/I	N/I	≤ 25 points (original); ≤ 22 points (Conservative)
N/I	N/I	1-4 years: 88.1%; 5-8 years: 84.6%; ≥ 8 years:70.8%; Total: 83.2%	1-4 years: 83.5%; 5-8 years: 96%; 78.8% ≥ 8 years: 90% Total: 84.6%	1-4 years: 0.915 (< 0.001); 5-8 years: 0.922 (< 0.001; ≥ 8 years: 0.813 (< 0.001)	1-4 years: 79 pts; 5-8 years: 80 pts; ≥ 8 years: 90 pts; Total: 25 pts
N/I	N/I	74.2%- 85.8% (Mendez, Shulman, Sunderland)	66.7%-89.9% (Mendez, Shulman, Sunderland)	0.840-0.860 (Mendez, Shulman, Sunderland)	N/I
N/I	N/I	N/I	N/I	N/I	N/I
N/I	N/I	N/I	N/I	N/I	N/I
N/I	N/I	N/I	N/I	N/I	N/I
N/I	N/I	GDS-30: 92.0% (95% IC: 70–98); GDS-15: 87% (95% IC: 71–95); GDS-	GDS-30: 79% (95% IC: 73–85); GDS-15: 82% (95% IC: 76–91); GDS-	N/I	GDS-30: 10/11; GDS-15: 4/5;GDS-10: 3/4; GDS-4: 0/1 ;GDS-1:
N/I	WHOQOL-BREF a = 0.832; SF-36 a = 0.868.	N/I	N/I	N/I	N/I

N/I	N/I	N/I	N/I	N/I	N/I
N/I	N/I	N/I	N/I	N/I	N/I
N/I	N/I	N/I	N/I	N/I	Appendix of article
N/I	N/I	100% (AD); 91% (OCD); 100% (paraphrenia)	64% (AD); 75% (OCD); 75% (paraphrenia)	0.756, p = 0.010 (AD); 0.585; p = 0.482 (OCD); 0.716; p =	5 pts (control); 4 pts (AD); 3 pts (paraphrenia)
N/I	N/I	N/I	N/I	N/I	N/I
N/I	N/I	MCI X AD (MoCA: 0.87 (0.78–0.97); MoCA-R: 0.85 (0.75–0.96))	MCIXAD (MoCA: 0.76 (0.64–0.88); MoCA-R: 0.87 (0.77–0.96))	MCIXAD (MoCA 0.91: (0.85–0.96); MoCA-R :0.95 (0.90–0.98))	MCIXAD (MoCA: 20.5; MoCA-R: 8.5); MCIXControl (MoCA: 24.5;
N/I	N/I	82.20%	92.30%	N/I	N/I
N/I	N/I	N/I	N/I	N/I	N/I
N/I	N/I	N/I	N/I	N/I	N/I
N/I	a = 0.925	N/I	N/I	N/I	N/I

N/I	N/I	N/I	N/I	N/I	N/I
N/I	a = 0.89	86.20%	70%	0.89	72 pts
K = 0.814	a = 0.818	N/I	N/I	CDR 0 and CDR ≥ 0 (AUC = 86.1%) and between CDR 0 and CDR 0.5	N/I
N/I	N/I	N/I	N/I	N/I	N/I
N/I	N/I	Clock test 75%; Appointment 90%; Belonging-item 55%; Belonging-	Clock test 75%; Appointment 85%; Belonging-item 65%; Belonging-	N/I	N/I
ρ = 0.85, p < 0.001	a = 0.91	83.30%	84.65%	N/I	13 pts (generalised anxiety)
N/I	a = 0.75	81% (MCI); 91% (AD)	77% (MCI); 100% (AD)	0.82 95% IC = 0.67–0.94 (MCI); 0.99 95% IC = 0.98–1.00) (AD)	25 pts (MCI)
0.875; p < 0.001	a = 0.77	81%	73%	N/I	N/I
N/I	a = 0.08; 95% IC 0.7-0.8)	95.6% (MCI)	90.16% (MCI)	0.8; 95% IC (0.7-0.9) (MCI)	20 pts (MCI)
N/I	N/I	91.20%	100%	N/I	N/I

N/I	K = 0.41, p < 0.001.	80%	44%	0.70 (95%IC: 609-791; p < 0.001)	N/I
N/I	GDS-30 a = 0.87; GHQ-12 a = 0.82, 0.86 and 0.81 for classical GHQ, Likert and	GDS-30: 73%; GHQ, c-GHQ: 66–75%	GDS-30: 65%; GHQ, c-GHQ: 57–62%	GDS-30 = 0.76; classical GHQ = 0.74; Likert = 0.76; c-GHQ = 0.73	GDS-30 (14/15), GHQ (4/5), Likert (15/16) and c-GHQ (5/6)
N/I	N/I	0.949	0.93 (0.077=1-spec)	0.98	80.5
N/I	a: PQOL = 0.80, C-PQOL = 0.83, CQOL = 0.86	N/I	N/I	N/I	N/I
N/I	N/I	illiterate 69% low ed 83%, med ed 90%	illiterate 69%, low ed 85%, mid ed 76%	illiterate 0.75, low ed 0.93, mid ed 0.91	illiterate 50/51, low ed 60/61, mid ed 69/70
mn 30 days ICC 0.87 - 0.94 (slightly lower 0.87 in dementia as	CA 0.89	N/I	N/I	N/I	N/I
N/I	N/I	(4/5) 86,(5/6) 81, (6/7) 73	(4/5) 63,(5/6) 71, (6/7) 78	At 5/6, AU ROC curve 0.85 (95% IC: 0.790.91).	optimal 5/6
N/I	N/I	N/I	N/I	N/I	N/I
N/I	a = Self-care ADLs (0.806), domestic ADLs (0.810), complex ADLs	74 + 0.806 (< 74 0.689)	74 + 0.791 (< 74 AUC 0.618)	74+ global 0.806. < 74 =0.725	total score 23/24 (case/non-case)
N/I	a = 0.831	N/I	N/I	N/I	N/I

N/I	N/I	81	88.5	AD vs controls 0.869 (p < 0.001), MCI vs controls 0.821 (p < 0.001)	4.005
N/I	N/I	IQCODE-L = 88.24 (81.66–94.82) IQCODE-S = 85.3	IQCODE-L = 77.97 (69.42–86.38), IQCODE-S = 93.1	IQCODE-L = 0.94 (95% IC: 0.88–1.00), IQCODE-S = 0.96 (95% IC:	IQCODE-L = 3.38, IQCODE-S = 3.53, IQCODE-SBr = 3.6
N/I	0.80 (IC95% 0.76; 0.84) (KR-20)	85.41%	84.56%	0.89	6 to 7/15
N/I	N/I	N/I	N/I	N/I	N/I
N/I	N/I	60%	65%	0.6760 (95% IC 0.390.599 - 0.753)	2 out of 3
ICC 0.946 (1 week)	N/I	N/I	N/I	N/I	N/I
N/I	N/I	78.7% sensitivity (no education), 91.9% (education)	77.8% (no education), 89.5% (Education)	0.87 (no education) 0.94% education)	14/15 (no education) 17/18 (education)
ICC = 0.92 (md 28 days range 14-60 days)	a = 0.94	N/I	N/I	N/I	N/I
N/I	N/I	83.30%	80.70%	0.83 (95% IC:0.766–0.893)	3.52
N/I	N/I	76.10%	74.60%	0.82-0.83	4 to 5

N/I	N/I	N/I	N/I	N/I	N/I
N/I	N/I	76.80%	63.80%	0.758	< 60
N/I	N/I	88%	66.40%	0.934	< 60
GAI: r = 0.58 (sig) GAI-SF: r = 0.97 (sig)	GAI: a = 0.89; GAI-SF: a = 0.62	N/I	N/I	N/I	N/I
N/I	a = 0.71	80% (1-7 y ed) 84.6% (8+y ed)	77.8% (1-7 y ed) 86.1% (8+y ed)	N/I	6.49 (1-7 years ed) 8.67 (8+ years ed)
N/I	N/I	100% dementia v control, 64% MCI v controls, 81% MCI vs dementia	95% dementia v control, 88% MCI v control, 88% MCI v dementia	0.99 dementia v control, 0.83 MCI v control, 0.91 MCI v dementia	92/93 dementia v controls 95/96 MCI vs control 85/86 MCI v
N/I	N/I	N/I	N/I	N/I	N/I
N/I	a = 0.86	MADRS = 83.3 CSDD = 83.33	MADRS = 60.0 CSDD = 53.85	MADRS = 0.75 CSDD = 0.71	MADRS = 10 CSDD = 13
N/I	a = 0.628	N/I	N/I	N/I	N/I
N/I	personal semantics (cronbach) 0.73 and (autobiographic	N/I	N/I	N/I	N/I

0.75-0.8 (3 months pearson	a = (0.75-0.89)	N/I	N/I	N/I	N/I
N/I	a = 0.79 (partiICpant) 0.83 carer total needs. Unmet needs 0.57	N/I	N/I	N/I	N/I
N/I	N/I	N/I	N/I	N/I	N/I
N/I	a = 0.87 all, 0.85 AD, 0.55 MCI, 0.60 controls	< 8 yr ed AD 93%, MCI 69% profile score AD 90%, MCI 67% (screening	< 8 yr ed AD 95%, MCI 79% profile score AD 95%, MCI 84% (screening	< 8 yr ed AD 0.981, MCI 0.815 profile score AD 0.973, MCI 0.810	< 8 yr ed AD 15.5, MCI 19.5 profile score AD 6.5, MCI 8.5 (screening
N/I	a = 0.868 (positive dimension); a = 0.70(negative dimension)	N/I	N/I	N/I	N/I
N/I	a = 0.94	N/I	N/I	N/I	N/I
ICC 0.98-0.99, weighted K = 0.64-1 15 days test-retest	a = 0.71-0.94 (overall test 0.9)	N/I	N/I	N/I	N/I
N/I	N/I	N/I	N/I	N/I	N/I
n = 40 ICC = 0.86 (95%IC = 0.76–0 .93); n = 50 ICC = 0.87	a = 0.69	87.7% (frailty)	68.3% (frailty)	0.851 (frailty)	8 (frailty) nb previous public health cut-off 11
as inter-rater reliability	ρcr = 0.85	N/I	N/I	N/I	N/I

N/I	N/I	N/I	N/I	N/I	N/I
N/I	N/I	N/I	N/I	N/I	N/I
N/I	a = 0.92	N/I	N/I	N/I	N/I
N/I	a = 0.95 ω = 0.98	1 (est)	0.91 (est)	1.00 (est)	12
N/I	a = 0.981	97%(I); 87%(II)	88.1% (I); 96.6% (II)	0.97(p < 0.0001, 95% IC: 0.94-1.00)	2.58 (I); 3.12 (II)
N/I	N/I	N/I	N/I	AD = 1.000; MCI = 0.970	N/I
N/I	N/I	N/I	N/I	N/I	N/I
N/I	N/I	Control v/s dementia: 90%; Control v/s impairment: 84%	Control v/s dementia: 77%; Control v/s impairment: 49%	N/I	Control v/s dementia: 15 pts; Control v/s impairment: 19 pts
NHP = 0.94; SF-36 = 0.83	NHP: a = 0.86; SF-36: a = 0.80	N/I	N/I	N/I	N/I
0.91 (95% IC 0.74–0.96; p < 0.001)	a = 0.77	N/I	N/I	Control: 95 (0.92–0.98); MCI: 0.94 (0.90–0.97); AD: 0.99	N/I

N/I	PCC = 0.60 to 0.93; ICC = 0.60 to 0.93	N/I	N/I	N/I	N/I
N/I	ICC = 0.7393	78%	84%	N/I	N/I
N/I	N/I	89%	96%	0.936	≤ 44 points for AD diagnosis.
N/I	N/I	MMSE: 0.89 (MD vs MCI) and 0.89 (MD vs Controls). ACE-R: 1.00	MMSE: 0.85 (MD vs MCI) and 1.00 (MD vs Controls). ACE-R: 0.74	MMSE: 0.84 (MD vs MCI), 0.96 (MD vs Controls), 0.69 (MCI vs	MMSE: 19.5 (MD vs MCI), 20 (MD vs Controls), 26.5 (MCI vs
N/I	Total LIFE-H: a = 0.93 Personal Care: a = 0.88 Community: a =	N/I	N/I	N/I	N/I
12-Month Follow-Up	N/I	N/I	N/I	N/I	N/I
N/I	a = 0.88	N/I	N/I	N/I	N/I
N/I	KR-20 0.85 penICl-and-paper version and 0.90 online version	N/I	N/I	N/I	N/I
N/I	N/I	N/I	N/I	N/I	N/I
N/I	N/I	Alzheimer's: 92% Vascular Dementia: 95%	Alzheimer's: 91% Vascular Dementia: 94%	Alzheimer's (AD): 0.958 Vascular Dementia (VD): 0.982	Alzheimer's (AD): 23 Vascular Dementia (VD): 22

N/I	a = 0.844	75.47%.	85.0%.	AUC= 0.865 (95% IC: 0.764 - 0.933; p < 0.0001)	≥ 14
N/I	N/I	Supermarket: 67.74%<br>Animal: 54.84%	Supermarket: 86.84%<br>Animal: 73.68%	Supermarket: 0.840 (95% IC: 0.746-0.933)<br>Animal: 0.671 (95% IC: 0.543-	Supermarket: ≥ 15<br>Animal: ≥ 12
N/I	a = 0.878	79.20%	80%	N/I	≥ 29 points.
N/I	CFI Self-report: a = 0.80 (95% IC 0.73–0.86).<br>CFI Partner report: a = 0.70	73.3% (for self-report).	81.5% (for self-report).	CFI Self-report: AUC = 0.865 (95% IC 0.779–0.951, p < 0.001).	CFI Self-report cutoff score = 2.0.
0.994 (p < 0.001).	ω = 0.844	N/I	N/I	N/I	Low: 61-74<br>Moderate: 75-87<br>High: 88-95
ICC = 0.835	a = 0.705	N/I	N/I	N/I	N/I
N/I	H-S/EAST (PB): a = 0.08<br>H-S/EAST (PE): a = 0.57<br>CTS-1 (PB): a =	N/I	N/I	N/I	N/I
ICC of 0.927 (p < 0.001)	a = 0.838	77.80%	95.70%	0.931 (p < 0.001)	≥ 7.5 (on a scale of 0–21)
N/I	N/I	AD vs. Controls (Memory Subscale): 83.3%<br>bvFTD vs.	AD vs. Controls (Memory Subscale): 83.0%<br>bvFTD vs.	AD vs. Controls (Memory Subscale): 0.876<br>Total ACE-R for AD vs. Controls:	Memory Subscale (AD vs. Controls): 16 points<br>Total ACE-R
N/I	a: 0.66 to 0.71 for the factors.<br>Composite Reliability: 0.74 to 0.83.	N/I	N/I	N/I	N/I

N/I	a = 0.92	N/I	N/I	N/I	N/I
N/I	a = 0.844	75.47%	85%	0.865 (95% IC: 0.764 - 0.933; p < 0.0001),	total score greater than 14
N/I	uni-factorial model(Ω = 0.83)	N/I	N/I	N/I	N/I
N/I	N/I	N/I	N/I	N/I	N/I
N/I	N/I	N/I	N/I	Validation cohort: 0.78 (95% IC: 0.66–0.90); Develpment	N/I
N/I	a = 0.88	N/I	N/I	N/I	N/I
N/I	N/I	N/I	N/I	N/I	N/I
N/I	a = 0.91-0.92	N/I	N/I	N/I	N/I
N/I	Religiosity ɑ = 0.87; Spirituality ɑ = 0.93.	N/I	N/I	N/I	N/I
N/I	Community integration a = 0.855 (0.933); Community participation	N/I	N/I	N/I	N/I

N/I	a = 0.72	0.88	0.9	N/I	≥ 2 pts (depression)
Rh=0.87	a = 0.85	0.93 (82.74-100.00)	0.90 (77.60-100.00)	0.97	≤ 20
N/I	N/I	30.8% (IC 95% 9%-61.4%)	90.2% (IC 95% 76.9%-97.3%)	0.77 (95% IC; 0.61-0.93)	N/I
N/I	a = 0.86–0.87	N/I	N/I	N/I	N/I
N/I	a = 0.931	N/I	N/I	N/I	N/I
N/I	0.90 CA	N/I	N/I	N/I	N/I
N/I	0.776 CA	93.1	82.2	AUC = 0.96	39
N/I	a = 0.918.	92%	93%	N/I	76
N/I	a = 0.8595	N/I	N/I	N/I	N/I
N/I	N/I	MMSE 93.6%, PFAQ 89.2%, Combined 94.4%	MMSE 46.1%, PFAQ 70.7%, Combined 83.3%	not stated, can be estimated from graph MMSE 0.78, PFAQ 0.92	MMSE 21/22, PFAQ 5/6, Combined

N/I	a = 0.80 (total scale); a = 0.50 (model c)	N/I	N/I	N/I	N/I
N/I	a = 0.915 (Spouse), a = 0.934 (Children), a = 0.942 (Others)	N/I	N/I	N/I	N/I
N/I	a = 0.797	80.80%	90.40%	0.92	13.5
N/I	a = 0.749	Mild NCD vs. HE=0.828, Mild and major NCD vs. HE= 0.856, Major NCD vs.	Mild NCD vs. HE= 0.841, Mild and major NCD vs. HE= 0.903, Major NCD vs.	Mild NCD vs. HE = 0.904, Mild and major NCD vs. HE= 0.942, Major NCD vs.	YOUDEN INDEX Mild NCD vs. HE=0.669, Mild and major NCD vs. HE= 0.759,
N/I	a: non-indigenous = 0.88, aymara = 0.83, Mapuche=	N/I	N/I	N/I	N/I
N/I	General scale a = 0.92; spiritual practices dimension a = 0.822; spiritual	N/I	N/I	N/I	N/I
N/I	Total: 0.73 Personal: 0.56 Social: 0.66 Well-being: 0.49	N/I	N/I	N/I	N/I
N/I	MMSE: 0.535 MoCA: 0.707 ACE-R: 0.81	N/I	N/I	N/I	MMSE (< 21), MoCA (< 20 or < 21), and ACE-R (< 76)
N/I	N/I	96.60%	81.80%	90%	26/27
N/I	a: 0.78 [95% IC: 0.75–0.81]. ω: 0.79 [95% IC: 0.75–0.80].	0.95 (with a cut-off score of 6).	0.76 (with a cut-off score of 6).	0.88 (SE = 0.04, 95% IC: 0.85–0.90).	6

N/I	N/I	N/I	N/I	N/I	N/I
N/I	N/I	Mild NCD vs. Major NCD (Free Recall First Trial): 98.4%	Major NCD vs. HOA (FCSRT Delay Recall task): 97.9%	Mild NCD vs. HOA (Free Recall third trial): 0.738 Major NCD vs.	
N/I	Family Support: a = 0.858, ω = 0.972. Friend Support: a =	N/I	N/I	N/I	N/I
N/I	a = between 0.86 and 0.88 for the scales.	N/I	N/I	N/I	p50 for classifying good or poor HRQoL.
N/I	Emotional Loneliness: KR-20 = 0.62 (non-indigenous), 0.44 (Aymara),	N/I	N/I	N/I	No loneliness (0-1 points): With loneliness (≥ 2 points):
N/I	N/I	N/I	N/I	N/I	N/I
N/I	ordinal alpha value of 0.856	N/I	N/I	N/I	25th percentile: 8 Median: 12 75th percentile: 16
N/I	KR-20: Non-Indigenous = 0.90, Aymara = 0.79, Mapuche = 0.85	N/I	N/I	N/I	N/I
N/I	N/I	Orientation: 0.952 Attention: 0.952 Memory: 0.968	Orientation: 0.814 Attention: 0.414 Memory: 0.832	Orientation: 0.945 Attention: 0.840 Memory: 0.958	Orientation: ≤ 8 Attention: ≤ 6 Memory: ≤ 14 Language: ≤ 23 Visuospatial: ≤
N/I	N/I	N/I	N/I	N/I	N/I

N/I	N/I	N/I	N/I	N/I	N/I
N/I	a = 0.7268	N/I	N/I	N/I	N/I
N/I	a = 0.82 BAS-DEP; a = 0.56 EBAS-DEP	0.77% (BAS-DEP); 0.80%(EBAS-DEP)	0.72% (BAS-DEP); 0.73%(EBAS-DEP)	BAS-DEP: 0.88 (Error Estándar = 0.035); EBAS-DEP: 0.82 (Error estándar =	5/6 (BAS-DEP) y 2/3 (EBAS-DEP).
N/I	a = 0.783	N/I	N/I	N/I	N/I
N/I	a = 0.86	83%	78%	N/I	18 pts
r = 0.88	a = 0.90	N/I	N/I	N/I	N/I
N/I	a = 0.84	85% (76% MCI and 92% MD)	79.50%	0.93 IC(0.89-0.96)	≥ 23 pts
ICC = 0.86, 95% IC (0.76–0.93).	N/I	N/I	N/I	N/I	N/I
N/I	GDS-15: a = 0.78	N/I	N/I	N/I	N/I
N/I	a = 0.925	N/I	N/I	N/I	N/I

r = 0.82 and ICC = 0.89; 95% IC, 0.83- 0.92; P < 0.001.	first application (a = 0.83 and w = 0.87) and in the second one (a = 0.85 and w	N/I	N/I	N/I	N/I
N/I	N/I	N/I	N/I	N/I	N/I
N/I	a = 0.92	N/I	N/I	N/I	N/I
N/I	a = 0.58	N/I	N/I	N/I	N/I
N/I	a = 0.82	86.9% (p < 0.05)	84% (p < 0.05)	0.93 (0.89–0.97)	4
N/I	Ω ≥ 0.70 (except 61-69 años). Total = 0.784 (0.747–0.822)	MMSE= 51.9% ACE-r = 78.5%	MMSE= 71.1% ACE-r = 63.3%	MMSE: AUC = 0.645 ACE-R: AUC = 0.750	ACE-R cut-off = 80.5, MMSE cut-off = 27.5
(ICC: 0.73; 95% IC: 0.55–0.84). Dimension 1: ICC = 0.70 (95% IC: 0.51–0.83)	a total scale: 0.90 (95% IC: 0.88–0.92) ω total scale: 0.90 (95% IC:	80.0% (IC 95%: 72.1-86.5).	57.6% (IC 95%: 44.1-70.4).	0.76 (IC 95%: 0.70-0.83)	10
N/I	N/I	DDT-Pro scores consistently discriminate between delirium, SSD	N/I	N/I	≥ 8 points for 'no delirium', 6-7 points for 'SSD', < 6 points for
N/I	a (total): 0.74<br>a (subcortical): 0.71<br>a (cortical): 0.20	N/I	N/I	0.95 (for PD-CRS against MoCA in diagnosing dementia).	62 points
N/I	MCS-15: a and ω of 0.91. The MCS-10: a and ω of 0.89.	N/I	N/I	N/I	N/I

N/I	For physiICan administration: 0.809 For nurse administration:	At the cutoff ≤6: 88.0% (physiICan), 90.0% (nurse) in the total	At the cutoff ≤6: 85.3% (physiICan), 86.7% (nurse) in the total	AUC for diagnosis according to DSM-5: 94.1% (physiICan),	A cutoff score of ≤6 was chosen for delirium diagnosis.
N/I	N/I	N/I	N/I	N/I	N/I
N/I	Factor 1: a = 0.81 Factor 2 a = 0.74 Total a: 0.84	N/I	N/I	N/I	N/I
N/I	a = 0. 879	89% (MMSE 56%)	72% (MMSE 83%)	0.936 (MMSE 0.638)	84/85 (≤84 for MCI and ≥ 85 for normal controls); (MMSE 25/26,
N/I	N/I	IFS for MCI =0.90, FAB MCI= 0.96	IFS for MCI =0.76, FAB MCI= 0.70	IFS for MCI =0.82, FAB MCI= 0.74	IFS: 20/21 FAB: 13/14
N/I	N/I	BHa = 0.91	BHa = 0.85	BHA AUC of 0.95 (0.91-0.98); with BHS, AUC of 0.99. For dementia,	N/I
N/I	a = 0.92	N/I	N/I	N/I	N/I
N/I	KR20= 0.73.	N/I	N/I	N/I	N/I
N/I	N/I	N/I	N/I	N/I	N/I
N/I	ω = 0.96 (bifactor model ωH = 0.84)	N/I	N/I	N/I	N/I

N/I	a = 0.64 to 0.87 (including China, India and Algeria)	Cuba = 97.2%; Dominican republic= 93.5%; Peru Urban = 92.0%;	Cuba = 87.7%; Dominican republic= 84.0%; Peru Urban = 84.5%;	Cuba = 0.97 IC (0.96-0.98); Dominican republic= 0.95 IC (0.94-0.96);	Cuba = 4/5 (0.85); Dominican republic= 4/5 (0.78); Peru
N/I	DEMQOL (w= 0.87–0.90) and DEMQOL-Proxy (w=0.88 –0.89). Both languages	N/I	N/I	N/I	N/I
N/I	a = 0.87, item homogeneity index,0.38.	N/I	N/I	N/I	N/I
N/I	a = 0.64 (0.71 if item 6 removed)	N/I	N/I	N/I	N/I
N/I	a = 0.90 to 0.95 for latin american sites	N/I	N/I	N/I	N/I
N/I	N/I	N/I	N/I	N/I	N/I
N/I	EUROD latin america a = 0.83 (range for centres: 0.64-0.91)	dementia 63% venezuela - 100% uruguay. Depression 90% stage 1 (70-80%	dementia 75% Mexico - 100% Chile. Data not available for depression. NB	N/I	N/I
N/I	a greater than .81 for all countries except Barbados.	N/I	N/I	N/I	5 or more positive items.
N/I	a = 0.84	N/I	N/I	N/I	N/I
Rs = 0.889 p < 0.001	a = 0.74	80.70%	68.70%	0.792	≥ 5 (9 total)

N/I	a = 0.89	80% MCI; 98% Dementia	75% MCI; 93% Dementia	0.886 (MCI); 0.997 (Dementia)	≤26 MCI; ≤24 Dementia (30 total)
r = 0.637 p < .001	a = 0.750	40% Mild NCD; 90% Major NCD	70% Mild NCD; 95% Major NCD	0.600 p < .001	14 Mild NCD; 12 Major NCD
N/I	a = 0.91	N/I	N/I	N/I	N/I
N/I	a = 0.94	N/I	N/I	N/I	N/I
N/I	a = 0.83	N/I	N/I	N/I	N/I
N/I	a = 0.91	N/I	N/I	N/I	N/I
N/I	a = 0.862	N/I	N/I	N/I	N/I
N/I	a = 0.80	N/I	N/I	N/I	N/I
N/I	N/I	N/I	N/I	N/I	N/I
N/I	N/I	NEUROPSI 82% (I), 87% (1-4), 93% (5-9); MMSE: 95% (I), 90% (1-4), 83%	NEUROPSI 95% (I), 98% (1-4), 98% (5-9); MMSE: 23% (I), 28% (1-4), 85%	N/I	N/I

N/I	N/I	N/I	N/I	N/I	N/I
N/I	a = 0.93	83.3% (ICD-10), 85% (DSM-IV)	90.2% (ICD-10), 83.2% (DSM-IV)	87% (ICD-10), 84% DSM-IV	CES-D 5
N/I	a = 0.769 case (dementia) and 0.790 controls	N/I	N/I	N/I	N/I
N/I	GDS: a = 0.87; CES-DR: a = 0.86	CES-DR 82% GDS 53.8%	CESDR 49.2%, GDS 78.9%	N/I	N/I
N/I	EAE-0 (orig): a = 0.74; EAE-A (adapt): a = 0.78	N/I	N/I	N/I	N/I
QOL-Adp ICC 0,9 (p < 0,001; 95%IC = 0,58-0,91) QOL-Adc 0,81 (p <	a = QOL-Adp 0.88, QOL-Adc 0.82	N/I	N/I	N/I	N/I
N/I	a = 0.82 (B-RCOPE+), 0.65 (B-RCOPE-), 0.91 (ARS)	N/I	N/I	N/I	N/I
N/I	a = 0.90-0.91	N/I	N/I	N/I	N/I
N/I	a = 0.84, IC95%(0.82-0.84)/glb=0.85	N/I	N/I	N/I	N/I
N/I	N/I	For the cutoff point of ≥ 5: 0.921; For the cutoff point of ≥ 4: 0.841.	For the cutoff point of ≥ 5: 0.850; For the cutoff point of ≥ 4: 0.894.	Executive Function: 0.738; Attention and Processing	≤10th percentile: ≥ 5; ≤5th percentile: ≥ 4

N/I	≥ 0.70	N/I	N/I	N/I	N/I
N/I	N/I	MixD: 98% (95% IC, 96-1.00)<br>AD: 96% (95% IC, 84-98)	MixD: 99% (95% IC, 94-1.00)<br>AD: 80% (95% IC, 78-91)	MixD: 0.985<br>AD: 0.920<br>VaD: 0.834<br>Mild NCD: 0.784	MixD: ≤16/20<br>AD: ≤16/20<br>VaD: ≤16/20<br>Mild NCD: ≤18/20
N/I	a = 0.881.	CH: 73%<br>SCD: 99%<br>MCI: 97%<br>Dementia: 75%	CH: 98%<br>SCD: 96%<br>MCI: 65%<br>Dementia: 74%	CH: 0.98 [95% IC: 0.96–0.99, p < 0.001]<br>SCD: 0.70 [95% IC: 0.58–0.82, p	CH: 42 points<br>SCD: 46 points<br>MCI: 52 points<br>Dementia: 85 points
N/I	a total= 0.899<br>asocial Loneliness= 0.892<br>a Emotional	N/I	N/I	N/I	N/I
N/I	KR-21 = 0.80	Discriminates between high (≥ 10 symptoms) and low (< 5 symptoms)	N/I	N/I	N/I
N/I	a = 0.79 to 0.87	The SF-36 adequately distinguishes between people in good	N/I	N/I	N/I
N/I	AAE-4Fm Model (Total Sample)<br>Fear of the Elderly: Ω =	N/I	N/I	N/I	N/I
N/I	a = 0.87	N/I	N/I	N/I	N/I
N/I	N/I	N/I	N/I	N/I	N/I
N/I	w= 0.89; (IC95%: 0.87-0.91); a = 0.86 (IC95%: 0.82-0.89).	N/I	N/I	N/I	N/I

N/I	N/I	N/I	N/I	N/I	N/I
N/I	a = 0.93; ω = 0.93; IC 95%: 0.92 - 0.95; glb = 0.94	N/I	N/I	N/I	N/I
N/I	a = 0.874 (IC 95% 0.84-0.90); ω = 0.878	N/I	N/I	N/I	N/I
N/I	N/I	MMSE: 64.1%; PDR-M: 89.3%; MMSE and PDR-M: 98.1%.	MMSE: 84.1%; PDR-M: 98.1%; MMSE and PDR-M: 84.1%.	MMSE: 0.74; PDR-M: 0.94; MMSE and PDR-M: 0.91.	N/I
N/I	a = 0.82	99%	83.50%	N/I	7 pts
N/I	a = 0.79 ($p < 0.01$).	AD/MCI: 100%; MCI/control: 97.53%	AD/MCI: 99.17%; MCI/control: 91.11%	AD/MCI: 99.6%; MCI/control: 99.56%	AD/MCI: 26; MCI/control: 35
N/I	a = 0.68	N/I	N/I	MCI/ controls: 99.0%; ED and MCI was 89.0%	MCI/ controls: < 24; ED and MCI: < 21
N/I	N/I	94.8%, 72.7%, 95.9%, 98.5%	86.8%, 72.0%, 84.2%, 95.5%	N/I	23 (ed ≥ 0 yrs), 20 (0-3yrs ed), 23 (4-7 yrs), 27 (ed ≥ 7 yrs),
N/I	a = 0.7	91.70%	57.40%	not give (youden 0.254)	27/28
N/I	N/I	N/I	N/I	N/I	N/I

N/I	N/I	83.90%	93.50%	~0.94 (from graph)	6
N/I	N/I	90.50%	83.30%	0.88 from graph	24
N/I	N/I	Dementia MMSE: 64.1%; PDR-M: 89.3%; MMSEXPDR-M: 98.1%	Dementia MMSE: 84.1%; PDR-M: 98.1%; MMSEXPDR-M: 84.1%	Dementia MMSE: 0.74; PDR-M: 0.94; MMSEXPDR-M: 0.91	N/I
N/I	N/I	N/I	N/I	Between cognitively healthy and impaired participants:	To differentiate between controls and cognitively impaired: 37
CCC = 0.61 between the test and retest phases ICC = 0.96	a = 0.65.	MCI vs. Control= 89%. MCI vs. Dementia = 95%	For MCI vs. Control: specificity = 93%. MCI vs. Dementia:	Control vs. MCI: RUDAS-PE = 0.9828 MCI vs. Dementia:	Control vs. MCI= 23. For MCI vs. Dementia = 19.
N/I	N/I	N/I	N/I	N/I	Youden Index: Cut off scores for cognitive impairment were ≥ 4 on SPMSQ, < 26
N/I	N/I	ACE-III: 67%; IFS: 76%; Mini-SEA: High	ACE-III: 94%; IFS: 67%; Mini-SEA: 83%	ACE-III: 0.85; IFS: 0.78; Mini-SEA: Ideal	ACE-III: 70; IFS: 19
N/I	a: 0.863	N/I	N/I	N/I	N/I
N/I	a = 0.89, ω= 0.86	N/I	N/I	N/I	N/I
N/I	N/I	N/I	N/I	VIC vs. controles: ACE-Pe: 0.99, IFS-Pe: 0.99, MMSE-Pe: 0.87	IC vs. controles: ACE-Pe: 74, IFS-Pe: 24, MMSE-Pe: 24 VD vs.

N/I	a = 0.903; ω = 0.905	N/I	N/I	N/I	N/I
N/I	a = 0.75	80%	100%	91.70%	251
N/I	a: 0.80.<br>CRI for common variance: 0.82.	N/I	N/I	N/I	N/I
ICC = 0.996 (IC: 0.995-0.998).	a: AD 0.937; All Groups = 0.962. Item 2 (“walking”) negatively	good sensitivity for detecting the most severe stages of AD (with	N/I	N/I	N/I
ICC:<br>Free Recall: 0.959<br>Total Recall: 0.967	a (free recall): 0.81<br>a (total recall): 0.77	N/I	N/I	AUC:<br>Control vs aMCI: 1<br>Control vs early ADD: 1	Free Recall: 16 (aMCI vs Controls)<br>Total Recall: 26 (aMCI vs
N/I	Model 4: CRI = 0.878 (general), 0.411 (speICfic factor).<br>Relative	N/I	N/I	N/I	N/I
N/I	a = 0.93	N/I	N/I	N/I	N/I
N/I	Omega coefficient = 0.80 (95% IC: 0.76 - 0.82)	N/I	N/I	N/I	N/I
N/I	Sensory skills: a = 0.94<br>Autonomy: a = 0.95<br>Past, present	N/I	N/I	N/I	N/I
N/I	a: Spain 0.83, Peru 0.87<br>Omega: Spain 0.82, Peru 0.87	N/I	N/I	N/I	N/I

Guttman split coeffiICent: Overall test reliability: 0.78.	a = 0.77	AD vs. MCI + control: = 0.97. AD + MCI vs. control: = 0.96. MCI vs.	AD vs. MCI + control: = 0.80. AD + MCI vs. control= 0.52. MCI vs.	AD vs. MCI + control: AUC = .97. AD + MCI vs. control: AUC =	≥ 92.25 pts: normal performance (no C-L impairment).
N/I	a = 0.84	N/I	N/I	N/I	N/I
N/I	N/I	ICNI vs Control: MMSE and CDT combined: 54% CDT alone: 54%; MCI vs	ICNI vs Control: MMSE and CDT combined: 83% CDT alone: 36%; MCI vs	AD Mild vs Control 0.78 (0.69-0.88) The AUC for the combination of	N/I

Construct and/or Criterion Validity: Convergent, Concurrent, and Predictive	Divergent (Construct Validity)	Type (Exploratory /confirmatory)	Number of factors (Factor Analysis)	% Variance explained (Factor Analysis)
N/I	N/I	N/I	N/I	N/I
MMSE (-0.464: P < 0.001) and CDR (0.514; P < 0.001).	N/I	N/I	N/I	N/I
F(1, 57) = 23.58, p = 0.001 (ANOVA)	N/I	N/I	N/I	N/I
MEC (MMSE), ACE-R, PFAQ. r = 0.678 with MEC. 0.597 with ACE-R, with PFAQ -0.578. Dementia following full clinical assesment and neuroimaging.	N/I	N/I	N/I	N/I
MMSE, CDR, Hospital anxiety and depression scale, zaarit burden interview, neuropsychiatric inventory	distress and depression subscales - MMSE	principal components analysis	3 factors	46% (frequency) 58% global subscale
MMSE (sig), signoret memory battery (sig); MCI clinical diagnosis by neuropsychologist assessment	N/I	N/I	N/I	N/I
MMSE (sig), FAQ (sig)TMT a and B (sig) RAVLT delayed (sig) age (sig) BNT (sig); AD NINCDS-ADRA, MCI	N/I	N/I	N/I	N/I
MMSE (sig), CDT (sig); mild AD NINCDS-ADRA, MCI	N/I	N/I	N/I	N/I
Gold standard	N/I	N/I	N/I	N/I

CDR: Rho = 0–.425, P < .017	N/I	N/I	N/I	N/I
N/I	N/I	N/I	N/I	N/I
N/I	N/I	N/I	N/I	N/I
N/I	N/I	N/I	N/I	N/I
N/I	N/I	N/I	N/I	N/I
CRC and the digit and verbal fluency tests are significant at the p < 0.01 level; Significant correlations with digits and fluency at the same time.	N/I	N/I	N/I	N/I
MMSE: rs=0.710, rs=0.710 (p < 0.0001).	N/I	N/I	N/I	N/I
N/I	N/I	Confirmatory	1 factor	N/I
N/I	N/I	Exploratory and confirmatory	2 factors	38%
MoCA: r = 0.40 (p < 0.001).	N/I	Confirmatory	1 factor	AVE = 0.41

N/I	N/I	N/I	N/I	N/I
The Frailty Index (FI) r = 0.79, p < 0.05	N/I	N/I	N/I	N/I
GPM-p (pain): r = 0.495, p < 0.001); GDS (depression):r = 0,59, p < 0.001	N/I	N/I	N/I	N/I
MMSE: r = 0.44, P < 0001.	N/I	N/I	N/I	N/I
N/I	N/I	Exploratory	3 factors	47.50%
N/I	N/I	Exploratory	2 factors	50.10%
N/I	N/I	N/I	N/I	N/I
VAS r = 0.64; p < 0.001	N/I	N/I	N/I	N/I
N/I	N/I	N/I	N/I	N/I
N/I	N/I	N/I	N/I	N/I

N/I	N/I	N/I	N/I	N/I
MMSE-AD/MMSE-MO: r = 0.878, p < 0.001.	N/I	N/I	N/I	N/I
MMSE: k=-0.02	N/I	Exploratory	2 factors	57%
MMSE: r = 0.92, p = 0.01	N/I	N/I	N/I	N/I
MMSE: r = 0.31, p < 0.04 (control); r = 0.50, p < 0.01 (Alzheimers)	N/I	N/I	N/I	N/I
N/I	N/I	N/I	N/I	N/I
N/I	N/I	N/I	N/I	N/I
r = 0.56 (WHOQOL-OLD)	r = -.057 (EBADEP-A)	Exploratory	6 factors	43.60%
N/I	N/I	Confirmatory	2 factors	N/I
CCQ22: r = 0.945; MMSE: r = -0.679; CAMCOG: r = -0.653;CDR: 0.873; FAQ: r = 0.858; IQCODE: r = 0.769; NPI: r = 0.479 (p < 0.05).	N/I	N/I	N/I	N/I

N/I	N/I	Exploratory	4 Factors	74%
N/I	N/I	N/I	N/I	N/I
N/I	N/I	N/I	N/I	N/I
MMSE: Rho = 0.77; P = 0.001. Blessed Dementia scale (r = 0.98; P = 0.0001).	N/I	N/I	N/I	N/I
MMSE: r = 0.59; p < 0,001).	N/I	N/I	N/I	N/I
Depression (BDI) with Frequency r = -0.381; Obtained Pleasure r = -0.323.	N/I	Confirmatory	2 factors	N/I
N/I	N/I	N/I	N/I	N/I
N/I	N/I	N/I	N/I	N/I
MMSE: r = −0.66, p < 0.0001; CDT: r = −0.57, p < 0.0001.	N/I	N/I	N/I	N/I
N/I	N/I	Exploratory	4 factors (excluded sensory abilities and intiMCIy)	51.10%

N/I	N/I	N/I	N/I	N/I
N/I	N/I	N/I	N/I	N/I
N/I	N/I	Exploratory	4 factors	43.60%
69.6% (positive values) and 96.0% (negative values)	N/I	N/I	N/I	N/I
MMSE r = 0.59; p < 0,001.	N/I	N/I	N/I	N/I
N/I	N/I	N/I	N/I	N/I
N/I	N/I	N/I	N/I	N/I
N/I	N/I	N/I	N/I	N/I
N/I	N/I	N/I	N/I	N/I
GDS-15 r = 0.76; p < 0.001; d = 2.34; IC 95% = 2.09 – 2.59)	MMSE (r = 0.36; p < 0.001; d = 077; IC 95% = 0.57 – 0.97).	N/I	N/I	N/I

N/I	N/I	N/I	N/I	N/I
N/I	N/I	Confirmatory	4 factors	N/I
N/I	N/I	N/I	N/I	N/I
N/I	N/I	N/I	N/I	N/I
CAMCOG-R (r 1⁄4 .46, P < .01), MMSE scores (r 1⁄4 .58, P < .01), and IADL (r 1⁄4 .32, P < .01);FAQ scores (0.59, P < .01).	N/I	N/I	N/I	N/I
N/I	N/I	N/I	N/I	N/I
N/I	N/I	Exploratory	2 factors	48.10%
N/I	N/I	N/I	N/I	N/I
N/I	N/I	N/I	N/I	N/I
GDS-15 (rho 0.82) GDS-10 (rho 0.82) and GDS-4 (rho 0.81) v/s MADRS.	N/I	N/I	N/I	N/I
GDS-15:				

N/I	N/I	Exploratory	4 factors	48.90%
SAHLPAxPCAS: Rho = 0.82, p < 0.0001; NEUROPSI Total xPCAS: Rho = 0.73, p < 0.0001.	N/I	N/I	N/I	N/I
N/I	N/I	N/I	N/I	N/I
N/I	N/I	N/I	N/I	N/I
MMSE: 0.700–0.730; (p < 0.001); CAMCOG: 0.753–0.779; (p < 0.001)	N/I	N/I	N/I	N/I
Clock Setting, reading and drawing x MMSE r = 0.464, 0.389 and 0.227 (p ≤ 0.05); Clock Setting, reading and drawing x Block design r = 0.476, 0.361 and 0.202 (p ≤0.05);	Clock Setting, reading and drawing x CES-D r = -0.140, -0.064 and -	N/I	N/I	N/I
N/I	N/I	N/I	N/I	N/I
MMSE: Rho = 0.483, p < 0.001; Figure Memory test: Rho = 0.022, < 0.784; Verbal fluency: Rho = 0.360, p < 0.001; CDT: Rho = 0.383, p < .001; Digit span forward: Rho =	N/I	N/I	N/I	N/I
GDS-30: PPV 49% and NPV was 98%; GDS-15: PPV 51% and NPV was 97%.;GDS-10:PPV 46% and NPV 94%; GDS-4: PPV 415 and NPV 96% ;GDS-1: PPV 69% and NPV 90%	N/I	N/I	N/I	N/I
SF-36X WHOQOL-BREF: PF: r = 0.32*; RP: r = 0.34*; BP: r = 0.34; GH: r = 0.42*; VT: r = 0.51*; SF: r = 0.35*; RE: 0.23*; MH: 0.50*; *=p < 0.05.	N/I	N/I	N/I	N/I

N/I	N/I	Confirmatory	5 factors	N/I
N/I	N/I	Exploratory (PCA) and confirmatory	5 factors	N/I
N/I	N/I	N/I	N/I	N/I
N/I	N/I	N/I	N/I	N/I
MMSE X CDR 0: 0.12- 0.14, p = 0.098- 0.190; 0.5: 0.40-.46, p = 0.0001- 0.004; 1: .36-.45, p = 0.0001; 1: 0.36- 0.45, p = 0.0001. 2: 0.51- 0.53, p = 0.0001. CAMCOG X	PFAQn X CDR 0: - 0.14- 0.08, p.138-423; 0.5: - 0.35 -0.21, p =0.06- 0.13; 1: -	N/I	N/I	N/I
N/I	N/I	N/I	N/I	N/I
MEEM (r = 0.81; p < 0.0001); CAMCOG (r = 0.86; p < 0.0001); verbal fluency (r = 0.70; p < 0.0001).	QAFP (r = -0.67; p < 0.0001)	N/I	N/I	N/I
N/I	N/I	N/I	N/I	N/I
N/I	N/I	N/I	N/I	N/I
BDI and BHS scores were statistically significant (figures not revealead in the article)	N/I	N/I	N/I	N/I

SAOF (ADL: p = 0.42; Total: p = 0.001); role checklist´s number (ADL: p = 0.31;Total: p = 0.017)	N/I	N/I	N/I	N/I
N/I	N/I	N/I	N/I	N/I
Diagnosis of dementia: p < 0.001, MMSE: p = 0.047, and ADL: PFisher = 0.004.	N/I	N/I	N/I	N/I
Blessed scale: r = 0.96; p = 0.001; MEEM Spearman r = -0.72; p = 0.001	N/I	N/I	N/I	N/I
N/I	N/I	N/I	N/I	N/I
Beck Anxiety Inventory (ρ = 0.68, p < 0.001) and the State-Trait Anxiety Inventory (ρ = 0.61, p < 0.001.	N/I	N/I	N/I	N/I
CAMCOG: r = 0.82; MMSE: r = 0.74 (n = 82 subsample)	N/I	N/I	N/I	N/I
MoCA test, r = 0.76, p < 0.001	N/I	Confirmatory	3 factors	N/I
MMSE (0.7, 95% IC=0.6-0.8, p < 0.001) and FAQ (–0.7, 95% IC= –0.8-[–0.6], P < 0.001).	N/I	N/I	N/I	N/I
N/I	N/I	N/I	N/I	N/I

Rho = 0.387, p < 0.0001	N/I	N/I	N/I	N/I
N/I	N/I	N/I	N/I	N/I
With MMSE (MEEM) and FAB, education and age. DSM-IV (pre-diagnosed/known) dementia AD.	N/I	N/I	N/I	N/I
PQOL, MMSE, GDS, Cornell C-PQOL, CQOL, IADL, ADL, NPI, Cornell scale (by carers), and Beck Depression	N/I	N/I	N/I	N/I
DSM-IV following blinded neuropsychologist and geriatrician assessment	N/I	N/I	N/I	N/I
N/I	N/I	N/I	N/I	N/I
DSM-IV depression using the SICD	N/I	N/I	N/I	N/I
N/I	N/I	N/I	N/I	N/I
Significant spearman correlation p0.0001 level with domestic, complex and global GADL score and MMSE; MCI vs AD NINCDS-ADRA	N/I	Exploratory, principal axis factoring and othogonal factoring design	3: self-care ADLs (eigenvalue: 4.97), complex ADLs	1 33%, 2 13%, 3 7%
Mini-Mental State Exam (MMSE), Clock Drawing Test (CDT) Geriatric Depression Scale; CDR, MMSE	N/I	exploratory, principal axis factoring and oblique rotation	2	59%

CDR (r = 0.65, p < 0.001), age p = 0.016; DSM-IV, NINCDS-ADRDA (AD) MCI Petersen	N/I	N/I	N/I	N/I
N/I	N/I	N/I	N/I	N/I
CAMDEX 0.67 (sig), MMSE (NS), CAMCOG (NS), BOMFAQ depression scale 0.41 (sig); DSM-IV depression	N/I	linear factor analysis in a correlation matrix	3 factors	52.72% (varimax)
N/I	N/I	N/I	N/I	N/I
DSM-IV dementia	N/I	N/I	N/I	N/I
CC - COGTEL score and MMSE (r = 0.682, p < 0.001), schooling level (r = 0.604, p < 0.001)	N/I	N/I	N/I	N/I
DSM-IV using 10/66 protocol	N/I	N/I	N/I	N/I
N/I	N/I	N/I	N/I	N/I
MMSE; DSM-IV	N/I	N/I	N/I	N/I
GMS-AGE cat diagnosis by algorithm 'psychosis' or 10/66 algirithm dementia	N/I	N/I	N/I	N/I

N/I	N/I	N/I	N/I	N/I
N/I	N/I	N/I	N/I	N/I
N/I	N/I	N/I	N/I	N/I
Beck anxiety inventory, SRQ-20	MMSE, IQCODE, B-ADL	N/I	N/I	N/I
MMSE (sig); DSM-IV dementia	GDS	N/I	N/I	N/I
MMSE (sig), verbal fluency (sig), Clock drawing test (Sig); DSM-IV dementia, peterson criteria MCI	N/I	N/I	N/I	N/I
N/I	N/I	N/I	N/I	N/I
N/I	N/I	N/I	N/I	N/I
Age (sig for lower), education, sex, income level (sig for higher) (MWU/KW test)	N/I	N/I	N/I	N/I
MFT-(memories of figures test)DR (delayed recall) 0.84 ($p < 0.001$), RKc (remember-know coeffiICent) 0.71 ($p < 0.001$)	N/I	N/I	N/I	N/I

Metamemory in Adulthood scale (MIA) (sig)	HADS depression, HADS anxiety (sig MMQ ability)	N/I	N/I	N/I
dementia caseness (higher), depression caseness (higher for participants), WHO-DAS (total needs r =0.4-0.55) unmet needs (r =0.42-0.44)	N/I	N/I	N/I	N/I
N/I	N/I	N/I	N/I	N/I
MMSE (sig) CAMCOG memory subscale (Sig) CAMCOG total (sig); Clinical diagnosis AD and MCI	N/I	N/I	N/I	N/I
WHO-QUOL-Brev (sig)	positive and negative dimensions of the scale (r = 0.022, p =	Exploratory	11 factors	57.46%
N/I	N/I	Exploratory and confirmatory	EFA: 4 factors CFA: 3 and 4 factors	N/I
N/I	Control patients (sig in all domains and subscales)	Exploratory	4 factors	62.20%
Medical Outcomes Study (MOS) 36-item Short-Form Health Survey (SF-36). (sig, high correlation with all domains)	New York Heart association (NYHA) Classes I, II and III/IV	N/I	N/I	N/I
frailty (sig < 0.001); hospitalizations, worsening disability, ER visits, falls (all sig correlated with AMPI-AB)	N/I	N/I	N/I	N/I
DVAE (H/S-EAST: 0.13bτ= and CTS-1 subscales:0.15 - 0.37bτ=); caregiver burden (Zarit BI: 0.40bτ=); and depression (GDS: 0.32bτ	N/I	Exploratory and confirmatory	1 factor	0.454

N/I	N/I	Confirmatory	1 factor	N/I
correlation with education, when age was held constant, was r = 0.71, p < 0.001	N/I	N/I	N/I	N/I
N/I	N/I	N/I	N/I	N/I
N/I	N/I	Exploratory	1 factor	N/I
N/I	N/I	N/I	N/I	N/I
N/I	N/I	N/I	N/I	N/I
Rho = 0.47 (p < 0.001)	N/I	N/I	N/I	N/I
N/I	N/I	N/I	N/I	N/I
N/I	N/I	N/I	N/I	N/I
MMSE: Rho = 0.750 (p < 0.001)	N/I	N/I	N/I	N/I

N/I	N/I	N/I	N/I	N/I
MIA = 0.47 to 0.68 (ITEMS)	Anxiuos and depressive symptoms (-0.63, -0.54, respectively)	N/I	N/I	N/I
MMSE (rho = 0.85). PFAQ (rho = -0.67); Significant correlation with MMSE; predictive value in AD identification (83.3%)	N/I	N/I	N/I	N/I
schooling level and MMSE (0.481, p ≤ 0.001), ACE-R (0.484, p ≤ 0.001). The study compares cognitive measures (MMSE, ACE-R) among different groups (Controls, MCI,	N/I	N/I	N/I	N/I
N/I	N/I	Exploratory	5 factors identified, 4 retained	44%
Scores from different scales are compared at the same time, showing that all document disease progression	N/I	N/I	N/I	N/I
Loneliness and depresion (r = 0,665, p < 0,001). loneliness andsocial support (r = -0.576, p < 0.001).	N/I	Exploratory	1 factor	43.60%
N/I	N/I	N/I	N/I	N/I
GDS-15 and number of health problems: r = 0.189, p < 0.001. GDS-15 and functional performance: r = -0.204, p < 0.001.	N/I	N/I	N/I	N/I
Bender vs MEEM: r = -0.72<br>Bender vs CAMCOG: r = -0.75<br>Bender vs QAFP: r = -0.80;	N/I	N/I	N/I	N/I

K10 and SRQ-20 (ρ = 0.722, p < 0.001)	N/I	N/I	N/I	N/I
Supermarket: r = 0.632, p < 0.001<br>Animal: r = 0.438, p < 0.001	N/I	N/I	N/I	N/I
HADS Anxiety: r = 0.48<br>HADS Depression: r = 0.57<br>GDS: r = 0.60<br>FSS: r = 0.63<br>PFS-16: r = 0.73;	Cognitive Performance: r = -0.30	N/I	N/I	N/I
Significant positive correlation with GAI (rS = 0.413, p < 0.001) and GDS (rS = 0.444, p < 0.001).	Weak correlations with age, education, and gender.	N/I	N/I	N/I
Positive significant correlations with SF-12 and BMI.	N/I	Exploratory and Confirmatory	4 factors	Preventive Behavior: 26.61%<br>Food/Diet: 14.13%
N/I	N/I	Exploratory	5 factors	N/I
N/I	N/I	N/I	N/I	N/I
pain intensity reported by caregivers, except for "expressive eyes" (r: 0.106; p: 0.462).	N/I	N/I	N/I	N/I
N/I	N/I	N/I	N/I	N/I
MMSE and verbal fluency tasks, showing a negative association with lower education and cognitive ability.	N/I	Exploratory	2 factors	38%

N/I	N/I	Exploratory	3 factors	50%
K10 and SRQ - 20: ρ=0.722 (p < 0.001)	N/I	N/I	N/I	N/I
The PSI-16 showed significant correlations in the non-clinical group with other instruments such as the GDS-15 (0.534), MMSE (-0.240), and FAB (-0.403)	N/I	Confirmatory	1 factor	N/I
N/I	N/I	N/I	1 factor	N/I
N/I	N/I	N/I	N/I	N/I
N/I	N/I	Confirmatory	4 factors	N/I
B=0.3953, p = 0.0001) (controls vs demented); B =0.5544, p < 0.001) (severe vs mild-moderate dementia)	N/I	N/I	N/I	N/I
N/I	N/I	Confirmatory	5 factors	N/I
Religiosity AVE = 0.70; Spirituality α = 0.55.	N/I	Confirmatory	2 factors	N/I
N/I	N/I	Exploratory and Confirmatory	3 factors (items deleted 1,4,9 and 13)	EFa = 81%

GDS 15-ITEM: 0.92, p < 0.001; 0.88 (positive values) and 0.90 (negative values)	N/I	N/I	N/I	N/I
N/I	N/I	N/I	N/I	N/I
N/I	N/I	N/I	N/I	N/I
N/I	N/I	Exploratory	2 factors	65.30%
Depression scales (CES-D): Rho = 0.549, p < .01, Rumination (RSS): Rho = 0.618; p < 0.01; Experiential avoiding: Rho = 0.485; p < 0.01.	Psychologycal well-being: Rho = -0.699 (p < 0.01).	Exploratory	1 Factor	43.60%
life satisfaction scale of Diener (r = 0.623 p = 0.01,)	GDS (abbreviated) - 0.746 p = 0.01	principal components analysis	6 factors	factor 1 (31.7%) factor 2 7.1% factor 3 5.3%
MMSE, FAB, ACE-R; CDR, AD8, IADL pfeffer ADQ, T-ADLQ; DSM-IV dementia	Performance of control group on test	N/I	N/I	N/I
MMSE (r = 0,952p < 0,001), r = -0,822 with CDR, (r = -0,70 with ADLQ-Ch; r = -0,725 with PFAQ-Ch; r = 0,650 with IADL Scale) and cognitive changes (r = -0,633 with	N/I	N/I	N/I	N/I
N/I	N/I	Exploratory	3 factors	83.07%
DSM-III-R dementia and CDR rating	N/I	N/I	N/I	N/I

N/I	N/I	N/I	1 factor (model c)	N/I
With spouse: Total (r = 0.328) With children: Total (r = 0.447) With other: Total (r = 0.196); r^2 = 0.11 with spouse (11% of the shared variance).	N/I	Confirmatory	3	N/I
(correlation with MMSE): r = 0.83 (correlation with WCST): r = 0.678 (correlation with categorical fluency): r = 0.71	N/I	Exploratory	1 factor	41%
rho = 0.798, p < 0.001 (validity of the MoCA using the MMSE)	N/I	N/I	N/I	N/I
GDS-15: -0.29 to -0.56, WHOQOL: 0.41 to 0.46, PWI: 0.05 to 0.48, DJGLS-6: -0.29 to -0.51. Convergent validity varies between ethnic groups, being highest with	N/I	N/I	N/I	N/I
N/I	N/I	Confirmatory	2 factors	N/I
(0.717; 0.719; 0.677) Significant correlations with the personal,social, and overall well-being dimensions	No significant correlation with the self-esteem scale (r = 0.015; p = 0.895)	N/I	N/I	N/I
N/I	N/I	Confirmatory	MMSE: One factor, after excluding memory. MoCA: One	N/I
N/I	N/I	N/I	N/I	N/I
ICDI was used as the gold standard, with a prevalence of MDD of 3.6% in the sample.	N/I	Confirmatory	1 factor	N/I

N/I	N/I	N/I	N/I	N/I
N/I	N/I	N/I	N/I	N/I
N/I	N/I	Confirmatory	3 factors	N/I
Depression and dependency. High correlation between PC and PR, PF, BP scales; CM correlates with MH, V, ER, and PF. PC: Physical Component	N/I	N/I	N/I	N/I
Correlations of DJGLS-6 with GDS (0.48 to 0.62), BRCS (−0.36 to −0.52), PWI (−0.17 to −0.52).	N/I	Confirmatory	1 factpr	N/I
N/I	N/I	Confirmatory	1 factor model for most subscales, except for Orientation and	N/I
N/I	N/I	Confirmatory	6 factors	N/I
Non-Indigenous: PWI (−0.57), BRCS (−0.56)<br>Aymara: PWI (−0.24), BRCS (−0.24)<br>Mapuche: PWI (−0.48), BRCS (−0.40)	N/I	N/I	n	N/I
N/I	N/I	Confirmatory	All dimentions with exception of attention are unidemensional	N/I
N/I	N/I	N/I	N/I	N/I

SHS sig correlation pearson 0.512 (p≤0.01) Chile 0.394 (p≤0.01) Ecuador	N/I	confirmatory (polychoric correlation matrix)	1 factor	not stated. Partial invariance in one factor
N/I	N/I	N/I	N/I	N/I
BAS-DEP/EBAS-DEP: r = 0.87 p≤ 0.001; GDS/ BAS-DEP r = 0.63 (p≤.001);EBAS-DEP: r = 0.59 (p≤.001).	N/I	principal Components Analysis	2 factors	28.80%
N/I	N/I	Exploratory	2 factors	37.30%
MMSE: r = 0.596, p < 0.001	N/I	Exploratory	4 factors	61.65%
N/I	N/I	Exploratory	4 Factors	55.90%
MMSE: 0.755; 95% IC [0.687, 0.811]). Diagnosis of the Memory Clinic K = of 0.69; 95% IC [0.59, 0.80].	N/I	N/I	N/I	N/I
N/I	N/I	N/I	N/I	N/I
N/I	N/I	Exploratory (GDS-15 and GDS-5)	GDS-15 = 2 Factors; GDS-5 = 1 factor	GDS-15 = 37.1%; GDS-5 = 49.5%
N/I	N/I	Exploratory	4 factors	58.60%

N/I	N/I	Exploratory	2 factors	42.60%
N/I	N/I	N/I	N/I	N/I
N/I	N/I	PCA of residual components of rasch analysis	1 factor	40.10%
HADS anxiety, HADS depression, GHQ-12, Beck, multidimensional fatigue inventory (MFI) EORTC european organisation organistion for research and treatment (all sig	N/I	confirmatory	2 factors	N/I
N/I	N/I	Exploratory	1	77.93%
N/I	N/I	N/I	N/I	N/I
Zarit (r = 0.28), NPI (r = 0.37), Mini-Mental (r = −0.22), Yesavage (r = −0.25); The regression analysis showed that the AQ-D score is assoICated with other measures	N/I	Confirmatory	4 factors	N/I
DDT-Pro scores are related to other diagnostic criteria at the same point in time (DSM-5, TMF, DRS-R98).	N/I	N/I	N/I	N/I
Correlation between subcortical and cortical scores: r = 0.39, p < 0.001. Correlation between non-mnesic and mnesic factors: r = 0.42, p = 0.000; ICC with MoCA:	N/I	Exploratory	2 factors	First factor: 0.40. Second factor: 0.13. Cumulative:
MCS-10 with A-MMSE: −0.43 (p < 0.001).<br>MCS-10 with MoCA: −0.38 (p < 0.001).	N/I	Exploratory and Confirmatory	1 factor	45.3% from EFA and 50.9% from CFA.

Inverse correlation with DRS-R-98: Spearman's Rho = -0.698 (total), -0.829 (dementia subgroup).	N/I	N/I	N/I	N/I
N/I	N/I	Confirmatory. (Tested three alternative factor structures)	8 factors	N/I
N/I	N/I	Exploratory	2 factors	39.40% (22.06% and 17.34% for each factor).
MMSE: r = 0.92, p < 0.01; CDR: r = –0.63, p < 0.01.	N/I	N/I	N/I	N/I
The study provides evidence for concurrent validity by comparing the IFS and FAB scores with the MCI diagnosis.	N/I	N/I	N/I	N/I
High correlation with verbal and visual memory measures (e.g. CERAD r = 0.85).	N/I	N/I	N/I	N/I
PDQ-39 (sig) PD scale (hoehn and Yahr)(Sig) Schwab and england scale (sig)	N/I	N/I	N/I	N/I
N/I	N/I	Confirmatory	3 factors	43.60%
N/I	N/I	Confirmatory	2 factors	N/I
N/I	N/I	Exploratory and confirmatory	EFA: 4 Factors; CFA: bifactor model	N/I

Cuba r = (-0.036, 0.41 and -0.49); Dominican republic r = (-0.43, 0.48 and -.032); Peru Urban r = (-0.45, 0.46 and -0.41); Peru rural r = (-0.20, 0.37 and -0.17); Puerto Rico r	N/I	principal components factor analysis	3 Factors (Dominican republic, Perú, Puerto Rico and Venezuela) 2	36.4-45.8% (2 factors)
N/I	N/I	Confirmatory	See suplementary material of the paper	N/I
PDQ-39 (r = 0.62), SCOPA-MS (r = 0.82), ICSI-PD (weak r = 0.57), HADS (r = 0.61-62), HY PD staging (r = 0.45), disease duration (r = 0.31).	N/I	exploratory - principal components analysis	one (after parallel analysis) reliability of factor 0.89	48.58
psychosis clinical diagnosis sig high (r_S = 0.56)	SCOPA-Cog (r = -0.12), ICSI-PD (rS ≤ 0.30)	Exploratory, principal components analysis, rotation	2 factors	58.50%
N/I	N/I	Exploratory and confirmatory. Subsequent Mokken analysis	PCA (two factors cuba Dom Rep) others one. Final pooled	75.01 variance eigenvalue 9.01
effect of age, education, sex and geographical region evaluated (see summary)	N/I	N/I	N/I	N/I
DSM-IV dementia and CDR severity, MADRS (depression) score 18+	N/I	Exploratory (EURO-D only)	2 factors (EURO-D)	44% (EURO-D) 36% F1 8% F2
N/I	N/I	Exploratory	1 factor	More than 83% of total variance.
WHOQOL-OLD and Subjective Well-Being Scale: r = 0.624, p ≤ 0.001.	WHO-QOL-OLD and GDS: r = –0.569, p ≤ 0.001.	Exploratory	6	65%
N/I	N/I	N/I	N/I	N/I

N/I	N/I	N/I	N/I	N/I
*Only for Perseveration: MMSE r = 0.63 p < 0.05; MoCA r = 0.74 p < 0.05	N/I	N/I	N/I	N/I
N/I	N/I	principal Components Analysis	4 factors	N/I
Diener Well-being Scale (r = 0.11, p = 0.01)	Geriatric Depression Scale (GDS) (r = -0.20, p = 0.01)	Exploratory and confirmatory	EFA = 1 factor; CFA = 1 factor	EFA = 77%
N/I	N/I	N/I	N/I	N/I
Beck´s Scale (r = 0.41-0.75, p < 0.01) CESD-20 (r = 0.28-0.59, p < 0.01)	N/I	Exploratory	2 Factors	48.80%
N/I	N/I	Exploratory and confirmatory	EFA: 5 factors; CFA: 4 factors	EFA: 63.124%; CFA: 63.969
SF-12 (5 item physical health): Rho = 0.32, p = 0.001	CESD-7 (depression): Rho = -0.38, p = 0.001	principal Components analysis and Confirmatory analysis	3 factors	59.06%
MMSE x ROSA cognition and communication Rho = 0.469 (p < 0.001);	IQCODE cognition x ROSA cognition Rho = -0.503 (p < 0.001);	N/I	N/I	N/I
N/I	N/I	N/I	N/I	N/I

N/I	N/I	N/I	N/I	N/I
Beck depression inventory; ICD-10, DSM-IV	N/I	N/I	N/I	N/I
iadl, adl, mmse, sf-36, charlson indeex	N/I	N/I	N/I	N/I
clinical diagnosis by a psychiatrist DSM-IV	N/I	principal components analysis, orthogonal varimax	GDS = 8 factores; CES-DR = 9 factors	GDS = 53.5%; CESDR = 57.9%
Suicidal Ideational Scale (EIS) (sig), (Qs. when is one old? when is one too old? (sig)); Moderate association with Philadelphia Geriatric Center Morale Scale, APE	N/I	exploratory PCA and varimax rotation	EAE-O (orig) 3 EAE-A (adapt) 4	EAE-O (orig)53.1% EAE-A (adapt) 60.8
N/I	MMSE, MNA (nutriICon, physical function) r = 0.39 , GDS r =	N/I	N/I	N/I
ARS Loneliness Multiphase Inventory IMSOL (religious coping with loneliness subscale) (sig); Spiritual Wellbeing Scale, EBE (relationship with God	N/I	Exploratory	1 factor (B-RCOPE), 1 factor (ARS), 2 factors (EBE)	49.5% (B-RCOPE), 70.2% (ARS), 59.3% (EBE)
GDS-30 (but correlation not stated)	N/I	Exploratory	1	25%
N/I	N/I	Confirmatory	1	49%
N/I	N/I	N/I	N/I	N/I

N/I	N/I	Confirmatory	4 factors	≥ 75%
MMSE: ρ = 0.830 (P < 0.001)	N/I	N/I	N/I	N/I
MMSE: r = 0.681 [p < 0.001]; also correlated with MoCA-E and ADLs/IADLs.	N/I	N/I	N/I	N/I
no loneliness vs high/very high loneliness groups (t = -21.5, p < 0.001).	N/I	Exploratory and Confirmatory	2 factors	Total: 67,2% (Factor 1: 51,7%, Factor 2: 15,5%).
−0.783 with subjective well-being and −0.569 with quality of life	N/I	N/I	N/I	N/I
Moderate to low correlations withsocial support groups.	N/I	Exploratory	2 factors	39.40%
N/I	Women: F1 and F2 correlation = 0.51, F3 and F4 correlation = 0.74	Confirmatory	4 factors	72%
correlates MGH-SFQ scores with age and depression	N/I	Confirmatory	1 factor	N/I
z-MMSE X z-LCT: r = 0.597 (p < 0.001); z-MMSE X z-MoCA: r = 0.698 (p b 0.001); z-LCT X z-MoCA: r = 0.501 (p < 0.001).	N/I	N/I	N/I	N/I
SWLS (r = 0.826, p < 0.01 [IC95%: 0.78, 0.93]).	GDS-5 (r = -0,598, p < 0,01 [IC95%: -0,51,-0,81]).	Confirmatory	1 factor	79.90%

SWLS: r = 0.56, p < 0.01 [IC95%: 0.46, 0.79].	GDS-5: r = -0.48, p < 0.01 [IC95%: -0.37, -0.73].	Exploratory and Confirmatory analysis	1 factor	EFA: 55.7%
sisl: r = 0.898, p < 0.01 [IC 95%: 0.87, 0.96]); brcs: r = 0.992, p < 0.01 [IC 95%: 0.98, 0.99].	GDS-5: r = -0.584, p < 0.01 [IC 95%: -0.49, -0.79].	Confirmatory	1 factor	N/I
SWLS (r = 0.500, p < 0.01, IC 99% 0.42-0.73); CHS (r = 0.360, p < 0.01, IC 99% 0.26-0.62)	GDS-5 (r = -0.309, p < 0.01, IC 99% -0.21 – -0.57).	Confirmatory	1 factor	N/I
N/I	N/I	N/I	N/I	N/I
MMSE: r = 0.85, p < 0.001	N/I	N/I	N/I	N/I
r = 0.79; p < 0.01	N/I	N/I	N/I	N/I
Cognitive status CDR: 0.65 [95% confidence interval: 0.32-0.81], P < 0.001; Functional status: PFAQ: 0.53 [95% confidence interval: 0.12-0.67], P < 0.001; BCTs (r = 0.79	N/I	N/I	N/I	N/I
age, education years and reading activity. Cognitive decline on clinical interview based on daily activities. No gold standad assessment	N/I	N/I	N/I	N/I
MMSE, PFAQ; MMSE was used as gold standard for cognitve impairment	N/I	N/I	N/I	N/I
N/I	N/I	Confirmatory	1 factor	N/I

MMSE (sig not reported); DSM-IV dementia	N/I	N/I	N/I	N/I
MMSE r = 0.73	N/I	N/I	N/I	N/I
N/I	N/I	N/I	N/I	N/I
The Pearson correlation coefficient between M@T and biomarkers was: M@T and β-amyloid: 0.66 (95% IC: 0.57–0.74) M@T and t-Tau: -0.77 (95% IC: -	N/I	N/I	N/I	N/I
RUDAS-PE/MMSE (ρ = 0.86; SD: 0.14, IC 95%), RUDAS-PE/IFS (ρ = 0.87; SD: 0.09, IC 95%), RUDAS-PE/PFAQ (ρ = 0.83; SD: 0.27, IC 95%), and RUDAS-PE/CDR (ρ =	N/I	N/I	N/I	N/I
High correlation between MMSE and MoCA	N/I	N/I	N/I	N/I
N/I	N/I	N/I	N/I	N/I
N/I	N/I	Confirmatory	6 factors	N/I
Anxiety: r = 0.72; $p < 0.01$. depression: r = 0.53; $p < 0.01$.	N/I	Confirmatory	1 factor	N/I
N/I	N/I	N/I	N/I	N/I

N/I	N/I	Confirmatory	1 factor	N/I
Memory and Attention Test (r = 0.52); MAT: r = 0.52, p = 0.00 Pfeiffer: r = -0.49, p = 0.00 MMSE: r = 0.38, p = 0.00 Barthel: r = 0.22, p = 0.00	N/I	Exploratory	2 factors	62%
DJGLS and UCLA-3: r = 0.88, p < 0.01; DJGLS and Life Satisfaction: r = -0.71; DJGLS and Depression: r = 0.76; DJGLS and Resilience: r = -0.72	N/I	Confirmatory	2 factors	N/I
High correlation with BADL, IADL, MMSE, ACE, CDR, and GDS, except with ADAScog. MMSE (0.726), ACE (0.839), and CDR (0.828).	N/I	N/I	N/I	N/I
Free recall: FCSRT-Picture version vs RUDAS-PE= 0.85; vs PFAQ= 0.81; and vs CDR = 0.92 Total recall: FCSRT-Picture vs RUDAS-PE = 0.89; vs PFAQ= 0.88; and vs CDr = 0.91	N/I	N/I	N/I	N/I
N/I	N/I	Confirmatory	2 factors	N/I
resilience (r = 0.86); life satisfaction (r = 0.82) Depression (r = -0.66).	N/I	Confirmatory	1 factor	N/I
Life satisfaction (r = 0.88, p < 0.01), depression (r = -0.56, p < 0.01)	N/I	Confirmatory	Unidimensional	Average Variance Extracted (AVE) =0.50
Autonomy: rs = 0.13, p < 0.05 Social involvement: rs = 0.16, p < 0.01; QLI for autonomy and social involvement.	N/I	Confirmatory	6	N/I
N/I	N/I	Confirmatory	2 factor	N/I

MMSE: r = .87.	N/I	N/I	N/I	N/I
N/I	N/I	Exploratory	4 factors	61.38%
MMSE and CDT correlation: r = 0.549, p < 0.0001	N/I	N/I	N/I	N/I

Fit indexes (Factor Analysis)	Rasch model	Other
N/I	N/I	Diagnostic Accuracy was 63%, The Positive Predictive Value was 61% while the Negative Predictive Value was 64%.
N/I	N/I	Translation and adaptation
N/I	N/I	Time to complete instrument: 30 minutes. Likelihood ratio of a positive test, 3.52; and likelihood ratio of a negative test, 0.69.
N/I	N/I	test duration 2-5 minutes. Age and education affected TYM score (lower at higher age and with lower education).
N/I	N/I	Translation, adaptation and thurston scaling exercise for assessing interval properties was completed.
N/I	N/I	N/I
N/I	N/I	N/I
N/I	N/I	not related to education (MMSE was related)
N/I	N/I	N/I

N/I	N/I	N/I
N/I	N/I	N/I
N/I	N/I	N/I
N/I	N/I	ANOVA showed significant differences in MoCA total score based on educational level: F (2, 222) = 21.14; p < 0.01 /Correction for Multiple
N/I	N/I	Content validity was assessed through expert judgement, and areas for improvement in some items were identified and approved by the experts. The
N/I	High discrimination: Education (3.75) Occupational	N/I
N/I	N/I	N/I
NFI, NNFI, CFI: Acceptable values ≥ .90 RMSEA: Acceptable value < .08.	N/I	N/I
CFI: 0.937 TLI: 0.908 RMSEA: 0.091 90% IC of RMSEA: 0.065–0.118	N/I	
CFI: 0.98 (original model). TLI: 0.97 (original model). RMSEA: 0.05	N/I	N/I

N/I	N/I	The study provides an analysis of the ROC curve to evaluate the diagnostic accuracy of the ACE-III in detecting cognitive decline in DTA and DFT-vc, highlighting its
N/I	N/I	Time to complete instrument: 5.3–15.2 minutes (n = 53)
N/I	N/I	N/I
N/I	N/I	Multiple linear regression showed that the variables schooling (b= 0.263 p = 0.009, B= 0.983) and MMSE score (b= 0.516 p = 0.000, B=
N/I	N/I	N/I
N/I	N/I	There were positive relations between Cognitive Reappraisal and life satisfaction and positive affect; and there were negative relations with depression.
N/I	N/I	Translation and cultural adaptation
N/I	N/I	Takes about 5-7 minutes to complete
N/I	N/I	Positive predictive value 10.5%, negative predictive value 98. I%, and overall misclassification rate 38.5%
N/I	N/I	Translation and cross cultural adaptation. The mean time for administering the ANU-ADRI was 25 (±5) minutes.

N/I	N/I	A poor agreement between MMSE-ad and MMSE-mo was observed in 1-4 years degree (K=0.14) and in 5-8 years degree (K= 0.18) groups.The agree-
N/I	N/I	Negative correlation exists between age and scores: individuals of 1-4 (r = -0.298, p < 0.01 and r = -0.212, p < 0.05), for ad-MMSE and mo-MMSE,
N/I	N/I	Cultural adaptation and translation
N/I	N/I	N/I
N/I	N/I	The Behavioral Assessment of the Dysexecutive Syndrome item that best discriminated controls from patients was the Modified Six Elements - adapted
N/I	N/I	73.8% positive predictive value, and 100% negative predictive value.
N/I	N/I	Translation. It takes 15 minutes to be administered.
N/I	N/I	N/I
MLQ-P: RMSEA = 0.010 (0.00-0.083); SRMr = 0.016; TLI=1.00; CFI=1.00. MLQ-S: RMSEA =	N/I	N/I
N/I	N/I	Correlation of CCQ-22: CCQ8: r = 0.945; MMSE: r = -0.665; CAMCOG: r = -0.646;CDR: 0.859; FAQ: r = 0.872; IQCODE: r = 0.824; NPI: r = 0.475 (p < 0.05).

N/I	N/I	Transcultural adaptation
N/I	N/I	N/I
N/I	N/I	Translation and adaptation. Test-retest results are mentioned, but without the t-student coeffICents.
N/I	N/I	N/I
N/I	N/I	N/I
Frequency scale ($\chi 2/df = 2.57$; CFI = 0.728: GFI= 0.627; RMSEA = 0.07; p[RMSEA < 0.05] <	N/I	N/I
N/I	N/I	The average time for the Timed Up and Go Test was 10.0 (SD=3.2)
N/I	N/I	N/I
N/I	N/I	Application time of SKT is 10 to 15 minutes,
N/I	N/I	N/I

N/I	N/I	Time to complete instrument: 2 minutes
N/I	N/I	intra: K = = 0.91; inter: K = = 0.77 (0-3 score normal); intra: K = = 0.71; inter: K = = 0.56 (4-5 score abnormal)
N/I	N/I	N/I
N/I	N/I	N/I
N/I	N/I	Time to complete instrument:30 to 40 minutes
N/I	N/I	N/I
N/I	N/I	N/I
N/I	N/I	Translation, back translation, pilot study and jusgement (technical review of the semantic)
N/I	N/I	Normative data
N/I	N/I	ANOVA performance among three groups [F(2; 202) = 23,42; p < 0,001; d =0,83; IC 95% = 0,55 – 1,18]; Post-Hoc (Teste de Tukey HSD) (Community dwelling

N/I	N/I	Semantic analysis and focus group
(CFI = 0.910, RMSEA = 0.060, SRMR = 0.073),	N/I	Translation, back translation and cultural adaptation. Internal consistency of the items.
N/I	N/I	Takes about 5 minutes to complete. Translation and cultural adaptation
N/I	N/I	N/I
N/I	N/I	N/I
N/I	N/I	N/I
N/I	N/I	Translation
N/I	N/I	MMSE scores were assoICated with age ($r = -0.41$, $p < 0.001$) and schooling ($F=12.69$, $p < 0.001$).
N/I	N/I	N/I
N/I	N/I	Results for othet cut-off points are available for GDS-15 and GDS-10

N/I	N/I	N/I
N/I	N/I	N/I
N/I	N/I	Cut-off points related with educational background
N/I	N/I	N/I
N/I	N/I	N/I
N/I	N/I	Translation and Cross-cultural analysis
N/I	N/I	N/I
N/I	N/I	Schooling: Rho = 0.654, $p < 0.001$
N/I	N/I	N/I
N/I	N/I	N/I

no constraints: χ2 = 9553.88; p < 0.001; (df) = 1600; AIC = 10,553.88; (GFI) = 0.91; (NFI) = 0.90; TLI	N/I	N/I
Confirmatory: All three models had a GFI more than 0.90 and a Bentler-Bonett NFI and a NNFI	N/I	N/I
N/I	N/I	N/I
N/I	N/I	N/I
N/I	N/I	N/I
N/I	N/I	N/I
N/I	N/I	N/I
N/I	X2 = 215.96, df = 90, p < 0.001	N/I
N/I	“death and dying” item: χ2 = 51.72, p = 0.00012. "sensory	N/I
N/I	N/I	N/I

N/I	N/I	N/I
N/I	N/I	N/I
N/I	N/I	It takes 2.3 ± 0.1 minutes.
N/I	N/I	N/I
N/I	N/I	N/I
N/I	N/I	N/I
N/I	N/I	N/I
Bentler Comparative Fit Index = 0.96, Root Mean Square Error of Approximation = 0.09	N/I	N/I
N/I	N/I	N/I
N/I	N/I	N/I

N/I	N/I	between scales depressive symptoms K = 0.40 ($p < 0.001$); intensity K = 0.42.
N/I	N/I	N/I
N/I	N/I	Adaptation also described. Normative values listed for age and education
N/I	N/I	N/I
N/I	N/I	N/I
N/I	N/I	first testing, mean time 43 ± 9.4, range 23-90 minutes; overall mean 68.3 ± 12.9, range 36-96 points. Retest mean time 40.5 ± 8.9, range 24-90 minutes; overall
N/I	N/I	N/I
N/I	N/I	139 initial items evaluated. Highest third =46. Two rounds of expert dimension allocated resulted in agreement, 8 dimensions allocated using clinical
N/I	N/I	factor analysis was used to combine the scales. They started with the Katz and Lawton scales combined and developed a modified combined assessment.
N/I	N/I	N/I

N/I	N/I	years of schooling (r = –0.33, p = 0.021)
N/I	N/I	no significant association withsocial class or education
N/I	N/I	Results are also given for depression and dysthymia combined sens 79.92%, spec 78.29%, AUC 0.84 cut point 5/6 from 15
N/I	N/I	Average administration time of the MoCA was 12.4 ± 1.9 min. Education r = 0.332 (p < 0.001); Age r = –0.191
N/I	N/I	N/I
N/I	N/I	Translation and back translation described. Completed by committee and piloted in different age groups prior to study commencement
N/I	N/I	Regression model. MMSE score assoICated with sex, age, education and income adjusted for dementia
N/I	N/I	Cultural adaptation, by committee and pilot with 100 carers, translation and back translation described
N/I	N/I	positive predictive (PPV) and negative predictive values (NPV) were 70% and 88%, respec-tively.
N/I	N/I	N/I

N/I	N/I	Scores and subscores compared by educational level
N/I	N/I	N/I
N/I	N/I	N/I
N/I	N/I	Sig association GAI and GAI SF and SRQ. No association with other measures of covergent and disrICminant validity (MMSE BADL age education)
N/I	N/I	STADP score was affected by age and education
N/I	N/I	CAMDEX performed better than MMSE, clock drawing test and verbal fluency particularly in identifying MCI
N/I	N/I	Floor and ceiling effects of individual questions identified and adapted. Translation and back translation completed
N/I	N/I	Correlation between both scales Rs=0.46 ($p < 0.01$)
N/I	N/I	Details of forward and back translation tabulated.
N/I	N/I	N/I

N/I	N/I	Translation/back-translation process outlined.
N/I	N/I	The mean total needs identified by participants, caregivers and interviewers were, respectively: 4.9 (sd 3.6), 4.7 (sd 4.0) and 3.8 (sd 2.7). mean total of unmet
N/I	N/I	pilot phase and translation is described though not in detail
N/I	N/I	ROC curve analyses indicated high accuracy to differentiate NC from AD patients, and, moderate accuracy to differentiate NC from MCI.
N/I	N/I	Positive SRC was higher in older adults with religion ($p = 0.002$), good perceived health ($p < 0.001$), formal education ($p < 0.001$)living in own home ($p <$
4 factor model - factorial weights = 0.83-0.98; $\chi 2$ /df = 3.47; CFI = 0.99; TLI = 0.98; RMSEA = 0.08	N/I	N/I
N/I	N/I	Development process described in detail. The Scale of Relevance and Clarity (Alexandre and ColuIC 2011) was used to arrive at two independent and speICfic
N/I	N/I	N/I
N/I	N/I	Med AMPI-AB score 5 (3–8). Mean time to complete the AMPI-AB 05:44 ± 02:42 min. Survival analysis.
WRMr = 0.985, RMSEA = 0.056, CFI=0.967 and TLI=0.969	N/I	N/I

RMSEA = 0.051; 90 % IC: 0.047-0.055), final abridged RMSEA = 0.058; 90 % IC: 0.053-0.064 no	N/I	a shortened scale of 20 items is presented with acceptable adjustment indices
N/I	N/I	Translation and back translation processes are described as well as necessary adaptations and rationale. N-42 aged ≥ 60 mn score on BNIS 38.3 (SD 5,6). ≤4y
N/I	N/I	Guttman scaling method - coeffiICents reproduICbility (98%), scalability (84%) minimum marginal reproduICbility (87%).
RMSEA = 0039 CFI = 0.982 TLI = 0.983	N/I	Diagnostic accuracy calculated post-hoc based on known depression diagnosis
N/I	N/I	N/I
N/I	N/I	N/I
N/I	N/I	N/I
N/I	N/I	Normative data
N/I	N/I	N/I
N/I	N/I	N/I

N/I	N/I	N/I
N/I	N/I	N/I
N/I	N/I	N/I
N/I	N/I	N/I
N/I	N/I	N/I
N/I	N/I	N/I
$\chi^2 = 241.89$, gl = 170; $p < 0.001$ RMSEA = 0.056 CFI = 0.971 TLI = 0.967	N/I	N/I
N/I	N/I	translation and adaptation
N/I	N/I	No significant differences in scores by gender ($t(57.143) = -1.493$, $p = 0.141$).
N/I	N/I	N/I

N/I	N/I	N/I
N/I	N/I	N/I
N/I	N/I	N/I
N/I	N/I	Translation and adaptation
RMSEA = 0.037<br>CFI = 0.977<br>GFI = 0.953<br>NFI = 0.904<br>NNFI = 0.973	N/I	Discriminant validity analysis observing differences by BMI and comorbidities.
RMSEA = 0.030; TLI = 0.959; X2 = 151.590<br>p ≥ 0.05	N/I	The importance of cultural adaptation and the face-to-face interview method to include older adults with low education levels is emphasized. Limitations of the
N/I	N/I	Highlights the lack of valid and reliable instruments for measuring elder abuse in the Brazilian context.
N/I	N/I	N/I
N/I	N/I	The study emphasizes the differences in cognitive performance among groups, indicating the ACE-R's utility in differentiating between types of
N/I	N/I	Reliability Indicators:<br>H Index: Factor 1 = .82, Factor 2 = .86.<br>FDI: Factor 1 = .90, Factor 2 = .93.<br>SR (Score Reliability): Factor 1 =

N/I	N/I	N/I
N/I	N/I	N/I
CFI = 0.96, TLI = 0.95, RMSEA = 0.03 (0.03–0.06), SRMR = 0.07	N/I	Correlations between age, education level, and PSI-16 scores, finding significant correlations in the non-clinical group but not in
N/I	Infit Mean Square of 0.99 (SD = 0.14). Outfit Mean Square is 1.04	A DIF (Differential Item Functioning) analysis by sex, age, and education was conducted. It was found that MMSE scores vary significantly across these groups,
N/I	N/I	N/I
X2 = 1407.47 (p < 0.01); RMSE = 0.056; CFI = 0.84	N/I	N/I
N/I	N/I	N/I
X2 = 575,647; p < 0,01; RMSEA = 0,059; CFI=0,956; AGFI= 0,904; NNFI=0 ,95;	N/I	Time to complete instrument: 30-40 minutes
TLI = 0.95; CFI = 0.96; RMSEA = 0.15 (IC: 0.15-0.62)	N/I	N/I
X2[gl=32=110.130]; p < .001; CFI=.989; TLI=.984; RMSEA = .079),	N/I	N/I

N/I	N/I	K = coefficiente between GDS 15-item and 5-item: 0,78; p < 0,001.
N/I	N/I	Adaptation of the questions
N/I	N/I	Comparison with the MMSE: sensitivity 84.6% (95% IC; 54.6-98.1); specificity of 58.5% (95% IC; 42.1-73.7); AUC 0.82 (95% IC; 0.70-0.95).
N/I	N/I	The Lin's concordance coeffiICent between US-based Scoring algorithms and Chilean-SpeICfic were high for both MCS (q = 0.80; 95 % IC: 0.79–0.81)
N/I	N/I	N/I
N/I	N/I	Cut points on MUNSCH for happiness levels suggested. Median normative values for marital status and age bands given
N/I	N/I	1.29 ± 4.16 min (range: 5.27–20.25)
N/I	N/I	adaptation and translation of the chilean version is mentioned, and that the full document is available from the authors
N/I	N/I	N/I
N/I	N/I	Education level appeared to affect ROC curve results for MMSE and PFAQ - lower in lower education

χ2 = 9.983; CFI = 1.00; NFI = 0,98; GFI = 1.00; AGFI = 1.00; RMRS = 0.02; RMSEA = 0.012	N/I	N/I
CFI = 0.935 (Spouse), CFI = 0.996 (Children), CFI = 0.908 (Others). RMSEA = 0.044	N/I	N/I
N/I	N/I	N/I
N/I	N/I	N/I
N/I	Chi-square fit (χ²): χ²(16) = 26.29, p = 0.050 IR: M= -0.780 SD= 1.152	N/I
CFI=0.984; RMSEA = 0.102 IC95% [0.081 –0.125] and c2=105.890; p = 0.000	N/I	N/I
N/I	N/I	In general, 100% of the experts considered the scale a good instrument regarding the number of items, consistency, and relevance, and none suggested
MMSE: RMSEA = 0.054, CFI = 0.98, TLI = 0.93, SRMR = 0.030. MoCA: RMSEA = 0.000, CFI = 1.00, TLI	N/I	N/I
N/I	N/I	N/I
CFI: 0.99 TLI: 0.98 RMSEA: 0.04 [90% IC: 0.02–0.06].	N/I	N/I

N/I	N/I	Ordered logistic regression for RUDAS items with schooling as a predictor. Significant for the visuoconstruction item (p = 0.001, Or = 1.147).
N/I	N/I	Youden's Index = 0.899
All models showed CFI ≥ 0.95<br>All models showed TLI ≥ 0.95<br>All models had	N/I	The study explored modification indices, highlighting the correlation between items 1 and 10 in thesocial support factor.
N/I	N/I	N/I
$\chi2$ (df)= 4.99 (4)<br>RMSEA = 0.02<br>CFI= 0.99<br>RMSr = 0.01	DJGLS-6 did not fit the Rasch model ($\chi^2(24)$ = 170.46, P < .001). No DIF	N/I
Orientation: RMSEA = 0.032, CFI = 0.995, TLI = 0.994<br>Attention: RMSEA = 0.062, CFI = 0.983,	N/I	IRT analysis reveals that, overall, the revised versions of the subscales of the ACE-III show better fit and discrimination compared to the original versions.
CFI: 0.969<br>TLI: 0.961<br>RMSEA: 0.060<br>SRMR: 0.075	N/I	Content Validity, semantic validaty
N/I	PSI: 0.68<br>Fit: Unidimensional, no DIF except for item 4	N/I
CFI: Overall 0.945;<br>Orientation 0.995;<br>Attention 0.983;<br>Memory 0.958;<br>Language 0.968;	N/I	IRT: Attention subscale is the only one with a clear misfit (RMSEA ≥ 0.08). The other subscales have acceptable RMSEA values
N/I	N/I	Translation and content validity expert judgment

Comparative Fit Index CFI = 0.995; TLI = 0.993; RMSEA =0.061)	N/I	pilot phase is described
N/I	N/I	Biserialcorrelation coeffiICents: Rpbis=0.29-0.61 (GDS-15)
N/I	Items adjustemnt scores BAS-DEP and EBAS-DEP 0.5 - 1.5. Mean	N/I
N/I	N/I	Construct reliability= 0.700
N/I	20 of the 21 items fit the Rasch model (item p3 excluded).	N/I
N/I	N/I	cultural adaptation and pilot study
N/I	N/I	N/I
N/I	N/I	N/I
N/I	N/I	Mosier contruct validity: GDS-15 = 0.87; GDS-5 = 0.83
N/I	N/I	N/I

N/I	N/I	N/I
N/I	Wolfe and Smith model of rasch analysis. Reliability 0.80, measure	N/I
N/I	Rasch analyses category function, item and person fit, differential	Translation and adaption is described
CFI 0.983, RMSEA 0.044, AIC 56.878, NFI 0.978, GFI -0.993 (all ages, two factor model). Age 51-60	N/I	N/I
N/I	N/I	Cultural and Semantic Adaptation: Modifications made to improve understanding by older adults.
N/I	N/I	N/I
RMSEA = 0,06 (IC 90%: 0,06-0,07) CFI = 0,97 TLI = 0,97	N/I	N/I
N/I	N/I	cluster analysis to differentiate patients with SSD from non-delirium
coefficient of determination: 0.96 CFI: 0.95 TLI: 0.93 RMSEA: 0.06	N/I	The relationship between Hoehn and Yahr stage and total PD-CRS score was significant ($p = 0.012$) after adjusting for age and years of education.
CFA: $\chi^2 = 778.9$, df = 90, $p < 0.001$, χ^2/df ratio = 8.6, RMSEA = 0.06, CFI = 0.94, TLI = 0.93, SMSR = 0.03.	N/I	'Differential Item Functioning (DIF): Kendall's tau values by gender for the MCS-10 ranged from 0.02 to 0.19, indicating no DIF for this instrument.'

N/I	N/I	translation and face validity.
RMSEA = 0.067; CFI = 0.935; TLI = 0.926	N/I	N/I
N/I	N/I	Expert judgment was used for item adaptation. Items 11, 12, 16, and 20 were removed for not meeting the adequate factor loading (below 0.35).
N/I	N/I	N/I
N/I	N/I	N/I
N/I	N/I	N/I
N/I	N/I	N/I
RMSEA = 0.05 (95% IC 0.029-0.07); KMO = 0.74; Bartlett's Test of Sphericity significant (p = 0.00).	N/I	N/I
Pooled sample:CFI=0.964; TLI=0.955; RMSEA = 0.005; Cuba: CFI=0.976,	N/I	The factor correlation was r = 0.66 in the overall sample and it ranged from r = 0.55 in Peru to r = 0.77 in Cuba.
EFA: χ2 = 786.05, df = 227,RMSEA = 0.025; 90%IC = 0.023–0.027, CFI = 0.991. CFA: CFI	N/I	N/I

N/I	N/I	N/I
N/I	N/I	multiple-group CFA
RMSR = 0.079, mean fitted residual -0.05	N/I	known-groups validity analyses - gradual influence of severity category and disease duration on SCOPA-PS scores (P < 0.0001). SEMvalue 8.24 (7 - 12 in previous
RMSR = 0.08, mean fitted residual (−0.05)	N/I	Known-group validity analyses gradual and significant increase in mPPRS with HY stages (P < 0.001). SEM for the mPPRS was 1.06 (range across countries:
RMSEA0.12-0.20 (latin america) TLI 0.66-0.84, AIC 995.7 (Urban mexico) 4263.3 (cuba).	N/I	For all sites factor loadings for the one factor solution all exceeded 0.4 therefire a meaningful unidimensional scale could be constructed by summing items.
N/I	N/I	Latin America. Adjusted means CSID Cogscore 30.0, verbal fluency 16.0, word list memory 13.5, word list recall 4.3
N/I	N/I	Translation forward and backwards and face validity process described. Local training also described. They present selection of the cognitive test
N/I	Moderate reliability indices. Certain items (e.g., item 9) showed	Some items have psychometric and cultural difficulties, suggesting the possibility of multiple dimensions in the scale.
N/I	N/I	N/I
N/I	N/I	N/I

N/I	N/I	N/I
N/I	N/I	N/I
N/I	N/I	Translation and adaptation
CFA = X2 = 12.47, gl = 9, p = 1.88	N/I	N/I
N/I	N/I	N/I
N/I	N/I	Time to complete instrument: 40 minutes
N/I	N/I	N/I
(X2 [41, N = 255] = 77.30, p = 0,001, GFI = 0,96, AGFI = 0,935, and RMSEA = 0,059),	N/I	mean validity 4,29 (content validity, experets criteria)
N/I	N/I	The best Spearman correlation coeffiICents were found for the AD group, followed for the FTD and DM groups, and the worst were for VD. In the
N/I	N/I	N/I

N/I	N/I	Normative Data
N/I	N/I	N/I
N/I	N/I	Factorial model of all possible answer combinations, screen was able to discriminate in all dimensions, mobility, self care, usual activities, pain/discomfort,
N/I	N/I	CES-DR classified 82% with significant depressive symptoms; low probability (49.2%) classifying participants without depressive symptoms. GDS low sensitivity
N/I	N/I	Adaptation
N/I	N/I	Translation and adaptation process is described. Controls and patients did not differ on age and education but MMSE, GDS, Charlson index and QOL-AD were
N/I	N/I	differing scores by age, education and religious group presented
N/I	N/I	They compare results with the original CES-D administered at the start of the interview. Fourteen individuals who were not classified as possible cases in the original
Chi-Square/gl: Original: $X^2(90)$ = 1285.04, Norm X^2 = 14.27 Modified: $X^2(9)$ =	N/I	N/I
N/I	N/I	N/I

GFI = 0.966, RMSEA = 0.042, CFI = 0.987	N/I	Factor invariance was assessed between two subsamples, showing equivalence in the basic models, metric and strong invariance.
N/I	N/I	Linear regression model: The model adjusted for age and education shows how the 5WT discriminates between mild and major dementia, Mild dementia: β
N/I	N/I	N/I
CFI: 0.966<br>TLI: 0,955<br>RMSEA: 0,059 (IC 95% [0,051-0,068]).	N/I	A discriminant validity analysis was performed (inter-factor correlation r = 0.319) and it was verified that CR and AVE were adequate, suggesting that the
N/I	N/I	N/I
N/I	N/I	N/I
AAE-4Fm Model: GFI: 0.966; RMSEA: 0.051, CFI: 0.976	N/I	Invariance analysis between women and men shows that the basic measurement models are equivalent.
Men RMSEA = 0.04; CFI= 0.92;<br>Woman RMSEA = 0.03; CFI= 0.95	N/I	N/I
N/I	N/I	Agreement, as per κ statistic, between the MMSE and LCT was 60.4%, between MMSE and MoCA was 63.3%, and between MoCA and LCT was 28.1%.
X2 = 7,72, df = 5, p = 0,17, 2/df = 1,54, GFI =0,988, CFI = 0,996, NFI = 0,988; RMSEA = 0,048 [IC90%	N/I	N/I

S-Bχ2 = 54.02, df = 345 p = 0.001; S-Bχ2/df = 1.54; CFI = 0.97; RMSEA = 0.052 [IC90% 0.021, 0.078];	N/I	N/I
χ2 = 10.960, df = 5, p = .05, χ2/df = 2.192, gfi = .983, cfi = .994, nfi = .988; RMSEA = .071 [IC 90% .000,	N/I	N/I
X2(2) = 4.514, p = 0.10, CFI = 0.995, RMSEA = 0.073, IC 90% 0.000-0.165, SRMR = 0.016	N/I	N/I
N/I	N/I	N/I
N/I	N/I	N/I
N/I	N/I	N/I
N/I	N/I	N/I
N/I	N/I	Total MMSE score correlated with orientation (r = 0.736, p < 0.001), atention (r = 0.700, p < 0.001) lenguage (r = 0.580, p < 0.001)
N/I	N/I	N/I
Peru: χ25= 51.20, p < 0.001, CFI= 0.990, TLI= 0.980, RMSEA = 0.166 [0.126 – 0.208])	N/I	one-dimensional structure

N/I	N/I	Positive predictive value 92.9% and negative predictive value 85.3%. AD moderate and severe included. NB case control design susceptible to spectrum bias
N/I	N/I	N/I
N/I	N/I	N/I
N/I	N/I	The discrimination analyses between neurodegeneration and the ATN criteria showed an AUC of 0.87 for M@T, with an optimal cutoff point of 39.2, indicating its
N/I	N/I	N/I
N/I	N/I	N/I
N/I	N/I	Behavioral deterioration evaluated through FBI, IRI-EC, IRI-PT; high sensitivity.
CMIN/DF = 2.825; CFI = 0.990; GFI = 0.986; NFI = 0.985; RMSEA = 0.065	N/I	N/I
χ^2 = 13.71; gl = 5; p = 0.018 RMSEA = 0.080 [IC90% 0.031–0.132] SRMR = 0.026; CFI =	N/I	N/I
N/I	N/I	N/I

χ^2 = 19.08; df = 9, χ^2/df = 2.12 CFI = 0.985, TLI = 0.974, SRMR = 0.032, RMSEA = 0.064 (90%	N/I	Floor effect with more than 15% of participants scoring the minimum on an item. Multigroup invariance suggests gender-dependent measurement,
X^2/df: 0.009 NFI: 0.914 TLI: 0.92 CFI: 0.95 IFI: 0.95	N/I	Effect size (d = 2.94) indicates a significant difference between clinical and normal groups. Theta coefficient: 0.80
CFI: 0.98. RMSEA: 0.05 (90% IC [0.04–0.07]). SRMR: 0.05.	Use of Rasch Model: Unidimensional and multidimension	N/I
N/I	N/I	N/I
N/I	N/I	content validity by 5 dementia experts
Model 4: RMSEA: 0.047 (with a 90% confidence interval of [0.008–0.079]) CFI: 0.997	N/I	N/I
CFI = 0.999 RMSEA = 0.066 SRMR = 0.014	N/I	Translation
Chi-square (v^2) = 11.24 (df = 5, p = 0.05) CFI = 0.99 RMSEA = 0.06	N/I	Factorial invariance across gender. It demonstrates that the MAAS-5 scale shows strict factorial invariance between men and women.
χ^2 = 435 CFI = 0.97 TLI = 0.97 SRMR = 0.024 RMSEA = 0.06	N/I	Homogeneity of Items: Item 20 was eliminated as it did not meet homogeneity criteria. Descriptive analyses were performed, showing means
CFI: Peru 0.996, Spain 0.986 RMSEA: Peru 0.123, Spain 0.182	N/I	N/I

N/I	N/I	N/I
N/I	N/I	N/I
N/I	N/I	Overall Classification Accuracy: 21.1%, indicating a low ability to distinguish between clinical groups and a high error rate in the classification of controls.

Key findings that relate to the scoping review question/ Conclusion

The MDRS does not seem to be a useful tool to detect MCI since it generates numerous misclassified cases. The development of more accurate tools becomes fundamental in order to detect MCI.

The AD8-arg is a tool for quick and easy administration with adequate psychometric properties for use in screening of dementia in primary care in our region.

The SV-BNT showed good Construct and/or Criterion Validity bout poor concurrent validity, epeICally a very low sensitivity.

This was a case-control design so possibly vulnerable to spectrum bias. Pearson correlation 0.678 with MEC. 0.597 with ACE-R, with PFAQ -0.578. Also give PPV 45 (42-47, NPV 95 (93.6-97.7)

An easy tool that is easy to administer. Self administered and lastign 15-20 minutes. Global scorees and all subscales had strong correlation with the caegiver burden and psychiatric scales (NPI-12, zarit, HADS-A and HADS-D) no correlation with age as expected

The Argentine version of the MBT correlated significantly with the MMSE and the memory battery and is a useful tool in the detection of MCI. The operating characteristics of the MBT are well suited, surpassing other tests commonly used for detecting MCI. diagnostic accuracy was higher than that of the MMSE

ECog total score ranged from 39 to 134. Ceiline effects not observable. The ECog scale showed high accuracy and discriminative power in differentiating CN, MCI, and mild AD. More accuracte than pFAQ

Phototest correlates significantly with MMSE and CDT. The Phototest is aneffiICent instrument for the detection of mild dementia or MCI, with good accuracy and goodcorrelation with tests measuring overall cognitive impairment.

CSID seems a suitable dementia screening test for low educated population

In this paper we show some of the psychometric properties of the ACE in patients with low soICo-educational level. Also a cutoff point is provided for clinical use.The results could be used in patients in the lowestsocial strata,which is more common in the Public Health Sub sectorin our country

As a screening tool, the CSIS can be administered by trained sanitary agents and nurses, those who usually have the first contact to the patient in the public health system, offering a comprehensive outlook of several cognitive functions status. The CSIS is composed of a global score that considers the performance in various screening tests widely used for cognitive defiICts detection.

This final version was tested on 31 participants. The result of this process was the Argentinian Spanish version of the MLSE, which is intended to be equivalent to the other versions under development

The normative data obtained suggest a cut-off point of 18 for people with primary education and 22 for people with secondary or higher education. It should be noted that these are below those indicated in the pre-existing normative data. The importance of using norms adjusted to the soICo-cultural context is highlighted.

The results show that the CQC has good validity and reliability. Thus, the content validity was positive in the expert consultation and the value of its reliability is considered acceptable. Beyond this, it is important to consider the importance of conducting a normative study to establish the expected results in a healthy population.

This study is the first analysis of CRQ from IRT, concluding that the instrument is more reliable when applied to subjects with less reserve. The CRQ has acceptable convergent validity.

The MoCA-A is an accurate reliable screening test for MCI and MD in Argentina.

As a result, an adequate adjustment of the model to the empirical data was verified for both versions (penICl-paper and Internet), as well as an adequate metric invariance between them. These findings provide evidence of validity and reliability of the GDS-VE instrument, on the Argentine population.

The results of this study have implications for psychological assessment and intervention in terms of knowledge and enhancement of coping factors used by older adults.

The Cognitive Reserve Questionnaire is an instrument with acceptable psychometric properties for use in an Argentinian population. More research is required to further investigate the low factor loadings of items 5 and 8 and to strengthen the evidence of reliability.

our results show that the ACE-III may be considered a useful tool for detecting cognitive impairment in patients with ATD and bv-FTD. Thus, this brief, sensitive test may be of great value in clinical practice for the initial assessment of patients with suspected dementia and for determining the need for complementary examinations.

Study presents preliminary support for the validity of the instrument as a multidomain geriatric screening tool for fast-paced settings. In the study, the TaGA was more accurate than the ISAR, which is the most commonly used tool in that setting.

The GEAP-b scale has proven to be reliable and valid in the screening of pain-related depression in the elderly.

The Brazilian version of the DAD is an adequate and reliable tool for assessing functional ability in AD patients.

The instrument seemed to be psychometrically suitable when applied to older people. However, further cross-sectional and longitudinal studies, carried out in different contexts, may explain the effects of somatic and situational variables on the results of the instrument in older people.

Increased use of Cognitive Reappraisal is an indication of emotional health in the elderly.

The usefulness of MMSE as a screening test is confirmed. The CERAD battery seems to be a quick and valid se of test for the diagnosis of dementia, which could be further shortened by the suppression of sub-tests like memory recognition without loss of effiICency.

Had adequate measuring properties for use with elderly presenting limited communication.

The MSQ is a quick and simple test, but as far as we could determine, its use is not free from problems. Further studies with the MSQ and other screening instruments are strongly recommended to improve the epidemiological assessment of the elderly.

In conclusion, after the cross-cultural adaptation of the ANU-ADRI, the wording of the instrument was found to be easily understandable by the Brazilian population.

Comparison between the adapted MMSE and modified MMSE

The modification of copy and calculation items of ad-MMSE, are responsible by the best performance in mo-MMSE. Individuals with more than eight years of formal instruction are protected against a reduction of their capacity to solve cognitive tests. However, low instructed individuals have not this capacity and so they present signals of intellectual aging before they become elderly people.

The Brazilian version of the LCT has acceptable levels of reliability for use in low-educated elderly individuals, given that it minimizes the education bias present in most cognitive assessment scales.

The Brazilian telephone version of the Mini Mental State Examination was significantly and strongly correlated with the in-person MMSE. Both versions were considered to be interchangeable. In addition we were able to predict a future score on MMSE based on the previous score on the Braztel-MMSE and vice versa. Additionally this telephone version was shown to be

Behavioral Assessment of the Dysexecutive Syndrome was effective in detecting executive function defiICts in mild Alzheimer's disease patients, particularly the task switching, time monitoring, and rule-shift subtests.

The Brazilian version of the ACE-R proved to be an accurate and brief cognitive tool for the diagnosis of mild AD in our milieu.

The Brazilian version of the ACE-R proved to be a promising cognitive instrument for testing both in research and clinical settings.

Further studies are necessary to validate the VITOR QLSE in a Brazilian elderly population.

Our results suggest that the MLQ is a reliable measurement to evaluate presence and search for meaning in life in the Brazilian population in a wide variety of age groups.

The CCQ8 can be used to accurately differentiate between normal subjects and individuals with cognitive impairment, constituting a brief and appropriate instrument for cognitive screening.

The Vulnerability to Abuse Screening Scale proved to be a valid instrument with good psychometric capacity for screening domestic abuse against older adults in Brazil.

The data presented here suggest that the RUDAS-BR is a accurate as the MMSE for classifying AD.

Translation of the RSQ is the first step towards the validation of an attachment evaluation instrument for use in the elderly population in Brazil, allowing for future studies on this topic.

This Portuguese version of the CDR may be appropriately applied for research in this setting.

DRS is a useful instrument for cognitive assessment in LTICs. In this study, it was more sensitive than MMSE.

Poor adjustement of the American factor structure to the Brazilian sample. The authors suggested to perform an exploratory analysis.

A score corresponding to 12 points in the 90 percentile on a scale from zero to 40 was observed, which suggests severe disability. The score in WHODAS 2.0-BO increased with the advance in age, as well as in the presence of comorbidities, negative health perception, depression, high blood pressure, visual and hearing impairment and mobility impairment.

The test had good sensitivity to discriminate MCI from unimpaired controls in the sub-sample of individuals with more than 8 years of schooling. Our findings suggest that the SKT is a good screening test for cognitive impairment and dementia. However, test results must be interpreted

The Brazilian version of the SKT maintains its original psychometric properties and displays significant correlation with previously validated screening tools for dementia.

The present study introduces a methodology for the development of health instruments that proposes the association of a quality approach (item generating) and a quantitative approach (objective measurement of the instrument’s performance).

This research provides evidence for the usefulness of the 10-CS as a screening tool to predict delirium in older adults with hip fracture

The CDT scored by the Shulman (2000) method appears to have good to excellent reliability in an elderly population with very low formal educational level. However, difficulties in distinguishing between scores 4 and 5, and a low proportion of score 1 tests suggest these scores may not be totally adequate for this population. Further studies are necessary to determine the consistency of our results in

The results obtained already provide validity evidence of the internal structure of COPPES-BR,

The results indicate that the MAST is a good screening test for the detection of alcohol abuse and dependence in an elderly male population and that the MAST should be used in combination with a questionnaire assessing the frequency/quantity of alcohol consumption to optimize the detection of cases and characterize their current drinking patterns

Dementia Rating Scale showed a high sensitivity to detect cognitive impairment in this population,

For low schooling elderly, the combination of the FAQ and CVF represented a very simple method of increasing the chances of correct screening. For those with higher schooling, the combination of the FAQ and CDT was more suitable.

Two simple and easy-to-apply instruments showed high sensitivity and reasonable specificity, and are probably useful for the screening of cognitive impairment in the elderly in outpatient services

The Brazilian version of the MAST-G presented internal consistency values similar to the original English version, showing it to be adequate for use in the national context.

The adapted BNT appears to be the most suitable for use in the low-educated Brazilian population. The present study provided normative data for low-educated elderly on several different versions of the BNT, which may be helpful in diagnosing naming defiICts among elderly in these strata of the population.

The EBADEP-ID showed validity evidence to be used with the elderly people.

The Brazilian version shows satisfactory equivalence to the original and good reliability levels.

In this small exploratory study the CASP-19 Brazil demonstrated good psychometric properties.

The PACSLAC-P is a simple and adapted for use in Brazil. This makes it easier to assess pain in older people with severe dementia.

It seems that it is not adequate for dementia screening in individuals with less than 5 years of formal education; in addition, the four scoring methods tested had similar accuracy to screen older outpatients for dementia.

The IQCODE-BR is an instrument with good accuracy for the detection of dementia syndrome in Brazilian older person.

While screening elderly outpatients for dementia, schooling must be considered in the choice of the best cutoff point in the Mini-Mental State Examination.

The Perceived Stress Scale proved to be a clear and reliable tool to measure the perceived stress of Brazilian elderly, showing suitable psychometric performance.

The use of the cut-off points described above can serve as a guideline for the clinical investigation of elderly people with suspected dementia in our environment.

These results indicate that the short GDS versions with 1 and 4 items are unreliable for use in clinical practice. In contrast, the GDS with 10 and 15 items produced consistent results in the assessment of elderly patients when total scores were used as

GDS-15, GDS-10 and GDS-4 are good screening instruments for major depression as de®ned by both the ICD-10 and DSM-IV. The shorter four- and one-item versions are of limited clinical value due to low reliability and failure to monitor the severity of the depressive episode. General practitioners may bene®t from the systematic use of short GDS versions to increase detection rates of depression among the elderly.

The factorial structure analysis of the GDS items revealed that it is not appropriate to generalize the results of this structure for the population in general, so that the results found suggest further researches, particularly with regard to factor analysis of the Scale.

The instrument developed in this study has shown good properties and can be used as a valid estimate of
premorbid cognitive abilities in low-educated populations. The applicability of the PCAS, both as an estimate of premorbid
intelligence and cognitive reserve, is discussed.

normative data for the MoCA and the MoCA-MIS that will
faICIitate the use of the test in Brazil and, potentially, in other populations with substantial proportions of low-educated individuals.

The CAMCOG can be used as a cognitive test for patients with low educational level with good accuracy. Patients with higher education showed lower scores than previously reported.

The CDT is a robust screening test when compared with the MMSE or the CAMCOG, independent of the scale used for its interpretation. The combination with the MMSE improves its performance significantly, becoming equivalent to the CAMCOG.

The translated and validated Clock Test into a community sample of aged showed to be a brief screening instrument, with good Construct and/or Criterion Validity when compared to other studies.

The web versions of the WHOQOL-bref and WHOQOL-old questionnaires presented good evidence of accordance and reproduICbility.

The results suggested that education influences IGT performance, with worse scores among the illiterate. Results may be used by cliniICans to interpret IGT performance among seniors with low literacy levels.

The GDS-30, GDS-15, GDS-10 and GDS-4 proved to be good screening instruments for depression in primary care clinics in Brazil, whereas the GDS-1 failed to perform adequately

The SF-36 and WHOQOL-BREF are reliable instruments for clinical and research uses in Brazilian older women.

Norms for the mini-mental state examination - Adjustment of the cut-off point in population-based studies (evidences from the Bambui health aging study)

This study provides support for the cross-sectional equivalence of the MMSE, suggesting that most of the items and underlying constructs remain meaningful after alteration and translation in a low-education sample with lower overall distribution of scores.

In the absence of comparable cut-off points, percentile distributions are more adequate for population-based studies of elderly with low schooling level.

It can be concluded the PDT has tha confluence of information (cognitive and motor) a more robust con fluence than other simplified instruments such as the MMSE.

The CDT did not show a strong correlation with MMSE and CAMCOG, both important instruments in Brazilian population to investigate dementia. For elderly individuals with high education levels, the CDT did not seem to be a good test to detect cognitive impairment.

Not all MoCA subtests might be fundamental to clinical diagnosis of MCI. The reduced versions of MoCA did not add diagnostic accuracy.

The MoCA test showed the greatest predictive value to differentiate AD from MCI and also differ MCI from normal controls. Furthermore, MoCA was significantly correlated with the age variable and MMSE, CAMCOG, CDT, Verbal Fluency and PFAQ tests, instruments that are already validated and widely used in Brazil.

The 15-item GDS proved not to fit the Rasch Model in its original format and, thus, unsuitable for total score calculations in a Brazilian sample. Item deletion led to a 10-item solution, which has a strong scale structure and is suitable for all sorts of parametric statistics with no loss of performance.

Unidimensionality and local independence were seen in all domains. Changes in the response scale and deletion of problematic items improved the scale’s performance.

The WHOQOL-BREF instrument shows suitable psychometric performance in a sample of Brazilian older adults, becoming a useful alternative in the measurement of quality of life in this population.Results are not clear and didn´t support the general conclusion of the text.

Moreover, the Brazilian version of the COPM proved valid for examining ADL and a reliable instrument for identifying and measuring ADL in MCI patients.

ADCS-ADL scale presents satisfactory psychometric properties to discriminate between MCI, AD and normal cognition.

The Brazilian version of the AD8 is a valid, reliable, quick, and easy screening instrument for dementia.

The global score agreement of the CDR scale with the gold standard was good, and with the MMSE was moderate. We also observed face validity for dementia severity. No impact of education was observed upon CDR global scores.

The clock and the animal tests showed similar specificity, but higher sensitivity than the RBMT subtests.

GAI-BR has demonstrated very good psychometric properties and can be a reliable instrument to measure anxiety in Brazilian elderly people.

The present results indicate that the MoCA-BR maintains its core diagnostic properties rendering it a valid and reliable tool for the screening of MCI among older individuals with at least 4 years of education.

The CANS-MCI-BR maintains adequate psychometric characteristics that render it suitable to identify elderly adults with probable cognitive impairment to whom a more extensive evaluation by formal neuropsychological tests may be required.

The M-ACE is a brief screening test which provided high accuracy for diagnosing AD in this sample. The suggested cut-off point in this study was 20 points for AD.

The Portuguese version of the Clinical Dementia Rating is a valid instrument for classifying the dementia status of the elderly. Almost half the cases considered normal by the diagnostic criteria of the Mini Mental State Examination were questionable cases according to the Clinical Dementia Rating and might correspond to cases of mild cognitive impairment, with an increased risk of conversion to dementia cases.

Both scales showed moderate agreement and were useful for detecting a relevant prevalence of the target outcome of depression among the elderly.

Neither screen was suffiICently predictive of SCAN ICD-10 diagnosis to recommend their use in two-phase surveys theoretical advantages, the GDS-30 performed no better than the GHQ-12, and was biased in similar ways.

The results reveal high reliability (α = 0.89) and high discriminatory power for identification of cognitive disorders associated with Alzheimer's disease DA (ASC = 0.98; sensitivity/specificity = 94.9/92.3%). The results suggest psychometric psychometric adequacy of the instrument.

Tested 3 versions PQOL, C-PQOL, CQOL . All had good reliability and all correlated with behavioural distUrbance, depression, IADL and dementia severity (convergent validity)

The tool was useful in cognitive screening for those with mid education, but accuracy was limited in those who were illiterate. Cut points of the tool also differed. NB screening tool completed blind to diagnosis and vice versa. Type of sampling not stated but assumed to be clinic attenders (even convenience) therefore spectrum bias less likely

Good reliability across, gender groups, age groups, education and dementia status. Rest reteest reliability slightly worse for those without education 0.87 ICC but still good. The high score of Cronbach' alpha found in this study for the instrument' set of items suggests that the Br-CAMCOG-R evaluates one same construct

In the sample 16 cases major depression (5.3%) 35 of dysthymia (11.6%).

participants were selected to be representative of older persons with varying dependence and disability. Resulted in an instrument with eight dimensions and 43 items. The Clinical Impact Method proved to be appropriate for the construction process.

NB diagnostic accuracy was completed with MCI and dementia cases only, no controls. Findings should be interpreted as such. Screening accuracy (MCI vs dementia) for subscales and total GADL was significant for the older cohort (74+) but not younger participants

RAVLT showed a high internal consistency, weak correlations with the MMSE and CDT, and a bifactorial structure, which is related to the processes of learning and episodic memory retrieval. Only age and gender affected test performance.

Preliminary findings suggest that the short IQCODE can be used for the screening of MCI and dementia in Brazil.

See notes (next box) Both short and long versions of the IQ code had good diagnostic accuracy for dementia that surpassed that of the MMSE. The instrument was not significantly assoICated with education orsocial class

GDS-15 presented good reliability and validity (concurrent and of criterion). In cardiologic settings, its use, which is simple and fast, can be utilized in the screening for depression

Cognitive performance on MOCA-BR by healthy elderly was strongly influenced by schooling and, to a lower degree, by age. The average MoCA scores in this study were lower than those of the study that validated the MoCA in Brazil and those of the initial study of the MoCA. The average score of the MoCA-BR of the participants in this study was 23.2 ± 2.7, ranging from 17 to 29.

Mini-cog is not a good screening measure in low-literate elders (below 5 years education). They did not really actually do the mini cog they extracted the questions from other measures.

Test-retest reliability was high for total COGTEL score and acceptable-to-high for the remaining six subtests of the instrument. Similar test-results were found for MMSE score and level of schooling.

Best fitting cut-points, the MMSE estimate of the prevalence of dementia wasfour times higher than determined by the DSM-IV criteria. Education, age, sex and income influencedMMSE scores, independently of dementia caseness.

The reliability levels lead to the conclusion that the IQCODE-BR version is easy to comprehend. Satisfactory equivalence to the original version was observed.

There was no statistically significant association between the IQCODE-BR scores, and elderly participants' age andeducational level. In contrast, the MMSE scores werenegatively correlated with age ($r = –0.202$, $P < 0.01$) andpositively correlated with years of education ($r = 0.430$, $P < 0.01$).

Logistic regression analyses showed that women, older participants and participants with lower education or income levels were more likely to be misclassified as positive by the SRQ-20. After adjustment for gender, age group and income, the association between education and SRQ-20 misclassification was not significant ($p = 0.53$).

Total mean score for CG and AD was 10.9 and 22.9 for level I, 7.8 and 22.4 for level II, and 6.2 and 15.4 for level III, respectively. These results indicate that our version of the ADAS-Cog is useful to identify mild dementia, though there may be an overlapping when comparing high education demented with low education non-demented subjects.

Diagnostic interpretation of the ROC curve revealed that cut-off < 60 for overall quality of life obtained excellent sensitivity and negative predictive value for tracking older adults with probable worse quality of life and dissatisfied with health.

VES-13 score ≥ 2 and WHOQOL-bref score < 60 adequately detected poor QoL in patients treated in primary health care

GAI and GAI-SF can be easily applied in primary care, including in the low-educated population. The GAI showed high internal consistency (0.89), proving comparable to the original validation study and the Brazilian version of the GAI but the short form had lower internal consistency and test retest reliability

STADP is a valid test for screening Alzheimer's disease. Different cut offs are given for higher and lower education

The total CAMCOG score was more accurate than its subtests Mini-mental StateExamination, Verbal Fluency Test and Clock Drawing Test when used separately.ConclusionsThe CAMCOG discriminated controls and MCI from demented patients, but was less accurate todiscriminate MCI from controls. The best cut-off value to differentiate controls and demented was higher than suggestedin the original

verage of 43 ± 9.4 minutes, and an average total score of 67 ± 14.8 points. The Br-CAMCOG-R can be a useful tool for the cognitive evaluation of elders that tested positive during initial screening.

To the best of our knowledge, this is the first study on the MADRS and CSDD cut-offs for depression in elderly with and without dementia in Latin America.

Scale had moderate consistency. This is the first adaptation to Brazilian portuguese of the scale. Purpose was higher in lower age (80-84) and higher income.

Education and Age did not significantly influence the final EAMI score ($F = 3.47$, $p = 0.08$ and $F = 0.7$, $p = 0.4$, respectively). EAMI total score, personal semantics and autobiographical events elements all sig differentiated cases and controls as did autonoetic awareness assessment

logistic regression analyses showed thatwomen, older participants, and participants with lower educational or income levels were more likely tobe misclassified as positives by the SRQ-20. After theadjustment by gender, age group, and incom

Adaptations were necessary to suit the local health system, and these together with the need to explain carefully the concept of need to uneducated participants meant that the duration of the assessment was around 40 minutes. More practically orientated domains such as "looking after home", "food", "self-care", and "benefits" were better understood, and more easily rated. Domains such as "psychological distress", "behavior" and

The results of this study suggest, therefore, that the Portuguese version of the ENEDAM can be considered a reliable tool to be applied to Brazilian patients with a presumed diagnosis of dementia.

The Brazilian version of the RBMT seems to be an appropriate instrument to identify memory decline in Brazilian older adults.

Strong empirical support was found for the proposed structure of four of seven positive and all four negative sub-dimensions. More work needs to be done to assess and, ideally, further refine the positive sub-dimensions of this Brazilian behavioral scale.

The four-factor structure of the MOS-SSS was found to be suitable and presented adequate Construct and/or Criterion Validity for the assessment ofsocial support in elderly users of primary healthcare faIClities.

The questionnaire developed is a valid, statistically appropriate and clinically effective self-administered instrument for individuals with PD.

LHFQ exhibited satisfactory psychometric properties, mainly for its physical domain for which discriminative, convergent and divergent validities were confirmed. The emotional domain was less able to discriminate between NYHA classes, and the convergent and divergent validity was partially supported.

The AMPI-AB is a valid and reliable tool for managing older adults in resource-limited primary care settings.

In spite of some remaining dimensionality issues needing refinement and the relatively restricted correlations with expected variables, the CASE may be reaffirmed as a promising detection tool for risk of abuse in clinical practice and applied research.

A one-factor dimensional structure and a reduced version with 20 locally independent items were the most tenable solution. However, although promising, this simpler structure requires further examination before it may be fully supported and recommended.

Educational level positively correlatedwith test performance on the BNIS-PT and was repeatedly observed to overshadow the effects ofage, suggesting its important role in the development of higher cerebral functions in multipledomains in a Brazilian sample of normally functioning individual. correlation with education, when age was held constant, was $r = 0.71$, $p < 0.001$

care needs were classified into the following scale - no need (requires no caregiver), minimum need (requires caregiver sporadically), moderate need (requires caregiver intermittently) and maximum need (requires full-time caregiver)

A unidimensional model was the best solution compared to a bifactor one and had high internal consistency. Post hoc diagnostic accuracy appears high.

The results suggest that the Bayer - Activities of Daily Living scale applied to aninformant can help in the diagnosis and follow-up of Brazilian patients with mild to moderate dementia

The MoCA' subtest (cube draw) should receive more attention and be corrected considering types of errors. MoCA-VC proved to be more effiICent than MoCA original in differentiating healthy elderly from cognitive impairment, in patients with more than five years of study.

The prevalence of depressive symptoms was high in this sample and the Visual Analogue Scale of Happiness and Cornell Scale for Depression in Dementia should not be used as similar alternatives for evaluating the presence of depressive symptoms, at least in populations with low educational level

The MoCA test did not have a high accuracy for detecting ICND in the population with a low educational level. Nevertheless, this tool may be used to detect dementia, espeICally in individuals with more than five years of education, if a lower cutoff score is adopted

The SF-36 demonstrated to be more adequate regarding the ceiling and floor effects, whereas the NHP presented a higher internal consistency and reliability levels. These findings should be considered for selecting instruments for the assessment of HRQOL of community-dwelling elderly

The results of the study show that the Brazilian version of MoCA is a reliable cognitive tracking tool and is accurate for the detection of MCI and early stage AD

An adequate reliability and applicability was observed in our sample after adjustments and adaptations of the scale for use in elderly

Similar to ‘growth charts’, we propose a method which factors in age and education to help determine whether elderly individuals show abnormal performance on serial MMSE. A significant decline on cognitive charts should prompt further investigation while a non-significant decline reliably identifies those individuals who do not need further cognitive work up

Our study presented valid psychometric data indicating that the CAMCOG-BILL has adequate sensitivity and specificity to assess illiterate elderly with possible AD. Our instrument also presented convergent validity compared to the MMSE (a similar cognitive instrument), with the tools presenting significant and robust correlations.

Our study assessed ACE-R performance in dementia, MCI and cognitively healthy adults

We demonstrated that a 4-dimensional model for the LIFE-H 3.1-Brasil was valid and reliable to measure the quality ofsocial participation of independents older adults living in the community.

All scales used (FTD-FRS, CDR-FTLD, CDR, ACE-R, and MMSE) documented a significant decline in scores after 12 months.
It was observed that patients with bvFTD and PPA experienced a greater decline compared to those with AD, except for the CDR-FTLD.

the UCLA Loneliness Scale version 3, presents evidence of satisfactory validity and reliability, and can be used to assess loneliness among aged Brazilians.

Finally, the report on the process of adapting the pTRSLG-EI provides some possibilities and alternatives for future studies on adapting instruments with pictorial stimuli. SpeICfically with regard to the pTRSLG-EI, the wider use of the version developed in this study requires the accumulation of more validity evidence. However, to date, the adaptation of the pTRSLG-EI has provided a tool that is suitable for the Brazilian context

The normative data presented here may be useful for samples with similar soICodemographic characteristics to those found in this stud

Bender was a test that pointed out satisfactory psychometric data to be applied in the elderly with dementia

The K10 scale proved to be valid and reliable for verifying mental distress in elderly people in PHC.

The supermarket category of semantic verbal fluency provides better accuracy than the animal category for the identification of dementia in a Brazilian elderly population with low educational level.

The MFIS-PD/BR is valid and reproduICble to use in assessing the fatigue symptom in Brazilian PD subjects.

CFI proved to be an instrument with good accuracy and easy applicability to identify older adults with SCD.

Our completed “Older Adult Lifestyle Scale” (OALS) is composed of 19 items divided into four subscales. The OALS has shown good psychometric qualities for Brazilian older adults over 60 years of age, and we can now recommend its use in this population.

EMRII-BR is a valid and reliable instrument for measuring resilience in Brazilian older adults.

only the Conflict Tactics Scale turned out to be reliable and stable for determining physical and psychological violence among the elderly, thus contributing as a way of uncovering the phenomenon.

The PIMD-p showed satisfactory psychometric properties for measuring intensity of pain in demented older adults with impaired verbal communication.

The Brazilian ACE-R achieved a good diagnostic accuracy for differentiating AD from bvFTD patients and for
differentiating AD and bvFTD from the controls in the present sample.

DEX presented a satisfactory factorial structure for older adults, which can be considered a reliable self-report measure for complaints of executive functions.

The proposed structural model has shown Construct and/or Criterion Validity and an appropriate internal consistency, explaining 50% of data variability. Its application can promote the understanding ofsocial reality and encourage a positive attitude and elderly engagement in leisure activities.

the scale was robust for screening mental distress, given its high reliability, in terms of homogeneity, and adequate criterion validity, whose best cut-off point for screening was a score greater than 14.

The PSI-16 scores for prefrontal symptoms were higher in the clinical than in the non-clinical group. In general, the PSI-16 is a valid and reliable tool for clinical assessment of elderly individuals with and without neurodegenerative pathology.

Among other implications, this study suggests that the MMSE and its cut off points should be used carefully. For prudence, this measure needs to be administered in conjunction with other instruments that evaluate the same construct or at least related constructs, such the Clock Drawing Test, even if the goal is only to screen cognitive status.

This simple predictive model highlights functional status and a proxy for dehydration as a useful tool for identifying older patients that may benefit from close monitoring and preventive care for early diagnosis of delirium.

Limitation = non randomised sampling methods; Adaptation: pilot test based on the Spanish version.

Only normative data

Reliable instrument for chilean elderly population.

Confirmatory factor analysis shows that the two-factor solution fits the data from the Chilean sample. In this way, this brief index has adequate psychometric properties

The three factors explained together 82% of the variance of the construct. This questionnaire constitutes a tool for gerontologicalsocial intervention in order to prevent the isolation or lack ofsocial integration of the elderly

The 5-item GDS seems to be a promising screening tool for depression. If revalidated against clinical evaluation, it might be the preferred screening tool for depression in the Chilean
community-dwelling elderly.

The adapted version of the Eurotest it is a valid and reliable screening instrument for the diagnosis of dementia in Chile.

MMSE-EFAM has a low sensitivity to detect patients with Dementia and it is not an effective screening tool.

We have demonstrated the reliability and validity of SF-36 questionnaire to evaluate health related quality of life, reporting Chilean-SpeICfic factor score coeffiICents for MCS and PCS based in national Chilean means and standard deviations for older people.

The Geriatric Anxiety Inventory has very good psychometric properties measuring anxiety in elderly people, being an adequate instrument for the screening of anxiety on this population.

The MUNSH proved to be a very reliable instrument (Cronbach's = 0.90) as a measure of happiness in the population of older adults (65 years and older) of the two largest older adults (65 years and over) in the two largest cities of the cities of the Maule region, Talca and Curicó, in Chile.

Gives cut point also for MCI. Relates performance to dementia sevrity on CDR. However, not a blinded assessment and a convenience sample was used. Use of MCI patients reduces spectrum bias. Also gives AUC and other measures alongside correlation for MOCA, MMSE and ACE-R.

The ACE-R-Ch showed acceptable psychometric properties, becoming a valid and reliable instrument to assess global cognitive effiICency or cognitive impairment. Its diagnostic utility to detect dementia patients also worked very well in a Chilean elderly sample.

This scale is applicable in elderly population treated at the Primary Health Care in Chile.

This screening test, us-ing MMSE and PFAQ, has a good sensitivity and specificity for the diagnosis of dementia inChile. Being simple and of low cost, it can be applied in primary health care

The authors propose a unifactorial solution of 6 questions which they present in the addexe being the only one with acceptable psychometric properties. Goodness of fit results are given as follows agFi= adjusted goodness of Fit index; rMrs= standardized root Mean square residual; RMSEA = root Mean square error of approximation. best models are 6 factors or 1 factor model C - items 04, 06, 17, 19, 20, 24 of the original scale

PSSQ is an acceptable, reliable, and valid instrument for assessing perceivedsocial support among Chilean older adults, including ethnic minorities.

This study shows that FAB is a useful tool to discriminate between healthy people and people with dementia.

Overall diagnostic accuracy can be considered as outstanding (AUC ≥ 0.904) when discriminating HE from both mild NCD and major NCD. These results showed that the MoCA is a suitable tool to identify mild NCD and major NCD.

The BRCS is a feasible, acceptable, reliable, unidimensional and valid questionnaire, with good measurement properties, for assessing resilience among older Chilean adults.

The general scale and the subscales of spiritual beliefs and practices showed adequate reliability.

It can be concluded that the EAPAR is valid for use in Chile. It shows adequate internal consistency and validity. The results obtained are more robust than those obtained in the Canadian population and similar to those in the French population. Therefore, it is stated that the EAPAR corresponds to the reality of the phenomenon being studied

MoCA and the ACE-R scales appear as better instruments to detect dementia in older people.

PT proved to be more accurate in identifying MCI in elderly people in rural Chile using TNP compared with the gold-standard measure. Its use is recommended in primary health care contexts to detect preclinical cognitive alterations, such as MCI.

The PHQ-9 has adequate psychometric properties for elderly primary care users. In clinical settings, it showed its greatest utility in ruling out the presence of an MDD, however, its clinical value for identifying possible cases of MDD is limited. In cases above the cut-off point, it is recommended to perform a more thorough evaluation.

This study showed that RUDAS is a recommended instrument for evaluating older people with low educational levels. However, more studies are needed to prove the validity of the RUDAS on Chilean older people.

The observed area under the curve (AUC) was higher than .90 in all the FCSRT measures in the major cognitive disorders and healthy older people. According to the AUCs, it was shown that Free Recall, Sensitivity to Cueing Index, and Delay Recall of the FCSRT are suitable to detect major neurocognitive disorders.

The results support the existence of three factors for the Multidimensional Scale of Perceivedsocial Support (MSPSS), differentiating the support perceived from Family, Friends, and significant others. All factors present good or excellent reliability. This solution is theoretically consistent and coherent with the literature, and it presents evidence in favor of the use of MSPSS as a measurement to distinguish the support

The validity of the SF-12 questionnaire for the assessment of HRQoL among older people with depression or dependence was demonstrated.

The results indicate that DJGLS-6 is a reliable and adequate scale for non-indigenous Chilean older people. However, it should be culturally adapted to indigenous ethnic minorities.

The results showed that, globally, the Addenbrooke's Cognitive Examination III possesses adequate psychometrics properties. Furthermore, the information function test shows that the subscales have different sensitivity to different levels of impairment. These results can contribute to determining patterns of cognitive deterioration for the adequate detection of different levels of dementia. An optimized version is suggested
These values warrant recommending the use of the SLO, since it offers a view of losses in old age that is both broad and detailed, thus faIClitating the production of cumulative and comparable knowledge in the field of psychogerontology while also making it possible to establish interdisICplinary connections.

The GDS-15 showed satisfactory psychometric characteristics for the samples studied. However, the better results observed for the non-indigenous group suggest that some characteristics and content of the rating scale are not fully appropriate for the indigenous older population.

ACE-III is a valid, reliable, and useful measure for the clinical detection of dementia. The combined use of Orientation and Memory subscales is proposed as an alternative and time-saving ACE-III indicator.

The version obtained in Spanish of the Quality of Life in Alzheimer's Disease scale is valid from the point of view of its content and equivalent to its original version.

the SWLS would not be a valid instrument for cross-cultural comparisons of the levels of life satisfaction across older adults from Chile and Ecuador. These results emphasize the importance of estab-lishing measurement invariance of the scale before comparing the SWLS scores across different cultures orcountries.

Construct and/or Criterion Validity is mentioned, but there were no t done any psychometric analysis to support it. Only revalence information and reliability without mentioned the coefficnet used to this test.

Both scales are similarly effiICent to detect depression in the sample. The BAS-DEP showed an appropriate level of reliability, but low reliability of the EBAS-DEP.

The GDS-15 showed acceptable internal consistency and construct reliability. However, its two-dimensional factor structure is not satisfactory. It is possible that the GDS15 exhibits low ability to identify depressive episodes in elders with low scholarship.

Based on the results of the psychometric properties and characteristics of applicability of Pesotest, their use is recommended in primary care as a screening tool with predictive utility in the diagnosis of dementias.

ESTE scale validation indicates that it can be used as an adult loneliness measurement instrument in Colombia’s adult population.

The MoCA-S is a valid screening tool and is useful for identifying MCI and MD in Colombia.

The results of MOCA-S are compared with the Leganes Cognitive Test to show simililarities, but there are not statistical analysis to support it. Only comparison of results.

Both version have aceptable reliability. Further research is necessary.

The ESTE-R sacle is a useful tool to measure loneliness in a sample of older people in the city of Bucaramanga.

ASEQ is a two-dimensional and reliable scale in older adults attending a Life Center in Cartagena, Colombia. New studies are required to evaluate the performance in a representative sample.

Local dependence was identified between the attention and calculation items. These produce a second measure that affects the unidimensionality of the instrument. They suggest that attention and calculation should be considered separately/differentiated from the rest of the measure. And that the MMSE has limitations in terms of reliability and generalisability in this context.
A unidimensional, interval-scale measure of quality of life can be obtained from the WHOQOL-BREF for older adults. All items and a high proportion of older adults showed adequate adjustment to the Rasch model. Differential item functioning was identified in five items, although their impact on overall measure was low. category structure had to be collapsed from 5 to 4 choices

The LOT-R in its Spanish version is an appropriate and practical tool for screening purposes in individual diagnostics and epidemiological research in Latin American samples

The FAMOASQ, adapted and validated in Colombia, meets the required psychometric properties

The Colombian version of the ACE-R demonstrates to be a valid and reliable global cognitive screening tool. It is effective at discerning MCI individuals from healthy within a group of participants with a low education level.

The AQ-D is valid and reliable for screening anosognosia in Colombian patients with MND.

All DDT-Pro items, which represent the three delirium core domains, are important for SSD diagnosis.

PD-CRS has acceptable psychometric properties for the Colombian population and has significant correlation and agreement with a validated scale (MoCA).

This scale helps explore frequent
forgetfulness in daily life in older adults in the general population.

The DDT-Pro had high validity and reliability in provisional delirium diagnosis by physiICans and nonexpert cliniICans, although further validation is warranted before widespread use can be recommended.

The original empirical model of the SF-36 with eight first-order factors is supported in our sample of Costa Rican older adults.

The scale generated is reliable and valid to assess resilience in an elderly people with similar characteristics to the present study.

The Cuban version of ACE-R is an accurate neuropsychological battery which constitutes a valid screening instrument useful for discriminating cognitively healthy subjects from patients with MCI.

The present findings showed that, in comparison with FAB, the IFS presented higher sensitivity for the detection of MCI patients (md-aMCI subtype) with executive dysfunctions. We recommend the inclusion of such test in screening protocols for dementia for the early detection of executive dysfunctions in MCI patients, in all levels of the Cuban Public Health System

The BHA has excellent performance characteristics in detecting cognitive impairment including dementia and MCI in a Hispanic population in Cuba and outperformed the MoCA. These results support potential application of digital cognitive assessment for older adults in LMCI.

The PDQ-EV has satisfactory internalconsistency, and converging and discriminating validity.

The study validates the Spanish version of the Geriatric Depression Scale (GDS-15) for use in Ecuadorian subjects, emphasizing the need for locally validated instruments for diagnosing depression in older adults.

The current study adds evidence for the Construct and/or Criterion Validity of Euro-D and for the possible differential association of depression symptom-clusters with gender and verbal fluency in older adults. First, our findings add support for the cross-cultural validity
of Euro-D depression scale in Latin American countries.

A HAI with excellent psychometric properties was created by using items of functional ability and intrinsic
capacity in a subset of six low-and-middle income countries.

There is evidence for the cross-cultural validity of the EURO-D scale at Latin American and Indian settings
and its potential applicability in comparative epidemiological studies.

DEMQOL and DEMQOL-Proxy carry the same meaning, sensitivity, and rel-
evance for respondents in the United Kingdom and Latin America.

known-groups validity analyses indicated a gradual influence of severity cat-egory and disease duration on SCOPA-PS scores (P < 0.0001). SEMvalue was 8.24 (7 to 12 in previous studies). These magnitudes may beindicative of the threshold for a real change and a minimum importantdifference

The results suggest that the mPPRS is a useful tool for evaluation of psychosis in PD. The results show that some psychometric properties of the mPPRS are satisfactory albeit there is room for the improvement of scale's content validity and internal consistency

Psychometric properties of the mPPRS are satisfactory albeit there is room for the improvement of scale's content validity and internal consistency. Mokken analysis suggests it conforms to IRT so is scalable. Similarity of goodness of fit measures of one and two factor models suggests measurement invariance across sites.

Older age and lower levels of education were consistently assoICated with poorer cognitive test performance on scores for all four tests, across all sites. The effect of sex on cognitive test performance was smaller and more variable, both between tests and between sites. Effects of age, sex and education on cognitive test performance were each significantly modified by site for all four cognitive tests. However, the effects were

GMS-AGEcat performed worse in latin america than other settings (china, india etc). Higher false postive rate in those with lower education

The scale was found to have good internal consistency indicators with as above 0.8 in most countries. When evaluated with the Rasch model, several items are identified that do not fit the model, which could be related to cultural contexts or to the fact that it is a general population.

Abstract says: "It is concluded that the Spanish version of WHOQOLOLD
is a reliable and valid questionnaire for Mexican older adults", but this conclusion is not present in the body of the text. Main key finding: Factorial structure of the tool.

The tool is validated for the target population. Internal consistency good to excellent. Better sensitivity than commonly used tool in clinical setting but lower specificity.

The tool is validated for the target population, for the screening of both MCI and Dementia. The authors highlight its high sensitivity and specificity for the screening of MCI. Education level does not severely impact its diagnostic accuracy (multinomial regression).

The tool is validated for the target population, for screening of NCD. Internal consistency and temporal stability were satisfactory regardless of the qualitative scoring method. Convergent validity is based **only one** category of the test. High diagnostic accuracy for major NCD, but particularly low sensitivity for mild NCD.

Tthe Health Perceptions Questionnaire-Revised is valid for evaluating self-perceptions of health among older Spanish speakers and could be used as a complement to the physical examination

The index of Spirituality is a valid and reliable instrument adequate to Mexican elders.

It is mentioned an EFA and correlations with other variables. However, the results of the EFA are not mentioned and the other variables correlated are not measured with any psychometric tool.ex: Depression was measured by the question Are you depressed?

internal consistency and a coherent structure,

We therefore conclude that this instrument is suitable for application in Mexican population to evaluate the quality of life construct.

The 11-items LSITA scale shows preliminary good properties of reliability and validity in Mexican elderly people.

We studied the ROSA ́s concurrent validation in patients with several dementia types, even when this scale was speICfically developed for Alzheimer ́s patients. We got similar results in patients with AD and FTD,
nevertheless ROSA ́s behavioral section correlation with NPI was only significant for the FTD group.

Accurate clinical diagnosis of MCI can be enhanced by the use of objective and structured cognitive status examinations such as the test currently used in this study.

These findings highlight the need for population-based norms for the CCCE, which has been used in population-based studies. Demogra- phic factors such as age and education must be considered when interpreting the cognitive measures.

Prevalence of depression 42% (IC95% 35-48) ICD10; 35% (IC95% 29-41) DSM-IV;36% (IC95% 30-43) Beck.BDI. criterio de referencia ICD-10 y el CESD-7 ≥ 5 fue de 87%, con un valor K = = 0.74 (p < 0.01), mientras que con el DSM-IV el grado de acuerdo fue de 83%, K = = 0.66 (p < 0.01).

Discriminated in all items of the test between cases and controls. 30.4% of controls and 8.8% of cases reported no problems in any domain

CES-DR and GDS scales have high reliability and adequate validity but the CES-DR reports higher sensitivity.

Results contributed by the psicometric analysis of both versionsof the EAE have allowed to confirm their utility in Mexicansamples. Together, the EAE-O seems to investigate the anxietybefore aging as a stage of life; as a state of being, and perhaps couldbe speICally useful on people who are not necessarily elders (oldpeople caretakers, adults between 50 and 60 years old in a franktransition towards thesocially conceived old Adulthood,

Appears useful in mexico for dementia subtypes. Good psychometric properties including reliability and Construct and/or Criterion Validity

The IMSOL Reli-gious Coping subscale proved to have the best psychometric prop-erties, as regards internal consistency, validity and factorial struc-ture for its use in research protocols dealing with older Mexicanadults. Although EBE's Relationship with God subscale resultedin two clearly defined components instead of one, it was also shownto have psychometric qualities that make it useful for research.

Based on this new version, it was estimated that 2% of men and 13% of women in this sample exhibited the symptoms required for the diagnosis of a major depressive episode according to the DSM-IV.

The final version, after its application and analysis, has six items and an adequate reliability, which shows that it is a measurement of the same construct.

The Norma Latina Battery successfully discriminated between individuals with AD and healthy controls. For this reason, it is recommended that both cliniICans and researchers use this battery in the evaluation of Mexican people with AD. In addition, the Norma Latina Battery can also be a useful tool at the rehabilitation stage as it may be used to know how effective an intervention can be for people with AD.

The factor structure of the SF-36 was analyzed through confirmatory factor analysis (CFA). The analyses show an adequate four-factor structure. The four-factor structure (Physical Function, Body Pain, Physical Role and Psychological Health) shows adequate reliability and validity indices. In addition, the results from the CFA analyses for the subsamples provide strong evidence of the stability of the four-factor structure. Future

5WT is a rapid test with neuropsychological validation for the exploration of cognitive characteristics in major NCD type MixD, regardless of age and education.

The M-ECog scale proves to be valid and reliable for measuring everyday abilities mediated by cognition. It is self-applicable without requiring extensive prior formation. It is useful to screen for SCD and MCI in older Mexican adults.

Spanish version of the 11-item DJGLS shows adequate psychometric characteristics for its use in Mexican older adults. Despite this study being conducted in Mexico, the validation of this scale shows that it could be used in other Latin American countries where Spanish is spoken.

This study highlights the importance of having a validated scale for screening depression in the elderly. This study provides an evidence for the use of GDS-15 in Ecuadorian elderly population to screen for depression.

The SF-36 showed good discrimination between groups of individuals with and without chronic diseases, and high correlation between depressive symptoms, econoMCI situation, andsocial support. Results show that the SF-36 is adequate for use in Mexican older persons. In future studies, health outcomes for this population group in Mexico may be consistently assessed using the SF-36.

The factor structure obtained in the present investigation, due to the number of items and their theoretical coherence with the original version by Lasher and Faulkender, can be considered a short and adapted version of the Anxiety about Aging Scale for use in older adults.

The MGH-SFQ is a questionnaire that provides reliable and valid measures of sexual functioning in Mexican elderly men and woman.

This study shows that three of the most common tools available to assess cognitive impairment in the elderly population have poor agreement in resource-constrained settings in a Latin America country.

The CHS-5 has excellent psychometric properties, being a measure that provides valid and reliable interpretations to be used in elderly Peruvians with depression.

The results suggest that the EAEE presents evidence of validity based on the internal structure, convergent and discriminant, as well as an adequate reliability. *Not clear the analysis of reliability

The conclusion of considering swls a measure that provides valid and reliable interpretations of ls in Peruvian ep. The results are important in so far as they can be used in the clinic ambit or as part of an ep general assessment system, or, within limits, for research purposes.

The results show that the BRCS has proved valid and reliable, supporting its use as a short measure of resilience in older Peruvians.

The combination of MMSE and PDR-M show good discriminative ability to detect moderate and severe dementia in population living in Urban community in Lima.

The PDR-M is a brief and reliable instrument for the detection of dementia in the Urban population of Lima, Peru.

The M@T is a short test with a good performance to discriminate controls, aMCI and early AD in individuals with low level of education from Urban settings.

The RUDAS-PE has acceptable psychometric properties performing well in its ability to discriminate controls from patients with MCI and ED.

They did not have a gold standard or clinical assessment but deICded who had cognitive decline based on description of daily activities at interview. MMSE score was affected by education more than any other factor ($F=167.86$, $p < 0.001$; Eta = 0.216). The optimal cut point was 23/30 but the sensitivity and specificity were best at 0-3 years of schooling in this version. The MMSE has some limitations in this setting.

Evaluated against the MMSE, the FOTOtest seemed to perform well. Clinical assessment seemed to consist of screening tools only and collateral histology was limited.

The SWLS is a valid instrument for intercultural comparisons between Spanish and Peruvian population. Comparison of latent means showed only small differences in construct between the groups

As a screening tool for the detection of dementia in elderly patients older adults with suspected Alzheimer's disease Alzheimer's disease, the 10-point clock test may be useful for use during daily clinical practice.

This was a phase one diagnostic study e.g. case control 50% test pretest probabililty. The Peruvian adapted version of the Eurotest, called prueba de la moneda peruana could be useful in screening for cognitive impairment among older adult

The combination of MMSE and PDR-M show good discriminative ability to detect moderate and severe dementia in population living in Urban community in Lima

The M@T had excellent discrimination of aMCI due to AD and dementia due to AD in Lima, Peru, a LMCI. It was also strongly correlated with CSF biomarkers and had good discrimination of neurodegeneration.

Based on its excellent psychometric properties, we find the RUDAS-PE suitable to aid in the opportune detection of dementia in a geriatric illiterate population with low-levels of education.

There was an excellent agreement between MMSE and MoCA and both tests can be used as a tool for assessment of cognitive impairment. SPMSQ cannot be recommended for the initial screening of cognitive impairment in the elderly.

Our findings suggest that a combination of tests—the Mini-SEA, r-SMS, and IFS—could improve the diagnostic and discriminative capacity of patients with cognitive impairment and behavioral symptoms.

This test is reliable, as it has the ability to determine the level of cognitive ability to determine the level of cognitive impairment in the patients tested.

This study reports that the Spanish version of the CAS applied to Peruvian older adults is a unidimensional instrument, with adequate reliability, and that it is significantly related to anxiety, depression, psychological well-being and psychological distress.

The IFS-Pe has the best diagnostic accuracy for detecting VIC and discriminating between pre-dementia (VIC-ND) and dementia (VD) stages.

the COV19-QoL is a valid measurement scale of the impact of the COVID-19 pandeMCI on the quality of life of Peruvian older adults.

It is concluded that the EMSEA battery presents evidence of criterion and Construct and/or Criterion Validity, internal consistency and a predictive.

The Spanish version of the DJGLS is a valid and reliable instrument tomeasure loneliness in Peruvian older adults, even though it presents somemethod bias assoICated to negatively worded items. Thus, the now validatedDJGLS will allow for the evaluation of loneliness in the mother language ofthe participants of this study, increase the scope of the measure and pro-vide more opportunities for a better understanding of this variable

The ADCS-ADL scale is reliable in a population with ad in Lima, Peru. Future work may validate a tool for Peruvians with lower educational levels.

The FCSRT-Picture had better performance characteristics for distinguishing controls from aMCI compared with several other BCS tools, but similar characteristics between controls and early ADD. The FCSRT-Picture is a reliable BCS tool for illiteracy in Peru.

The Spanish version of the Fear of COVID-19 Scale presents evidence of validity and reliability to assess fear of COVID-19 in the Peruvian older adult population.

The Spanish version of the WTLS provides evidence of validity and reliability for measuring well-being in older adults. The small number of items in the WTLS helps reduce boredom and fatigue assoICated with administering long tests with repetitive items.

The preliminary results back the use of the MAAS-5 as a self-report measure of mindfulness that has an adequate unifactorial structure that is reliable and invariant across gender for measuring the full attention state in elderly Peruvians

The WHOQOL-OLD Quality of Life Questionnaire has empirical evidence of validity and reliability that makes it an appropriate instrument to measure the quality of life variable.

Nevertheless, the evidence shown by this research is suffiICent to conclude that the BRCS is a short measure of resilience that has shown good psychometric properties and scalar invariance in the two countries. These results support the use of the BRCS in comparative studies of older adults in Peru and Spain.

This study confirmed that the NeuroBel is a suitable test for detecting cognitive decline based on language impairment in Spanish-speaking elderly people.

The psychometric values obtained with the Scale accredit it as a valid and reliable instrument.

The General Linear Model and logistic regressions revealed that these tests have a moderate degree of sensitivity when discriminating between the control and Alzheimer's Disease mild groups while controlling for age and years of education, but do not assist with the differential diagnosis with the other clinical groups. The predictive validity of both tests used together is comparable to the one observed when the tests are used
